# Design of a multi-epitope vaccine against the pathogenic fungi *Candida tropicalis* using an in silico approach

**DOI:** 10.1186/s43141-022-00415-3

**Published:** 2022-09-29

**Authors:** Nahid Akhtar, Arshwinder Singh, Atul Kumar Upadhyay, M. Amin-ul Mannan

**Affiliations:** 1 Department of Molecular Biology and Genetic Engineering, School of Bioengineering and Biosciences, Lovely Professional University, Phagwara, 144401 Punjab India; 2 Department of Biotechnology, Thapar Institute of Engineering and Technology, Patiala, Punjab, 147004 India; 3 Division of Infectious Disease, The Lundquist Institute for Biomedical Innovation at Harbor, University of California Los Angeles (UCLA) Medical Center, Los Angeles, CA USA

**Keywords:** Antifungals, Candidiasis, Computational biology, Epitope vaccine, Molecular simulation, Reverse vaccinology

## Abstract

**Background:**

*Candida tropicalis* causes tropical invasive fungal infections, with a high mortality. This fungus has been found to be resistant to antifungal classes such as azoles, echinocandins, and polyenes in several studies. As a result, it is vital to identify novel approaches to prevent and treat *C. tropicalis* infections. In this study, an in silico technique was utilized to deduce and evaluate a powerful multivalent epitope-based vaccine against *C. tropicalis*, which targets the secreted aspartic protease 2 (SAP2) protein. This protein is implicated in virulence and host invasion.

**Results:**

By focusing on the Sap2 protein, 11 highly antigenic, non-allergic, non-toxic, and conserved epitopes were identified. These were subsequently paired with RS09 and flagellin adjuvants, as well as a pan HLA DR-binding epitope (PADRE) sequence to create a vaccine candidate that elicited both cell-mediated and humoral immune responses. It was projected that the vaccine design would be soluble, stable, antigenic, and non-allergic. Ramachandran plot analysis was applied to validate the vaccine construct’s 3-dimensional model. The vaccine construct was tested (at 100 ns) using molecular docking and molecular dynamics simulations, which demonstrated that it can stably connect with MHC-I and Toll-like receptor molecules. Based on in silico studies, we have shown that the vaccine construct can be expressed in *E. coli*. We surmise that the vaccine design is unrelated to any human proteins, indicating that it is safe to use.

**Conclusions:**

The vaccine design looks to be an effective option for preventing *C. tropicalis* infections, based on the outcomes of the studies. A fungal vaccine can be proposed as prophylactic medicine and could provide initial protection as sometimes diagnosis of infection could be challenging. However, more in vitro and in vivo research is needed to prove the efficacy and safety of the proposed vaccine design.

**Supplementary Information:**

The online version contains supplementary material available at 10.1186/s43141-022-00415-3.

## Background

Infections caused by fungi are on the rise all over the world [1]. The number of people who are susceptible to fungal infections is growing, especially among the elderly and those who are immunocompromised [2]. Fungal infections are expected to impact more than 1 billion individuals worldwide each year [3]. The majority of these infections are superficial and easy to treat, but roughly 150 million cases might be serious or life-threatening to individuals [4]. Every year, invasive fungal infections kill over a million people around the world [5]. Human fungal diseases are also a major cause of socio-economic burden. So far, several billion dollars have been utilized in combatting fungal infections or their associated comorbidities [6]. Dissatisfactory outcomes and adverse effects of commonly utilized antifungal medicines increase the public health concern posed by fungal infections [5]. The emergence of resistance to important antifungal medication classes in numerous species of human fungal diseases belonging to genera such as *Candida*, *Aspergillus*, and *Cryptococcus* is another cause for concern [7–9]. As a result, new methods for treating fungal infections are needed. Vaccine development could be one of the strategies for combating harmful human fungal illnesses. Although in mouse models, various vaccine constructs have shown protection against different pathogenic fungi, no vaccine has been approved against fungal infections so far in spite of the grave public health threat posed by the fungal infections [10]. The major limitations in developing vaccines against fungi are diverse infection sites in hosts, intraspecies and interspecies antigenic variations, difficulty in translation from animal models to humans, and lack of commercial interests [10]. Furthermore, the individuals with a high risk of fungal infections are immunocompromised; hence, these individuals are less likely to respond to subunit or inactivated whole organism vaccination because of their debilitated immune status [11]. Hence, it is necessary to look for alternative strategies to develop safe and effective vaccines against fungal infections.

Using in silico approaches, the secreted aspartic protease 2 (SAP2) protein of *C. tropicalis* is targeted for creating a safe and effective vaccination. It is considered to be the second most virulent and third most prevalent *Candida* species worldwide especially in Asia and South America [12]. *C. tropicalis* mostly infects patients admitted in intensive care units and those suffering from neutropenia [13]. The high mortality rate of *C. tropicalis* infections is one of the key concerns. According to various research, *C. tropicalis* infections have a death rate of more than 40%, most of which are owing to predisposing factors and resistance to the existing drugs [14, 15]. Different studies have reported the emergence of antifungal drug resistance to drugs such as caspofungin, amphotericin B, and fluconazole in various clinical isolates [16–18]. In a study in Iran, 64 *C. tropicalis* bloodstream isolates were obtained and they were found to be resistant to itraconazole (2/64), micafungin (2/64), fluconazole (4/64), voriconazole (7/64), pan-azole (1/64), and fluconazole + voriconazole (2/64) treatment [19]. The study also reported a very high mortality rate of 60% suggesting a possible role of antifungal drug resistance in a high mortality rate of patients infected with *C. tropicalis* [19]. Furthermore, the emergence of drug resistance in *C. tropicalis* poses a higher risk of clinical infections, poor clinical outcomes for patients such as prolonged stay at hospital and mortality [9]. Emergence of drug resistance is associated with high morbidity and mortality in patients suffering from fungal infections [20]. Because of its high mortality rate and the evolution of antifungal medication resistance, *C. tropicalis* infections constitute a severe concern to public health. As a result, innovative medicines to manage and treat *C. tropicalis* infections are critical.

The secreted aspartic protease (*SAP*) gene family consists of 10 isoenzymes (SAP1-10) [21]. These SAP proteins are extremely virulent, aiding in the infection of both humans and animals [22]. They break down various cell proteins, disintegrate cell membranes, and destroy the host extracellular matrix, assisting the fungi in penetrating the host [21, 22]. They are also responsible for phenotypic switching, hypha formation, and adherence to host cells [22]. SAP2 protein specifically helps in evading host complement system attack, causes tissue damage, modifies cytokine response, and induces inflammatory response [23–26]. Sap2p has been previously targeted for developing a vaccine against *Candida*. According to Bernardis et al., intranasal and intravaginal immunization of rat candida vaginitis models with SAP preparations that largely consisted of Sap2p promoted the release of anti-SAP antibodies and offered protection against fungus infection [27]. In mouse vaginitis models, the recombinant vaccines PEV7 and rSap2t, both made of Sap2p, have also been demonstrated to induce an immune response and protective effects [28, 29]. A hybrid phage vaccine displaying SLAQVKYTSASSI epitope and recombinant Sap2p-induced potent humoral and cell-mediated immunity against *C. albicans* in mouse models [30]. The Sap2p has been evaluated as possible vaccine candidates against other *Candida* species also. Immunization of *C. tropicalis*-infected mice with recombinant Sap2p obtained from *C. parapsilosis* increased anti-Sap2 antibody production and the survival of infected mice [31]. SAP2 protein has been demonstrated to confer immunization against fungal infections in a variety of species; therefore, it could be a promising option for developing fungal infection vaccines. As per our understanding, no in silico approach has been used to study Sap2p as a target for developing a *C. tropicalis* multi-epitope peptide vaccine construct. Hence, this study aims to develop a novel and effective vaccine candidate against *C. tropicalis* by targeting the SAP2 protein using computational techniques. We have previously demonstrated the success of the same procedure in creating a vaccine candidate against the fast-evolving and highly pathogenic *C. auris* utilizing an in silico approach [32].

The Sap2p of *C. tropicalis* was analyzed using different webservers to anticipate various highly antigenic and safe B cell and T cell epitopes. To create the final vaccine design, the highly antigenic and safe epitopes were connected with two adjuvants, RS09 and flagellin protein, using a GGS linker. The final vaccine design’s physiochemical characteristics, antigenicity, solubility, and allergenicity were all analyzed. The vaccine construct’s secondary and tertiary structure was predicted next, followed by a Ramachandran plot to validate the vaccine construct’s 3D model. The vaccine’s ability to bind with Toll-like receptor (TLR) and major histocompatibility complex (MHC) molecules was predicted using molecular docking and molecular dynamics simulation. Finally, an in silico cloning experiment was carried out to confirm the vaccine construct’s capacity to be cloned in a vector suitable for commercial manufacturing on a wide scale. We believe that the present study can be employed by other researchers working on developing novel strategies to control *C. tropicalis* infections.

## Methods

### Protein sequence retrieval and analysis

Sap2p protein sequence of *C. tropicalis* (protein ID: AAD33216.1_1) was retrieved from European Nucleotide Archive. The Sap2p subcellular localization was determined using the CELLO2GO [33]. To determine the subcellular localization of the query proteins, this webserver uses a combination of BLAST homology searches and CELLO localization algorithms [33]. The CELLO2GO was used in this study because it can predict the subcellular localization of proteins with high accuracy (98.4% for the archaeal sequences, 99.1% for the Gram-negative bacterial, and 99.4% for the Gram-positive bacterial). Furthermore, it can predict the subcellular localization of proteins even when the homologs have not been predicted by BLAST or gene ontology annotations are less in number [33]. The localization of protein was also performed with WoLF PSORT (an updated version of PSORT II that predicts the subcellular localization of eukaryotic proteins) to improve the accuracy of the subcellular localization prediction [34]. Furthermore, a good vaccine candidate should trigger a significant immune response against the disease. Hence, the protein’s antigenicity was predicted via the Vaxijen 2.0 website [35]. Vaxijen was used because it uses an alignment-free approach based on auto-cross-covariance (ACC) transformation of protein sequences into uniform vectors of principal amino acid properties and can predict the antigenic peptides with the accuracy of 70–89% [35]. A fungal protein must have a Vaxijen score of 0.5 to be antigenic. To determine fungal, viral, bacterial, and tumor antigenic proteins, this server uses an auto-cross-covariance technique. Using the NCBI-BLASTp program, the homology and conservancy of Sap2p were assessed [36].

### Prediction of T and B cell epitopes

The NETMHC 2.3 website was used to predict T helper cell epitopes that might bind to MHC class II molecules [37]. The webserver utilizes an artificial neural network (ANN). The NETMHC 2.3 webserver was used because in previous studies where different tools for predicting peptides binding to MHC were compared, it was found to be better than other tools such as PickPocket, PRPPRED, MULTIPRED, ADT, and KISS [37–39]. T-cytotoxic cell epitopes that might bind to MHC class I molecules and elicit a cellular immune response were predicted using the NETMHC 4.0 website [40]. This server uses a machine learning method and predicts epitopes that may interact with different animal and human MHC class I alleles with 98% specificity [40]. HLA alleles DRB1_0101, DRB1_0301, DRB1_0401, DRB1_0701, DRB1_0801, DRB1_0901, DRB1_1001, DRB1_1101, DRB1_1201, DRB1_1301, DRB1_1501, and DRB1_1602 were selected while predicting T helper cell epitopes. HLA alleles HLA-A0101, HLA A0201, HLA-A0301, HLA-A2402, HLA-A260, HLA-B0702, HLA-B0801, HLA-B2705, HLA-B3901, HLA-B4001, and HLA-B5801 HLA were selected while predicting the T cytotoxic cell epitopes. Multiple linear B cell epitopes were predicted using the IEDB B cell epitope prediction website’s Bepipred linear epitope prediction 2.0 approach [41].

### Determination of the predicted epitopes’ toxicity, antigenicity, allergic potential, and interferon-γ activating potential

The epitopes that are non-allergic, non-toxic, and highly antigenic can aid in the development of an effective and safe vaccine [42, 43]. The ToxinPred web service was used to determine the toxicity of the proposed B and T cell epitopes, which uses a support vector machine (SVM)-based approach to predict peptide toxicity [44]. The ToxinPred webserver was used because it can predict the toxicity of peptides with 94.5% accuracy [44]. Vaxijen 2.0 website was used to determine the antigenic properties of the epitopes [35]. This webserver predicts if the bacterial, fungal, viral, and tumor peptides are antigenic or not by evaluating various physiochemical properties of the proteins using an auto-cross-covariance method [35]. The AllergenFP version 1.0 webserver which applies the Tanimoto coefficient was used to predict if the epitopes are allergic or not [45]. The AllergenFP webserver was used because in comparison with different tools such as AlgPred, AllerTOP, AllerHunter, and APPEL, AllernFP was found to be most accurate in identifying both allergens and non-allergens [45]. The capacity of epitopes to trigger interferon production was tested using the IFNepitope online tool. This online application employs machine learning to design and forecast peptides that could activate interferon-γ [46].

### Epitope conservancy analysis

IEDB Epitope Conservancy Tool projected epitope conservancy among distinct *C. tropicalis* strains. The predicted epitopes’ conservation was tested in Sap2p of *C. tropicalis* strains ZRCT61, ZRCT01, and ZRCT54. The Sap2p of the ZRCT61, ZRCT01, and ZRCT54 strains has the accession numbers AXK68742.1, AXK68682.1, and AXK68735.1, respectively. The selection of epitopes that are conserved among different *C. tropicalis* strains will help to overcome limitations posed by antigenic shift and antigenic drifts. The epitopes that were 100% conserved (the sequence identity of the epitopes had a 100% match) among these *C. tropicalis* strains were chosen for further investigation as they have less probability of future mutations [47].

### Designing the vaccine construct and determining its physiochemical properties

For vaccination against *C. tropicalis*, epitopes that were discovered to be conserved, non-toxic, highly antigenic, non-allergic, and capable of triggering interferon production were chosen. RS09 (APPHALS) and the N and C terminals of *Salmonella dublin* flagellin protein were employed as adjuvants in the final vaccination (UNIPROT ID: Q06971). These adjuvants, in combination with the PADRE sequence, were previously employed in the development of epitope-based vaccines against dengue virus, human cytomegalovirus, and *Candida auris* [27, 47]. The PADRE sequence contributes to the vaccine construct’s stability [48]. Finally, the GGS linker sequence was used to connect all of the detected epitopes, adjuvants, and PADRE sequences. The ProtParam (ExPASy) web service was used to evaluate the vaccine design’s physiochemical characteristics [49].

This website aids in the prediction of several properties of query proteins, including isoelectric point, number of amino acids, stability, amount of positively and negatively charged amino acids, molecular weight, half-life, extinction coefficient, aliphatic index, and other features [49]. For the prediction of surface accessible amino acids in the final vaccine candidate sequence, NetSurfP 2.0 webserver available at https://services.healthtech.dtu.dk/service.php?NetSurfP-2.0 was used. The candidate vaccine sequence in FASTA format was provided as input. This server uses an artificial neural network trained using various experimentally determined protein structures to determine whether the amino acids are buried or exposed residues [50]. The final vaccine construct’s solubility was assessed using the Solpro online program [51]. After being expressed in *E. coli*, this online program predicts the solubility of proteins with a 74% accuracy [51]. Using the Vaxijen 2.0 and the AllergenFP version 1.0 webserver, the antigenicity and allergenicity of the final *C. tropicalis* vaccine design were predicted [35, 45].

### Prediction of the final vaccine’s structure

PSIPRED was used to predict the vaccine’s secondary structure. This method uses feed-forward neural networks to predict various protein secondary structure properties such as coils, alpha helices, and beta sheets [52]. The 3D model of the final vaccination was created using the I-Tasser website, which combines iterative template-based fragment assembly simulations and different threading algorithms to predict protein tertiary structure [53]. The I-Tasser webserver was used because it has been highly ranked by the Critical Assessment of Techniques for Protein Structure Prediction (CASP), a community-wide experiment for testing the state-of-the-art of protein structure predictions [53]. The PROCHECK was then utilized to verify the proposed final vaccine construct’s tertiary structure. This server helps to make a Ramachandran plot, which can be used to assess the quality of a query protein’s predicted 3D model [54]. The PDB file of the 3D model of protein predicted by I-Tasser was used as input.

### Molecular docking of the vaccine construct with HLA and TLR molecules

The online protein–protein docking service ClusPro 2.0 was used to dock the final vaccine design with the Toll-like receptor TLR5 molecule and MHCII HLA DRB 0101 [55]. The ClusPro webserver was used because it has been ranked ahead of other docking tools like HADDOCK, SWARMDOCK, and GRAMM-X by CAPRI (Critical Assessment of Predicted Interactions) evaluation meetings [55]. The PDB ID for TLR 5 and HLA DRB_0101 molecules are 3J0A and 4AH2, respectively. By identifying the centers of densely populated clusters of low-energy docked structures, the ClusPro website generates ten models of docked complexes [55].

### Molecular simulation studies

Protein structure and related information were procured from the RCSB database available at (https://www.rcsb.org/) [56]. For the prediction of the possible active site of protein receptors, the CASTp website was employed [57]. In the CASTp webserver, the PDB ID of the receptors TLR5A (PDB: 3J0A) and HLA DR1 (PDB: 4AH2) receptors were utilized as input. After the output were obtained, the “show pockets” option was clicked and the pocket ID with the most volume was selected. After selecting the pocket with the highest volume, the “update” button was clicked which then showed the active site residues of the receptors. GROMACS 2020.1 was used to do molecular dynamics (MD) simulations of the best-docked conformation with the most negative energy [58]. Protein–protein elements make up the simulation system. The CHARMM36 force field was used to do all of the MD simulations. The topology was created using the official CHARMM General Force Field server (CGenFF). The protein–protein complex was encased in a triclinic box, which was then solvated with water model SPC216. 16 NA + ions were used to neutralize the system. Position restraint was used after energy minimization, followed by NVT and NPT equilibrium. NVT (constant number of particles, volume, and temperature) was performed on protein–protein and water-ion coupling groups at 300 K and 0.1 ps coupling constant for 100 ps, whereas NPT (constant number of particles, volume, and temperature) was performed on protein–protein and water-ion coupling groups at 300 K and 0.1 ps coupling constant for 100 ps.

### In silico cloning and codon use adaption parameters

The JCat website was used to optimize the codons of the final vaccine construction gene for expression in *E. coli* K12 strain to ensure effective cloning and expression of the vaccine construct [59]. As stated by Hasan et al., rho-independent transcription terminators, bacterial ribosomal binding sites, and cleavage sites of various restriction enzymes were eliminated during the codon optimization of the vaccine design [48]. Finally, in silico cloning was performed using the SnapGene restriction cloning module. During in silico cloning, the codon-optimized vaccine construct’s sequence was sandwiched between the pET28a( +) vector’s XhoI (158) and EcoRI (192) restriction sites.

## Results

### Retrieval and analysis of the protein sequence

The Sap2p was subjected to a protein BLAST analysis to determine its similarity with diverse human proteins. Sap2p has fewer than 30% homology with human proteins, according to the findings (Supplementary Table S4). The CELLO2GO server identified the protein as an extracellular or plasma membrane protein. The WoLF PSORT tool also predicted the protein as extracellular. The protein was predicted to be antigenic by the Vaxijen server (Vaxijen score: 0.8735). These findings suggested that the SAP2 protein could be a good target for vaccine development.

### T cell and B cell epitope prediction

Altogether, 53 T helper cell epitopes were predicted as strong binders to the selected HLA alleles. The predicted strong binder T helper cell epitopes have been listed in Supplementary Table S1. Similarly, 45 T cytotoxic epitopes were predicted as strong binders. The predicted strong binder T cytotoxic cell epitopes have been listed in Supplementary Table S2. The peptides that were predicted as weak binders were not considered for further analysis. Thirteen linear B cell epitopes were predicted out of which only six were selected for further analysis as they had 9 or more residues. In Supplementary Table S3, the selected B cell epitopes predicted from SAP2 protein are listed.

### Determination of the anticipated epitopes’ toxicity, antigenicity, and allergic potential

Conservancy, toxicity, antigenicity, allergenicity, and interferon-activation ability were all evaluated for the anticipated epitopes. Supplementary Tables S1, S2, and S3 detail the attributes of T helper, T cytotoxic, and B cell epitopes, respectively. Epitopes with a Vaxijen score greater than 1.1 were chosen for additional investigation because they are thought to be particularly antigenic [60, 61]. Antigenic, non-allergic, and non-toxic epitopes were found, and additional examination with the Epitope Conservancy Tool revealed that 11 of the 14 epitopes analyzed were conserved among the *C. tropicalis* strains. Three of the 11 epitopes were thought to have the capacity to activate interferons. The 11 conserved epitopes are included in Table 1 along with their antigenicity, allergic potential, toxicity, and ability to activate interferon.

**Table 1:** List of final epitopes selected for vaccine engineering along with their properties

Epitope type	Epitope sequence	Vaxijen score	Antigenic/non-antigenic	Allergic/non-allergic	Toxin	Interferon-γ activation ability
T helper	YNRPIGAYI	3.0712	Antigenic	Non-allergic	Non-toxin	Yes
T helper	FTIQTNSAT	2.1036	Antigenic	Non-allergic	Non-toxin	No
T helper	EFTIQTNSA	2.1227	Antigenic	Non-allergic	Non-toxin	No
T helper	ILYGENFNI	4.2936	Antigenic	Non-allergic	Non-toxin	No
T cytotoxic	QELGKSFNI	4.9939	Antigenic	Non-allergic	Non-toxin	Yes
T cytotoxic	GLMGNFFDK	1.7030	Antigenic	Non-allergic	Non-toxin	Yes
T cytotoxic	KYTGSLTTL	2.3614	Antigenic	Non-allergic	Non-toxin	No
T cytotoxic	DTVGINGAI	1.9910	Antigenic	Non-allergic	Non-toxin	No
T cytotoxic	LPLTSNREF	2.7926	Antigenic	Non-allergic	Non-toxin	No
B cell	AKYTGSLTTL	2.0617	Antigenic	Non-allergic	Non-toxin	No
B cell	IGGDITYNRPIGAYIWSCNRNGK	2.1725	Antigenic	Non-allergic	Non-toxin	No

### Designing the vaccine construct and determining its physiochemical properties

**Fig. 1:**
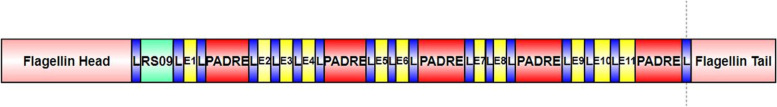
Map of the vaccine construct: E1 to E11 are the selected epitopes. L is a GGS linker. Flagellin head and tail are shown at the N and C terminal ends. RS09 and PADRE sequence are placed in between E1 and E11

The 11 conserved epitopes were employed in vaccine development because they were highly antigenic, non-toxic, and non-allergic. Adjuvants RS09 (APPHALS) and *Salmonella dublin* flagellin protein along with the selected epitopes and PADRE sequence (AKFVAAWTLKAAA) were linked together by a GGS linker to design the final vaccine construct. The map of the final vaccine construct is shown in Fig. 1 which was made by Illustrator for Biological Sequences web server [62]. The amino acid sequence of the complete vaccine construct is MAQVINTNSLSLLTQNNLNKSQSALGTAIERLSSGLRINSAKDDAAGQAIANRFTANIKGLTQASRNANDGISIAQTTEGALNEINNNLQRVRELAVQSANSTNSQSDLDSIQAEITQRLNEIDRVSGQTQFNGVKVLAQDNTGGSAPPHALSGGS**YNRPIGAYI**GGSAKFVAAWTLKAAAGGSS**FTIQTNSAT**GGSG**EFTIQTNSA**GGS**ILYGENFNI**GGSAKFVAAWTLKAAAGGS**QELGKSFNI**GGS**GLMGNFFDK**GGSAKFVAAWTLKAAAGGS**KYTGSLTTL**GGS**DTVGINGAIGGS**AKFVAAWTLKAAAGGS**LPLTSNREF**GGS**AKYTGSLTTL**GGS**IGGDITYNRPIGAYIWSCNRNGK**GGSAKFVAAWTLKAAAGGSLGNTVNNLTSARSRIEDSDYATEVSNMSRAQILQQAGTSVLAQANQVPQNVLSLLR. The vaccine construct’s instability index score was projected by the ExPASy ProtParam online service to be 29.17, implying that the vaccine construct is stable, and the negative GRAVY score (− 0.166) implying that the vaccine construct is hydrophilic [49]. Using the Solpro web server, the vaccine construct was also determined to be soluble following expression in *E. coli* [35]. The vaccine construct’s various physicochemical properties are listed in Table 2. The relative surface accessibility of the amino acid residues in the epitope of the designed vaccine candidate as predicted by NetSurfP 2.0 is provided in Supplementary Table S5. In the two epitopes LPLTSNREF and DTVGINGAI, all of the residues were exposed while in other epitopes the residues were mixed consisting of both exposed and buried residues.

**Table 2:** Physiochemical properties of the *C. tropicalis* vaccine construct

Molecular weight	44,915.80
Antigenicity (Vaxijen sever)	Antigen (Vaxijen score: 1.5419)
Allergenicity	Non-allergen (AllergenFP)
Number of amino acids	441
Theoretical pI	9.71
Instability index	29.17 (stable)
Total number of atoms	6277
Aliphatic index	79.84
Extinction coefficient	44,920
Grand average of hydropathicity (GRAVY)	− 0.166
Estimated half-life	> 20 h in yeast, > 10 h in *E. coli*, and 30 h in mammalian reticulocytes
Solubility	Soluble (Solpro)

### Structure prediction of the final vaccine construct and its validation

**Fig. 2:**
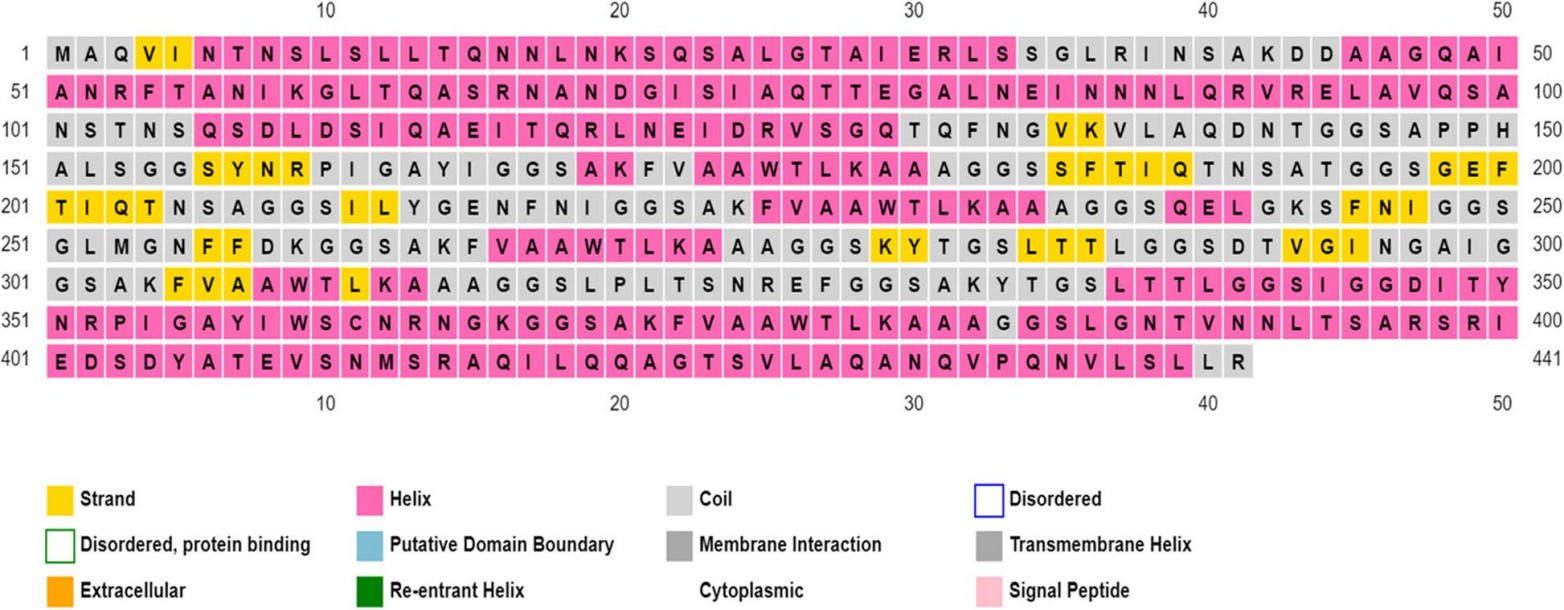
Secondary structure prediction of the vaccine constructs: The strands are represented in yellow, the helix sections are shown in pink, and membrane contact and transmembrane helix regions are shown in gray color

**Fig. 3:**
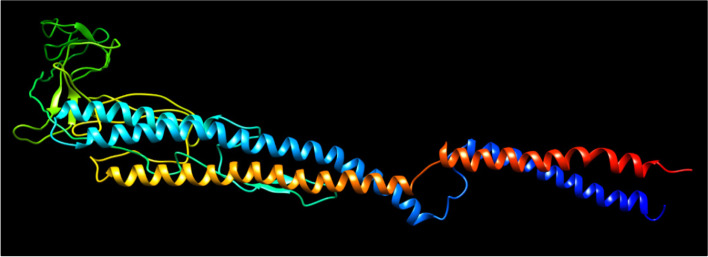
The I-Tasser server anticipated the vaccine construct’s tertiary structure

**Fig. 4:**
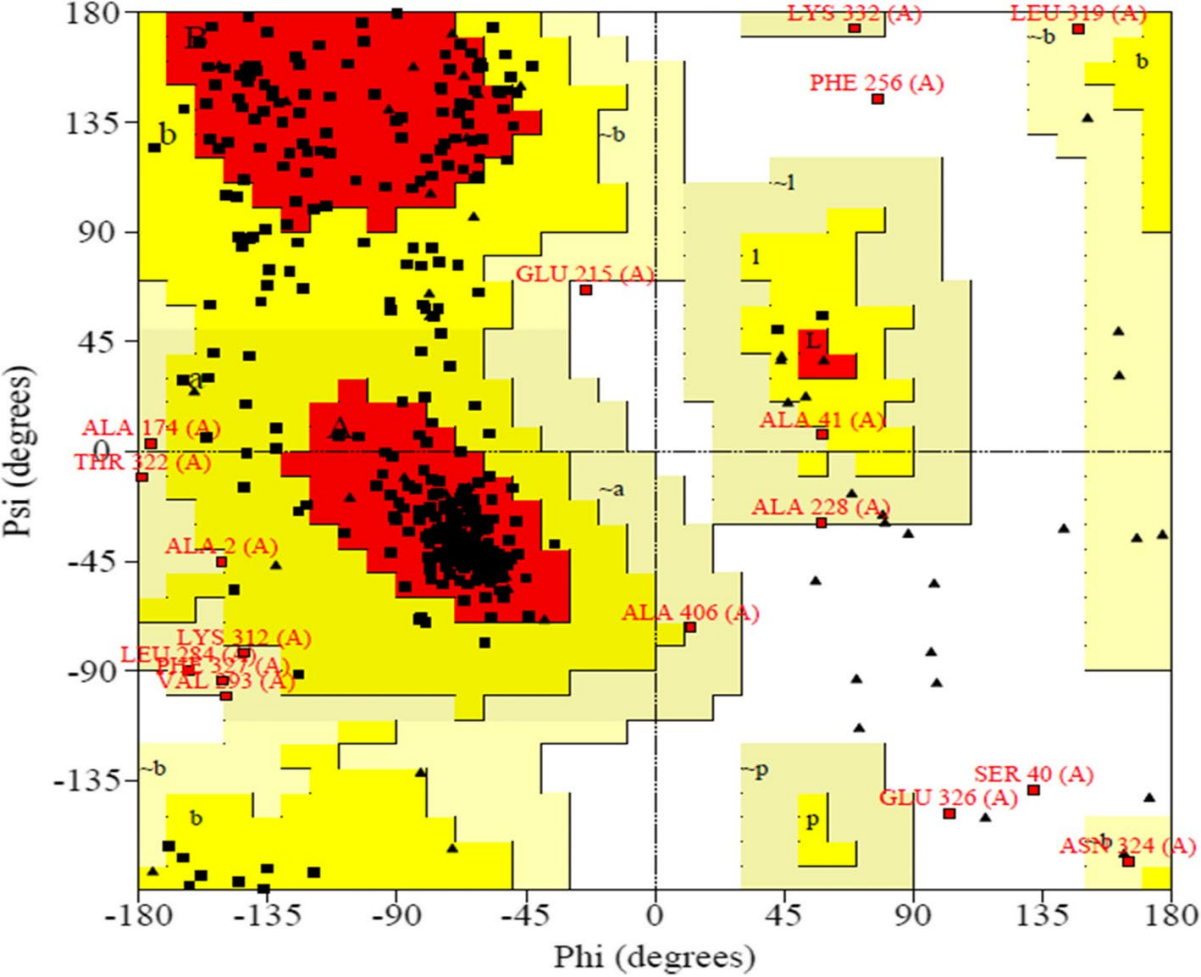
Ramachandran plot, PROCHECK tool generated a 3D model of the vaccine construct

The presence of all three types of secondary structure elements was predicted by the PSIPRED webserver’s secondary structure prediction: helix, coils, and strands (Fig. 2). Figure 3 depicts the tertiary structure of the final vaccine construct predicted by the I-Tasser server. The vaccine construct’s estimated 3D model has a C-score of 0.19. The RMSD value of the best model predicted by I-Tasser is 6.6 ± 4.0. The PROCHECK server validates protein structure in terms of energetically allowed and disallowed dihedral angles psi (ψ) and phi (ϕ) of amino acid. Out of all the residues, 83.6% of residues were in the most favored regions, 15% residues in the additional allowed region, and 0.8% residues were in the generously allowed region. Only 0.5% residues were present in disallowed regions according to the Ramachandran plot. Furthermore, the tertiary model of the vaccine candidate has 60 proline residues (represented as triangles in Fig. 4) and 6 proline residues.

### Molecular docking analysis of the final vaccine construct with HLA and TLR molecules

**Fig. 5:**
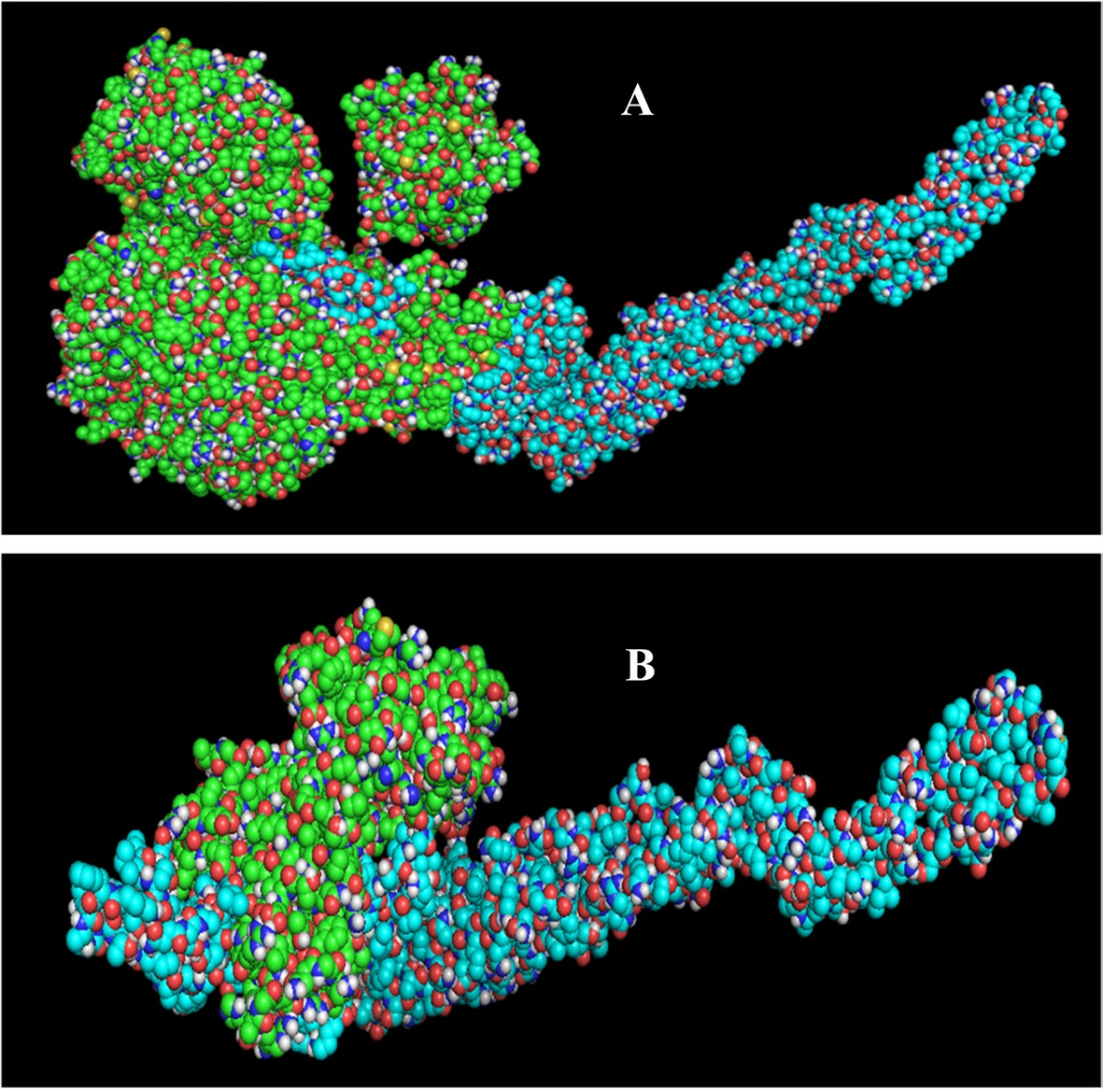
Molecular docking analysis: **A** TLR5 receptor docking with the *C. tropicalis* vaccine design (blue hue) (green). **B** HLA allele docking with the *C. tropicalis* vaccine construct (blue) (green in color)

The ClusPro 2.0 web server was used to dock the final vaccine construct with TLR5 (3J0A) and the MHC class II allele HLA DRB 0101 (PDB:4AH2). Following docking, 29 models were produced for both the vaccine construct docking with TLR5 molecules and the vaccine construct docking with HLA DRB 0101. The energy exchanged between the vaccine design and 3J0A was − 1609.2 kcal/mol. The energy of the vaccine construct and 4AH2 interaction was − 1178.4 kcal/mol. The vaccine construct interacts with TLR5 and HLA DRB 0101, as demonstrated in Fig. 5.

### Molecular dynamics simulation

**Fig. 6:**
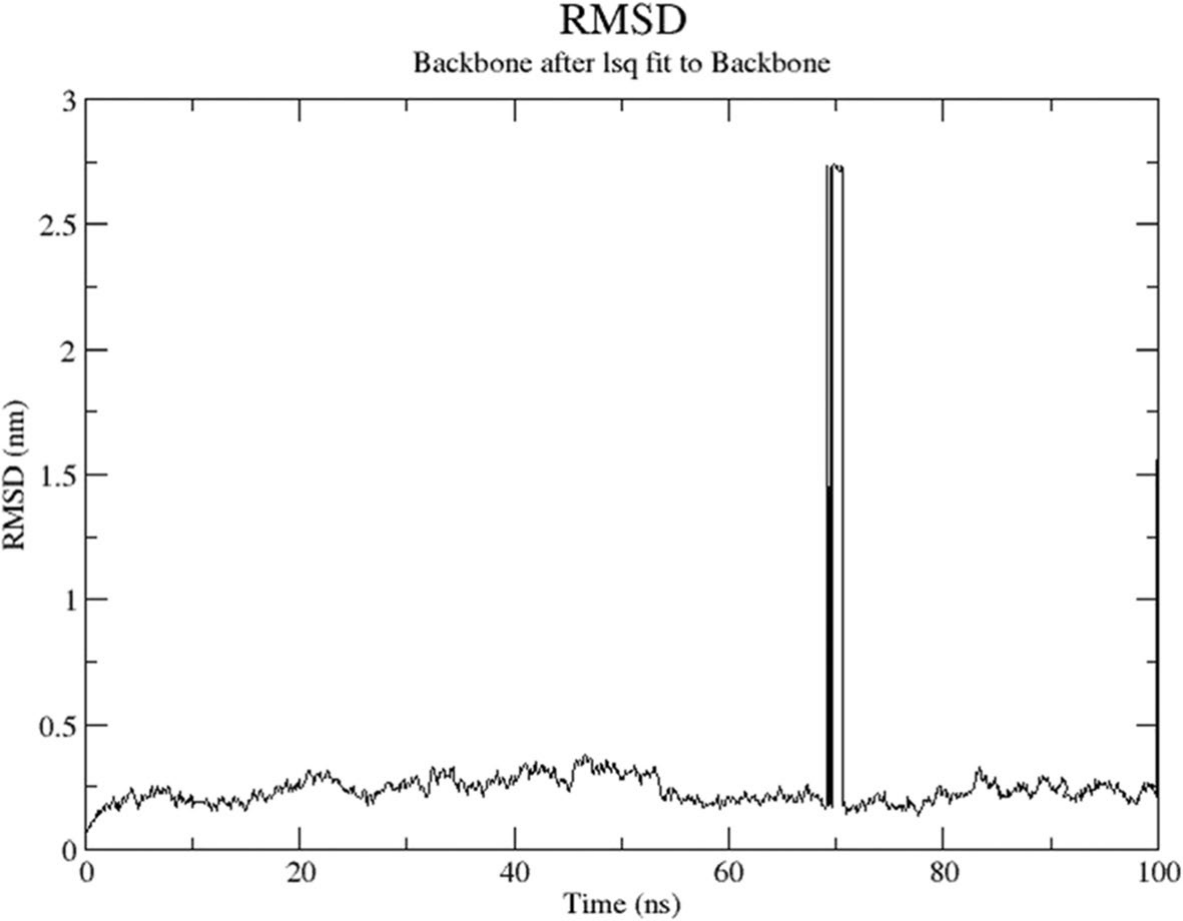
RMSD values of the docked complex of TLR5A (PDB: 3JOA) and the designed construct during molecular simulation analysis at the 100-ns time scale

**Fig. 7:**
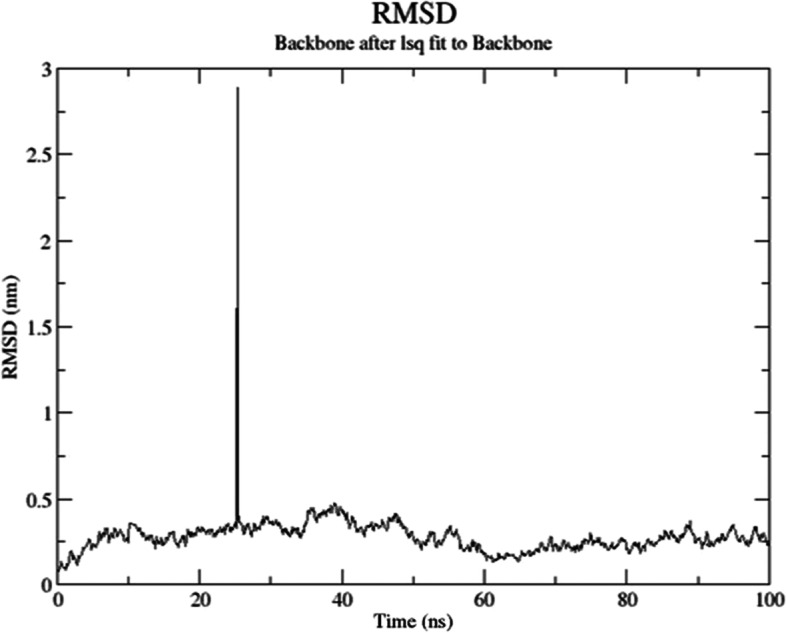
RMSD values of the docked complex of HLA DR1 (PDB: 4AH2) and the designed construct during molecular simulation analysis at the 100-ns time scale

The root mean square deviation was used to assess the protein–protein complex’s conformational stability during MD simulation (RMSD). Backbone atoms’ RMSD was calculated. TLR5A’s standard deviation (RMSD) (PDB: 3JOA) (Fig. 6) steadied after 0.5 ns until 70 ns when there was a rapid rise. HLA DR1 (PDB: 4AH2) (Fig. 7) was steady throughout the simulation, and the system kept within the RMSD window of 0–0.5 nm. The RMSD continuously grew until it reached 0.5 ns, after which it stabilized. At around 25 ns, the RMSD spikes, but the system quickly stabilizes and stays that way until the simulation is finished. After that, the value remained stable within a 25-nm range until the simulation ended.

### Codon usage adaptation and in silico cloning

**Fig. 8:**
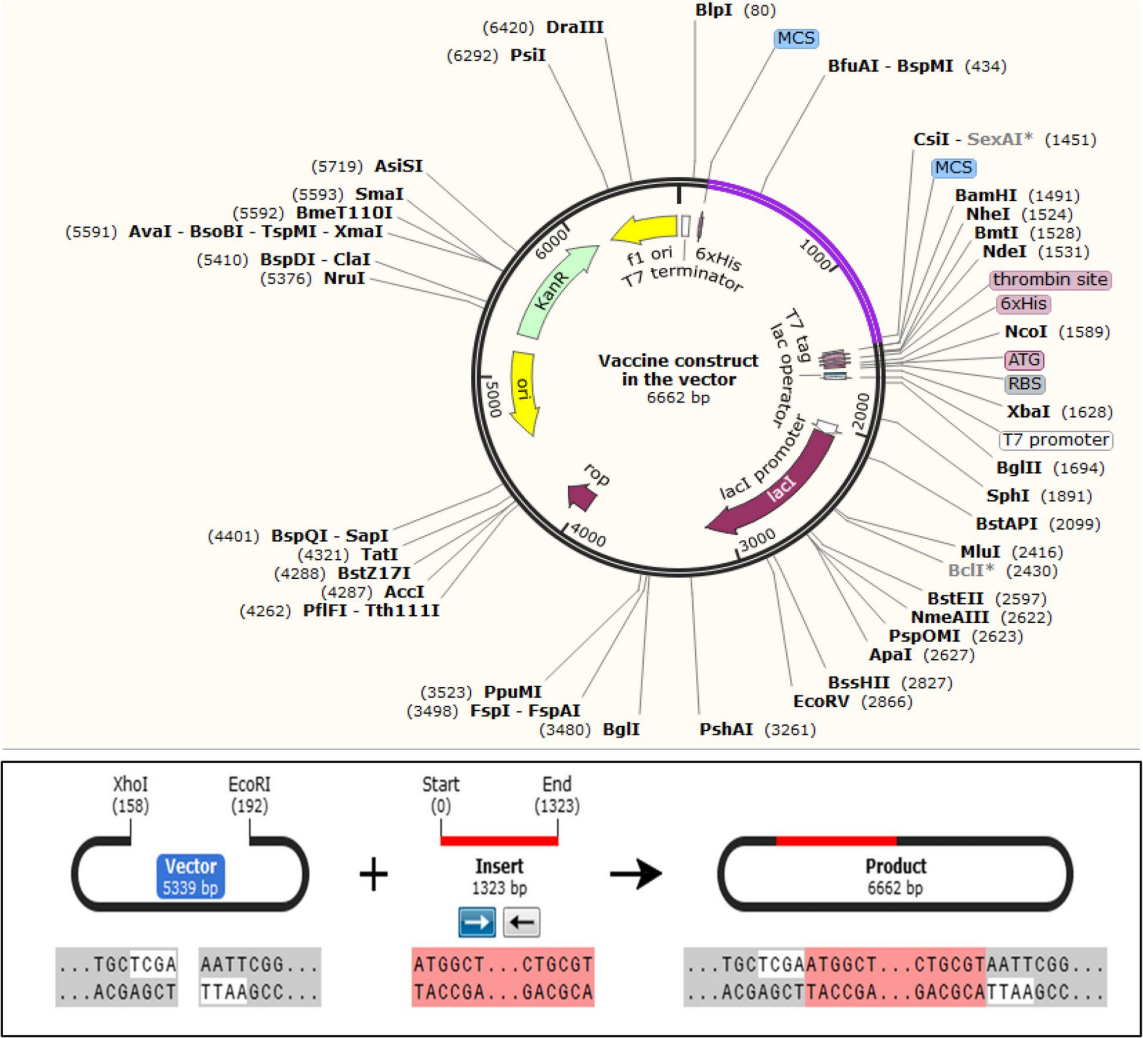
In silico cloning of the designed vaccine construct in the pET28a ( +) vector

The vaccine design’s codon use adaptation is required to speed up the expression of the vaccine construct in prokaryotic hosts and ensure large-scale commercial production. The vaccine construct’s Codon Adaptation Index was 0.9943 after the JCat website codon adaptation, indicating a high degree of sequence expression. The codon-adapted vaccine sequence has 51.77% GC content. The vaccine construct was inserted between XhoI (158) and EcoRI (192) restriction sites of the pET28a ( +) vector. The final cloned vaccine construct is demonstrated in Fig. 8 and the inserted vaccine construct is displayed in purple color. Furthermore, we were able to express vaccine construct with C-terminus histidine in the pET28a system in *E. coli* host and found that the desired construct was expressed as a soluble fraction (data not shown).

## Discussions

*C. tropicalis* is a non-albicans *Candida* (NAC) species that is frequently isolated from urine and blood samples of patients in intensive care units, catheterized, cancer, and neutropenia patients [63]. One of the significant issues connected with *C. tropicalis* infections is antifungal resistance [64]. Another source of worry is the high mortality rate associated with *C. tropicalis* infections [14, 15]. As a result, it is critical to explore new ways to combat *C. tropicalis* infections. In this study, an in silico technique was employed to create and evaluate a novel and powerful multivalent epitope-based vaccine against the pathogen. Previously, vaccinations such as NDV-3 and PEV7 were developed and tested against various *Candida* species. In mice models, these vaccinations were proven to prevent *C. auris* and *C. albicans* infections [28, 65]. The details of these and other anti-candida vaccines have been aptly discussed in the review by Tso et al. [66]. The immunization of *C. tropicalis*-infected mice with recombinant SAP2p isolated from *C. parapsilosis* has been shown to enhance the secretion of anti-SAP2 antibodies and survival of the infected mice [31]. However, the in silico-designed vaccine against *C. tropicalis* has not been reported so far. The popularity of in silico-developed epitope-based vaccines has risen recently. The computational method could be a speedy and low-cost option to develop vaccinations that elicit a significant immunological response [67, 68]. Antigenic shift, antigenic drift, and genetic variants are all issues that can be dealt with that epitope-based vaccinations [67, 69]. Candidate vaccines against SARS-CoV-2, human papillomavirus, Oropouche virus, Ebola virus, Lassa virus, Nipah virus, Zika virus, dengue virus, canine circovirus, and human cytomegalovirus have all been developed using an in silico technique [42, 60, 61, 70–77]. Similarly, the in silico approach has been used to design candidate vaccine against fungal pathogens such as *C. auris* and *C. albicans* [32, 78]. Akhtar et al. predicted 8 epitopes namely FTSSSNTLQ (T helper), SYQATVSFS (T helper), GTDTLVIEV (T cytotoxic), RPYININAA (T cytotoxic), SSYQATVSF (T cytotoxic), NAGSTSDEVNL (linear B cell), RTWTGSVTTTETLTAPSGGTE (linear B cell), and PTPVTTITKTWTGSVTTTETIPAPSGGTET (linear B cell) by targeting Als3p of *C. auris* which were used to design candidate vaccine using in silico method [32]. Tarang et al. predicted 18 epitopes by targeting 8 proteins involved in hypha formation namely Als4p, Als3p, Fav2p, Als2p, Eap1p, Hyr1p, Hwp1p, and Sap2p proteins of *C. albicans* which were then linked by AAY and GPGPG linkers to design a 349 amino acid long multi-epitope candidate vaccine using in silico approaches [78]. The in silico multi-epitope vaccine design method has also been used to design vaccine candidates against *Rhizopus microsporus*, a mucormycosis-causing fungus [79]. Soltan et al. targeted the spore coat and serine protease of *R. microsporus* to predict immunogenic T helper, T cytotoxic, and B cell epitopes which were joined to PADRE peptide and beta-defensin adjuvant to design a vaccine candidate using the various computational tools [79]. The in silico multi-epitope vaccine design has been employed to design vaccine candidates against cancer. Sanami et al. used this approach to design a vaccine candidate against cervical cancer by targeting the E6 and E7 oncoproteins of human papillomavirus (HPV16) [80].

The SAP2p was chosen as the target for developing an epitope-based vaccination in this study because it is necessary for *Candida* species’ virulence and pathogenesis [21–23]. Moreover, this protein has been frequently targeted to design various recombinant and hybrid phage vaccines against different *Candida* species [28–31]. The protein was predicted as antigenic and extracellular or plasma membrane protein in this study. A protein is regarded as a suitable candidate for vaccine creation if it is antigenic and extracellular or plasma membrane [43]. The similarity between the host and pathogen epitopes can increase the chances of cross-reactivity. The SAP2 protein from *C. tropicalis* has relatively little homology (less than 30%) with human proteins, implying that there is very little chance of cross-reactivity. The protein was then utilized to predict a number of B and T cell epitopes. Helper and cytotoxic T cells bind to MHC-II and MHC-I molecules, respectively, and play an important role in the development of a strong cell-mediated immune response.

After epitopes were predicted, their antigenicity, toxicity, allergenicity, conservancy, and interferon-activating potential were determined. Epitopes with a Vaxijen score of greater than 1.1 were chosen because they are thought to be antigenic [60]. If the epitopes are non-allergic and non-toxic, there can be low chances of adverse effects. Interferon activation helps to activate the innate and adaptive immune systems while also protecting against invasive Candidiasis and Aspergillosis [81–83]. Furthermore, epitope conservation between strains can aid in overcoming obstacles such as antigenic shift and antigenic drift. The selection of conserved epitopes can provide better coverage and protection against the pathogenic infections [78]. As a result, for the vaccine construct design, highly antigenic, non-toxic, and non-allergic epitopes that were conserved across several strains were chosen. Altogether, 11 epitopes were predicted as antigenic, non-toxic, and non-allergenic. These epitopes along with adjuvants were joined together by the GGS linker. Antigen processing is assisted by linkers, which also aid in the prevention of junctional epitope formation in multi-epitope vaccines. Candidate vaccines against human papillomavirus, human cytomegalovirus, *C. auris*, and dengue virus have already been developed using these adjuvants and linkers [32, 42, 71, 76]. RS09 and flagellin protein are TLR4 and TLR5 agonists and help in the generation of the innate and adaptive immune response [84–86]. PADRE (Pan DR epitope) sequence which activates CD4 + T cells, provides stability to the vaccine, and helps in overcoming the problems due to polymorphism of HLA-DR molecules was also added to the vaccine construct [48, 87]. Finally, a vaccine construct with 441 amino acids was developed. Antigenic, soluble, stable, and non-allergic qualities were predicted for the designed vaccine construct. The majority of the residues (95.4%) were in favored or authorized regions, indicating that the vaccine construct’s tertiary structure was satisfactory and reliable and can be used for further studies like molecular docking and molecular dynamics analysis. C-score, which ranges from − 5 to 2, was also utilized to validate the vaccine construct’s tertiary structure. A higher C-score indicates that the protein’s 3D model is more important [53]. The vaccine construct’s C-score was 0.19, indicating that the model was of good quality. Additional research, such as molecular docking and molecular dynamics analysis, were conducted using the vaccine construct’s 3D model. The vaccine design was docked with TLR and HLA DRB 0101 molecules once the 3D model was validated. The vaccine design interacted with these molecules with a negative binding energy, implying better binding affinity and interaction. A molecular dynamic simulation analysis was used to assess the stability of interactions between the vaccine construct and the HLA DRB 0101 and TLR5 molecules. The interactions were found to be both stable and adaptable, according to the research. Finally, the vaccine design was successfully cloned in *Escherichia coli* using in silico cloning. The vaccine design was found to be dissimilar to human proteins (taxid: 9606; *Homo sapiens*) in a protein–protein BLAST investigation, indicating that it could be safe for human use. These findings suggest that the vaccine developed in this study could be a safe and effective choice for preventing *C. tropicalis* infections. However, different in vitro and in vivo research are required to further validate these in silico experimental results.

## Conclusion

After conducting extensive in silico studies, a vaccine candidate against *C. tropicalis* was developed using multiple safe and antigenic epitopes predicted from the Sap2p. Both cell-mediated and humoral immune responses can be elicited by the multi-epitope vaccination design. It was projected that the vaccine design would be soluble, stable, antigenic, and non-allergic. The results of the molecular dynamics simulation analysis reveal that HLA DR1 (PDB: 4AH2) binds more successfully against the intended construct than TLR5A (PDB: 3JOA). These computer simulations show that the vaccine design could be a safe and effective way to treat *C. tropicalis* infections. Because the results are based on an in silico experiment, in vivo research is needed to confirm them.

## Supplementary Information

**Additional file 1: Table S1.** Prediction of helper T cell epitopes and their antigenicity, allergenicity, toxicity and interferon-γ inducing ability.

**Additional file 2: Table S2.** Prediction of cytotoxic T cell epitopes and their antigenicity, allergenicity, toxicity and interferon-γ inducing ability.

**Additional file 3: Table S3.** Prediction of B cell epitopes and their antigenicity, allergenicity, toxicity and interferon-γ inducing ability.

**Additional file 4: Table S4.** Program: BLASTP.

**Additional file 5: Table S5.** Relative surface accessibility of the amino acid residues in the epitope of the designed vaccine candidate.

## Data Availability

Data would be made available on request.

## Abbreviations

BLAST: Basic local alignment search tool
HLA: Human leukocyte antigen
HMM: Hidden Markov model
MHC: Major histocompatibility complex
PADRE: Pan DR epitope
PDB: Protein data bank
RMSD: Root mean square deviation
SAP: Secreted aspartyl proteinase
TLR: Toll-like receptor
