# Supplementary material for: Design of a multi-epitope vaccine against the pathogenic fungi Candida tropicalis using an in silico approach
Source: J Genet Eng Biotechnol. 2022 Sep 29;20:140. doi: 10.1186/s43141-022-00415-3 (PMC9521867; doi:10.1186/s43141-022-00415-3)
Supplement: Supplementary file 1 — Additional file 1: Table S1. Prediction of helper T cell epitopes and their antigenicity, allergenicity, toxicity and interferon-γ inducing ability. [file 43141_2022_415_MOESM1_ESM.docx]

Prediction of helper T cell epitopes and their antigenicity, allergenicity, toxicity and interferon-γ inducing ability

| Protein ID | Allele | Peptide | Binding Affinity (nM) | Vaxijen score | Antigen/Non-antigen | Allergenicity | Toxicity | Interferon-γ inducing ability |
| --- | --- | --- | --- | --- | --- | --- | --- | --- |
| AAD33216.1 | DRB1_0101 | LTYLPQSVV | 351.9 | -2.2982 | Non-antigen | Allergen | Non-toxin | No |
|  |  | ISLAPVVYT | 919.2 | -0.1584 | Non-antigen | Allergen | Non-toxin | No |
|  |  | SLYLNAPSA | 991.9 | 1.7697 | Antigen | Non-allergen | Non-toxin | No |
|  |  | FNILGDNFL | 1179.0 | 1.3801 | Antigen | Allergen | Non-toxin |  |
|  |  | YNRPIGAYI | 1254.5 | 3.0712 | Antigen | Non-allergen | Non-toxin | Yes |
|  |  | LDALTISLA | 1431.0 | 1.1536 | Antigen | Allergen | Non-toxin | No |
|  |  | DNFLRHAYV | 1487.0 | -0.4545 | Non-antigen | Allergen | Non-toxin | Yes |
|  | DRB1_0301 | ILGDNFLRH | 3549.0 | 0.2811 | Non-antigen | Allergen | Non-toxin | No |
|  |  | LLLDSGTTL | 4925.5 | -1.0216 | Non-antigen | Non-allergen | Non-toxin | Yes |
|  |  | FVVNEYSKR | 6255.0 | 1.6028 | Antigen | Allergen | Non-toxin | No |
|  |  | NILGDNFLR | 7598.4 | 0.7548 | Antigen | Allergen | Non-toxin | No |
|  | DRB1_0401 | FTIQTNSAT | 1119.9 | 2.1036 | Antigen | Non-allergen | Non-toxin | No |
|  |  | YSLYLNAPS | 2333.2 | 2.5855 | Antigen | Allergen | Non-toxin | No |
|  |  | LYNEQVTYS | 4011.2 | -0.8383 | Non-antigen | Non-allergen | Non-toxin | No |
|  |  | YTSDSNVTI | 5569.3 | 2.7233 | Antigen | Allergen | Non-toxin | No |
|  |  | LYLNAPSAT | 6137.7 | 1.5982 | Antigen | Allergen | Non-toxin | Yes |
|  |  | VYTSDSNVT | 6262.8 | 2.4300 | Antigen | Allergen | Non-toxin | No |
|  |  | LLLDSGTTL | 6729.8 | -1.0216 | Non-antigen | Non-allergen | Non-toxin | Yes |
|  |  | YNEQVTYSA | 6962.7 | -0.1080 | Non-antigen | Non-allergen | Non-toxin | No |
|  |  | EFTIQTNSA | 7174.0 | 2.1227 | Antigen | Non-allergen | Non-toxin | No |
|  |  | WVKDTVGIN | 7398.5 | 1.2578 | Antigen | Allergen | Non-toxin | No |
|  |  | YLNAPSATS | 7629.1 | 1.1722 | Antigen | Allergen | Non-toxin | No |
|  | DRB1_0701 | FINTNAYSL | 122.7 | 0.9970 | Antigen | Allergen | Non-toxin | No |
|  |  | LTISLAPVV | 127.8 | 0.7500 | Antigen | Allergen | Non-toxin | No |
|  |  | ILYGENFNI | 163.0 | 4.2936 | Antigen | Non-allergen | Non-toxin | No |
|  |  | FTIGPHFVV | 185.2 | 2.8195 | Antigen | Allergen | Non-toxin | No |
|  |  | VTYSANFTV | 188.6 | 2.8501 | Antigen | Allergen | Non-toxin | No |
|  |  | VAGFCALGI | 305.3 | -0.1475 | Non-antigen | Non-allergen | Non-toxin | Yes |
|  |  | YNRPIGAYI | 330.0 | 3.0712 | Antigen | Non-allergen | Non-toxin | Yes |
|  | DRB1_0801 | VWVIGLMKQ | 673.6 | -1.1985 | Non-antigen | Allergen | Non-toxin | No |
|  | DRB1_0901 | LTISLAPVV | 150.7 | 0.7500 | Antigen | Allergen | Non-toxin | No |
|  |  | WVIGLMKQV | 338.9 | -0.1818 | Non-antigen | Allergen | Non-toxin | Yes |
|  |  | VAGFCALGI | 376.3 | -0.1475 | Non-antigen | Non-allergen | Non-toxin | Yes |
|  |  | LYYSNGAVA | 472.0 | -1.1274 | Non-antigen | Allergen | Non-toxin | No |
|  |  | FINTNAYSL | 624.9 | 0.9970 | Antigen | Allergen | Non-toxin | No |
|  |  | YNRPIGAYI | 666.1 | 3.0712 | Antigen | Non-allergen | Non-toxin | Yes |
|  |  | VFGLTVEGL | 806.4 | 0.7920 | Antigen | Non-allergen | Non-toxin | Yes |
|  | DRB1_1001 | YSLYLNAPS | 236.4 | 2.5855 | Antigen | Allergen | Non-toxin | No |
|  |  | LDALTISLA | 1543.3 | 1.1536 | Antigen | Allergen | Non-toxin | No |
|  | DRB1_1101 | YIWSCNRNG | 3478.1 | 1.4717 | Antigen | Allergen | Non-toxin | No |
|  | DRB1_1201 | TISLAPVVY | 695.0 | 0.2720 | Non-antigen | Non-allergen | Non-toxin | No |
|  |  | VIGLMKQVF | 834.7 | -0.0157 | Non-antigen | Allergen | Non-toxin | Yes |
|  |  | VYNLDALTI | 944.0 | 1.1551 | Antigen | Allergen | Non-toxin | No |
|  | DRB1_1301 | FVVNEYSKR | 67.9 | 1.6028 | Antigen | Allergen | Non-toxin | No |
|  | DRB1_1501 | YSLYLNAPS | 2756.1 | 2.5855 | Antigen | Allergen | Non-toxin | No |
|  |  | ILYGENFNI | 3121.9 | 4.2936 | Antigen | Non-allergen | Non-toxin | No |
|  |  | IGLMKQVFI | 4230.4 | 0.2378 | Non-antigen | Allergen | Non-toxin | No |
|  | DRB1_1602 | YSLYLNAPS | 2670.9 | 2.5855 | Antigen | Allergen | Non-toxin | No |
|  |  | YVVYNLDAL | 3523.1 | 0.2211 | Non-antigen | Non-allergen | Non-toxin | No |
|  |  | FINTNAYSL | 4094.7 | 0.9970 | Antigen | Allergen | Non-toxin | No |
|  |  | KQVFISIVF | 4975.2 | -1.6503 | Non-antigen | Non-allergen | Non-toxin | Yes |
|  |  | YLNAPSATS | 5286.1 | 1.1722 | Antigen | Allergen | Non-toxin | No |
|  |  | SLYLNAPSA | 5352.3 | 1.7697 | Antigen | Non-allergen | Non-toxin | No |
