# Supplementary material for: Design of a multi-epitope vaccine against the pathogenic fungi Candida tropicalis using an in silico approach
Source: J Genet Eng Biotechnol. 2022 Sep 29;20:140. doi: 10.1186/s43141-022-00415-3 (PMC9521867; doi:10.1186/s43141-022-00415-3)
Supplement: Supplementary file 2 — Additional file 2: Table S2. Prediction of cytotoxic T cell epitopes and their antigenicity, allergenicity, toxicity and interferon-γ inducing ability. [file 43141_2022_415_MOESM2_ESM.docx]

**Prediction of cytotoxic T cell epitopes and their antigenicity, allergenicity, toxicity and interferon-γ inducing ability**

| Protein ID | Allele | Peptide | Binding Affinity (nM) | Vaxijen score | Antigen/Non-antigen | Allergenicity | Toxicity | Interferon-γ inducing ability |
| --- | --- | --- | --- | --- | --- | --- | --- | --- |
| AAD33216.1 | HLA-A0101 | YSDLAVPLY | 2.74 | 0.4730 | Non-antigen | Allergen | Non-toxin | No |
|  |  | STDTIYENF | 682.94 | -0.0368 | Non-antigen | Allergen | Non-toxin | No |
|  |  | TSDSNVTIV | 708.31 | 2.6946 | Antigen | Allergen | Non-toxin | No |
|  | HLA-A0201 | ILYGENFNI | 9.07 | 4.2936 | Antigen | Non-allergen | Non-toxin | No |
|  |  | ALTISLAPV | 26.15 | 0.8586 | Antigen | Allergen | Non-toxin | No |
|  |  | LLLDSGTTL | 26.87 | -1.0216 | Non-antigen | Non-allergen | Non-toxin | Yes |
|  |  | GVLSLELYV | 27.99 | 0.8796 | Antigen | Non-allergen | Non-toxin | No |
|  |  | VTYSANFTV | 30.52 | 2.8501 | Antigen | Allergen | Non-toxin | No |
|  |  | NLDALTISL | 35.22 | 1.9350 | Antigen | Allergen | Non-toxin | No |
|  | HLA-A0301 | MVWVIGLMK | 33.08 | -1.5958 | Non-antigen | Allergen | Non-toxin | No |
|  |  | GLMGNFFDK | 50.73 | 1.7030 | Antigen | Non-allergen | Non-toxin | Yes |
|  | HLA-A2402 | VYNLDALTI | 72.30 | 1.1551 | Antigen | Allergen | Non-toxin | No |
|  |  | LYGENFNIL | 90.74 | 4.5267 | Antigen | Allergen | Non-toxin | No |
|  |  | KYTGSLTTL | 182.46 | 2.3614 | Antigen | Non-allergen | Non-toxin | No |
|  |  | RNGKVTYNF | 318.21 | -0.3394 | Non-antigen | Allergen | Non-toxin | No |
|  | HLA-A2601 | DTVGINGAI | 149.21 | 1.9910 | Antigen | Non-allergen | Non-toxin | No |
|  |  | YSNGAVAGF | 173.29 | 1.0093 | Antigen | Allergen | Non-toxin | No |
|  |  | STIDINTGL | 330.13 | 0.6067 | Antigen | Allergen | Non-toxin | No |
|  |  | ELYNEQVTY | 497.16 | -0.8811 | Non-antigen | Allergen | Non-toxin | No |
|  |  | YVVYNLDAL | 821.16 | 0.2211 | Non-antigen | Non-allergen | Non-toxin | No |
|  | HLA-B0702 | APSATSGTI | 18.08 | 0.7171 | Antigen | Allergen | Non-toxin | No |
|  |  | LPLTSNREF | 93.41 | 2.7926 | Antigen | Non-allergen | Non-toxin | No |
|  | HLA-B0801 | LMKQVFISI | 67.96 | -1.0823 | Non-antigen | Non-allergen | Non-toxin | Yes |
|  |  | FINTNAYSL | 100.03 | 0.9970 | Antigen | Allergen | Non-toxin | No |
|  |  | NFLRHAYVV | 655.53 | 0.6469 | Antigen | Allergen | Non-toxin | Yes |
|  | HLA-B3901 | NHDDSNFTI | 12.64 | 1.0878 | Antigen | Non-allergen | Non-toxin | No |
|  |  | FINTNAYSL | 39.06 | 0.9970 | Antigen | Allergen | Non-toxin | No |
|  |  | NLDALTISL | 90.82 | 1.9350 | Antigen | Allergen | Non-toxin | No |
|  |  | RHAYVVYNL | 92.35 | 0.0285 | Non-antigen | Allergen | Non-toxin | Yes |
|  |  | DHAKYTGSL | 166.67 | 0.7087 | Antigen | Allergen | Non-toxin | Yes |
|  |  | YTSDSNVTI | 357.89 | 2.7233 | Antigen | Allergen | Non-toxin | No |
|  | HLA-B4001 | VEGLAISKL | 352.88 | 0.6094 | Antigen | Allergen | Non-toxin | No |
|  |  | QELGKSFNI | 541.55 | 4.9939 | Antigen | Non-allergen | Non-toxin | Yes |
|  |  | KQVFISIVF | 1191.89 | -1.6503 | Non-antigen | Non-allergen | Non-toxin | Yes |
|  | HLA-B5801 | RSTPGVLSL | 39.75 | 0.6961 | Antigen | Non-allergen | Non-toxin | No |
|  |  | YSNGAVAGF | 45.40 | 1.0093 | Antigen | Allergen | Non-toxin | No |
|  |  | YSDLAVPLY | 206.52 | 0.4730 | Non-antigen | Allergen | Non-toxin | No |
|  |  | LTSNREFTI | 297.90 | 3.6406 | Antigen | Non-allergen | Non-toxin | No |
|  | HLA-B1501 | KQVFISIVF | 12.83 | -1.6503 | Non-antigen | Non-allergen | Non-toxin | Yes |
|  |  | FLRHAYVVY | 29.47 | 0.2176 | Non-antigen | Allergen | Non-toxin | Yes |
|  |  | LMKQVFISI | 43.27 | -1.0823 | Non-antigen | Non-allergen | Non-toxin | Yes |
|  |  | YSNGAVAGF | 59.76 | 1.0093 | Antigen | Allergen | Non-toxin | No |
|  |  | EQVTYSANF | 69.25 | 1.3413 | Antigen | Allergen | Non-toxin | No |
|  |  | TIQELGKSF | 206.67 | 0.3907 | Non-antigen | Non-allergen | Non-toxin | Yes |
|  |  | TISLAPVVY | 265.57 | 0.2720 | Non-antigen | Non-allergen | Non-toxin | No |
