# Supplementary material for: Design of a multi-epitope vaccine against the pathogenic fungi Candida tropicalis using an in silico approach
Source: J Genet Eng Biotechnol. 2022 Sep 29;20:140. doi: 10.1186/s43141-022-00415-3 (PMC9521867; doi:10.1186/s43141-022-00415-3)
Supplement: Supplementary file 3 — Additional file 3: Table S3. Prediction of B cell epitopes and their antigenicity, allergenicity, toxicity and interferon-γ inducing ability. [file 43141_2022_415_MOESM3_ESM.docx]

Prediction of B cell epitopes and their antigenicity, allergenicity, toxicity and interferon-γ inducing ability

| Protein ID | Peptide | Vaxijen | Antigen/Non-antigen | Allergen | Toxin | Interferon |
| --- | --- | --- | --- | --- | --- | --- |
| AAD33216.1 | KLNDRGAAPGLMGNFFDKRSTPGVLS | 0.4875 | Non-antigen | Non-allergen | Non-toxin | Yes |
|  | LYVNRNHDDSNFTIGPHFVVNEYSKRDDYI | 1.0304 | Antigen | Non-allergen | Non-toxin | Yes |
|  | ANCQEKSGYSSDYCFSGGTYDPSSSSTIQE | 0.4355 | Non-antigen | Allergen | Non-toxin | Yes |
|  | TSVSQGILGIGLDTNESTDTIYEN | 1.0629 | Antigen | Allergen | Non-toxin | No |
|  | AKYTGSLTTL | 2.0617 | Antigen | Non-allergen | Non-toxin | No |
|  | IGGDITYNRPIGAYIWSCNRNGK | 2.1725 | Antigen | Non-allergen | Non-toxin | No |
