# Supplementary material for: Design of a multi-epitope vaccine against the pathogenic fungi Candida tropicalis using an in silico approach
Source: J Genet Eng Biotechnol. 2022 Sep 29;20:140. doi: 10.1186/s43141-022-00415-3 (PMC9521867; doi:10.1186/s43141-022-00415-3)
Supplement: Supplementary file 4 — Additional file 4: Table S4. Program: BLASTP. [file 43141_2022_415_MOESM4_ESM.doc]

RID: 72Z4KKNY016

Job Title:lcl|AF115320.1_prot_AAD33216.1_1 [gene=SAPT2]

Program: BLASTP

Database: nr All non-redundant GenBank CDS translations+PDB+SwissProt+PIR+PRF excluding environmental samples from WGS projects

Query #1: lcl|AF115320.1_prot_AAD33216.1_1 [gene=SAPT2] [protein=secreted aspartic protease 2] [protein_id=AAD33216.1] [location=276..1544] [gbkey=CDS] Query ID: lcl|Query_25703 Length: 422

Sequences producing significant alignments:

Scientific Common Max Total Query E Per. Acc.

Description Name Name Taxid Score Score cover Value Ident Len Accession

Crystal Structure of an activation intermediate of Cathepsin E... Homo sapiens human 9606 108 108 75% 4e-25 30.92 351 1TZS_A

cathepsin E isoform a preproprotein [Homo sapiens] Homo sapiens human 9606 108 108 84% 6e-25 30.05 396 NP_001901.1

cathepsin E isoform X1 [Homo sapiens] Homo sapiens human 9606 102 102 84% 7e-23 29.67 401 XP_011507546.1

pepsin A-5 preproprotein [Homo sapiens] Homo sapiens human 9606 100 100 75% 4e-22 30.65 388 NP_055039.1

Pepsinogen 5, group I (pepsinogen A) [Homo sapiens] Homo sapiens human 9606 100 100 75% 5e-22 30.65 388 AAI71897.1

pepsinogen [Homo sapiens] Homo sapiens human 9606 99.0 99.0 72% 1e-21 27.16 385 AAA60062.1

gastricsin isoform 1 preproprotein [Homo sapiens] Homo sapiens human 9606 99.0 99.0 72% 1e-21 27.16 388 NP_002621.1

Activation Intermediate 2 Of Human Gastricsin From Human Stoma... Homo sapiens human 9606 97.8 97.8 72% 1e-21 27.16 329 1AVF_A

unnamed protein product [Homo sapiens] Homo sapiens human 9606 98.2 98.2 75% 2e-21 30.36 388 BAF84553.1

Crystal Structure Of Human Uropepsin At 2.45 A Resolution [Hom... Homo sapiens human 9606 97.4 97.4 75% 2e-21 30.36 326 1FLH_A

pepsin A-4 preproprotein [Homo sapiens] Homo sapiens human 9606 98.2 98.2 75% 2e-21 30.36 388 NP_001073276.1

pepsin A-3 preproprotein [Homo sapiens] Homo sapiens human 9606 98.2 98.2 75% 3e-21 30.36 388 NP_001073275.1

The Crystal Structure Of Human Pepsin And Its Complex With... Homo sapiens human 9606 97.1 97.1 75% 3e-21 30.36 326 1PSN_A

Pepsinogen 4, group I (pepsinogen A) [Homo sapiens] Homo sapiens human 9606 97.8 97.8 75% 3e-21 30.36 388 AAI71808.1

Pepsinogen 4, group I (pepsinogen A) [Homo sapiens] Homo sapiens human 9606 97.8 97.8 75% 3e-21 30.36 388 AAI71814.1

Pepsinogen 3, group I (pepsinogen A) [Homo sapiens] Homo sapiens human 9606 97.4 97.4 75% 4e-21 30.36 388 AAI71815.1

pepsinogen A [Homo sapiens] Homo sapiens human 9606 97.4 97.4 75% 4e-21 30.36 388 AAA60061.1

napsin A aspartic peptidase, isoform CRA_c [Homo sapiens] Homo sapiens human 9606 89.4 89.4 57% 1e-18 31.37 328 EAW71849.1

napsin-A preproprotein [Homo sapiens] Homo sapiens human 9606 90.1 90.1 62% 2e-18 30.80 420 NP_004842.1

Napsin A aspartic peptidase [Homo sapiens] Homo sapiens human 9606 89.7 89.7 62% 2e-18 30.80 420 AAH17842.1

napsin A aspartic peptidase, isoform CRA_b [Homo sapiens] Homo sapiens human 9606 87.8 87.8 61% 6e-18 30.74 357 EAW71848.1

cathepsin E isoform c [Homo sapiens] Homo sapiens human 9606 84.7 84.7 43% 3e-17 32.99 288 NP_001304260.1

cathepsin E isoform b precursor [Homo sapiens] Homo sapiens human 9606 85.5 85.5 53% 4e-17 30.51 363 NP_683865.1

cathepsin E isoform X2 [Homo sapiens] Homo sapiens human 9606 79.0 79.0 53% 6e-15 29.88 368 XP_011507547.1

cathepsin D preproprotein [Homo sapiens] Homo sapiens human 9606 74.7 74.7 72% 2e-13 26.95 412 NP_001900.1

Ketopiperazine-Based Renin Inhibitors: Optimization of the C... Homo sapiens human 9606 73.2 73.2 57% 4e-13 27.13 333 2FS4_A

Human renin in complex with remikiren [Homo sapiens] Homo sapiens human 9606 73.2 73.2 57% 4e-13 27.13 341 3D91_A

Human renin/PF02342674 complex [Homo sapiens] Homo sapiens human 9606 73.2 73.2 57% 4e-13 27.13 336 2I4Q_A

CRYSTALLOGRAPHIC STUDIES ON THE BINDING MODES OF P2-P3... Homo sapiens human 9606 73.2 73.2 57% 5e-13 27.13 337 1BIL_A

Structure-based design of a new series of... Homo sapiens human 9606 73.2 73.2 57% 5e-13 27.13 339 5SXN_A

X-RAY ANALYSES OF PEPTIDE INHIBITOR COMPLEXES DEFINE THE... Homo sapiens human 9606 73.2 73.2 57% 5e-13 27.13 340 1BBS_A

Crystal structure of human prorenin [Homo sapiens] Homo sapiens human 9606 72.8 72.8 57% 5e-13 27.17 335 3VCM_A

renin [Homo sapiens] Homo sapiens human 9606 73.2 73.2 57% 6e-13 27.45 403 AAA60364.1

Crystal structure of human angiotensinogen complexed with reni... Homo sapiens human 9606 72.8 72.8 57% 7e-13 27.13 383 2X0B_A

Crystal structure of the complex of human angiotensinogen and... Homo sapiens human 9606 72.4 72.4 57% 7e-13 27.13 340 6I3F_B

renin preproprotein [Homo sapiens] Homo sapiens human 9606 72.8 72.8 57% 1e-12 27.13 406 NP_000528.1

renin [Homo sapiens] Homo sapiens human 9606 72.4 72.4 57% 1e-12 27.06 403 AAR03502.1

unnamed protein product [Homo sapiens] Homo sapiens human 9606 60.8 60.8 54% 3e-09 28.57 282 BAG62845.1

gastricsin isoform 2 preproprotein [Homo sapiens] Homo sapiens human 9606 57.4 57.4 34% 6e-08 29.30 315 NP_001159896.1

Crystal Structure of human Beta Secretase complexed with IXS... Homo sapiens human 9606 57.8 57.8 59% 7e-08 26.64 411 2QZL_A

Crystal structure of human Beta secretase complexed with... Homo sapiens human 9606 57.4 57.4 59% 8e-08 26.64 405 1TQF_A

Crystal structure of BACE1 bound to inhibitor [Homo sapiens] Homo sapiens human 9606 57.0 57.0 59% 1e-07 26.64 413 3EXO_A

Design and synthesis of hydroxyethylamine (hea) BACE-1... Homo sapiens human 9606 57.0 57.0 59% 1e-07 26.64 408 3QI1_A

Structure of BACE1 complex with a HEA-type inhibitor [Homo... Homo sapiens human 9606 57.0 57.0 59% 1e-07 26.64 388 4TRY_A

beta-secretase 1 isoform A preproprotein [Homo sapiens] Homo sapiens human 9606 57.0 113 59% 1e-07 26.64 501 NP_036236.1

BACE1 compound 28 [Homo sapiens] Homo sapiens human 9606 57.0 57.0 59% 1e-07 26.64 501 6EJ2_A

Aminoimidazoles as BACE-1 Inhibitors: From De Novo Design to A... Homo sapiens human 9606 56.6 56.6 59% 1e-07 26.64 385 4B70_A

Lead Generation of BACE1 Inhibitors by Coupling Non-amidine Ne... Homo sapiens human 9606 56.6 56.6 59% 1e-07 26.64 384 4B0Q_A

New Aminoimidazoles as BACE-1 Inhibitors: From Rational Design... Homo sapiens human 9606 56.6 56.6 59% 1e-07 26.64 388 4B1D_A

Crystal structure of the human BACE1 catalytic domain in compl... Homo sapiens human 9606 56.6 56.6 59% 1e-07 26.64 405 2ZJI_A

APO structure of BACE1 [Homo sapiens] Homo sapiens human 9606 57.0 57.0 59% 1e-07 26.64 433 3TPJ_A

Crystal structure of beta-secretase complexed with an... Homo sapiens human 9606 56.6 56.6 59% 1e-07 26.64 388 2FDP_A

beta-secretase 1 precursor variant 1 [Homo sapiens] Homo sapiens human 9606 57.0 57.0 59% 1e-07 26.64 501 ACA05927.1

Design and synthesis of potent hydroxyethylamine (hea) bace-1... Homo sapiens human 9606 56.6 56.6 59% 1e-07 26.64 386 4EWO_A

Structure of BACE bound to SCH346572 [Homo sapiens] Homo sapiens human 9606 56.6 56.6 59% 1e-07 26.64 395 3KMX_A

Structure of BACE1 bound to SCH626485 [Homo sapiens] Homo sapiens human 9606 56.6 56.6 59% 1e-07 26.64 395 2QK5_A

Structure of BACE Bound to SCH727596 [Homo sapiens] Homo sapiens human 9606 56.6 56.6 59% 1e-07 26.64 390 3CIB_A

BACE-1 in complex with a HEA-macrocyclic type inhibitor [Homo... Homo sapiens human 9606 56.6 56.6 59% 1e-07 26.64 391 4DPF_A

Beta-secretase 1 complexed with statine-based inhibitor [Homo... Homo sapiens human 9606 56.6 56.6 59% 1e-07 26.64 406 3DM6_A

memapsin 2 [Homo sapiens] Homo sapiens human 9606 56.6 56.6 59% 1e-07 26.64 488 AAF13715.1

Crystal Structure of Unbound Beta-Secretase Catalytic Domain... Homo sapiens human 9606 56.6 56.6 59% 1e-07 26.64 389 1SGZ_A

Crystal Structure of Human BACE-1 in Complex with... Homo sapiens human 9606 56.6 56.6 59% 1e-07 26.64 396 6FGY_A

Structure of Beta-Secretase Complexed with Inhibitor [Homo... Homo sapiens human 9606 56.6 56.6 59% 1e-07 26.64 391 1FKN_A

Crystal structure of the human BACE1 catalytic domain in compl... Homo sapiens human 9606 56.6 56.6 59% 2e-07 26.64 405 2ZJH_A

Crystal structure of the human BACE1 catalytic domain in compl... Homo sapiens human 9606 56.6 56.6 59% 2e-07 26.64 405 2ZJN_A

Potent beta-secretase 1 inhibitor [Homo sapiens] Homo sapiens human 9606 56.6 56.6 59% 2e-07 26.64 405 3IXK_A

Crystal structure of human beta secretase complexed with... Homo sapiens human 9606 56.6 56.6 59% 2e-07 26.64 402 1YM2_A

Rational Design and Synthesis of Aminopiperazinones as Beta... Homo sapiens human 9606 56.6 56.6 59% 2e-07 26.64 390 3U6A_A

human Bace (beta secretase) in complex with... Homo sapiens human 9606 56.6 113 59% 2e-07 26.64 412 2WJO_A

co-crystal structure of BACE with inhibitor AM-6494 [Homo... Homo sapiens human 9606 56.6 56.6 59% 2e-07 26.64 411 6PZ4_A

Crystal Structure of BACE with Compound 1 [Homo sapiens] Homo sapiens human 9606 56.2 56.2 59% 2e-07 26.64 404 3UDH_A

Crystal structure of the human BACE1 catalytic domain in compl... Homo sapiens human 9606 56.2 56.2 59% 2e-07 27.37 405 2ZJK_A

CRYSTAL STRUCTURE OF BACE-1 IN COMPLEX WITH... Homo sapiens human 9606 56.2 56.2 59% 2e-07 26.64 390 5EZX_A

Apo Structure of BACE (Beta Secretase) [Homo sapiens] Homo sapiens human 9606 56.2 56.2 59% 2e-07 26.64 411 1W50_A

Crystal Structure of human beta-secretase (BACE) in the presen... Homo sapiens human 9606 56.2 56.2 59% 2e-07 26.64 406 2HM1_A

Crystal structure of BACE1 in complex with OM99-2 at pH 5.0... Homo sapiens human 9606 56.2 56.2 59% 2e-07 26.64 411 2ZHR_A

X-ray crystal structure of beta secretase complexed with... Homo sapiens human 9606 56.2 56.2 59% 2e-07 26.64 402 2OF0_A

Structure of bace (beta secretase) in Complex with EV0 [Homo... Homo sapiens human 9606 56.2 56.2 59% 2e-07 26.64 411 3HVG_A

BACE1 in complex with... Homo sapiens human 9606 56.2 56.2 59% 2e-07 26.64 412 5HTZ_A

Structure of BACE Bound to SCH589432 [Homo sapiens] Homo sapiens human 9606 56.2 56.2 59% 2e-07 26.64 414 3L58_A

Crystal structure of BACE-1 in complex with inhibitor [Homo... Homo sapiens human 9606 56.2 56.2 59% 2e-07 26.64 412 3CKP_A

Crystal Structure of beta secetase in complex with... Homo sapiens human 9606 56.2 56.2 59% 2e-07 26.64 416 3VV6_A

BACE-1 IN COMPLEX WITH LIGAND 32397778 [Homo sapiens] Homo sapiens human 9606 56.2 56.2 59% 2e-07 26.64 432 5MXD_A

Crystal structure of BACE1 complexed with an inhibitor [Homo... Homo sapiens human 9606 56.2 56.2 59% 2e-07 26.64 433 3TPR_A

BACE-1 complexed with compound 1 [Homo sapiens] Homo sapiens human 9606 56.2 56.2 59% 2e-07 26.64 409 3BRA_A

CRYSTAL STRUCTURE OF BACE-1 IN COMPLEX WITH Pep#3 [Homo sapiens] Homo sapiens human 9606 56.2 56.2 59% 2e-07 26.64 409 5MBW_A

1,4-Oxazine BACE1 inhibitors [Homo sapiens] Homo sapiens human 9606 56.2 56.2 59% 2e-07 26.64 401 5CLM_A

Structure Basis of Allosteric Inhibition of BACE1 by an... Homo sapiens human 9606 56.2 56.2 59% 2e-07 26.64 402 3R1G_B

BACE1 with Compound 1 [Homo sapiens] Homo sapiens human 9606 56.2 56.2 59% 2e-07 26.64 415 2QU2_A

Crystal structure of human beta secretase complexed with... Homo sapiens human 9606 56.2 56.2 59% 2e-07 26.64 408 1YM4_A

Diethylaminosulfur Trifluoride-Mediated Intramolecular... Homo sapiens human 9606 56.2 56.2 59% 2e-07 26.64 409 4L7G_A

Crystal Structure of BACE with amino thiazine inhibitor... Homo sapiens human 9606 56.2 56.2 59% 2e-07 26.64 442 4X7I_A

BACE1 in complex with inhibitor 5g [Homo sapiens] Homo sapiens human 9606 56.2 56.2 59% 2e-07 26.64 441 5V0N_A

Crystal Structure of human beta-secretase (BACE) in the presen... Homo sapiens human 9606 55.8 55.8 59% 2e-07 26.64 455 2HIZ_A

X-ray crystal structure of beta secretase complexed with... Homo sapiens human 9606 55.8 55.8 59% 2e-07 26.64 455 2VA5_A

Structure of BACE complexed to compound 3a [Homo sapiens] Homo sapiens human 9606 55.8 55.8 59% 3e-07 27.27 385 2Q15_A

Structure of BACE complexed to compound 1 [Homo sapiens] Homo sapiens human 9606 55.8 55.8 59% 3e-07 27.27 388 2Q11_A

Crystal structure of the human BACE1 catalytic domain in compl... Homo sapiens human 9606 55.8 55.8 59% 3e-07 26.30 405 2ZJJ_A

Alignments:

>Crystal Structure of an activation intermediate of Cathepsin E [Homo sapiens]

Sequence ID: 1TZS_A Length: 351

Range 1: 23 to 342

Score:108 bits(269), Expect:4e-25,

Method:Compositional matrix adjust.,

Identities:107/346(31%), Positives:169/346(48%), Gaps:53/346(15%)

Query 94 VTYSANFTVGSNSQKQNVIVDTGSSDLWVVDSSANCQEKSGYSSDYCFSGGTYDPSSSST 153

+ Y ++GS Q VI DTGSS+LWV S C +S C + + PS SST

Sbjct 23 MEYFGTISIGSPPQNFTVIFDTGSSNLWV--PSVYC------TSPACKTHSRFQPSQSST 74

Query 154 IQELGKSFNIRYGDGSSSSGTWVKDTVGINGAIILNQQFGDVNSTSVSQ--------GIL 205

+ G+SF+I+YG GS S G D V + G ++ QQFG+ + T Q GIL

Sbjct 75 YSQPGQSFSIQYGTGSLS-GIIGADQVSVEGLTVVGQQFGE-SVTEPGQTFVDAEFDGIL 132

Query 206 GIGLDTNE--STDTIYENFPINLKEQGFINTNAYSLYLNA-PSATSGT-IIFGGIDHAKY 261

G+G + +++N + Q ++ +S+Y+++ P +G+ +IFGG DH+ +

Sbjct 133 GLGYPSLAVGGVTPVFDN----MMAQNLVDLPMFSVYMSSNPEGGAGSELIFGGYDHSHF 188

Query 262 TGSLTTLPLTSNREFTIQTNSATVGTSTIDINTGL--LLDSGTTLTYLPQSVVDSIANAI 319

+GSL +P+T + I ++ VG + + + G ++D+GT+L P + + NAI

Sbjct 189 SGSLNWVPVTKQAYWQIALDNIQVGGTVMFCSEGCQAIVDTGTSLITGPSDKIKQLQNAI 248

Query 320 GGDITYNRPI-GAYIWSC---NRNGKVTYNFPQGLNIDIPYSDLAVPLYYSNGAVAG--F 373

G P+ G Y C N VT+ +N +PY+ L+ Y V G F

Sbjct 249 GA-----APVDGEYAVECANLNVMPDVTFT----IN-GVPYT-LSPTAYTLLDFVDGMQF 297

Query 374 CALGILYGENFN-------ILGDNFLRHAYVVYNLDALTISLAPVV 412

C+ G G + + ILGD F+R Y V++ + LAP V

Sbjct 298 CSSG-FQGLDIHPPAGPLWILGDVFIRQFYSVFDRGNNRVGLAPAV 342

>cathepsin E isoform a preproprotein [Homo sapiens]

Sequence ID: NP_001901.1 Length: 396

>RecName: Full=Cathepsin E; Contains: RecName: Full=Cathepsin E form I; Contains: RecName: Full=Cathepsin E form II; Flags: Precursor [Homo sapiens]

Sequence ID: P14091.3 Length: 396 >cathepsin E precursor [Homo sapiens]

Sequence ID: AAA52130.1 Length: 396 >cathepsin E [Homo sapiens]

Sequence ID: AAA52300.1 Length: 396 >Cathepsin E [Homo sapiens]

Sequence ID: AAH42537.1 Length: 396

Range 1: 44 to 395

Score:108 bits(270), Expect:6e-25,

Method:Compositional matrix adjust.,

Identities:116/386(30%), Positives:180/386(46%), Gaps:62/386(16%)

Query 55 ELYVNRNHDDSNFTIGPHFVVNEYSKRDDYISVELYNE-QVTYSANFTVGSNSQKQNVIV 113

E + + N D FT E D L N + Y ++GS Q VI

Sbjct 44 EFWKSHNLDMIQFT--------ESCSMDQSAKEPLINYLDMEYFGTISIGSPPQNFTVIF 95

Query 114 DTGSSDLWVVDSSANCQEKSGYSSDYCFSGGTYDPSSSSTIQELGKSFNIRYGDGSSSSG 173

DTGSS+LWV S C +S C + + PS SST + G+SF+I+YG GS S G

Sbjct 96 DTGSSNLWV--PSVYC------TSPACKTHSRFQPSQSSTYSQPGQSFSIQYGTGSLS-G 146

Query 174 TWVKDTVGINGAIILNQQFGDVNSTSVSQ--------GILGIGLDTNE--STDTIYENFP 223

D V + G ++ QQFG+ + T Q GILG+G + +++N

Sbjct 147 IIGADQVSVEGLTVVGQQFGE-SVTEPGQTFVDAEFDGILGLGYPSLAVGGVTPVFDN-- 203

Query 224 INLKEQGFINTNAYSLYLNA-PSATSGT-IIFGGIDHAKYTGSLTTLPLTSNREFTIQTN 281

+ Q ++ +S+Y+++ P +G+ +IFGG DH+ ++GSL +P+T + I +

Sbjct 204 --MMAQNLVDLPMFSVYMSSNPEGGAGSELIFGGYDHSHFSGSLNWVPVTKQAYWQIALD 261

Query 282 SATVGTSTIDINTGL--LLDSGTTLTYLPQSVVDSIANAIGGDITYNRPI-GAYIWSC-- 336

+ VG + + + G ++D+GT+L P + + NAIG P+ G Y C

Sbjct 262 NIQVGGTVMFCSEGCQAIVDTGTSLITGPSDKIKQLQNAIGA-----APVDGEYAVECAN 316

Query 337 -NRNGKVTYNFPQGLNIDIPYSDLAVPLYYSNGAVAG--FCALGILYGENFN-------I 386

N VT+ +N +PY+ L+ Y V G FC+ G G + + I

Sbjct 317 LNVMPDVTFT----IN-GVPYT-LSPTAYTLLDFVDGMQFCSSG-FQGLDIHPPAGPLWI 369

Query 387 LGDNFLRHAYVVYNLDALTISLAPVV 412

LGD F+R Y V++ + LAP V

Sbjct 370 LGDVFIRQFYSVFDRGNNRVGLAPAV 395

>cathepsin E isoform X1 [Homo sapiens]

Sequence ID: XP_011507546.1 Length: 401

Range 1: 44 to 400

Score:102 bits(255), Expect:7e-23,

Method:Compositional matrix adjust.,

Identities:116/391(30%), Positives:180/391(46%), Gaps:67/391(17%)

Query 55 ELYVNRNHDDSNFTIGPHFVVNEYSKRDDYISVELYNE-QVTYSANFTVGSNSQKQNVIV 113

E + + N D FT E D L N + Y ++GS Q VI

Sbjct 44 EFWKSHNLDMIQFT--------ESCSMDQSAKEPLINYLDMEYFGTISIGSPPQNFTVIF 95

Query 114 DTGSSDLWVVDSSANCQEKSGYSSDYCFSGGTYDPSSSSTIQELGKSFNIRYGDGSSSSG 173

DTGSS+LWV S C +S C + + PS SST + G+SF+I+YG GS S G

Sbjct 96 DTGSSNLWV--PSVYC------TSPACKTHSRFQPSQSSTYSQPGQSFSIQYGTGSLS-G 146

Query 174 TWVKDTVG-----INGAIILNQQFGDVNSTSVSQ--------GILGIGLDTNE--STDTI 218

D V + G ++ QQFG+ + T Q GILG+G + +

Sbjct 147 IIGADQVSAFSYQVEGLTVVGQQFGE-SVTEPGQTFVDAEFDGILGLGYPSLAVGGVTPV 205

Query 219 YENFPINLKEQGFINTNAYSLYLNA-PSATSGT-IIFGGIDHAKYTGSLTTLPLTSNREF 276

++N + Q ++ +S+Y+++ P +G+ +IFGG DH+ ++GSL +P+T +

Sbjct 206 FDN----MMAQNLVDLPMFSVYMSSNPEGGAGSELIFGGYDHSHFSGSLNWVPVTKQAYW 261

Query 277 TIQTNSATVGTSTIDINTGL--LLDSGTTLTYLPQSVVDSIANAIGGDITYNRPI-GAYI 333

I ++ VG + + + G ++D+GT+L P + + NAIG P+ G Y

Sbjct 262 QIALDNIQVGGTVMFCSEGCQAIVDTGTSLITGPSDKIKQLQNAIGA-----APVDGEYA 316

Query 334 WSC---NRNGKVTYNFPQGLNIDIPYSDLAVPLYYSNGAVAG--FCALGILYGENFN--- 385

C N VT+ +N +PY+ L+ Y V G FC+ G G + +

Sbjct 317 VECANLNVMPDVTFT----IN-GVPYT-LSPTAYTLLDFVDGMQFCSSG-FQGLDIHPPA 369

Query 386 ----ILGDNFLRHAYVVYNLDALTISLAPVV 412

ILGD F+R Y V++ + LAP V

Sbjct 370 GPLWILGDVFIRQFYSVFDRGNNRVGLAPAV 400

>pepsin A-5 preproprotein [Homo sapiens]

Sequence ID: NP_055039.1 Length: 388

>RecName: Full=Pepsin A-5; AltName: Full=Pepsinogen-5; Flags: Precursor [Homo sapiens]

Sequence ID: P0DJD9.1 Length: 388 >Pepsinogen 5, group I (pepsinogen A) [Homo sapiens]

Sequence ID: AAH29055.1 Length: 388 >Pepsinogen 5, group I (pepsinogen A) [Homo sapiens]

Sequence ID: AAI47000.1 Length: 388 >Pepsinogen 5, group I (pepsinogen A) [Homo sapiens]

Sequence ID: AAI71889.1 Length: 388 >pepsinogen 5, group I (pepsinogen A) [Homo sapiens]

Sequence ID: EAW73928.1 Length: 388

Range 1: 74 to 387

Score:100 bits(249), Expect:4e-22,

Method:Compositional matrix adjust.,

Identities:103/336(31%), Positives:160/336(47%), Gaps:40/336(11%)

Query 94 VTYSANFTVGSNSQKQNVIVDTGSSDLWVVDSSANCQEKSGYSSDYCFSGGTYDPSSSST 153

+ Y +G+ +Q V+ DTGSS+LWV S C SS C + ++P SST

Sbjct 74 MEYFGTIGIGTPAQDFTVVFDTGSSNLWV--PSVYC------SSLACTNHNRFNPEDSST 125

Query 154 IQELGKSFNIRYGDGSSSSGTWVKDTVGINGAIILNQQFGDVNSTSVS-------QGILG 206

Q ++ +I YG GS + G DTV + G NQ FG + S GILG

Sbjct 126 YQSTSETVSITYGTGSMT-GILGYDTVQVGGISDTNQIFGLSETEPGSFLYYAPFDGILG 184

Query 207 IGLDTNESTDTIYENFPI--NLKEQGFINTNAYSLYLNAPSATSGTIIFGGIDHAKYTGS 264

+ + S+ P+ N+ QG ++ + +S+YL+A + +IFGGID + YTGS

Sbjct 185 LAYPSISSSGAT----PVFDNIWNQGLVSQDLFSVYLSADDKSGSVVIFGGIDSSYYTGS 240

Query 265 LTTLPLTSNREFTIQTNSATVGTSTIDINTGL--LLDSGTTLTYLPQSVVDSIANAIGGD 322

L +P+T + I +S T+ TI G ++D+GT+L P S + +I + IG

Sbjct 241 LNWVPVTVEGYWQITVDSITMNGETIACAEGCQAIVDTGTSLLTGPTSPIANIQSDIGAS 300

Query 323 ITYNRPIGAYIWSCNRNGKV-----TYNFPQGLNIDIPYSDLAVPLYYSNGAVAGFCALG 377

+ G + SC+ + T N G+ +P S A L ++GF +

Sbjct 301 ENSD---GDMVVSCSAISSLPDIVFTIN---GVQYPVPPS--AYILQSEGSCISGFQGMN 352

Query 378 ILY--GENFNILGDNFLRHAYVVYNLDALTISLAPV 411

+ GE + ILGD F+R + V++ + LAPV

Sbjct 353 VPTESGELW-ILGDVFIRQYFTVFDRANNQVGLAPV 387

>Pepsinogen 5, group I (pepsinogen A) [Homo sapiens]

Sequence ID: AAI71897.1 Length: 388

Range 1: 74 to 387

Score:100 bits(248), Expect:5e-22,

Method:Compositional matrix adjust.,

Identities:103/336(31%), Positives:160/336(47%), Gaps:40/336(11%)

Query 94 VTYSANFTVGSNSQKQNVIVDTGSSDLWVVDSSANCQEKSGYSSDYCFSGGTYDPSSSST 153

+ Y +G+ +Q V+ DTGSS+LWV S C SS C + ++P SST

Sbjct 74 MEYFGTIGIGTPAQDFTVVFDTGSSNLWV--PSVYC------SSLACTNHNRFNPEDSST 125

Query 154 IQELGKSFNIRYGDGSSSSGTWVKDTVGINGAIILNQQFGDVNSTSVS-------QGILG 206

Q ++ +I YG GS + G DTV + G NQ FG + S GILG

Sbjct 126 YQSTSETVSITYGTGSMT-GILGYDTVQVGGISDTNQIFGLSETEPGSFLYYAPFDGILG 184

Query 207 IGLDTNESTDTIYENFPI--NLKEQGFINTNAYSLYLNAPSATSGTIIFGGIDHAKYTGS 264

+ + S+ P+ N+ QG ++ + +S+YL+A + +IFGGID + YTGS

Sbjct 185 LAYPSISSSGAT----PVFDNIWNQGLVSQDLFSVYLSADDKSGSVVIFGGIDSSYYTGS 240

Query 265 LTTLPLTSNREFTIQTNSATVGTSTIDINTGL--LLDSGTTLTYLPQSVVDSIANAIGGD 322

L +P+T + I +S T+ TI G ++D+GT+L P S + +I + IG

Sbjct 241 LNWVPVTVEGYWQITVDSITMNGETIACAEGCQAIVDTGTSLLTGPTSPIANIQSDIGAS 300

Query 323 ITYNRPIGAYIWSCNRNGKV-----TYNFPQGLNIDIPYSDLAVPLYYSNGAVAGFCALG 377

+ G + SC+ + T N G+ +P S A L ++GF +

Sbjct 301 ENSD---GDMVVSCSAISSLPDIVFTIN---GVQYPVPPS--AYILQSEGSCISGFQGMN 352

Query 378 ILY--GENFNILGDNFLRHAYVVYNLDALTISLAPV 411

+ GE + ILGD F+R + V++ + LAPV

Sbjct 353 VPTESGELW-ILGDVFIRKYFTVFDRANNQVGLAPV 387

>pepsinogen [Homo sapiens]

Sequence ID: AAA60062.1 Length: 385

Range 1: 68 to 374

Score:99.0 bits(245), Expect:1e-21,

Method:Compositional matrix adjust.,

Identities:91/335(27%), Positives:155/335(46%), Gaps:55/335(16%)

Query 94 VTYSANFTVGSNSQKQNVIVDTGSSDLWVVDSSANCQEKSGYSSDYCFSGGTYDPSSSST 153

Y ++G+ Q V+ DTGSS+LWV S CQ ++ C S ++PS SST

Sbjct 68 AAYFGEISIGTPPQNFLVLFDTGSSNLWV--PSVYCQSQA------CTSHSRFNPSESST 119

Query 154 IQELGKSFNIRYGDGSSSSGTWVKDTVGINGAIILNQQFGDVNSTSVS-------QGILG 206

G++F+++YG G S +G + DT+ + + NQ+FG + + GI+G

Sbjct 120 YSTNGQTFSLQYGSG-SLTGFFGYDTLTVQSIQVPNQEFGLSENEPGTNFVYAQFDGIMG 178

Query 207 I---GLDTNESTDTIYENFPINLKEQGFINTNAYSLYL-NAPSATSGTIIFGGIDHAKYT 262

+ L +E+T + + ++G + + +S+YL N ++ G ++FGG+D + YT

Sbjct 179 LAYPALSVDEATTAMQ-----GMVQEGALTSPVFSVYLSNQQGSSGGAVVFGGVDSSLYT 233

Query 263 GSLTTLPLTSNREFTIQTNSATVG---TSTIDINTGLLLDSGTTLTYLPQSVVDSIANAI 319

G + P+T + I +G + ++D+GT+L +PQ + ++ A

Sbjct 234 GQIYWAPVTQELYWQIGIEEFLIGGQASGWCSEGCQAIVDTGTSLLTVPQQYMSALLQAT 293

Query 320 GGDITYNRPIGAYIWSCNRNGKVTYNFPQ------GLNIDIPYSDLAVPLYYSNGAVAGF 373

G G ++ +CN N P G+ +P S + SN G+

Sbjct 294 GAQ---EDEYGQFLVNCNS----IQNLPSLTFIINGVEFPLPPSSYIL----SNN---GY 339

Query 374 CALGI-------LYGENFNILGDNFLRHAYVVYNL 401

C +G+ G+ ILGD FLR Y VY+L

Sbjct 340 CTVGVEPTYLSSQNGQPLWILGDVFLRSYYSVYDL 374

>gastricsin isoform 1 preproprotein [Homo sapiens]

Sequence ID: NP_002621.1 Length: 388

>RecName: Full=Gastricsin; AltName: Full=Pepsinogen C; Flags: Precursor [Homo sapiens]

Sequence ID: P20142.1 Length: 388 >pepsinogen C [Homo sapiens]

Sequence ID: AAA60063.1 Length: 388 >pepsinogen [Homo sapiens]

Sequence ID: AAA60074.1 Length: 388 >gastricsin [Homo sapiens]

Sequence ID: AAB18273.1 Length: 388 >Progastricsin (pepsinogen C) [Homo sapiens]

Sequence ID: AAH73740.1 Length: 388 >progastricsin (pepsinogen C) [Homo sapiens]

Sequence ID: EAX04059.1 Length: 388

Range 1: 71 to 377

Score:99.0 bits(245), Expect:1e-21,

Method:Compositional matrix adjust.,

Identities:91/335(27%), Positives:155/335(46%), Gaps:55/335(16%)

Query 94 VTYSANFTVGSNSQKQNVIVDTGSSDLWVVDSSANCQEKSGYSSDYCFSGGTYDPSSSST 153

Y ++G+ Q V+ DTGSS+LWV S CQ ++ C S ++PS SST

Sbjct 71 AAYFGEISIGTPPQNFLVLFDTGSSNLWV--PSVYCQSQA------CTSHSRFNPSESST 122

Query 154 IQELGKSFNIRYGDGSSSSGTWVKDTVGINGAIILNQQFGDVNSTSVS-------QGILG 206

G++F+++YG G S +G + DT+ + + NQ+FG + + GI+G

Sbjct 123 YSTNGQTFSLQYGSG-SLTGFFGYDTLTVQSIQVPNQEFGLSENEPGTNFVYAQFDGIMG 181

Query 207 I---GLDTNESTDTIYENFPINLKEQGFINTNAYSLYL-NAPSATSGTIIFGGIDHAKYT 262

+ L +E+T + + ++G + + +S+YL N ++ G ++FGG+D + YT

Sbjct 182 LAYPALSVDEATTAMQ-----GMVQEGALTSPVFSVYLSNQQGSSGGAVVFGGVDSSLYT 236

Query 263 GSLTTLPLTSNREFTIQTNSATVG---TSTIDINTGLLLDSGTTLTYLPQSVVDSIANAI 319

G + P+T + I +G + ++D+GT+L +PQ + ++ A

Sbjct 237 GQIYWAPVTQELYWQIGIEEFLIGGQASGWCSEGCQAIVDTGTSLLTVPQQYMSALLQAT 296

Query 320 GGDITYNRPIGAYIWSCNRNGKVTYNFPQ------GLNIDIPYSDLAVPLYYSNGAVAGF 373

G G ++ +CN N P G+ +P S + SN G+

Sbjct 297 GAQ---EDEYGQFLVNCNS----IQNLPSLTFIINGVEFPLPPSSYIL----SNN---GY 342

Query 374 CALGI-------LYGENFNILGDNFLRHAYVVYNL 401

C +G+ G+ ILGD FLR Y VY+L

Sbjct 343 CTVGVEPTYLSSQNGQPLWILGDVFLRSYYSVYDL 377

>Activation Intermediate 2 Of Human Gastricsin From Human Stomach [Homo sapiens]

Sequence ID: 1AVF_A Length: 329

>Activation Intermediate 2 Of Human Gastricsin From Human Stomach [Homo sapiens]

Sequence ID: 1AVF_J Length: 329 >Crystal And Molecular Structures Of Human Progastricsin At 1.62 Angstroms Resolution [Homo sapiens]

Sequence ID: 1HTR_B Length: 329

Range 1: 12 to 318

Score:97.8 bits(242), Expect:1e-21,

Method:Compositional matrix adjust.,

Identities:91/335(27%), Positives:155/335(46%), Gaps:55/335(16%)

Query 94 VTYSANFTVGSNSQKQNVIVDTGSSDLWVVDSSANCQEKSGYSSDYCFSGGTYDPSSSST 153

Y ++G+ Q V+ DTGSS+LWV S CQ ++ C S ++PS SST

Sbjct 12 AAYFGEISIGTPPQNFLVLFDTGSSNLWV--PSVYCQSQA------CTSHSRFNPSESST 63

Query 154 IQELGKSFNIRYGDGSSSSGTWVKDTVGINGAIILNQQFGDVNSTSVS-------QGILG 206

G++F+++YG G S +G + DT+ + + NQ+FG + + GI+G

Sbjct 64 YSTNGQTFSLQYGSG-SLTGFFGYDTLTVQSIQVPNQEFGLSENEPGTNFVYAQFDGIMG 122

Query 207 I---GLDTNESTDTIYENFPINLKEQGFINTNAYSLYL-NAPSATSGTIIFGGIDHAKYT 262

+ L +E+T + + ++G + + +S+YL N ++ G ++FGG+D + YT

Sbjct 123 LAYPALSVDEATTAMQ-----GMVQEGALTSPVFSVYLSNQQGSSGGAVVFGGVDSSLYT 177

Query 263 GSLTTLPLTSNREFTIQTNSATVG---TSTIDINTGLLLDSGTTLTYLPQSVVDSIANAI 319

G + P+T + I +G + ++D+GT+L +PQ + ++ A

Sbjct 178 GQIYWAPVTQELYWQIGIEEFLIGGQASGWCSEGCQAIVDTGTSLLTVPQQYMSALLQAT 237

Query 320 GGDITYNRPIGAYIWSCNRNGKVTYNFPQ------GLNIDIPYSDLAVPLYYSNGAVAGF 373

G G ++ +CN N P G+ +P S + SN G+

Sbjct 238 GAQ---EDEYGQFLVNCNS----IQNLPSLTFIINGVEFPLPPSSYIL----SNN---GY 283

Query 374 CALGI-------LYGENFNILGDNFLRHAYVVYNL 401

C +G+ G+ ILGD FLR Y VY+L

Sbjct 284 CTVGVEPTYLSSQNGQPLWILGDVFLRSYYSVYDL 318

>unnamed protein product [Homo sapiens]

Sequence ID: BAF84553.1 Length: 388

Range 1: 74 to 387

Score:98.2 bits(243), Expect:2e-21,

Method:Compositional matrix adjust.,

Identities:102/336(30%), Positives:159/336(47%), Gaps:40/336(11%)

Query 94 VTYSANFTVGSNSQKQNVIVDTGSSDLWVVDSSANCQEKSGYSSDYCFSGGTYDPSSSST 153

+ Y +G+ +Q V+ DTGSS+LWV S C SS C + ++P SST

Sbjct 74 MEYFGTIGIGTPAQDFTVVFDTGSSNLWV--PSVYC------SSLACTNHNRFNPEDSST 125

Query 154 IQELGKSFNIRYGDGSSSSGTWVKDTVGINGAIILNQQFGDVNSTSVS-------QGILG 206

Q ++ +I YG GS + G DTV + G NQ FG + S GILG

Sbjct 126 YQSTSETVSITYGTGSMT-GILGYDTVQVGGISDTNQIFGLSETEPGSFLYYAPFDGILG 184

Query 207 IGLDTNESTDTIYENFPI--NLKEQGFINTNAYSLYLNAPSATSGTIIFGGIDHAKYTGS 264

+ + S+ P+ N+ QG ++ + +S+YL+A + +IFGGID + YTGS

Sbjct 185 LAYPSISSSGAT----PVFDNIWNQGLVSQDLFSVYLSADDQSGSVVIFGGIDSSYYTGS 240

Query 265 LTTLPLTSNREFTIQTNSATVGTSTIDINTGL--LLDSGTTLTYLPQSVVDSIANAIGGD 322

L +P+T + I +S T+ I G ++D+GT+L P S + +I + IG

Sbjct 241 LNWVPVTVEGYWQITVDSITMNGEAIACAEGCQAIVDTGTSLLTGPTSPITNIQSDIGAS 300

Query 323 ITYNRPIGAYIWSCNRNGKV-----TYNFPQGLNIDIPYSDLAVPLYYSNGAVAGFCALG 377

+ G + SC+ + T N G+ +P S A L ++GF +

Sbjct 301 ENSD---GDMVVSCSAISSLPDIVFTIN---GVQYPVPPS--AYILQSEGSCISGFQGMN 352

Query 378 ILY--GENFNILGDNFLRHAYVVYNLDALTISLAPV 411

+ GE + ILGD F+R + V++ + LAPV

Sbjct 353 LPTESGELW-ILGDVFIRQYFTVFDRANNQVGLAPV 387

>Crystal Structure Of Human Uropepsin At 2.45 A Resolution [Homo sapiens]

Sequence ID: 1FLH_A Length: 326

Range 1: 12 to 325

Score:97.4 bits(241), Expect:2e-21,

Method:Compositional matrix adjust.,

Identities:102/336(30%), Positives:159/336(47%), Gaps:40/336(11%)

Query 94 VTYSANFTVGSNSQKQNVIVDTGSSDLWVVDSSANCQEKSGYSSDYCFSGGTYDPSSSST 153

+ Y +G+ +Q V+ DTGSS+LWV S C SS C + ++P SST

Sbjct 12 MEYFGTIGIGTPAQDFTVVFDTGSSNLWV--PSVYC------SSLACTNHNRFNPEDSST 63

Query 154 IQELGKSFNIRYGDGSSSSGTWVKDTVGINGAIILNQQFGDVNSTSVS-------QGILG 206

Q ++ +I YG GS + G DTV + G NQ FG + S GILG

Sbjct 64 YQSTSETVSITYGTGSMT-GILGYDTVQVGGISDTNQIFGLSETEPGSFLYYAPFDGILG 122

Query 207 IGLDTNESTDTIYENFPI--NLKEQGFINTNAYSLYLNAPSATSGTIIFGGIDHAKYTGS 264

+ + S+ P+ N+ QG ++ + +S+YL+A + +IFGGID + YTGS

Sbjct 123 LAYPSISSSGAT----PVFDNIWNQGLVSQDLFSVYLSADDQSGSVVIFGGIDSSYYTGS 178

Query 265 LTTLPLTSNREFTIQTNSATVGTSTIDINTGL--LLDSGTTLTYLPQSVVDSIANAIGGD 322

L +P+T + I +S T+ I G ++D+GT+L P S + +I + IG

Sbjct 179 LNWVPVTVEGYWQITVDSITMNGEAIACAEGCQAIVDTGTSLLTGPTSPIANIQSDIGAS 238

Query 323 ITYNRPIGAYIWSCNRNGKV-----TYNFPQGLNIDIPYSDLAVPLYYSNGAVAGFCALG 377

+ G + SC+ + T N G+ +P S A L ++GF +

Sbjct 239 ENSD---GDMVVSCSAISSLPDIVFTIN---GVQYPVPPS--AYILQSEGSCISGFQGMN 290

Query 378 ILY--GENFNILGDNFLRHAYVVYNLDALTISLAPV 411

+ GE + ILGD F+R + V++ + LAPV

Sbjct 291 VPTESGELW-ILGDVFIRQYFTVFDRANNQVGLAPV 325

>pepsin A-4 preproprotein [Homo sapiens]

Sequence ID: NP_001073276.1 Length: 388

>RecName: Full=Pepsin A-4; AltName: Full=Pepsinogen-4; Flags: Precursor [Homo sapiens]

Sequence ID: P0DJD7.1 Length: 388 >pepsinogen [Homo sapiens]

Sequence ID: AAA98529.1 Length: 388 >Pepsinogen 4, group I (pepsinogen A) [Homo sapiens]

Sequence ID: AAI50660.1 Length: 388 >Pepsinogen 4, group I (pepsinogen A) [Homo sapiens]

Sequence ID: AAI71910.1 Length: 388 >Pepsinogen 4, group I (pepsinogen A) [Homo sapiens]

Sequence ID: AAI71920.1 Length: 388

Range 1: 74 to 387

Score:98.2 bits(243), Expect:2e-21,

Method:Compositional matrix adjust.,

Identities:102/336(30%), Positives:159/336(47%), Gaps:40/336(11%)

Query 94 VTYSANFTVGSNSQKQNVIVDTGSSDLWVVDSSANCQEKSGYSSDYCFSGGTYDPSSSST 153

+ Y +G+ +Q V+ DTGSS+LWV S C SS C + ++P SST

Sbjct 74 MEYFGTIGIGTPAQDFTVVFDTGSSNLWV--PSVYC------SSLACTNHNRFNPEDSST 125

Query 154 IQELGKSFNIRYGDGSSSSGTWVKDTVGINGAIILNQQFGDVNSTSVS-------QGILG 206

Q ++ +I YG GS + G DTV + G NQ FG + S GILG

Sbjct 126 YQSTSETVSITYGTGSMT-GILGYDTVQVGGISDTNQIFGLSETEPGSFLYYAPFDGILG 184

Query 207 IGLDTNESTDTIYENFPI--NLKEQGFINTNAYSLYLNAPSATSGTIIFGGIDHAKYTGS 264

+ + S+ P+ N+ QG ++ + +S+YL+A + +IFGGID + YTGS

Sbjct 185 LAYPSISSSGAT----PVFDNIWNQGLVSQDLFSVYLSADDQSGSVVIFGGIDSSYYTGS 240

Query 265 LTTLPLTSNREFTIQTNSATVGTSTIDINTGL--LLDSGTTLTYLPQSVVDSIANAIGGD 322

L +P+T + I +S T+ I G ++D+GT+L P S + +I + IG

Sbjct 241 LNWVPVTVEGYWQITVDSITMNGEAIACAEGCQAIVDTGTSLLTGPTSPIANIQSDIGAS 300

Query 323 ITYNRPIGAYIWSCNRNGKV-----TYNFPQGLNIDIPYSDLAVPLYYSNGAVAGFCALG 377

+ G + SC+ + T N G+ +P S A L ++GF +

Sbjct 301 ENSD---GDMVVSCSAISSLPDIVFTIN---GVQYPVPPS--AYILQSEGSCISGFQGMN 352

Query 378 ILY--GENFNILGDNFLRHAYVVYNLDALTISLAPV 411

+ GE + ILGD F+R + V++ + LAPV

Sbjct 353 LPTESGELW-ILGDVFIRQYFTVFDRANNQVGLAPV 387

>pepsin A-3 preproprotein [Homo sapiens]

Sequence ID: NP_001073275.1 Length: 388

>RecName: Full=Pepsin A-3; AltName: Full=Pepsinogen-3; Flags: Precursor [Homo sapiens]

Sequence ID: P0DJD8.1 Length: 388

Range 1: 74 to 387

Score:98.2 bits(243), Expect:3e-21,

Method:Compositional matrix adjust.,

Identities:102/336(30%), Positives:159/336(47%), Gaps:40/336(11%)

Query 94 VTYSANFTVGSNSQKQNVIVDTGSSDLWVVDSSANCQEKSGYSSDYCFSGGTYDPSSSST 153

+ Y +G+ +Q V+ DTGSS+LWV S C SS C + ++P SST

Sbjct 74 MEYFGTIGIGTPAQDFTVVFDTGSSNLWV--PSVYC------SSLACTNHNRFNPEDSST 125

Query 154 IQELGKSFNIRYGDGSSSSGTWVKDTVGINGAIILNQQFGDVNSTSVS-------QGILG 206

Q ++ +I YG GS + G DTV + G NQ FG + S GILG

Sbjct 126 YQSTSETVSITYGTGSMT-GILGYDTVQVGGISDTNQIFGLSETEPGSFLYYAPFDGILG 184

Query 207 IGLDTNESTDTIYENFPI--NLKEQGFINTNAYSLYLNAPSATSGTIIFGGIDHAKYTGS 264

+ + S+ P+ N+ QG ++ + +S+YL+A + +IFGGID + YTGS

Sbjct 185 LAYPSISSSGAT----PVFDNIWNQGLVSQDLFSVYLSADDQSGSVVIFGGIDSSYYTGS 240

Query 265 LTTLPLTSNREFTIQTNSATVGTSTIDINTGL--LLDSGTTLTYLPQSVVDSIANAIGGD 322

L +P+T + I +S T+ I G ++D+GT+L P S + +I + IG

Sbjct 241 LNWVPVTVEGYWQITVDSITMNGEAIACAEGCQAIVDTGTSLLTGPTSPIANIQSDIGAS 300

Query 323 ITYNRPIGAYIWSCNRNGKV-----TYNFPQGLNIDIPYSDLAVPLYYSNGAVAGFCALG 377

+ G + SC+ + T N G+ +P S A L ++GF +

Sbjct 301 ENSD---GDMVVSCSAISSLPDIVFTIN---GVQYPVPPS--AYILQSEGSCISGFQGMN 352

Query 378 ILY--GENFNILGDNFLRHAYVVYNLDALTISLAPV 411

+ GE + ILGD F+R + V++ + LAPV

Sbjct 353 LPTESGELW-ILGDVFIRQYFTVFDRANNQVGLAPV 387

>The Crystal Structure Of Human Pepsin And Its Complex With Pepstatin [Homo sapiens]

Sequence ID: 1PSN_A Length: 326

>The crystal structure of human pepsin and its complex with pepstatin [Homo sapiens]

Sequence ID: 1PSO_E Length: 326 >Human pepsin 3A in complex with a phosphonate inhibitor IVA-VAL-VAL-LEU(P)-(O)PHE-ALA-ALA-OME [Homo sapiens]

Sequence ID: 1QRP_E Length: 326 >Human pepsin 3b [Homo sapiens]

Sequence ID: 3UTL_A Length: 326

Range 1: 12 to 325

Score:97.1 bits(240), Expect:3e-21,

Method:Compositional matrix adjust.,

Identities:102/336(30%), Positives:159/336(47%), Gaps:40/336(11%)

Query 94 VTYSANFTVGSNSQKQNVIVDTGSSDLWVVDSSANCQEKSGYSSDYCFSGGTYDPSSSST 153

+ Y +G+ +Q V+ DTGSS+LWV S C SS C + ++P SST

Sbjct 12 MEYFGTIGIGTPAQDFTVVFDTGSSNLWV--PSVYC------SSLACTNHNRFNPEDSST 63

Query 154 IQELGKSFNIRYGDGSSSSGTWVKDTVGINGAIILNQQFGDVNSTSVS-------QGILG 206

Q ++ +I YG GS + G DTV + G NQ FG + S GILG

Sbjct 64 YQSTSETVSITYGTGSMT-GILGYDTVQVGGISDTNQIFGLSETEPGSFLYYAPFDGILG 122

Query 207 IGLDTNESTDTIYENFPI--NLKEQGFINTNAYSLYLNAPSATSGTIIFGGIDHAKYTGS 264

+ + S+ P+ N+ QG ++ + +S+YL+A + +IFGGID + YTGS

Sbjct 123 LAYPSISSSGAT----PVFDNIWNQGLVSQDLFSVYLSADDQSGSVVIFGGIDSSYYTGS 178

Query 265 LTTLPLTSNREFTIQTNSATVGTSTIDINTGL--LLDSGTTLTYLPQSVVDSIANAIGGD 322

L +P+T + I +S T+ I G ++D+GT+L P S + +I + IG

Sbjct 179 LNWVPVTVEGYWQITVDSITMNGEAIACAEGCQAIVDTGTSLLTGPTSPIANIQSDIGAS 238

Query 323 ITYNRPIGAYIWSCNRNGKV-----TYNFPQGLNIDIPYSDLAVPLYYSNGAVAGFCALG 377

+ G + SC+ + T N G+ +P S A L ++GF +

Sbjct 239 ENSD---GDMVVSCSAISSLPDIVFTIN---GVQYPVPPS--AYILQSEGSCISGFQGMN 290

Query 378 ILY--GENFNILGDNFLRHAYVVYNLDALTISLAPV 411

+ GE + ILGD F+R + V++ + LAPV

Sbjct 291 LPTESGELW-ILGDVFIRQYFTVFDRANNQVGLAPV 325

>Pepsinogen 4, group I (pepsinogen A) [Homo sapiens]

Sequence ID: AAI71808.1 Length: 388

Range 1: 74 to 387

Score:97.8 bits(242), Expect:3e-21,

Method:Compositional matrix adjust.,

Identities:102/336(30%), Positives:159/336(47%), Gaps:40/336(11%)

Query 94 VTYSANFTVGSNSQKQNVIVDTGSSDLWVVDSSANCQEKSGYSSDYCFSGGTYDPSSSST 153

+ Y +G+ +Q V+ DTGSS+LWV S C SS C + ++P SST

Sbjct 74 MEYFGTIGIGTPAQDFTVLFDTGSSNLWV--PSVYC------SSLACTNHNRFNPEDSST 125

Query 154 IQELGKSFNIRYGDGSSSSGTWVKDTVGINGAIILNQQFGDVNSTSVS-------QGILG 206

Q ++ +I YG GS + G DTV + G NQ FG + S GILG

Sbjct 126 YQSTSETVSITYGTGSMT-GILGYDTVQVGGISDTNQIFGLSETEPGSFLYYAPFDGILG 184

Query 207 IGLDTNESTDTIYENFPI--NLKEQGFINTNAYSLYLNAPSATSGTIIFGGIDHAKYTGS 264

+ + S+ P+ N+ QG ++ + +S+YL+A + +IFGGID + YTGS

Sbjct 185 LAYPSISSSGAT----PVFDNIWNQGLVSQDLFSVYLSADDQSGSVVIFGGIDSSYYTGS 240

Query 265 LTTLPLTSNREFTIQTNSATVGTSTIDINTGL--LLDSGTTLTYLPQSVVDSIANAIGGD 322

L +P+T + I +S T+ I G ++D+GT+L P S + +I + IG

Sbjct 241 LNWVPVTVEGYWQITVDSITMNGEAIACAEGCQAIVDTGTSLLTGPTSPIANIQSDIGAS 300

Query 323 ITYNRPIGAYIWSCNRNGKV-----TYNFPQGLNIDIPYSDLAVPLYYSNGAVAGFCALG 377

+ G + SC+ + T N G+ +P S A L ++GF +

Sbjct 301 ENSD---GDMVVSCSAISSLPDIVFTIN---GVQYPVPPS--AYILQSEGSCISGFQGMN 352

Query 378 ILY--GENFNILGDNFLRHAYVVYNLDALTISLAPV 411

+ GE + ILGD F+R + V++ + LAPV

Sbjct 353 LPTESGELW-ILGDVFIRQYFTVFDRTNNQVGLAPV 387

>Pepsinogen 4, group I (pepsinogen A) [Homo sapiens]

Sequence ID: AAI71814.1 Length: 388

Range 1: 74 to 387

Score:97.8 bits(242), Expect:3e-21,

Method:Compositional matrix adjust.,

Identities:102/336(30%), Positives:159/336(47%), Gaps:40/336(11%)

Query 94 VTYSANFTVGSNSQKQNVIVDTGSSDLWVVDSSANCQEKSGYSSDYCFSGGTYDPSSSST 153

+ Y +G+ +Q V+ DTGSS+LWV S C SS C + ++P SST

Sbjct 74 MEYFGTIGIGTPAQDFTVLFDTGSSNLWV--PSVYC------SSLACTNHNRFNPEDSST 125

Query 154 IQELGKSFNIRYGDGSSSSGTWVKDTVGINGAIILNQQFGDVNSTSVS-------QGILG 206

Q ++ +I YG GS + G DTV + G NQ FG + S GILG

Sbjct 126 YQSTSETVSITYGTGSMT-GILGYDTVQVGGISDTNQIFGLSETEPGSFLYYAPFDGILG 184

Query 207 IGLDTNESTDTIYENFPI--NLKEQGFINTNAYSLYLNAPSATSGTIIFGGIDHAKYTGS 264

+ + S+ P+ N+ QG ++ + +S+YL+A + +IFGGID + YTGS

Sbjct 185 LAYPSISSSGAT----PVFDNIWNQGLVSQDLFSVYLSADDQSGSVVIFGGIDSSYYTGS 240

Query 265 LTTLPLTSNREFTIQTNSATVGTSTIDINTGL--LLDSGTTLTYLPQSVVDSIANAIGGD 322

L +P+T + I +S T+ I G ++D+GT+L P S + +I + IG

Sbjct 241 LNWVPVTVEGYWQITVDSITMNGEAIACAEGCQAIVDTGTSLLTGPTSPIANIQSDIGAS 300

Query 323 ITYNRPIGAYIWSCNRNGKV-----TYNFPQGLNIDIPYSDLAVPLYYSNGAVAGFCALG 377

+ G + SC+ + T N G+ +P S A L ++GF +

Sbjct 301 ENSD---GDMVVSCSAISSLPDIVFTIN---GVQYPVPPS--AYILQSEGSCISGFQGMN 352

Query 378 ILY--GENFNILGDNFLRHAYVVYNLDALTISLAPV 411

+ GE + ILGD F+R + V++ + LAPV

Sbjct 353 LPTESGELW-ILGDVFIRQYFTVFDRANNQVGLAPV 387

>Pepsinogen 3, group I (pepsinogen A) [Homo sapiens]

Sequence ID: AAI71815.1 Length: 388

>unnamed protein product [Homo sapiens]

Sequence ID: BAG35783.1 Length: 388 >unnamed protein product [Homo sapiens]

Sequence ID: BAG54225.1 Length: 388

Range 1: 74 to 387

Score:97.4 bits(241), Expect:4e-21,

Method:Compositional matrix adjust.,

Identities:102/336(30%), Positives:159/336(47%), Gaps:40/336(11%)

Query 94 VTYSANFTVGSNSQKQNVIVDTGSSDLWVVDSSANCQEKSGYSSDYCFSGGTYDPSSSST 153

+ Y +G+ +Q V+ DTGSS+LWV S C SS C + ++P SST

Sbjct 74 MEYFGTIGIGTPAQDFTVLFDTGSSNLWV--PSVYC------SSLACTNHNRFNPEDSST 125

Query 154 IQELGKSFNIRYGDGSSSSGTWVKDTVGINGAIILNQQFGDVNSTSVS-------QGILG 206

Q ++ +I YG GS + G DTV + G NQ FG + S GILG

Sbjct 126 YQSTSETVSITYGTGSMT-GILGYDTVQVGGISDTNQIFGLSETEPGSFLYYAPFDGILG 184

Query 207 IGLDTNESTDTIYENFPI--NLKEQGFINTNAYSLYLNAPSATSGTIIFGGIDHAKYTGS 264

+ + S+ P+ N+ QG ++ + +S+YL+A + +IFGGID + YTGS

Sbjct 185 LAYPSISSSGAT----PVFDNIWNQGLVSQDLFSVYLSADDQSGSVVIFGGIDSSYYTGS 240

Query 265 LTTLPLTSNREFTIQTNSATVGTSTIDINTGL--LLDSGTTLTYLPQSVVDSIANAIGGD 322

L +P+T + I +S T+ I G ++D+GT+L P S + +I + IG

Sbjct 241 LNWVPVTVEGYWQITVDSITMNGEAIACAEGCQAIVDTGTSLLTGPTSPIANIQSDIGAS 300

Query 323 ITYNRPIGAYIWSCNRNGKV-----TYNFPQGLNIDIPYSDLAVPLYYSNGAVAGFCALG 377

+ G + SC+ + T N G+ +P S A L ++GF +

Sbjct 301 ENSD---GDMVVSCSAISSLPDIVFTIN---GVQYPVPPS--AYILQSEGSCISGFQGMN 352

Query 378 ILY--GENFNILGDNFLRHAYVVYNLDALTISLAPV 411

+ GE + ILGD F+R + V++ + LAPV

Sbjct 353 LPTESGELW-ILGDVFIRQYFTVFDRANNQVGLAPV 387

>pepsinogen A [Homo sapiens]

Sequence ID: AAA60061.1 Length: 388

Range 1: 74 to 387

Score:97.4 bits(241), Expect:4e-21,

Method:Compositional matrix adjust.,

Identities:102/336(30%), Positives:158/336(47%), Gaps:40/336(11%)

Query 94 VTYSANFTVGSNSQKQNVIVDTGSSDLWVVDSSANCQEKSGYSSDYCFSGGTYDPSSSST 153

+ Y +G+ +Q V+ DTGSS+LWV S C SS C + ++P SST

Sbjct 74 MEYFGTIGIGTPAQDFTVLFDTGSSNLWV--PSVYC------SSLACTNHNRFNPEDSST 125

Query 154 IQELGKSFNIRYGDGSSSSGTWVKDTVGINGAIILNQQFGDVNSTSVS-------QGILG 206

Q ++ +I YG GS + G DTV + G NQ FG + S GILG

Sbjct 126 YQSTSETVSITYGTGSMT-GILGYDTVQVGGISDTNQIFGLSETEPGSFLYYAPFDGILG 184

Query 207 IGLDTNESTDTIYENFPI--NLKEQGFINTNAYSLYLNAPSATSGTIIFGGIDHAKYTGS 264

+ + S+ P+ N+ QG ++ + +S+YL+A + +IFGGID + YTGS

Sbjct 185 LAYPSISSSGAT----PVFDNIWNQGLVSQDLFSVYLSADDQSGSVVIFGGIDSSYYTGS 240

Query 265 LTTLPLTSNREFTIQTNSATVGTSTIDINTGL--LLDSGTTLTYLPQSVVDSIANAIGGD 322

L +P+T + I +S T+ I G ++D+GT+L P S + +I + IG

Sbjct 241 LNWVPVTVEGYWQITVDSITMNGEAIACAEGCQAIVDTGTSLLTGPTSPIANIQSDIGAS 300

Query 323 ITYNRPIGAYIWSCNRNGKV-----TYNFPQGLNIDIPYSDLAVPLYYSNGAVAGFCALG 377

+ G + SC+ + T N G+ +P S A L ++GF +

Sbjct 301 ENSD---GDMVVSCSAISSLPDIVFTIN---GVQYPVPPS--AYILQSEGSCISGFQGMN 352

Query 378 ILY--GENFNILGDNFLRHAYVVYNLDALTISLAPV 411

+ GE + ILGD F+R + V+ + LAPV

Sbjct 353 LPTESGELW-ILGDVFIRQYFTVFERANNQVGLAPV 387

>napsin A aspartic peptidase, isoform CRA_c [Homo sapiens]

Sequence ID: EAW71849.1 Length: 328

Range 1: 60 to 305

Score:89.4 bits(220), Expect:1e-18,

Method:Compositional matrix adjust.,

Identities:80/255(31%), Positives:114/255(44%), Gaps:21/255(8%)

Query 79 SKRDDYISVELYN-EQVTYSANFTVGSNSQKQNVIVDTGSSDLWVVDSSANCQEKSGYSS 137

S D I V L N V Y +G+ Q V DTGSS+LWV S C + S

Sbjct 60 SPGDKPIFVPLSNYRDVQYFGEIGLGTPPQNFTVAFDTGSSNLWV--PSRRCH----FFS 113

Query 138 DYCFSGGTYDPSSSSTIQELGKSFNIRYGDGSSSSGTWVKDTV---GINGA-IILNQQFG 193

C+ +DP +SS+ Q G F I+YG G G +D + GI GA +I +

Sbjct 114 VPCWLHHRFDPKASSSFQANGTKFAIQYGTG-RVDGILSEDKLTIGGIKGASVIFGEALW 172

Query 194 D---VNSTSVSQGILGIGLDTNESTDTIYENFPINLKEQGFINTNAYSLYLN--APSATS 248

+ V + + GILG+G S + + + L EQG ++ +S YLN

Sbjct 173 EPSLVFAFAHFDGILGLGFPIL-SVEGVRPPMDV-LVEQGLLDKPVFSFYLNRDPEEPDG 230

Query 249 GTIIFGGIDHAKYTGSLTTLPLTSNREFTIQTNSATVGTSTIDINTGL--LLDSGTTLTY 306

G ++ GG D A Y LT +P+T + I VG G +LD+GT+L

Sbjct 231 GELVLGGSDPAHYIPPLTFVPVTVPAYWQIHMERVKVGPGLTLCAKGCAAILDTGTSLIT 290

Query 307 LPQSVVDSIANAIGG 321

P + ++ AIGG

Sbjct 291 GPTEEIRALHAAIGG 305

>napsin-A preproprotein [Homo sapiens]

Sequence ID: NP_004842.1 Length: 420

>napsin-A isoform X1 [Homo sapiens]

Sequence ID: XP_011525842.1 Length: 420 >RecName: Full=Napsin-A; AltName: Full=Aspartyl protease 4; Short=ASP4; Short=Asp 4; AltName: Full=Napsin-1; AltName: Full=TA01/TA02; Flags: Precursor [Homo sapiens]

Sequence ID: O96009.1 Length: 420 >napsin A [Homo sapiens]

Sequence ID: AAD04917.1 Length: 420 >napsin 1 precursor [Homo sapiens]

Sequence ID: AAD13215.1 Length: 420 >aspartyl protease 4 [Homo sapiens]

Sequence ID: AAF17081.1 Length: 420 >napsin A aspartic peptidase, isoform CRA_a [Homo sapiens]

Sequence ID: EAW71847.1 Length: 420

Range 1: 60 to 323

Score:90.1 bits(222), Expect:2e-18,

Method:Compositional matrix adjust.,

Identities:85/276(31%), Positives:121/276(43%), Gaps:24/276(8%)

Query 79 SKRDDYISVELYN-EQVTYSANFTVGSNSQKQNVIVDTGSSDLWVVDSSANCQEKSGYSS 137

S D I V L N V Y +G+ Q V DTGSS+LWV S C + S

Sbjct 60 SPGDKPIFVPLSNYRDVQYFGEIGLGTPPQNFTVAFDTGSSNLWV--PSRRCH----FFS 113

Query 138 DYCFSGGTYDPSSSSTIQELGKSFNIRYGDGSSSSGTWVKDTV---GINGA-IILNQQFG 193

C+ +DP +SS+ Q G F I+YG G G +D + GI GA +I +

Sbjct 114 VPCWLHHRFDPKASSSFQANGTKFAIQYGTG-RVDGILSEDKLTIGGIKGASVIFGEALW 172

Query 194 D---VNSTSVSQGILGIGLDTNESTDTIYENFPINLKEQGFINTNAYSLYLN--APSATS 248

+ V + + GILG+G S + + + L EQG ++ +S YLN

Sbjct 173 EPSLVFAFAHFDGILGLGFPI-LSVEGVRPPMDV-LVEQGLLDKPVFSFYLNRDPEEPDG 230

Query 249 GTIIFGGIDHAKYTGSLTTLPLTSNREFTIQTNSATVGTSTIDINTGL--LLDSGTTLTY 306

G ++ GG D A Y LT +P+T + I VG G +LD+GT+L

Sbjct 231 GELVLGGSDPAHYIPPLTFVPVTVPAYWQIHMERVKVGPGLTLCAKGCAAILDTGTSLIT 290

Query 307 LPQSVVDSIANAIGGDITYNRPIGAYIWSCNRNGKV 342

P + ++ AIGG G YI C+ K+

Sbjct 291 GPTEEIRALHAAIGGIPLLA---GEYIILCSEIPKL 323

>Napsin A aspartic peptidase [Homo sapiens]

Sequence ID: AAH17842.1 Length: 420

Range 1: 60 to 323

Score:89.7 bits(221), Expect:2e-18,

Method:Compositional matrix adjust.,

Identities:85/276(31%), Positives:121/276(43%), Gaps:24/276(8%)

Query 79 SKRDDYISVELYN-EQVTYSANFTVGSNSQKQNVIVDTGSSDLWVVDSSANCQEKSGYSS 137

S D I V L N V Y +G+ Q V DTGSS+LWV S C + S

Sbjct 60 SPGDKPIFVPLSNYRDVQYFGEIGLGTPPQNFTVAFDTGSSNLWV--PSRRCH----FFS 113

Query 138 DYCFSGGTYDPSSSSTIQELGKSFNIRYGDGSSSSGTWVKDTV---GINGA-IILNQQFG 193

C+ +DP +SS+ Q G F I+YG G G +D + GI GA +I +

Sbjct 114 VPCWLHHRFDPKASSSFQANGTKFAIQYGTG-RVDGILSEDKLTIGGIKGASVIFGEALW 172

Query 194 D---VNSTSVSQGILGIGLDTNESTDTIYENFPINLKEQGFINTNAYSLYLN--APSATS 248

+ V + + GILG+G S + + + L EQG ++ +S YLN

Sbjct 173 EPSLVFAFAHFDGILGLGFPI-LSVEGVRPPMDV-LVEQGLLDKPVFSFYLNRDPEEPDG 230

Query 249 GTIIFGGIDHAKYTGSLTTLPLTSNREFTIQTNSATVGTSTIDINTGL--LLDSGTTLTY 306

G ++ GG D A Y LT +P+T + I VG G +LD+GT+L

Sbjct 231 GELVLGGSDPAHYIPPLTFVPVTVPAYWQIHMERVKVGPGLTLCAKGCAAILDTGTSLIT 290

Query 307 LPQSVVDSIANAIGGDITYNRPIGAYIWSCNRNGKV 342

P + ++ AIGG G YI C+ K+

Sbjct 291 GPTEEIRALHAAIGGIPLLA---GEYIILCSEIPKL 323

>napsin A aspartic peptidase, isoform CRA_b [Homo sapiens]

Sequence ID: EAW71848.1 Length: 357

Range 1: 3 to 260

Score:87.8 bits(216), Expect:6e-18,

Method:Compositional matrix adjust.,

Identities:83/270(31%), Positives:119/270(44%), Gaps:24/270(8%)

Query 85 ISVELYN-EQVTYSANFTVGSNSQKQNVIVDTGSSDLWVVDSSANCQEKSGYSSDYCFSG 143

I V L N V Y +G+ Q V DTGSS+LWV S C + S C+

Sbjct 3 IFVPLSNYRDVQYFGEIGLGTPPQNFTVAFDTGSSNLWV--PSRRCH----FFSVPCWLH 56

Query 144 GTYDPSSSSTIQELGKSFNIRYGDGSSSSGTWVKDTV---GINGA-IILNQQFGD---VN 196

+DP +SS+ Q G F I+YG G G +D + GI GA +I + + V

Sbjct 57 HRFDPKASSSFQANGTKFAIQYGTG-RVDGILSEDKLTIGGIKGASVIFGEALWEPSLVF 115

Query 197 STSVSQGILGIGLDTNESTDTIYENFPINLKEQGFINTNAYSLYLN--APSATSGTIIFG 254

+ + GILG+G S + + + L EQG ++ +S YLN G ++ G

Sbjct 116 AFAHFDGILGLGFPI-LSVEGVRPPMDV-LVEQGLLDKPVFSFYLNRDPEEPDGGELVLG 173

Query 255 GIDHAKYTGSLTTLPLTSNREFTIQTNSATVGTSTIDINTGL--LLDSGTTLTYLPQSVV 312

G D A Y LT +P+T + I VG G +LD+GT+L P +

Sbjct 174 GSDPAHYIPPLTFVPVTVPAYWQIHMERVKVGPGLTLCAKGCAAILDTGTSLITGPTEEI 233

Query 313 DSIANAIGGDITYNRPIGAYIWSCNRNGKV 342

++ AIGG G YI C+ K+

Sbjct 234 RALHAAIGGIPLLA---GEYIILCSEIPKL 260

>cathepsin E isoform c [Homo sapiens]

Sequence ID: NP_001304260.1 Length: 288

>unnamed protein product [Homo sapiens]

Sequence ID: BAG60360.1 Length: 288

Range 1: 1 to 183

Score:84.7 bits(208), Expect:3e-17,

Method:Compositional matrix adjust.,

Identities:65/197(33%), Positives:103/197(52%), Gaps:26/197(13%)

Query 94 VTYSANFTVGSNSQKQNVIVDTGSSDLWVVDSSANCQEKSGYSSDYCFSGGTYDPSSSST 153

+ Y ++GS Q VI DTGSS+LWV S C +S C + + PS SST

Sbjct 1 MEYFGTISIGSPPQNFTVIFDTGSSNLWV--PSVYC------TSPACKTHSRFQPSQSST 52

Query 154 IQELGKSFNIRYGDGSSSSGTWVKDTVGINGAIILNQQFGDVNSTSVSQ--------GIL 205

+ G+SF+I+YG G S SG D V + G ++ QQFG+ + T Q GIL

Sbjct 53 YSQPGQSFSIQYGTG-SLSGIIGADQVSVEGLTVVGQQFGE-SVTEPGQTFVDAEFDGIL 110

Query 206 GIGLDTNE--STDTIYENFPINLKEQGFINTNAYSLYLNA-PSATSGT-IIFGGIDHAKY 261

G+G + +++ N+ Q ++ +S+Y+++ P +G+ +IFGG DH+ +

Sbjct 111 GLGYPSLAVGGVTPVFD----NMMAQNLVDLPMFSVYMSSNPEGGAGSELIFGGYDHSHF 166

Query 262 TGSLTTLPLTSNREFTI 278

+GSL +P+T + I

Sbjct 167 SGSLNWVPVTKQAYWQI 183

>cathepsin E isoform b precursor [Homo sapiens]

Sequence ID: NP_683865.1 Length: 363

>cathepsin E, isoform CRA_b [Homo sapiens]

Sequence ID: EAW91593.1 Length: 363 >cathepsin E, alternative [Homo sapiens]

Sequence ID: CAB82849.1 Length: 363

Range 1: 44 to 258

Score:85.5 bits(210), Expect:4e-17,

Method:Compositional matrix adjust.,

Identities:72/236(31%), Positives:115/236(48%), Gaps:33/236(13%)

Query 55 ELYVNRNHDDSNFTIGPHFVVNEYSKRDDYISVELYNEQVTYSANFTVGSNSQKQNVIVD 114

E + + N D FT + +Y+ +E Y ++GS Q VI D

Sbjct 44 EFWKSHNLDMIQFTESCSMDQSAKEPLINYLDME-------YFGTISIGSPPQNFTVIFD 96

Query 115 TGSSDLWVVDSSANCQEKSGYSSDYCFSGGTYDPSSSSTIQELGKSFNIRYGDGSSSSGT 174

TGSS+LWV S C +S C + + PS SST + G+SF+I+YG G S SG

Sbjct 97 TGSSNLWV--PSVYC------TSPACKTHSRFQPSQSSTYSQPGQSFSIQYGTG-SLSGI 147

Query 175 WVKDTVGINGAIILNQQFGDVNSTSVSQ--------GILGIGLDTNE--STDTIYENFPI 224

D V + G ++ QQFG+ + T Q GILG+G + +++

Sbjct 148 IGADQVSVEGLTVVGQQFGE-SVTEPGQTFVDAEFDGILGLGYPSLAVGGVTPVFD---- 202

Query 225 NLKEQGFINTNAYSLYLNA-PSATSGT-IIFGGIDHAKYTGSLTTLPLTSNREFTI 278

N+ Q ++ +S+Y+++ P +G+ +IFGG DH+ ++GSL +P+T + I

Sbjct 203 NMMAQNLVDLPMFSVYMSSNPEGGAGSELIFGGYDHSHFSGSLNWVPVTKQAYWQI 258

>cathepsin E isoform X2 [Homo sapiens]

Sequence ID: XP_011507547.1 Length: 368

Range 1: 44 to 263

Score:79.0 bits(193), Expect:6e-15,

Method:Compositional matrix adjust.,

Identities:72/241(30%), Positives:115/241(47%), Gaps:38/241(15%)

Query 55 ELYVNRNHDDSNFTIGPHFVVNEYSKRDDYISVELYNEQVTYSANFTVGSNSQKQNVIVD 114

E + + N D FT + +Y+ +E Y ++GS Q VI D

Sbjct 44 EFWKSHNLDMIQFTESCSMDQSAKEPLINYLDME-------YFGTISIGSPPQNFTVIFD 96

Query 115 TGSSDLWVVDSSANCQEKSGYSSDYCFSGGTYDPSSSSTIQELGKSFNIRYGDGSSSSGT 174

TGSS+LWV S C +S C + + PS SST + G+SF+I+YG G S SG

Sbjct 97 TGSSNLWV--PSVYC------TSPACKTHSRFQPSQSSTYSQPGQSFSIQYGTG-SLSGI 147

Query 175 WVKDTVG-----INGAIILNQQFGDVNSTSVSQ--------GILGIGLDTNE--STDTIY 219

D V + G ++ QQFG+ + T Q GILG+G + ++

Sbjct 148 IGADQVSAFSYQVEGLTVVGQQFGE-SVTEPGQTFVDAEFDGILGLGYPSLAVGGVTPVF 206

Query 220 ENFPINLKEQGFINTNAYSLYLNA-PSATSGT-IIFGGIDHAKYTGSLTTLPLTSNREFT 277

+ N+ Q ++ +S+Y+++ P +G+ +IFGG DH+ ++GSL +P+T +

Sbjct 207 D----NMMAQNLVDLPMFSVYMSSNPEGGAGSELIFGGYDHSHFSGSLNWVPVTKQAYWQ 262

Query 278 I 278

I

Sbjct 263 I 263

>cathepsin D preproprotein [Homo sapiens]

Sequence ID: NP_001900.1 Length: 412

>RecName: Full=Cathepsin D; Contains: RecName: Full=Cathepsin D light chain; Contains: RecName: Full=Cathepsin D heavy chain; Flags: Precursor [Homo sapiens]

Sequence ID: P07339.1 Length: 412 >cathepsin D [Homo sapiens]

Sequence ID: AAA51922.1 Length: 412 >preprocathepsin D [Homo sapiens]

Sequence ID: AAB59529.1 Length: 412 >Cathepsin D [Homo sapiens]

Sequence ID: AAH16320.1 Length: 412

Range 1: 79 to 400

Score:74.7 bits(182), Expect:2e-13,

Method:Compositional matrix adjust.,

Identities:90/334(27%), Positives:137/334(41%), Gaps:39/334(11%)

Query 96 YSANFTVGSNSQKQNVIVDTGSSDLWVVDSSANCQEKSGYSSDYCFSGGTYDPSSSSTIQ 155

Y +G+ Q V+ DTGSS+LWV S +C+ C+ Y+ SST

Sbjct 79 YYGEIGIGTPPQCFTVVFDTGSSNLWV--PSIHCK----LLDIACWIHHKYNSDKSSTYV 132

Query 156 ELGKSFNIRYGDGSSSSGTWVKDTV-----------GINGAIILNQQFGDVNS------- 197

+ G SF+I YG G S SG +DTV + G + Q FG+

Sbjct 133 KNGTSFDIHYGSG-SLSGYLSQDTVSVPCQSASSASALGGVKVERQVFGEATKQPGITFI 191

Query 198 TSVSQGILGIGLDTNESTDTIYENFPINLKEQGFINTNAYSLYLNA-PSATS-GTIIFGG 255

+ GILG+ S + + F NL +Q ++ N +S YL+ P A G ++ GG

Sbjct 192 AAKFDGILGMAY-PRISVNNVLPVFD-NLMQQKLVDQNIFSFYLSRDPDAQPGGELMLGG 249

Query 256 IDHAKYTGSLTTLPLTSNREFTIQTNSATVGTSTIDINTGL--LLDSGTTLTYLPQSVVD 313

D Y GSL+ L +T + + + V + G ++D+GT+L P V

Sbjct 250 TDSKYYKGSLSYLNVTRKAYWQVHLDQVEVASGLTLCKEGCEAIVDTGTSLMVGPVDEVR 309

Query 314 SIANAIGGDITYNRPIGAYIWSCNRNG---KVTYNF-PQGLNIDIPYSDLAVPLYYSNGA 369

+ AIG G Y+ C + +T +G + L V

Sbjct 310 ELQKAIGAVPLIQ---GEYMIPCEKVSTLPAITLKLGGKGYKLSPEDYTLKVSQAGKTLC 366

Query 370 VAGFCALGILYGEN-FNILGDNFLRHAYVVYNLD 402

++GF + I ILGD F+ Y V++ D

Sbjct 367 LSGFMGMDIPPPSGPLWILGDVFIGRYYTVFDRD 400

>Ketopiperazine-Based Renin Inhibitors: Optimization of the C ring [Homo sapiens]

Sequence ID: 2FS4_A Length: 333

>Ketopiperazine-Based Renin Inhibitors: Optimization of the C ring [Homo sapiens]

Sequence ID: 2FS4_B Length: 333 >Ketopiperazine-based renin inhibitors: Optimization of the 'C' ring [Homo sapiens]

Sequence ID: 2G1N_A Length: 333 >Ketopiperazine-based renin inhibitors: Optimization of the 'C' ring [Homo sapiens]

Sequence ID: 2G1N_B Length: 333 >Ketopiperazine-Based Renin Inhibitors: Optimization of the 'C' Ring [Homo sapiens]

Sequence ID: 2G1O_A Length: 333 >Ketopiperazine-Based Renin Inhibitors: Optimization of the 'C' Ring [Homo sapiens]

Sequence ID: 2G1O_B Length: 333 >Ketopiperazine-Based Renin Inhibitors: Optimization of the C Ring [Homo sapiens]

Sequence ID: 2G1R_A Length: 333 >Ketopiperazine-Based Renin Inhibitors: Optimization of the C Ring [Homo sapiens]

Sequence ID: 2G1R_B Length: 333 >Ketopiperazine-Based Renin Inhibitors: Optimization of the C Ring [Homo sapiens]

Sequence ID: 2G1S_A Length: 333 >Ketopiperazine-Based Renin Inhibitors: Optimization of the C Ring [Homo sapiens]

Sequence ID: 2G1S_B Length: 333 >Ketopiperazine-Based Renin Inhibitors: Optimization of the 'C' Ring [Homo sapiens]

Sequence ID: 2G1Y_A Length: 333 >Ketopiperazine-Based Renin Inhibitors: Optimization of the 'C' Ring [Homo sapiens]

Sequence ID: 2G1Y_B Length: 333 >Ketopiperazine-Based Renin Inhibitors: Optimization of the C Ring [Homo sapiens]

Sequence ID: 2G20_A Length: 333 >Ketopiperazine-Based Renin Inhibitors: Optimization of the C Ring [Homo sapiens]

Sequence ID: 2G20_B Length: 333 >Ketopiperazine-Based Renin Inhibitors: Optimization of the 'C' Ring [Homo sapiens]

Sequence ID: 2G21_A Length: 333 >Ketopiperazine-Based Renin Inhibitors: Optimization of the 'C' Ring [Homo sapiens]

Sequence ID: 2G21_B Length: 333 >Ketopiperazine-Based Renin Inhibitors: Optimization of the 'C' Ring [Homo sapiens]

Sequence ID: 2G22_A Length: 333 >Ketopiperazine-Based Renin Inhibitors: Optimization of the 'C' Ring [Homo sapiens]

Sequence ID: 2G22_B Length: 333 >Ketopiperazine-Based Renin Inhibitors: Optimization of the 'C' Ring [Homo sapiens]

Sequence ID: 2G24_A Length: 333 >Ketopiperazine-Based Renin Inhibitors: Optimization of the 'C' Ring [Homo sapiens]

Sequence ID: 2G24_B Length: 333 >Ketopiperazine-Based Renin Inhibitors: Optimization of the 'C' Ring [Homo sapiens]

Sequence ID: 2G26_A Length: 333 >Ketopiperazine-Based Renin Inhibitors: Optimization of the 'C' Ring [Homo sapiens]

Sequence ID: 2G26_B Length: 333 >Ketopiperazine-Based Renin Inhibitors: Optimization of the 'C' Ring [Homo sapiens]

Sequence ID: 2G27_A Length: 333 >Ketopiperazine-Based Renin Inhibitors: Optimization of the 'C' Ring [Homo sapiens]

Sequence ID: 2G27_B Length: 333

Range 1: 13 to 254

Score:73.2 bits(178), Expect:4e-13,

Method:Compositional matrix adjust.,

Identities:70/258(27%), Positives:112/258(43%), Gaps:31/258(12%)

Query 96 YSANFTVGSNSQKQNVIVDTGSSDLWVVDSSANCQEKSGYSSDYCFSGGTYDPSSSSTIQ 155

Y +G+ Q V+ DTGSS++WV S+ C Y++ C +D S SS+ +

Sbjct 13 YYGEIGIGTPPQTFKVVFDTGSSNVWV--PSSKCSRL--YTA--CVYHKLFDASDSSSYK 66

Query 156 ELGKSFNIRYGDGSSSSGTWVKDTVGINGAIILNQQFGDVNSTSV-------SQGILGIG 208

G +RY G + SG +D + + G I + Q FG+V G++G+G

Sbjct 67 HNGTELTLRYSTG-TVSGFLSQDIITV-GGITVTQMFGEVTEMPALPFMLAEFDGVVGMG 124

Query 209 LDTNESTDTIYENFPI--NLKEQGFINTNAYSLYLNAPSATS----GTIIFGGIDHAKYT 262

I PI N+ QG + + +S Y N S S G I+ GG D Y

Sbjct 125 FIEQ----AIGRVTPIFDNIISQGVLKEDVFSFYYNRDSENSQSLGGQIVLGGSDPQHYE 180

Query 263 GSLTTLPLTSNREFTIQTNSATVGTSTIDINTGL--LLDSGTTLTYLPQSVVDSIANAIG 320

G+ + L + IQ +VG+ST+ G L+D+G + S ++ + A+G

Sbjct 181 GNFHYINLIKTGVWQIQMKGVSVGSSTLLCEDGCLALVDTGASYISGSTSSIEKLMEALG 240

Query 321 GDITYNRPIGAYIWSCNR 338

+ + Y+ CN

Sbjct 241 A----KKRLFDYVVKCNE 254

>Human renin in complex with remikiren [Homo sapiens]

Sequence ID: 3D91_A Length: 341

>Human renin in complex with remikiren [Homo sapiens]

Sequence ID: 3D91_B Length: 341 >Design and Preparation of Potent, Non-Peptidic, Bioavailable Renin Inhibitors [Homo sapiens]

Sequence ID: 3G6Z_A Length: 341 >Design and Preparation of Potent, Non-Peptidic, Bioavailable Renin Inhibitors [Homo sapiens]

Sequence ID: 3G6Z_B Length: 341 >Design and Preparation of Potent, Non-Peptidic, Bioavailable Renin Inhibitors [Homo sapiens]

Sequence ID: 3G70_A Length: 341 >Design and Preparation of Potent, Non-Peptidic, Bioavailable Renin Inhibitors [Homo sapiens]

Sequence ID: 3G70_B Length: 341 >New Classes of Potent and Bioavailable Human Renin Inhibitors [Homo sapiens]

Sequence ID: 3K1W_A Length: 341 >New Classes of Potent and Bioavailable Human Renin Inhibitors [Homo sapiens]

Sequence ID: 3K1W_B Length: 341 >Potent macrocyclic renin inhibitors [Homo sapiens]

Sequence ID: 3OWN_A Length: 341 >Potent macrocyclic renin inhibitors [Homo sapiens]

Sequence ID: 3OWN_B Length: 341

Range 1: 20 to 261

Score:73.2 bits(178), Expect:4e-13,

Method:Compositional matrix adjust.,

Identities:70/258(27%), Positives:112/258(43%), Gaps:31/258(12%)

Query 96 YSANFTVGSNSQKQNVIVDTGSSDLWVVDSSANCQEKSGYSSDYCFSGGTYDPSSSSTIQ 155

Y +G+ Q V+ DTGSS++WV S+ C Y++ C +D S SS+ +

Sbjct 20 YYGEIGIGTPPQTFKVVFDTGSSNVWV--PSSKCSRL--YTA--CVYHKLFDASDSSSYK 73

Query 156 ELGKSFNIRYGDGSSSSGTWVKDTVGINGAIILNQQFGDVNSTSV-------SQGILGIG 208

G +RY G + SG +D + + G I + Q FG+V G++G+G

Sbjct 74 HNGTELTLRYSTG-TVSGFLSQDIITV-GGITVTQMFGEVTEMPALPFMLAEFDGVVGMG 131

Query 209 LDTNESTDTIYENFPI--NLKEQGFINTNAYSLYLNAPSATS----GTIIFGGIDHAKYT 262

I PI N+ QG + + +S Y N S S G I+ GG D Y

Sbjct 132 FIEQ----AIGRVTPIFDNIISQGVLKEDVFSFYYNRDSENSQSLGGQIVLGGSDPQHYE 187

Query 263 GSLTTLPLTSNREFTIQTNSATVGTSTIDINTGL--LLDSGTTLTYLPQSVVDSIANAIG 320

G+ + L + IQ +VG+ST+ G L+D+G + S ++ + A+G

Sbjct 188 GNFHYINLIKTGVWQIQMKGVSVGSSTLLCEDGCLALVDTGASYISGSTSSIEKLMEALG 247

Query 321 GDITYNRPIGAYIWSCNR 338

+ + Y+ CN

Sbjct 248 A----KKRLFDYVVKCNE 261

>Human renin/PF02342674 complex [Homo sapiens]

Sequence ID: 2I4Q_A Length: 336

>Human renin/PF02342674 complex [Homo sapiens]

Sequence ID: 2I4Q_B Length: 336

Range 1: 16 to 257

Score:73.2 bits(178), Expect:4e-13,

Method:Compositional matrix adjust.,

Identities:70/258(27%), Positives:112/258(43%), Gaps:31/258(12%)

Query 96 YSANFTVGSNSQKQNVIVDTGSSDLWVVDSSANCQEKSGYSSDYCFSGGTYDPSSSSTIQ 155

Y +G+ Q V+ DTGSS++WV S+ C Y++ C +D S SS+ +

Sbjct 16 YYGEIGIGTPPQTFKVVFDTGSSNVWV--PSSKCSRL--YTA--CVYHKLFDASDSSSYK 69

Query 156 ELGKSFNIRYGDGSSSSGTWVKDTVGINGAIILNQQFGDVNSTSV-------SQGILGIG 208

G +RY G + SG +D + + G I + Q FG+V G++G+G

Sbjct 70 HNGTELTLRYSTG-TVSGFLSQDIITV-GGITVTQMFGEVTEMPALPFMLAEFDGVVGMG 127

Query 209 LDTNESTDTIYENFPI--NLKEQGFINTNAYSLYLNAPSATS----GTIIFGGIDHAKYT 262

I PI N+ QG + + +S Y N S S G I+ GG D Y

Sbjct 128 FIEQ----AIGRVTPIFDNIISQGVLKEDVFSFYYNRDSENSQSLGGQIVLGGSDPQHYE 183

Query 263 GSLTTLPLTSNREFTIQTNSATVGTSTIDINTGL--LLDSGTTLTYLPQSVVDSIANAIG 320

G+ + L + IQ +VG+ST+ G L+D+G + S ++ + A+G

Sbjct 184 GNFHYINLIKTGVWQIQMKGVSVGSSTLLCEDGCLALVDTGASYISGSTSSIEKLMEALG 243

Query 321 GDITYNRPIGAYIWSCNR 338

+ + Y+ CN

Sbjct 244 A----KKRLFDYVVKCNE 257

>CRYSTALLOGRAPHIC STUDIES ON THE BINDING MODES OF P2-P3 BUTANEDIAMIDE RENIN INHIBITORS [Homo sapiens]

Sequence ID: 1BIL_A Length: 337

>CRYSTALLOGRAPHIC STUDIES ON THE BINDING MODES OF P2-P3 BUTANEDIAMIDE RENIN INHIBITORS [Homo sapiens]

Sequence ID: 1BIL_B Length: 337 >CRYSTALLOGRAPHIC STUDIES ON THE BINDING MODES OF P2-P3 BUTANEDIAMIDE RENIN INHIBITORS [Homo sapiens]

Sequence ID: 1BIM_A Length: 337 >CRYSTALLOGRAPHIC STUDIES ON THE BINDING MODES OF P2-P3 BUTANEDIAMIDE RENIN INHIBITORS [Homo sapiens]

Sequence ID: 1BIM_B Length: 337 >HIGH RESOLUTION CRYSTAL STRUCTURES OF RECOMBINANT HUMAN RENIN IN COMPLEX WITH POLYHYDROXYMONOAMIDE INHIBITORS [Homo sapiens]

Sequence ID: 1HRN_A Length: 337 >HIGH RESOLUTION CRYSTAL STRUCTURES OF RECOMBINANT HUMAN RENIN IN COMPLEX WITH POLYHYDROXYMONOAMIDE INHIBITORS [Homo sapiens]

Sequence ID: 1HRN_B Length: 337 >Crystal structure of human renin complexed with a novel inhibitor [Homo sapiens]

Sequence ID: 3GW5_A Length: 337 >Crystal structure of human renin complexed with a novel inhibitor [Homo sapiens]

Sequence ID: 3GW5_B Length: 337 >Optimization of Orally Bioavailable Alkyl Amine Renin Inhibitors [Homo sapiens]

Sequence ID: 3KM4_A Length: 337 >Optimization of Orally Bioavailable Alkyl Amine Renin Inhibitors [Homo sapiens]

Sequence ID: 3KM4_B Length: 337 >Discovery of TAK-272: A Novel, Potent and Orally Active Renin In-hibitor [Homo sapiens]

Sequence ID: 5KOQ_A Length: 337 >Discovery of TAK-272: A Novel, Potent and Orally Active Renin In-hibitor [Homo sapiens]

Sequence ID: 5KOQ_B Length: 337 >Discovery of TAK-272: A Novel, Potent and Orally Active Renin In-hibitor [Homo sapiens]

Sequence ID: 5KOS_A Length: 337 >Discovery of TAK-272: A Novel, Potent and Orally Active Renin In-hibitor [Homo sapiens]

Sequence ID: 5KOS_B Length: 337 >Discovery of TAK-272: A Novel, Potent and Orally Active Renin In-hibitor [Homo sapiens]

Sequence ID: 5KOT_A Length: 337 >Discovery of TAK-272: A Novel, Potent and Orally Active Renin In-hibitor [Homo sapiens]

Sequence ID: 5KOT_B Length: 337 >Optimization of 3,5-Disubstitued Piperidine: Discovery of Non-Peptide mimetics as an Orally Active Renin Inhibitor [Homo sapiens]

Sequence ID: 5TMG_A Length: 337 >Optimization of 3,5-Disubstitued Piperidine: Discovery of Non-Peptide mimetics as an Orally Active Renin Inhibitor [Homo sapiens]

Sequence ID: 5TMG_B Length: 337 >Optimization of 3,5-Disubstitued Piperidine: Discovery of Non-Peptide mimetics as an Orally Active Renin Inhibitor [Homo sapiens]

Sequence ID: 5TMK_A Length: 337 >Optimization of 3,5-Disubstitued Piperidine: Discovery of Non-Peptide mimetics as an Orally Active Renin Inhibitor [Homo sapiens]

Sequence ID: 5TMK_B Length: 337 >Crystal Structure of Human Renin in Complex with a biphenylpipderidinylcarbinol [Homo sapiens]

Sequence ID: 5V8V_A Length: 337 >Crystal Structure of Human Renin in Complex with a biphenylpipderidinylcarbinol [Homo sapiens]

Sequence ID: 5V8V_B Length: 337 >Crystal Structure of Human Renin in Complex with a biphenylpipderidinylcarbinol [Homo sapiens]

Sequence ID: 5VPM_A Length: 337 >Crystal Structure of Human Renin in Complex with a biphenylpipderidinylcarbinol [Homo sapiens]

Sequence ID: 5VPM_B Length: 337 >Crystal Structure of Human Renin in Complex with a biphenylpipderidinylcarbinol [Homo sapiens]

Sequence ID: 5VRP_A Length: 337 >Crystal Structure of Human Renin in Complex with a biphenylpipderidinylcarbinol [Homo sapiens]

Sequence ID: 5VRP_B Length: 337

Range 1: 17 to 258

Score:73.2 bits(178), Expect:5e-13,

Method:Compositional matrix adjust.,

Identities:70/258(27%), Positives:112/258(43%), Gaps:31/258(12%)

Query 96 YSANFTVGSNSQKQNVIVDTGSSDLWVVDSSANCQEKSGYSSDYCFSGGTYDPSSSSTIQ 155

Y +G+ Q V+ DTGSS++WV S+ C Y++ C +D S SS+ +

Sbjct 17 YYGEIGIGTPPQTFKVVFDTGSSNVWV--PSSKCSRL--YTA--CVYHKLFDASDSSSYK 70

Query 156 ELGKSFNIRYGDGSSSSGTWVKDTVGINGAIILNQQFGDVNSTSV-------SQGILGIG 208

G +RY G + SG +D + + G I + Q FG+V G++G+G

Sbjct 71 HNGTELTLRYSTG-TVSGFLSQDIITV-GGITVTQMFGEVTEMPALPFMLAEFDGVVGMG 128

Query 209 LDTNESTDTIYENFPI--NLKEQGFINTNAYSLYLNAPSATS----GTIIFGGIDHAKYT 262

I PI N+ QG + + +S Y N S S G I+ GG D Y

Sbjct 129 FIEQ----AIGRVTPIFDNIISQGVLKEDVFSFYYNRDSENSQSLGGQIVLGGSDPQHYE 184

Query 263 GSLTTLPLTSNREFTIQTNSATVGTSTIDINTGL--LLDSGTTLTYLPQSVVDSIANAIG 320

G+ + L + IQ +VG+ST+ G L+D+G + S ++ + A+G

Sbjct 185 GNFHYINLIKTGVWQIQMKGVSVGSSTLLCEDGCLALVDTGASYISGSTSSIEKLMEALG 244

Query 321 GDITYNRPIGAYIWSCNR 338

+ + Y+ CN

Sbjct 245 A----KKRLFDYVVKCNE 258

>Structure-based design of a new series of N-piperidin-3-ylpyrimidine-5-carboxamides as renin inhibitors [Homo sapiens]

Sequence ID: 5SXN_A Length: 339

>Structure-based design of a new series of N-piperidin-3-ylpyrimidine-5-carboxamides as renin inhibitors [Homo sapiens]

Sequence ID: 5SXN_B Length: 339 >Structure-based design of a new series of N-piperidin-3-ylpyrimidine-5-carboxamides as renin inhibitors [Homo sapiens]

Sequence ID: 5SY3_A Length: 339 >Structure-based design of a new series of N-piperidin-3-ylpyrimidine-5-carboxamides as renin inhibitors [Homo sapiens]

Sequence ID: 5SY3_B Length: 339 >Structure-based design of a new series of N-piperidin-3-ylpyrimidine-5-carboxamides as renin inhibitors [Homo sapiens]

Sequence ID: 5SZ9_A Length: 339 >Structure-based design of a new series of N-piperidin-3-ylpyrimidine-5-carboxamides as renin inhibitors [Homo sapiens]

Sequence ID: 5SZ9_B Length: 339 >Novel Approach of Fragment-Based Lead Discovery applied to Renin Inhibitors [Homo sapiens]

Sequence ID: 5T4S_A Length: 339 >Novel Approach of Fragment-Based Lead Discovery applied to Renin Inhibitors [Homo sapiens]

Sequence ID: 5T4S_B Length: 339

Range 1: 19 to 260

Score:73.2 bits(178), Expect:5e-13,

Method:Compositional matrix adjust.,

Identities:70/258(27%), Positives:112/258(43%), Gaps:31/258(12%)

Query 96 YSANFTVGSNSQKQNVIVDTGSSDLWVVDSSANCQEKSGYSSDYCFSGGTYDPSSSSTIQ 155

Y +G+ Q V+ DTGSS++WV S+ C Y++ C +D S SS+ +

Sbjct 19 YYGEIGIGTPPQTFKVVFDTGSSNVWV--PSSKCSRL--YTA--CVYHKLFDASDSSSYK 72

Query 156 ELGKSFNIRYGDGSSSSGTWVKDTVGINGAIILNQQFGDVNSTSV-------SQGILGIG 208

G +RY G + SG +D + + G I + Q FG+V G++G+G

Sbjct 73 HNGTELTLRYSTG-TVSGFLSQDIITV-GGITVTQMFGEVTEMPALPFMLAEFDGVVGMG 130

Query 209 LDTNESTDTIYENFPI--NLKEQGFINTNAYSLYLNAPSATS----GTIIFGGIDHAKYT 262

I PI N+ QG + + +S Y N S S G I+ GG D Y

Sbjct 131 FIEQ----AIGRVTPIFDNIISQGVLKEDVFSFYYNRDSENSQSLGGQIVLGGSDPQHYE 186

Query 263 GSLTTLPLTSNREFTIQTNSATVGTSTIDINTGL--LLDSGTTLTYLPQSVVDSIANAIG 320

G+ + L + IQ +VG+ST+ G L+D+G + S ++ + A+G

Sbjct 187 GNFHYINLIKTGVWQIQMKGVSVGSSTLLCEDGCLALVDTGASYISGSTSSIEKLMEALG 246

Query 321 GDITYNRPIGAYIWSCNR 338

+ + Y+ CN

Sbjct 247 A----KKRLFDYVVKCNE 260

>X-RAY ANALYSES OF PEPTIDE INHIBITOR COMPLEXES DEFINE THE STRUCTURAL BASIS OF SPECIFICITY FOR HUMAN AND MOUSE RENINS [Homo sapiens]

Sequence ID: 1BBS_A Length: 340

>X-RAY ANALYSES OF PEPTIDE INHIBITOR COMPLEXES DEFINE THE STRUCTURAL BASIS OF SPECIFICITY FOR HUMAN AND MOUSE RENINS [Homo sapiens]

Sequence ID: 1BBS_B Length: 340 >THE CRYSTAL STRUCTURE OF RECOMBINANT GLYCOSYLATED HUMAN RENIN ALONE AND IN COMPLEX WITH A TRANSITION STATE ANALOG INHIBITOR [Homo sapiens]

Sequence ID: 1RNE_A Length: 340 >crystal structure of Renin-PF00074777 complex [Homo sapiens]

Sequence ID: 2BKS_A Length: 340 >crystal structure of Renin-PF00074777 complex [Homo sapiens]

Sequence ID: 2BKS_B Length: 340 >crystal structure of renin-pf00257567 complex [Homo sapiens]

Sequence ID: 2BKT_A Length: 340 >crystal structure of renin-pf00257567 complex [Homo sapiens]

Sequence ID: 2BKT_B Length: 340 >Crystal Structure of Human Renin Complexed with Inhibitor [Homo sapiens]

Sequence ID: 2IKO_A Length: 340 >Crystal Structure of Human Renin Complexed with Inhibitor [Homo sapiens]

Sequence ID: 2IKO_B Length: 340 >Crystal Structure of Human Renin Complexed with Inhibitors [Homo sapiens]

Sequence ID: 2IKU_A Length: 340 >Crystal Structure of Human Renin Complexed with Inhibitors [Homo sapiens]

Sequence ID: 2IKU_B Length: 340 >Crystal Structure of Human Renin Complexed with Inhibitor [Homo sapiens]

Sequence ID: 2IL2_A Length: 340 >Crystal Structure of Human Renin Complexed with Inhibitor [Homo sapiens]

Sequence ID: 2IL2_B Length: 340 >STRUCTURE OF RECOMBINANT HUMAN RENIN, A TARGET FOR CARDIOVASCULAR-ACTIVE DRUGS, AT 2.5 ANGSTROMS RESOLUTION [Homo sapiens]

Sequence ID: 2REN_A Length: 340 >Crystal Structure of Renin with Inhibitor 10 (Aliskiren) [Homo sapiens]

Sequence ID: 2V0Z_C Length: 340 >Crystal Structure of Renin with Inhibitor 10 (Aliskiren) [Homo sapiens]

Sequence ID: 2V0Z_O Length: 340 >Crystal Structure of Renin with Inhibitor 9 [Homo sapiens]

Sequence ID: 2V10_C Length: 340 >Crystal Structure of Renin with Inhibitor 9 [Homo sapiens]

Sequence ID: 2V10_O Length: 340 >Crystal Structure of Renin with Inhibitor 6 [Homo sapiens]

Sequence ID: 2V11_C Length: 340 >Crystal Structure of Renin with Inhibitor 6 [Homo sapiens]

Sequence ID: 2V11_O Length: 340 >Crystal Structure of Renin with Inhibitor 8 [Homo sapiens]

Sequence ID: 2V12_C Length: 340 >Crystal Structure of Renin with Inhibitor 8 [Homo sapiens]

Sequence ID: 2V12_O Length: 340 >Crystal Structure of Renin with Inhibitor 7 [Homo sapiens]

Sequence ID: 2V13_A Length: 340 >Crystal Structure of Renin with Inhibitor 3 [Homo sapiens]

Sequence ID: 2V16_C Length: 340 >Crystal Structure of Renin with Inhibitor 3 [Homo sapiens]

Sequence ID: 2V16_O Length: 340 >Design and Preparation of Potent, Non-Peptidic, Bioavailable Renin Inhibitors [Homo sapiens]

Sequence ID: 3G72_A Length: 340 >Design and Preparation of Potent, Non-Peptidic, Bioavailable Renin Inhibitors [Homo sapiens]

Sequence ID: 3G72_B Length: 340 >Crystal Structure Analysis of Renin-indole-piperazin inhibitor complexes [Homo sapiens]

Sequence ID: 3OOT_A Length: 340 >Crystal Structure Analysis of Renin-indole-piperazin inhibitor complexes [Homo sapiens]

Sequence ID: 3OOT_B Length: 340 >Crystal Structure Analysis of Renin-indole-piperazine inhibitor complexes [Homo sapiens]

Sequence ID: 3OQF_A Length: 340 >Crystal Structure Analysis of Renin-indole-piperazine inhibitor complexes [Homo sapiens]

Sequence ID: 3OQF_B Length: 340 >Crystal Structure Analysis of Renin-indole-piperazin inhibitor complexes [Homo sapiens]

Sequence ID: 3OQK_A Length: 340 >Crystal Structure Analysis of Renin-indole-piperazin inhibitor complexes [Homo sapiens]

Sequence ID: 3OQK_B Length: 340 >Alkyl Amine Renin Inhibitors: Filling S1 from S3 [Homo sapiens]

Sequence ID: 3Q3T_A Length: 340 >Alkyl Amine Renin Inhibitors: Filling S1 from S3 [Homo sapiens]

Sequence ID: 3Q3T_B Length: 340 >Clinically Useful Alkyl Amine Renin Inhibitors [Homo sapiens]

Sequence ID: 3Q4B_A Length: 340 >Clinically Useful Alkyl Amine Renin Inhibitors [Homo sapiens]

Sequence ID: 3Q4B_B Length: 340 >Clinically Useful Alkyl Amine Renin Inhibitors [Homo sapiens]

Sequence ID: 3Q5H_A Length: 340 >Clinically Useful Alkyl Amine Renin Inhibitors [Homo sapiens]

Sequence ID: 3Q5H_B Length: 340 >Structure-Based Optimization of Potent 4- and 6-Azaindole-3-Carboxamides as Renin Inhibitors [Homo sapiens]

Sequence ID: 3SFC_A Length: 340 >Structure-Based Optimization of Potent 4- and 6-Azaindole-3-Carboxamides as Renin Inhibitors [Homo sapiens]

Sequence ID: 3SFC_B Length: 340 >Human renin in complex with compound 8 [Homo sapiens]

Sequence ID: 3VSW_A Length: 340 >Human renin in complex with compound 8 [Homo sapiens]

Sequence ID: 3VSW_B Length: 340 >Human renin in complex with compound 18 [Homo sapiens]

Sequence ID: 3VSX_A Length: 340 >Human renin in complex with compound 18 [Homo sapiens]

Sequence ID: 3VSX_B Length: 340 >Human renin in complex with compound 5 [Homo sapiens]

Sequence ID: 3VUC_A Length: 340 >Human renin in complex with compound 5 [Homo sapiens]

Sequence ID: 3VUC_B Length: 340 >Human renin in complex with inhibitor 6 [Homo sapiens]

Sequence ID: 3VYD_A Length: 340 >Human renin in complex with inhibitor 6 [Homo sapiens]

Sequence ID: 3VYD_B Length: 340 >Human renin in complex with inhibitor 7 [Homo sapiens]

Sequence ID: 3VYE_A Length: 340 >Human renin in complex with inhibitor 7 [Homo sapiens]

Sequence ID: 3VYE_B Length: 340 >Human renin in complex with inhibitor 9 [Homo sapiens]

Sequence ID: 3VYF_A Length: 340 >Human renin in complex with inhibitor 9 [Homo sapiens]

Sequence ID: 3VYF_B Length: 340 >Crystal structure of renin in complex with NVP-AMQ838 (compound 5) [Homo sapiens]

Sequence ID: 4GJ5_A Length: 340 >Crystal structure of renin in complex with NVP-AMQ838 (compound 5) [Homo sapiens]

Sequence ID: 4GJ5_B Length: 340 >Crystal structure of renin in complex with NVP-AYZ832 (compound 6a) [Homo sapiens]

Sequence ID: 4GJ6_A Length: 340 >Crystal structure of renin in complex with NVP-AYZ832 (compound 6a) [Homo sapiens]

Sequence ID: 4GJ6_B Length: 340 >Crystal structure of renin in complex with NVP-BCA079 (compound 12a) [Homo sapiens]

Sequence ID: 4GJ7_A Length: 340 >Crystal structure of renin in complex with NVP-BCA079 (compound 12a) [Homo sapiens]

Sequence ID: 4GJ7_B Length: 340 >Crystal structure of renin in complex with PKF909-724 (compound 3) [Homo sapiens]

Sequence ID: 4GJ8_A Length: 340 >Crystal structure of renin in complex with PKF909-724 (compound 3) [Homo sapiens]

Sequence ID: 4GJ8_B Length: 340 >Crystal structure of renin in complex with GP055321 (compound 4) [Homo sapiens]

Sequence ID: 4GJ9_A Length: 340 >Crystal structure of renin in complex with GP055321 (compound 4) [Homo sapiens]

Sequence ID: 4GJ9_B Length: 340 >Crystal structure of renin in complex with NVP-AYL747 (compound 5) [Homo sapiens]

Sequence ID: 4GJA_A Length: 340 >Crystal structure of renin in complex with NVP-AYL747 (compound 5) [Homo sapiens]

Sequence ID: 4GJA_B Length: 340 >Crystal structure of renin in complex with NVP-BBV031 (compound 6) [Homo sapiens]

Sequence ID: 4GJB_A Length: 340 >Crystal structure of renin in complex with NVP-BBV031 (compound 6) [Homo sapiens]

Sequence ID: 4GJB_B Length: 340 >Crystal structure of renin in complex with NVP-BCH965 (compound 9) [Homo sapiens]

Sequence ID: 4GJC_A Length: 340 >Crystal structure of renin in complex with NVP-BCH965 (compound 9) [Homo sapiens]

Sequence ID: 4GJC_B Length: 340 >Crystal structure of renin in complex with NVP-BGQ311 (compound 12) [Homo sapiens]

Sequence ID: 4GJD_A Length: 340 >Crystal structure of renin in complex with NVP-BGQ311 (compound 12) [Homo sapiens]

Sequence ID: 4GJD_B Length: 340 >Crystal structure of renin in complex with compound4 [Homo sapiens]

Sequence ID: 4PYV_A Length: 340 >Crystal structure of renin in complex with compound4 [Homo sapiens]

Sequence ID: 4PYV_B Length: 340 >Structure-based design of 4-hydroxy-3,5-substituted piperidines as direct renin inhibitors [Homo sapiens]

Sequence ID: 4Q1N_A Length: 340 >Structure-based design of 4-hydroxy-3,5-substituted piperidines as direct renin inhibitors [Homo sapiens]

Sequence ID: 4Q1N_B Length: 340 >RENIN IN COMPLEXED WITH 4-methoxy-3-(3-methoxypropoxy)-N-{[(3S,4S)-4-{[(4-methylphenyl)sulfonyl]amino}pyrrolidin-3-yl]methyl}-N-(propan-2-yl)benzamide INHIBITOR [Homo sapiens]

Sequence ID: 4RYC_A Length: 340 >RENIN IN COMPLEXED WITH 4-methoxy-3-(3-methoxypropoxy)-N-{[(3S,4S)-4-{[(4-methylphenyl)sulfonyl]amino}pyrrolidin-3-yl]methyl}-N-(propan-2-yl)benzamide INHIBITOR [Homo sapiens]

Sequence ID: 4RYC_B Length: 340 >RENIN IN COMPLEXED WITH N-({(3S,4S)-4-[(benzylsulfonyl)amino]pyrrolidin-3-yl}methyl)-4-methoxy-3-(3-methoxypropoxy)-N-(propan-2-yl)benzamide INHIBITOR [Homo sapiens]

Sequence ID: 4RYG_A Length: 340 >RENIN IN COMPLEXED WITH N-({(3S,4S)-4-[(benzylsulfonyl)amino]pyrrolidin-3-yl}methyl)-4-methoxy-3-(3-methoxypropoxy)-N-(propan-2-yl)benzamide INHIBITOR [Homo sapiens]

Sequence ID: 4RYG_B Length: 340 >RENIN IN COMPLEXED WITH (3S,4S)-4-({[4-methoxy-3-(3-methoxypropoxy)benzoyl](propan-2-yl)amino}methyl)pyrrolidin-3-yl benzylcarbamate INHIBITOR [Homo sapiens]

Sequence ID: 4RZ1_A Length: 340 >RENIN IN COMPLEXED WITH (3S,4S)-4-({[4-methoxy-3-(3-methoxypropoxy)benzoyl](propan-2-yl)amino}methyl)pyrrolidin-3-yl benzylcarbamate INHIBITOR [Homo sapiens]

Sequence ID: 4RZ1_B Length: 340 >Renin in complex with (S)-1-(3-fluoro-5-(((S)-1-phenylethyl)carbamoyl)benzyl)-4-isopropyl-4-methyl-6-oxotetrahydropyrimidin-2(1H)-iminium [Homo sapiens]

Sequence ID: 4S1G_A Length: 340 >Renin in complex with (S)-1-(3-fluoro-5-(((S)-1-phenylethyl)carbamoyl)benzyl)-4-isopropyl-4-methyl-6-oxotetrahydropyrimidin-2(1H)-iminium [Homo sapiens]

Sequence ID: 4S1G_B Length: 340 >Renin in complex with (S)-1-(3-(benzylcarbamoyl)benzyl)-4-isopropyl-4-methyl-6-oxotetrahydropyrimidin-2(1H)-iminium [Homo sapiens]

Sequence ID: 4XX3_A Length: 340 >Renin in complex with (S)-1-(3-(benzylcarbamoyl)benzyl)-4-isopropyl-4-methyl-6-oxotetrahydropyrimidin-2(1H)-iminium [Homo sapiens]

Sequence ID: 4XX3_B Length: 340 >Renin in complex with (4S)-4-isopropyl-4-methyl-6-oxo-1-(3-(2-oxo-4-phenylpyrrolidin-1-yl)benzyl)tetrahydropyrimidin-2(1H)-iminium [Homo sapiens]

Sequence ID: 4XX4_A Length: 340 >Renin in complex with (4S)-4-isopropyl-4-methyl-6-oxo-1-(3-(2-oxo-4-phenylpyrrolidin-1-yl)benzyl)tetrahydropyrimidin-2(1H)-iminium [Homo sapiens]

Sequence ID: 4XX4_B Length: 340 >Structure-based design of a new series of N-piperidin-3-ylpyrimidine-5-carboxamides as renin inhibitors [Homo sapiens]

Sequence ID: 5SY2_A Length: 340 >Structure-based design of a new series of N-piperidin-3-ylpyrimidine-5-carboxamides as renin inhibitors [Homo sapiens]

Sequence ID: 5SY2_B Length: 340

Range 1: 20 to 261

Score:73.2 bits(178), Expect:5e-13,

Method:Compositional matrix adjust.,

Identities:70/258(27%), Positives:112/258(43%), Gaps:31/258(12%)

Query 96 YSANFTVGSNSQKQNVIVDTGSSDLWVVDSSANCQEKSGYSSDYCFSGGTYDPSSSSTIQ 155

Y +G+ Q V+ DTGSS++WV S+ C Y++ C +D S SS+ +

Sbjct 20 YYGEIGIGTPPQTFKVVFDTGSSNVWV--PSSKCSRL--YTA--CVYHKLFDASDSSSYK 73

Query 156 ELGKSFNIRYGDGSSSSGTWVKDTVGINGAIILNQQFGDVNSTSV-------SQGILGIG 208

G +RY G + SG +D + + G I + Q FG+V G++G+G

Sbjct 74 HNGTELTLRYSTG-TVSGFLSQDIITV-GGITVTQMFGEVTEMPALPFMLAEFDGVVGMG 131

Query 209 LDTNESTDTIYENFPI--NLKEQGFINTNAYSLYLNAPSATS----GTIIFGGIDHAKYT 262

I PI N+ QG + + +S Y N S S G I+ GG D Y

Sbjct 132 FIEQ----AIGRVTPIFDNIISQGVLKEDVFSFYYNRDSENSQSLGGQIVLGGSDPQHYE 187

Query 263 GSLTTLPLTSNREFTIQTNSATVGTSTIDINTGL--LLDSGTTLTYLPQSVVDSIANAIG 320

G+ + L + IQ +VG+ST+ G L+D+G + S ++ + A+G

Sbjct 188 GNFHYINLIKTGVWQIQMKGVSVGSSTLLCEDGCLALVDTGASYISGSTSSIEKLMEALG 247

Query 321 GDITYNRPIGAYIWSCNR 338

+ + Y+ CN

Sbjct 248 A----KKRLFDYVVKCNE 261

>Crystal structure of human prorenin [Homo sapiens]

Sequence ID: 3VCM_A Length: 335

>Crystal structure of human prorenin [Homo sapiens]

Sequence ID: 3VCM_B Length: 335

Range 1: 20 to 256

Score:72.8 bits(177), Expect:5e-13,

Method:Compositional matrix adjust.,

Identities:69/254(27%), Positives:111/254(43%), Gaps:28/254(11%)

Query 96 YSANFTVGSNSQKQNVIVDTGSSDLWVVDSSANCQEKSGYSSDYCFSGGTYDPSSSSTIQ 155

Y +G+ Q V+ DTGSS++WV S+ C Y++ C +D S SS+ +

Sbjct 20 YYGEIGIGTPPQTFKVVFDTGSSNVWV--PSSKCSRL--YTA--CVYHKLFDASDSSSYK 73

Query 156 ELGKSFNIRYGDGSSSSGTWVKDTVGINGAIILNQQFGDVNSTSV-------SQGILGIG 208

G +RY G + SG +D + + G I + Q FG+V G++G+G

Sbjct 74 HNGTELTLRYSTG-TVSGFLSQDIITV-GGITVTQMFGEVTEMPALPFMLAEFDGVVGMG 131

Query 209 LDTNESTDTIYENFPI--NLKEQGFINTNAYSLYLNAPSATSGTIIFGGIDHAKYTGSLT 266

I PI N+ QG + + +S Y N S G I+ GG D Y G+

Sbjct 132 FIEQ----AIGRVTPIFDNIISQGVLKEDVFSFYYNRDSL-GGQIVLGGSDPQHYEGNFH 186

Query 267 TLPLTSNREFTIQTNSATVGTSTIDINTGL--LLDSGTTLTYLPQSVVDSIANAIGGDIT 324

+ L + IQ +VG+ST+ G L+D+G + S ++ + A+G

Sbjct 187 YINLIKTGVWQIQMKGVSVGSSTLLCEDGCLALVDTGASYISGSTSSIEKLMEALGA--- 243

Query 325 YNRPIGAYIWSCNR 338

+ + Y+ CN

Sbjct 244 -KKRLFDYVVKCNE 256

>renin [Homo sapiens]

Sequence ID: AAA60364.1 Length: 403

Range 1: 86 to 324

Score:73.2 bits(178), Expect:6e-13,

Method:Compositional matrix adjust.,

Identities:70/255(27%), Positives:115/255(45%), Gaps:28/255(10%)

Query 96 YSANFTVGSNSQKQNVIVDTGSSDLWVVDSSANCQEKSGYSSDYCFSGGTYDPSSSSTIQ 155

Y +G+ Q V+ DTGSS++WV S+ C Y++ C +D S SS+ +

Sbjct 86 YYGEIGIGTPPQTFKVVFDTGSSNVWV--PSSKCSRL--YTA--CVYHKLFDASDSSSYK 139

Query 156 ELGKSFNIRYGDGSSSSGTWVKDTVGINGAIILNQQFGDVNSTS-----VSQ--GILGIG 208

G +RY G + SG +D + + G I + Q FG+V ++Q G++G+G

Sbjct 140 HNGTELTLRYSTG-TVSGFLSQDIITV-GGITVTQMFGEVTEMPALPFMLAQFDGVVGMG 197

Query 209 LDTNESTDTIYENFPI--NLKEQGFINTNAYSLYLNAPSAT-SGTIIFGGIDHAKYTGSL 265

I PI N+ QG + + +S Y N S + G I+ GG D Y G+

Sbjct 198 FIEQ----AIGRVTPIFDNIISQGVLKEDVFSFYYNRNSQSLGGQIVLGGSDPQHYEGNF 253

Query 266 TTLPLTSNREFTIQTNSATVGTSTIDINTGL--LLDSGTTLTYLPQSVVDSIANAIGGDI 323

+ L + IQ +VG+ST+ G L+D+G + S ++ + A+G

Sbjct 254 HYINLIKTGVWQIQMKGVSVGSSTLLCEDGCLALVDTGASYISGSTSCIEKLMEALGA-- 311

Query 324 TYNRPIGAYIWSCNR 338

+ + Y+ CN

Sbjct 312 --KKRLFDYVVKCNE 324

>Crystal structure of human angiotensinogen complexed with renin [Homo sapiens]

Sequence ID: 2X0B_A Length: 383

>Crystal structure of human angiotensinogen complexed with renin [Homo sapiens]

Sequence ID: 2X0B_C Length: 383 >Crystal structure of human angiotensinogen complexed with renin [Homo sapiens]

Sequence ID: 2X0B_E Length: 383 >Crystal structure of human angiotensinogen complexed with renin [Homo sapiens]

Sequence ID: 2X0B_G Length: 383 >Crystal structure at 2.6A of human prorenin [Homo sapiens]

Sequence ID: 4AMT_A Length: 383

Range 1: 63 to 304

Score:72.8 bits(177), Expect:7e-13,

Method:Compositional matrix adjust.,

Identities:70/258(27%), Positives:112/258(43%), Gaps:31/258(12%)

Query 96 YSANFTVGSNSQKQNVIVDTGSSDLWVVDSSANCQEKSGYSSDYCFSGGTYDPSSSSTIQ 155

Y +G+ Q V+ DTGSS++WV S+ C Y++ C +D S SS+ +

Sbjct 63 YYGEIGIGTPPQTFKVVFDTGSSNVWV--PSSKCSRL--YTA--CVYHKLFDASDSSSYK 116

Query 156 ELGKSFNIRYGDGSSSSGTWVKDTVGINGAIILNQQFGDVNSTSVS-------QGILGIG 208

G +RY G + SG +D + + G I + Q FG+V G++G+G

Sbjct 117 HNGTELTLRYSTG-TVSGFLSQDIITV-GGITVTQMFGEVTEMPALPFMLAEFDGVVGMG 174

Query 209 LDTNESTDTIYENFPI--NLKEQGFINTNAYSLYLNAPSATS----GTIIFGGIDHAKYT 262

I PI N+ QG + + +S Y N S S G I+ GG D Y

Sbjct 175 FIEQ----AIGRVTPIFDNIISQGVLKEDVFSFYYNRDSENSQSLGGQIVLGGSDPQHYE 230

Query 263 GSLTTLPLTSNREFTIQTNSATVGTSTIDINTGL--LLDSGTTLTYLPQSVVDSIANAIG 320

G+ + L + IQ +VG+ST+ G L+D+G + S ++ + A+G

Sbjct 231 GNFHYINLIKTGVWQIQMKGVSVGSSTLLCEDGCLALVDTGASYISGSTSSIEKLMEALG 290

Query 321 GDITYNRPIGAYIWSCNR 338

+ + Y+ CN

Sbjct 291 A----KKRLFDYVVKCNE 304

>Crystal structure of the complex of human angiotensinogen and renin at 2.55 Angstrom [Homo sapiens]

Sequence ID: 6I3F_B Length: 340

Range 1: 20 to 261

Score:72.4 bits(176), Expect:7e-13,

Method:Compositional matrix adjust.,

Identities:70/258(27%), Positives:111/258(43%), Gaps:31/258(12%)

Query 96 YSANFTVGSNSQKQNVIVDTGSSDLWVVDSSANCQEKSGYSSDYCFSGGTYDPSSSSTIQ 155

Y +G+ Q V+ DTGSS++WV S+ C Y++ C +D S SS+ +

Sbjct 20 YYGEIGIGTPPQTFKVVFDTGSSNVWV--PSSKCSRL--YTA--CVYHKLFDASDSSSYK 73

Query 156 ELGKSFNIRYGDGSSSSGTWVKDTVGINGAIILNQQFGDVNSTSV-------SQGILGIG 208

G +RY G + SG +D + + G I + Q FG+V G++G+G

Sbjct 74 HNGTELTLRYSTG-TVSGFLSQDIITV-GGITVTQMFGEVTEMPALPFMLAEFDGVVGMG 131

Query 209 LDTNESTDTIYENFPI--NLKEQGFINTNAYSLYLNAPSATS----GTIIFGGIDHAKYT 262

I PI N+ QG + + +S Y N S S G I+ GG D Y

Sbjct 132 FIEQ----AIGRVTPIFDNIISQGVLKEDVFSFYYNRDSENSQSLGGQIVLGGSDPQHYE 187

Query 263 GSLTTLPLTSNREFTIQTNSATVGTSTIDINTGLLLDSGTTLTYLPQSV--VDSIANAIG 320

G+ + L + IQ +VG+ST+ G L T +Y+ S ++ + A+G

Sbjct 188 GNFHYINLIKTGVWQIQMKGVSVGSSTLLCEDGCLALVATGASYISGSTSSIEKLMEALG 247

Query 321 GDITYNRPIGAYIWSCNR 338

+ + Y+ CN

Sbjct 248 A----KKRLFDYVVKCNE 261

>renin preproprotein [Homo sapiens]

Sequence ID: NP_000528.1 Length: 406

>RecName: Full=Renin; AltName: Full=Angiotensinogenase; Flags: Precursor [Homo sapiens]

Sequence ID: P00797.1 Length: 406 >renin [Homo sapiens]

Sequence ID: AAA60363.1 Length: 406 >renin [Homo sapiens]

Sequence ID: AAD03461.1 Length: 406

Range 1: 86 to 327

Score:72.8 bits(177), Expect:1e-12,

Method:Compositional matrix adjust.,

Identities:70/258(27%), Positives:112/258(43%), Gaps:31/258(12%)

Query 96 YSANFTVGSNSQKQNVIVDTGSSDLWVVDSSANCQEKSGYSSDYCFSGGTYDPSSSSTIQ 155

Y +G+ Q V+ DTGSS++WV S+ C Y++ C +D S SS+ +

Sbjct 86 YYGEIGIGTPPQTFKVVFDTGSSNVWV--PSSKCSRL--YTA--CVYHKLFDASDSSSYK 139

Query 156 ELGKSFNIRYGDGSSSSGTWVKDTVGINGAIILNQQFGDVNSTSV-------SQGILGIG 208

G +RY G + SG +D + + G I + Q FG+V G++G+G

Sbjct 140 HNGTELTLRYSTG-TVSGFLSQDIITV-GGITVTQMFGEVTEMPALPFMLAEFDGVVGMG 197

Query 209 LDTNESTDTIYENFPI--NLKEQGFINTNAYSLYLNAPSATS----GTIIFGGIDHAKYT 262

I PI N+ QG + + +S Y N S S G I+ GG D Y

Sbjct 198 FIEQ----AIGRVTPIFDNIISQGVLKEDVFSFYYNRDSENSQSLGGQIVLGGSDPQHYE 253

Query 263 GSLTTLPLTSNREFTIQTNSATVGTSTIDINTGL--LLDSGTTLTYLPQSVVDSIANAIG 320

G+ + L + IQ +VG+ST+ G L+D+G + S ++ + A+G

Sbjct 254 GNFHYINLIKTGVWQIQMKGVSVGSSTLLCEDGCLALVDTGASYISGSTSSIEKLMEALG 313

Query 321 GDITYNRPIGAYIWSCNR 338

+ + Y+ CN

Sbjct 314 A----KKRLFDYVVKCNE 327

>renin [Homo sapiens]

Sequence ID: AAR03502.1 Length: 403

>renin [Homo sapiens]

Sequence ID: EAW91505.1 Length: 403

Range 1: 86 to 324

Score:72.4 bits(176), Expect:1e-12,

Method:Compositional matrix adjust.,

Identities:69/255(27%), Positives:112/255(43%), Gaps:28/255(10%)

Query 96 YSANFTVGSNSQKQNVIVDTGSSDLWVVDSSANCQEKSGYSSDYCFSGGTYDPSSSSTIQ 155

Y +G+ Q V+ DTGSS++WV S+ C Y++ C +D S SS+ +

Sbjct 86 YYGEIGIGTPPQTFKVVFDTGSSNVWV--PSSKCSRL--YTA--CVYHKLFDASDSSSYK 139

Query 156 ELGKSFNIRYGDGSSSSGTWVKDTVGINGAIILNQQFGDVNSTSV-------SQGILGIG 208

G +RY G + SG +D + + G I + Q FG+V G++G+G

Sbjct 140 HNGTELTLRYSTG-TVSGFLSQDIITV-GGITVTQMFGEVTEMPALPFMLAEFDGVVGMG 197

Query 209 LDTNESTDTIYENFPI--NLKEQGFINTNAYSLYLNAPSAT-SGTIIFGGIDHAKYTGSL 265

I PI N+ QG + + +S Y N S + G I+ GG D Y G+

Sbjct 198 FIEQ----AIGRVTPIFDNIISQGVLKEDVFSFYYNRNSQSLGGQIVLGGSDPQHYEGNF 253

Query 266 TTLPLTSNREFTIQTNSATVGTSTIDINTGL--LLDSGTTLTYLPQSVVDSIANAIGGDI 323

+ L + IQ +VG+ST+ G L+D+G + S ++ + A+G

Sbjct 254 HYINLIKTGVWQIQMKGVSVGSSTLLCEDGCLALVDTGASYISGSTSSIEKLMEALGA-- 311

Query 324 TYNRPIGAYIWSCNR 338

+ + Y+ CN

Sbjct 312 --KKRLFDYVVKCNE 324

>unnamed protein product [Homo sapiens]

Sequence ID: BAG62845.1 Length: 282

Range 1: 74 to 256

Score:60.8 bits(146), Expect:3e-09,

Method:Compositional matrix adjust.,

Identities:66/231(29%), Positives:101/231(43%), Gaps:51/231(22%)

Query 94 VTYSANFTVGSNSQKQNVIVDTGSSDLWVVDSSANCQEKSGYSSDYCFSGGTYDPSSSST 153

+ Y +G+ +Q V+ DTGSS+LWV S C SS C + ++P SST

Sbjct 74 MEYFGTIGIGTPAQDFTVLFDTGSSNLWV--PSVYC------SSLACTNHNRFNPEDSST 125

Query 154 IQELGKSFNIRYGDGSSSSGTWVKDTV-GINGAIILNQQFGDVNSTSVSQGILGIGLDTN 212

Q ++ +I YG S + D V ING Q+ S IL

Sbjct 126 YQSTSETVSITYGISS------LPDIVFTINGV-----QY----PVPPSAYIL------- 163

Query 213 ESTDTIYENFPINLKEQGFINTNAYSLYLNAPSATSGTIIFGGIDHAKYTGSLTTLPLTS 272

++ + +S+YL+A + +IFGGID + YTGSL +P+T

Sbjct 164 ------------------LVSQDLFSVYLSADDQSGSVVIFGGIDSSYYTGSLNWVPVTV 205

Query 273 NREFTIQTNSATVGTSTIDINTGL--LLDSGTTLTYLPQSVVDSIANAIGG 321

+ I +S T+ I G ++D+GT+L P S + +I + IG

Sbjct 206 EGYWQITVDSITMNGEAIACAEGCQAIVDTGTSLLTGPTSPIANIQSDIGA 256

>gastricsin isoform 2 preproprotein [Homo sapiens]

Sequence ID: NP_001159896.1 Length: 315

>unnamed protein product [Homo sapiens]

Sequence ID: BAG62855.1 Length: 315

Range 1: 73 to 215

Score:57.4 bits(137), Expect:6e-08,

Method:Compositional matrix adjust.,

Identities:46/157(29%), Positives:79/157(50%), Gaps:24/157(15%)

Query 96 YSANFTVGSNSQKQNVIVDTGSSDLWVVDSSANCQEKSGYSSDYCFSGGTYDPSSSSTIQ 155

Y ++G+ Q V+ DTGSS+LWV S CQ S C S ++PS SST

Sbjct 73 YFGEISIGTPPQNFLVLFDTGSSNLWV--PSVYCQ------SQACTSHSRFNPSESSTYS 124

Query 156 ELGKSFNIRYGDGSSSSGTWVKDTVGINGAIILNQQFGDVNSTSVS-------QGILGI- 207

G++F+++YG G S +G + DT+ + + NQ+FG + + GI+G+

Sbjct 125 TNGQTFSLQYGSG-SLTGFFGYDTLTVQSIQVPNQEFGLSENEPGTNFVYAQFDGIMGLA 183

Query 208 --GLDTNESTDTIYENFPINLKEQGFINTNAYSLYLN 242

L +E+T + + ++G + + +S+YL+

Sbjct 184 YPALSVDEATTAMQ-----GMVQEGALTSPVFSVYLS 215

>Crystal Structure of human Beta Secretase complexed with IXS [Homo sapiens]

Sequence ID: 2QZL_A Length: 411

Range 1: 40 to 307

Score:57.8 bits(138), Expect:7e-08,

Method:Compositional matrix adjust.,

Identities:77/289(27%), Positives:117/289(40%), Gaps:58/289(20%)

Query 96 YSANFTVGSNSQKQNVIVDTGSSDLWVVDSSANCQEKSGYSSDYCFSGGTYDPSSSSTIQ 155

Y TVGS Q N++VDTGSS+ V ++ + F Y SST +

Sbjct 40 YYVEMTVGSPPQTLNILVDTGSSNFAVG------------AAPHPFLHRYYQRQLSSTYR 87

Query 156 ELGKSFNIRYGDGSSSSGTWVKD--------------TVGINGAIILNQQFGDVNSTSVS 201

+L K + Y + G W + TV N A I +N ++

Sbjct 88 DLRKGVYVPY-----TQGAWAGELGTDLVSIPHGPNVTVRANIAAITESDKFFINGSNW- 141

Query 202 QGILGIGLDTNESTDTIYENFPINLKEQGFINTNAYSLYL----------NAPSATSGTI 251

+GILG+ D E F +L +Q + N +SL L ++ G++

Sbjct 142 EGILGLAYAEIARPDDSLEPFFDSLVKQTHV-PNLFSLQLCGAGFPLNQSEVLASVGGSM 200

Query 252 IFGGIDHAKYTGSLTTLPLTSNREFTIQTNSATVGTSTIDINTGL--------LLDSGTT 303

I GGIDH+ YTGSL P+ RE+ + V + D+ ++DSGTT

Sbjct 201 IIGGIDHSLYTGSLWYTPI--RREWYYEVIIVRVEINGQDLKMDCKEYNYDKSIVDSGTT 258

Query 304 LTYLPQSVVDSIANAI-GGDITYNRPIGAYIWS---CNRNGKVTYN-FP 347

LP+ V ++ +I T P G ++ C + G +N FP

Sbjct 259 NLRLPKKVFEAAVKSIKAASSTEKFPDGFWLGEQLVCWQAGTTPWNIFP 307

>Crystal structure of human Beta secretase complexed with inhibitor [Homo sapiens]

Sequence ID: 1TQF_A Length: 405

>Crystal structure of human beta secretase complexed with inhibitor [Homo sapiens]

Sequence ID: 2B8L_A Length: 405 >Crystal structure of human Beta-secretase complexed with L-L000430,469 [Homo sapiens]

Sequence ID: 2B8V_A Length: 405 >Crystal structure of human Beta-secretase complexed with inhibitor [Homo sapiens]

Sequence ID: 2IRZ_A Length: 405 >Crystal structure of human Beta-secretase complexed with inhibitor [Homo sapiens]

Sequence ID: 2IS0_A Length: 405 >Crystal structure of Human Bace-1 bound to inhibitor [Homo sapiens]

Sequence ID: 2NTR_A Length: 405 >Crystal Structure of Human Beta Secretase Complexed with inhibitor [Homo sapiens]

Sequence ID: 2OAH_A Length: 405 >Crystal structure of human beta secretase complexed with inhibitor [Homo sapiens]

Sequence ID: 2P8H_A Length: 405 >Crystal Structure of Human Beta Secretase Complexed with inhibitor [Homo sapiens]

Sequence ID: 2PH6_A Length: 405 >Crystal Structure of Human Beta Secretase Complexed with inhibitor [Homo sapiens]

Sequence ID: 2PH8_A Length: 405 >Crystal structure of human Beta Secretase complexed with I21 [Homo sapiens]

Sequence ID: 2QZK_A Length: 405 >Crystal structure of the human BACE1 catalytic domain in complex with N-[1-(5-chloro-2-isopropoxy-3-methoxy-benzyl)-piperidin-4-yl]-2-(4-sulfamoyl-phenoxy)-acetamide [Homo sapiens]

Sequence ID: 2ZJM_A Length: 405 >Crystal Structure of Human Beta Secretase Complexed with Spiropiperdine Iminohydantoin Inhibitor [Homo sapiens]

Sequence ID: 3FKT_A Length: 405 >BACE1 in complex with 4-(cyclohexylamino)-1-(3-fluorophenyl)-8-(3-isopropoxybenzyl)-1,3,8-triazaspiro[4.5]dec-3-en-2-one [Homo sapiens]

Sequence ID: 4ZPE_A Length: 405 >BACE1 in complex with 8-(3-((1-aminopropan-2-yl)oxy)benzyl)-4-(cyclohexylamino)-1-(3-fluorophenyl)-1,3,8-triazaspiro[4.5]dec-3-en-2-one [Homo sapiens]

Sequence ID: 4ZPF_A Length: 405 >BACE1 in complex with 8-benzyl-4-(cyclohexylamino)-1-(3-fluorophenyl)-7-methyl-1,3,8-triazaspiro[4.5]dec-3-en-2-one [Homo sapiens]

Sequence ID: 4ZPG_A Length: 405

Range 1: 34 to 301

Score:57.4 bits(137), Expect:8e-08,

Method:Compositional matrix adjust.,

Identities:77/289(27%), Positives:117/289(40%), Gaps:58/289(20%)

Query 96 YSANFTVGSNSQKQNVIVDTGSSDLWVVDSSANCQEKSGYSSDYCFSGGTYDPSSSSTIQ 155

Y TVGS Q N++VDTGSS+ V ++ + F Y SST +

Sbjct 34 YYVEMTVGSPPQTLNILVDTGSSNFAVG------------AAPHPFLHRYYQRQLSSTYR 81

Query 156 ELGKSFNIRYGDGSSSSGTWVKD--------------TVGINGAIILNQQFGDVNSTSVS 201

+L K + Y + G W + TV N A I +N ++

Sbjct 82 DLRKGVYVPY-----TQGAWAGELGTDLVSIPHGPNVTVRANIAAITESDKFFINGSNW- 135

Query 202 QGILGIGLDTNESTDTIYENFPINLKEQGFINTNAYSLYL----------NAPSATSGTI 251

+GILG+ D E F +L +Q + N +SL L ++ G++

Sbjct 136 EGILGLAYAEIARPDDSLEPFFDSLVKQTHV-PNLFSLQLCGAGFPLNQSEVLASVGGSM 194

Query 252 IFGGIDHAKYTGSLTTLPLTSNREFTIQTNSATVGTSTIDINTGL--------LLDSGTT 303

I GGIDH+ YTGSL P+ RE+ + V + D+ ++DSGTT

Sbjct 195 IIGGIDHSLYTGSLWYTPI--RREWYYEVIIVRVEINGQDLKMDCKEYNYDKSIVDSGTT 252

Query 304 LTYLPQSVVDSIANAI-GGDITYNRPIGAYIWS---CNRNGKVTYN-FP 347

LP+ V ++ +I T P G ++ C + G +N FP

Sbjct 253 NLRLPKKVFEAAVKSIKAASSTEKFPDGFWLGEQLVCWQAGTTPWNIFP 301

>Crystal structure of BACE1 bound to inhibitor [Homo sapiens]

Sequence ID: 3EXO_A Length: 413

Range 1: 34 to 301

Score:57.0 bits(136), Expect:1e-07,

Method:Compositional matrix adjust.,

Identities:77/289(27%), Positives:117/289(40%), Gaps:58/289(20%)

Query 96 YSANFTVGSNSQKQNVIVDTGSSDLWVVDSSANCQEKSGYSSDYCFSGGTYDPSSSSTIQ 155

Y TVGS Q N++VDTGSS+ V ++ + F Y SST +

Sbjct 34 YYVEMTVGSPPQTLNILVDTGSSNFAVG------------AAPHPFLHRYYQRQLSSTYR 81

Query 156 ELGKSFNIRYGDGSSSSGTWVKD--------------TVGINGAIILNQQFGDVNSTSVS 201

+L K + Y + G W + TV N A I +N ++

Sbjct 82 DLRKGVYVPY-----TQGAWAGELGTDLVSIPHGPNVTVRANIAAITESDKFFINGSNW- 135

Query 202 QGILGIGLDTNESTDTIYENFPINLKEQGFINTNAYSLYL----------NAPSATSGTI 251

+GILG+ D E F +L +Q + N +SL L ++ G++

Sbjct 136 EGILGLAYAEIARPDDSLEPFFDSLVKQTHV-PNLFSLQLCGAGFPLNQSEVLASVGGSM 194

Query 252 IFGGIDHAKYTGSLTTLPLTSNREFTIQTNSATVGTSTIDINTGL--------LLDSGTT 303

I GGIDH+ YTGSL P+ RE+ + V + D+ ++DSGTT

Sbjct 195 IIGGIDHSLYTGSLWYTPI--RREWYYEVIIVRVEINGQDLKMDCKEYNYDKSIVDSGTT 252

Query 304 LTYLPQSVVDSIANAI-GGDITYNRPIGAYIWS---CNRNGKVTYN-FP 347

LP+ V ++ +I T P G ++ C + G +N FP

Sbjct 253 NLRLPKKVFEAAVKSIKAASSTEKFPDGFWLGEQLVCWQAGTTPWNIFP 301

>Design and synthesis of hydroxyethylamine (hea) BACE-1 inhibitors: prime side chromane-containing inhibitors [Homo sapiens]

Sequence ID: 3QI1_A Length: 408

Range 1: 30 to 297

Score:57.0 bits(136), Expect:1e-07,

Method:Compositional matrix adjust.,

Identities:77/289(27%), Positives:117/289(40%), Gaps:58/289(20%)

Query 96 YSANFTVGSNSQKQNVIVDTGSSDLWVVDSSANCQEKSGYSSDYCFSGGTYDPSSSSTIQ 155

Y TVGS Q N++VDTGSS+ V ++ + F Y SST +

Sbjct 30 YYVEMTVGSPPQTLNILVDTGSSNFAVG------------AAPHPFLHRYYQRQLSSTYR 77

Query 156 ELGKSFNIRYGDGSSSSGTWVKD--------------TVGINGAIILNQQFGDVNSTSVS 201

+L K + Y + G W + TV N A I +N ++

Sbjct 78 DLRKGVYVPY-----TQGAWAGELGTDLVSIPHGPNVTVRANIAAITESDKFFINGSNW- 131

Query 202 QGILGIGLDTNESTDTIYENFPINLKEQGFINTNAYSLYL----------NAPSATSGTI 251

+GILG+ D E F +L +Q + N +SL L ++ G++

Sbjct 132 EGILGLAYAEIARPDDSLEPFFDSLVKQTHV-PNLFSLQLCGAGFPLNQSEVLASVGGSM 190

Query 252 IFGGIDHAKYTGSLTTLPLTSNREFTIQTNSATVGTSTIDINTGL--------LLDSGTT 303

I GGIDH+ YTGSL P+ RE+ + V + D+ ++DSGTT

Sbjct 191 IIGGIDHSLYTGSLWYTPI--RREWYYEVIIVRVEINGQDLKMDCKEYNYDKSIVDSGTT 248

Query 304 LTYLPQSVVDSIANAI-GGDITYNRPIGAYIWS---CNRNGKVTYN-FP 347

LP+ V ++ +I T P G ++ C + G +N FP

Sbjct 249 NLRLPKKVFEAAVKSIKAASSTEKFPDGFWLGEQLVCWQAGTTPWNIFP 297

>Structure of BACE1 complex with a HEA-type inhibitor [Homo sapiens]

Sequence ID: 4TRY_A Length: 388

>Structure of BACE1 complex with a HEA-type inhibitor [Homo sapiens]

Sequence ID: 4TRY_B Length: 388 >Structure of BACE1 complex with a HEA-type inhibitor [Homo sapiens]

Sequence ID: 4TRY_C Length: 388 >Structure of BACE1 complex with 2-thiophenyl HEA-type inhibitor [Homo sapiens]

Sequence ID: 4TRZ_A Length: 388 >Structure of BACE1 complex with 2-thiophenyl HEA-type inhibitor [Homo sapiens]

Sequence ID: 4TRZ_B Length: 388 >Structure of BACE1 complex with 2-thiophenyl HEA-type inhibitor [Homo sapiens]

Sequence ID: 4TRZ_C Length: 388

Range 1: 16 to 283

Score:57.0 bits(136), Expect:1e-07,

Method:Compositional matrix adjust.,

Identities:77/289(27%), Positives:117/289(40%), Gaps:58/289(20%)

Query 96 YSANFTVGSNSQKQNVIVDTGSSDLWVVDSSANCQEKSGYSSDYCFSGGTYDPSSSSTIQ 155

Y TVGS Q N++VDTGSS+ V ++ + F Y SST +

Sbjct 16 YYVEMTVGSPPQTLNILVDTGSSNFAVG------------AAPHPFLHRYYQRQLSSTYR 63

Query 156 ELGKSFNIRYGDGSSSSGTWVKD--------------TVGINGAIILNQQFGDVNSTSVS 201

+L K + Y + G W + TV N A I +N ++

Sbjct 64 DLRKGVYVPY-----TQGKWEGELGTDLVSIPHGPNVTVRANIAAITESDKFFINGSN-W 117

Query 202 QGILGIGLDTNESTDTIYENFPINLKEQGFINTNAYSLYL----------NAPSATSGTI 251

+GILG+ D E F +L +Q + N +SL L ++ G++

Sbjct 118 EGILGLAYAEIARPDDSLEPFFDSLVKQTHV-PNLFSLQLCGAGFPLNQSEVLASVGGSM 176

Query 252 IFGGIDHAKYTGSLTTLPLTSNREFTIQTNSATVGTSTIDINTGL--------LLDSGTT 303

I GGIDH+ YTGSL P+ RE+ + V + D+ ++DSGTT

Sbjct 177 IIGGIDHSLYTGSLWYTPI--RREWYYEVIIVRVEINGQDLKMDCKEYNYDKSIVDSGTT 234

Query 304 LTYLPQSVVDSIANAI-GGDITYNRPIGAYIWS---CNRNGKVTYN-FP 347

LP+ V ++ +I T P G ++ C + G +N FP

Sbjct 235 NLRLPKKVFEAAVKSIKAASSTEKFPDGFWLGEQLVCWQAGTTPWNIFP 283

>beta-secretase 1 isoform A preproprotein [Homo sapiens]

Sequence ID: NP_036236.1 Length: 501

>RecName: Full=Beta-secretase 1; AltName: Full=Aspartyl protease 2; Short=ASP2; Short=Asp 2; AltName: Full=Beta-site amyloid precursor protein cleaving enzyme 1; Short=Beta-site APP cleaving enzyme 1; AltName: Full=Memapsin-2; AltName: Full=Membrane-associated aspartic protease 2; Flags: Precursor [Homo sapiens]

Sequence ID: P56817.3 Length: 501 >BACE1 compound 23 [Homo sapiens]

Sequence ID: 6EJ3_A Length: 501 >beta-site APP cleaving enzyme [Homo sapiens]

Sequence ID: AAF04142.1 Length: 501 >aspartyl protease 2 [Homo sapiens]

Sequence ID: AAF17079.1 Length: 501 >APP beta-secretase [Homo sapiens]

Sequence ID: AAF18982.1 Length: 501 >transmembrane aspartic proteinase Asp 2 [Homo sapiens]

Sequence ID: AAF26367.1 Length: 501

Range 1: 75 to 342

Score:57.0 bits(136), Expect:1e-07,

Method:Compositional matrix adjust.,

Identities:77/289(27%), Positives:117/289(40%), Gaps:58/289(20%)

Query 96 YSANFTVGSNSQKQNVIVDTGSSDLWVVDSSANCQEKSGYSSDYCFSGGTYDPSSSSTIQ 155

Y TVGS Q N++VDTGSS+ V ++ + F Y SST +

Sbjct 75 YYVEMTVGSPPQTLNILVDTGSSNFAVG------------AAPHPFLHRYYQRQLSSTYR 122

Query 156 ELGKSFNIRYGDGSSSSGTWVKD--------------TVGINGAIILNQQFGDVNSTSVS 201

+L K + Y + G W + TV N A I +N ++

Sbjct 123 DLRKGVYVPY-----TQGKWEGELGTDLVSIPHGPNVTVRANIAAITESDKFFINGSN-W 176

Query 202 QGILGIGLDTNESTDTIYENFPINLKEQGFINTNAYSLYL----------NAPSATSGTI 251

+GILG+ D E F +L +Q + N +SL L ++ G++

Sbjct 177 EGILGLAYAEIARPDDSLEPFFDSLVKQTHV-PNLFSLQLCGAGFPLNQSEVLASVGGSM 235

Query 252 IFGGIDHAKYTGSLTTLPLTSNREFTIQTNSATVGTSTIDINTGL--------LLDSGTT 303

I GGIDH+ YTGSL P+ RE+ + V + D+ ++DSGTT

Sbjct 236 IIGGIDHSLYTGSLWYTPI--RREWYYEVIIVRVEINGQDLKMDCKEYNYDKSIVDSGTT 293

Query 304 LTYLPQSVVDSIANAI-GGDITYNRPIGAYIWS---CNRNGKVTYN-FP 347

LP+ V ++ +I T P G ++ C + G +N FP

Sbjct 294 NLRLPKKVFEAAVKSIKAASSTEKFPDGFWLGEQLVCWQAGTTPWNIFP 342

Range 2: 75 to 342

Score:57.0 bits(136), Expect:1e-07,

Method:Compositional matrix adjust.,

Identities:77/289(27%), Positives:117/289(40%), Gaps:58/289(20%)

Query 96 YSANFTVGSNSQKQNVIVDTGSSDLWVVDSSANCQEKSGYSSDYCFSGGTYDPSSSSTIQ 155

Y TVGS Q N++VDTGSS+ V ++ + F Y SST +

Sbjct 75 YYVEMTVGSPPQTLNILVDTGSSNFAVG------------AAPHPFLHRYYQRQLSSTYR 122

Query 156 ELGKSFNIRYGDGSSSSGTWVKD--------------TVGINGAIILNQQFGDVNSTSVS 201

+L K + Y + G W + TV N A I +N ++

Sbjct 123 DLRKGVYVPY-----TQGKWEGELGTDLVSIPHGPNVTVRANIAAITESDKFFINGSN-W 176

Query 202 QGILGIGLDTNESTDTIYENFPINLKEQGFINTNAYSLYL----------NAPSATSGTI 251

+GILG+ D E F +L +Q + N +SL L ++ G++

Sbjct 177 EGILGLAYAEIARPDDSLEPFFDSLVKQTHV-PNLFSLQLCGAGFPLNQSEVLASVGGSM 235

Query 252 IFGGIDHAKYTGSLTTLPLTSNREFTIQTNSATVGTSTIDINTGL--------LLDSGTT 303

I GGIDH+ YTGSL P+ RE+ + V + D+ ++DSGTT

Sbjct 236 IIGGIDHSLYTGSLWYTPI--RREWYYEVIIVRVEINGQDLKMDCKEYNYDKSIVDSGTT 293

Query 304 LTYLPQSVVDSIANAI-GGDITYNRPIGAYIWS---CNRNGKVTYN-FP 347

LP+ V ++ +I T P G ++ C + G +N FP

Sbjct 294 NLRLPKKVFEAAVKSIKAASSTEKFPDGFWLGEQLVCWQAGTTPWNIFP 342

>BACE1 compound 28 [Homo sapiens]

Sequence ID: 6EJ2_A Length: 501

Range 1: 75 to 342

Score:57.0 bits(136), Expect:1e-07,

Method:Compositional matrix adjust.,

Identities:77/289(27%), Positives:117/289(40%), Gaps:58/289(20%)

Query 96 YSANFTVGSNSQKQNVIVDTGSSDLWVVDSSANCQEKSGYSSDYCFSGGTYDPSSSSTIQ 155

Y TVGS Q N++VDTGSS+ V ++ + F Y SST +

Sbjct 75 YYVEMTVGSPPQTLNILVDTGSSNFAVG------------AAPHPFLHRYYQRQLSSTYR 122

Query 156 ELGKSFNIRYGDGSSSSGTWVKD--------------TVGINGAIILNQQFGDVNSTSVS 201

+L K + Y + G W + TV N A I +N ++

Sbjct 123 DLRKGVYVPY-----TQGKWEGELGTDLVSIPHGPNVTVRANIAAITESDKFFINGSN-W 176

Query 202 QGILGIGLDTNESTDTIYENFPINLKEQGFINTNAYSLYL----------NAPSATSGTI 251

+GILG+ D E F +L +Q + N +SL L ++ G++

Sbjct 177 EGILGLAYAEIARPDDSLEPFFDSLVKQTHV-PNLFSLQLCGAGFPLNQSEVLASVGGSM 235

Query 252 IFGGIDHAKYTGSLTTLPLTSNREFTIQTNSATVGTSTIDINTGL--------LLDSGTT 303

I GGIDH+ YTGSL P+ RE+ + V + D+ ++DSGTT

Sbjct 236 IIGGIDHSLYTGSLWYTPI--RREWYYEVIIVRVEINGQDLKMDCKEYNYDKSIVDSGTT 293

Query 304 LTYLPQSVVDSIANAI-GGDITYNRPIGAYIWS---CNRNGKVTYN-FP 347

LP+ V ++ +I T P G ++ C + G +N FP

Sbjct 294 NLRLPKKVFEAAVKSIKAASSTEKFPDGFWLGEQLVCWQAGTTPWNIFP 342

>Aminoimidazoles as BACE-1 Inhibitors: From De Novo Design to Ab- lowering in Brain [Homo sapiens]

Sequence ID: 4B70_A Length: 385

Range 1: 15 to 282

Score:56.6 bits(135), Expect:1e-07,

Method:Compositional matrix adjust.,

Identities:77/289(27%), Positives:117/289(40%), Gaps:58/289(20%)

Query 96 YSANFTVGSNSQKQNVIVDTGSSDLWVVDSSANCQEKSGYSSDYCFSGGTYDPSSSSTIQ 155

Y TVGS Q N++VDTGSS+ V ++ + F Y SST +

Sbjct 15 YYVEMTVGSPPQTLNILVDTGSSNFAVG------------AAPHPFLHRYYQRQLSSTYR 62

Query 156 ELGKSFNIRYGDGSSSSGTWVKD--------------TVGINGAIILNQQFGDVNSTSVS 201

+L K + Y + G W + TV N A I +N ++

Sbjct 63 DLRKGVYVPY-----TQGKWEGELGTDLVSIPHGPNVTVRANIAAITESDKFFINGSN-W 116

Query 202 QGILGIGLDTNESTDTIYENFPINLKEQGFINTNAYSLYL----------NAPSATSGTI 251

+GILG+ D E F +L +Q + N +SL L ++ G++

Sbjct 117 EGILGLAYAEIARPDDSLEPFFDSLVKQTHV-PNLFSLQLCGAGFPLNQSEVLASVGGSM 175

Query 252 IFGGIDHAKYTGSLTTLPLTSNREFTIQTNSATVGTSTIDINTGL--------LLDSGTT 303

I GGIDH+ YTGSL P+ RE+ + V + D+ ++DSGTT

Sbjct 176 IIGGIDHSLYTGSLWYTPI--RREWYYEVIIVRVEINGQDLKMDCKEYNYDKSIVDSGTT 233

Query 304 LTYLPQSVVDSIANAI-GGDITYNRPIGAYIWS---CNRNGKVTYN-FP 347

LP+ V ++ +I T P G ++ C + G +N FP

Sbjct 234 NLRLPKKVFEAAVKSIKAASSTEKFPDGFWLGEQLVCWQAGTTPWNIFP 282

>Lead Generation of BACE1 Inhibitors by Coupling Non-amidine New Warheads to a Known Binding Scaffold [Homo sapiens]

Sequence ID: 4B0Q_A Length: 384

>Aminoimidazoles as BACE-1 Inhibitors: From De Novo Design to Ab- lowering in Brain [Homo sapiens]

Sequence ID: 4B78_A Length: 384

Range 1: 14 to 281

Score:56.6 bits(135), Expect:1e-07,

Method:Compositional matrix adjust.,

Identities:77/289(27%), Positives:117/289(40%), Gaps:58/289(20%)

Query 96 YSANFTVGSNSQKQNVIVDTGSSDLWVVDSSANCQEKSGYSSDYCFSGGTYDPSSSSTIQ 155

Y TVGS Q N++VDTGSS+ V ++ + F Y SST +

Sbjct 14 YYVEMTVGSPPQTLNILVDTGSSNFAVG------------AAPHPFLHRYYQRQLSSTYR 61

Query 156 ELGKSFNIRYGDGSSSSGTWVKD--------------TVGINGAIILNQQFGDVNSTSVS 201

+L K + Y + G W + TV N A I +N ++

Sbjct 62 DLRKGVYVPY-----TQGKWEGELGTDLVSIPHGPNVTVRANIAAITESDKFFINGSN-W 115

Query 202 QGILGIGLDTNESTDTIYENFPINLKEQGFINTNAYSLYL----------NAPSATSGTI 251

+GILG+ D E F +L +Q + N +SL L ++ G++

Sbjct 116 EGILGLAYAEIARPDDSLEPFFDSLVKQTHV-PNLFSLQLCGAGFPLNQSEVLASVGGSM 174

Query 252 IFGGIDHAKYTGSLTTLPLTSNREFTIQTNSATVGTSTIDINTGL--------LLDSGTT 303

I GGIDH+ YTGSL P+ RE+ + V + D+ ++DSGTT

Sbjct 175 IIGGIDHSLYTGSLWYTPI--RREWYYEVIIVRVEINGQDLKMDCKEYNYDKSIVDSGTT 232

Query 304 LTYLPQSVVDSIANAI-GGDITYNRPIGAYIWS---CNRNGKVTYN-FP 347

LP+ V ++ +I T P G ++ C + G +N FP

Sbjct 233 NLRLPKKVFEAAVKSIKAASSTEKFPDGFWLGEQLVCWQAGTTPWNIFP 281

>New Aminoimidazoles as BACE-1 Inhibitors: From Rational Design to Ab- lowering in Brain [Homo sapiens]

Sequence ID: 4B1D_A Length: 388

>New Aminoimidazoles as BACE-1 Inhibitors: From Rational Design to Ab- lowering in Brain [Homo sapiens]

Sequence ID: 4B1E_A Length: 388 >Aminoimidazoles as BACE-1 Inhibitors: From De Novo Design to Ab- lowering in Brain [Homo sapiens]

Sequence ID: 4B72_A Length: 388 >Aminoimidazoles as BACE-1 Inhibitors: From De Novo Design to Ab- lowering in Brain [Homo sapiens]

Sequence ID: 4B77_A Length: 388

Range 1: 18 to 285

Score:56.6 bits(135), Expect:1e-07,

Method:Compositional matrix adjust.,

Identities:77/289(27%), Positives:117/289(40%), Gaps:58/289(20%)

Query 96 YSANFTVGSNSQKQNVIVDTGSSDLWVVDSSANCQEKSGYSSDYCFSGGTYDPSSSSTIQ 155

Y TVGS Q N++VDTGSS+ V ++ + F Y SST +

Sbjct 18 YYVEMTVGSPPQTLNILVDTGSSNFAVG------------AAPHPFLHRYYQRQLSSTYR 65

Query 156 ELGKSFNIRYGDGSSSSGTWVKD--------------TVGINGAIILNQQFGDVNSTSVS 201

+L K + Y + G W + TV N A I +N ++

Sbjct 66 DLRKGVYVPY-----TQGKWEGELGTDLVSIPHGPNVTVRANIAAITESDKFFINGSN-W 119

Query 202 QGILGIGLDTNESTDTIYENFPINLKEQGFINTNAYSLYL----------NAPSATSGTI 251

+GILG+ D E F +L +Q + N +SL L ++ G++

Sbjct 120 EGILGLAYAEIARPDDSLEPFFDSLVKQTHV-PNLFSLQLCGAGFPLNQSEVLASVGGSM 178

Query 252 IFGGIDHAKYTGSLTTLPLTSNREFTIQTNSATVGTSTIDINTGL--------LLDSGTT 303

I GGIDH+ YTGSL P+ RE+ + V + D+ ++DSGTT

Sbjct 179 IIGGIDHSLYTGSLWYTPI--RREWYYEVIIVRVEINGQDLKMDCKEYNYDKSIVDSGTT 236

Query 304 LTYLPQSVVDSIANAI-GGDITYNRPIGAYIWS---CNRNGKVTYN-FP 347

LP+ V ++ +I T P G ++ C + G +N FP

Sbjct 237 NLRLPKKVFEAAVKSIKAASSTEKFPDGFWLGEQLVCWQAGTTPWNIFP 285

>Crystal structure of the human BACE1 catalytic domain in complex with N-[1-(2,6-dimethoxy-benzyl)-piperidin-4-yl]-4-mercapto-butyramide [Homo sapiens]

Sequence ID: 2ZJI_A Length: 405

Range 1: 34 to 301

Score:56.6 bits(135), Expect:1e-07,

Method:Compositional matrix adjust.,

Identities:77/289(27%), Positives:117/289(40%), Gaps:58/289(20%)

Query 96 YSANFTVGSNSQKQNVIVDTGSSDLWVVDSSANCQEKSGYSSDYCFSGGTYDPSSSSTIQ 155

Y TVGS Q N++VDTGSS+ V ++ + F Y SST +

Sbjct 34 YYVEMTVGSPPQTLNILVDTGSSNFAVG------------AAPHPFLHRYYQRQLSSTYR 81

Query 156 ELGKSFNIRYGDGSSSSGTWVKD--------------TVGINGAIILNQQFGDVNSTSVS 201

+L K + Y + G W + TV N A I +N ++

Sbjct 82 DLRKGVYVPY-----TQGKWEGELGTDLVSIPHGPNVTVRANIAAITESDKFFINGSNW- 135

Query 202 QGILGIGLDTNESTDTIYENFPINLKEQGFINTNAYSLYL----------NAPSATSGTI 251

+GILG+ D E F +L +Q + N +SL L ++ G++

Sbjct 136 EGILGLAYAEIARPDDSLEPFFDSLVKQTHV-PNLFSLQLCGAGFPLNQSEVLASVGGSM 194

Query 252 IFGGIDHAKYTGSLTTLPLTSNREFTIQTNSATVGTSTIDINTGL--------LLDSGTT 303

I GGIDH+ YTGSL P+ RE+ + V + D+ ++DSGTT

Sbjct 195 IIGGIDHSLYTGSLWYTPI--RREWYYEVIIVRVEINGQDLKMDCKEYNYDKSIVDSGTT 252

Query 304 LTYLPQSVVDSIANAI-GGDITYNRPIGAYIWS---CNRNGKVTYN-FP 347

LP+ V ++ +I T P G ++ C + G +N FP

Sbjct 253 NLRLPKKVFEAAVKSIKAASSTEKFPDGFWLGEQLVCWQAGTTPWNIFP 301

>APO structure of BACE1 [Homo sapiens]

Sequence ID: 3TPJ_A Length: 433

>APO Structure of BACE1 [Homo sapiens]

Sequence ID: 3TPL_A Length: 433 >APO Structure of BACE1 [Homo sapiens]

Sequence ID: 3TPL_B Length: 433 >APO Structure of BACE1 [Homo sapiens]

Sequence ID: 3TPL_C Length: 433 >Crystal structure of BACE1 complexed with an inhibitor [Homo sapiens]

Sequence ID: 3TPP_A Length: 433 >Crystal structure of Bace1 with its inhibitor [Homo sapiens]

Sequence ID: 3UQP_A Length: 433 >Crystal structure of BACE1 with its inhibitor [Homo sapiens]

Sequence ID: 3UQU_A Length: 433 >Crystal structure of BACE1 with its inhibitor [Homo sapiens]

Sequence ID: 3UQW_A Length: 433 >Crystal structure of BACE1 with its inhibitor [Homo sapiens]

Sequence ID: 3UQX_A Length: 433 >Crystal structure of BACE1 with its inhibitor [Homo sapiens]

Sequence ID: 4DV9_A Length: 433 >Crystal structure of BACE1 with its inhibitor [Homo sapiens]

Sequence ID: 4DVF_A Length: 433 >Crystal structure of BACE1 with its inhibitor [Homo sapiens]

Sequence ID: 4DVF_B Length: 433 >Crystal structure of bace1 with its inhibitor [Homo sapiens]

Sequence ID: 4FCO_A Length: 433 >Crystal structure of bace1 with novel inhibitor [Homo sapiens]

Sequence ID: 4FGX_A Length: 433 >Crystal structure of BACE1 with its inhibitor [Homo sapiens]

Sequence ID: 4IVS_A Length: 433 >Crystal structure of BACE1 with its inhibitor [Homo sapiens]

Sequence ID: 4IVT_A Length: 433

Range 1: 54 to 321

Score:57.0 bits(136), Expect:1e-07,

Method:Compositional matrix adjust.,

Identities:77/289(27%), Positives:117/289(40%), Gaps:58/289(20%)

Query 96 YSANFTVGSNSQKQNVIVDTGSSDLWVVDSSANCQEKSGYSSDYCFSGGTYDPSSSSTIQ 155

Y TVGS Q N++VDTGSS+ V ++ + F Y SST +

Sbjct 54 YYVEMTVGSPPQTLNILVDTGSSNFAVG------------AAPHPFLHRYYQRQLSSTYR 101

Query 156 ELGKSFNIRYGDGSSSSGTWVKD--------------TVGINGAIILNQQFGDVNSTSVS 201

+L K + Y + G W + TV N A I +N ++

Sbjct 102 DLRKGVYVPY-----TQGAWAGELGTDLVSIPHGPNVTVRANIAAITESDKFFINGSNW- 155

Query 202 QGILGIGLDTNESTDTIYENFPINLKEQGFINTNAYSLYLNAP----------SATSGTI 251

+GILG+ D E F +L +Q + N +SL L ++ G++

Sbjct 156 EGILGLAYAEIARPDDSLEPFFDSLVKQTHV-PNLFSLQLCGAGFPLNQSEVLASVGGSM 214

Query 252 IFGGIDHAKYTGSLTTLPLTSNREFTIQTNSATVGTSTIDINTGL--------LLDSGTT 303

I GGIDH+ YTGSL P+ RE+ + V + D+ ++DSGTT

Sbjct 215 IIGGIDHSLYTGSLWYTPI--RREWYYEVIIVRVEINGQDLKMDCKEYNYDKSIVDSGTT 272

Query 304 LTYLPQSVVDSIANAI-GGDITYNRPIGAYIWS---CNRNGKVTYN-FP 347

LP+ V ++ +I T P G ++ C + G +N FP

Sbjct 273 NLRLPKKVFEAAVKSIKAASSTEKFPDGFWLGEQLVCWQAGTTPWNIFP 321

>Crystal structure of beta-secretase complexed with an amino-ethylene inhibitor [Homo sapiens]

Sequence ID: 2FDP_A Length: 388

>Crystal structure of beta-secretase complexed with an amino-ethylene inhibitor [Homo sapiens]

Sequence ID: 2FDP_B Length: 388 >Crystal structure of beta-secretase complexed with an amino-ethylene inhibitor [Homo sapiens]

Sequence ID: 2FDP_C Length: 388 >Crystal structure of beta-secretase 1 in complex with selective beta-secretase 1 inhibitor [Homo sapiens]

Sequence ID: 3IXJ_A Length: 388 >Crystal structure of beta-secretase 1 in complex with selective beta-secretase 1 inhibitor [Homo sapiens]

Sequence ID: 3IXJ_B Length: 388 >Crystal structure of beta-secretase 1 in complex with selective beta-secretase 1 inhibitor [Homo sapiens]

Sequence ID: 3IXJ_C Length: 388 >Structure of beta-secretase complexed with inhibitor [Homo sapiens]

Sequence ID: 4GID_A Length: 388 >Structure of beta-secretase complexed with inhibitor [Homo sapiens]

Sequence ID: 4GID_B Length: 388 >Structure of beta-secretase complexed with inhibitor [Homo sapiens]

Sequence ID: 4GID_C Length: 388 >Structure of beta-secretase complexed with inhibitor [Homo sapiens]

Sequence ID: 4GID_D Length: 388 >Hydroxyethylamine-based inhibitors of BACE1: P1-P3 macrocyclization can improve potency, selectivity, and cell activity [Homo sapiens]

Sequence ID: 4K8S_A Length: 388 >Hydroxyethylamine-based inhibitors of BACE1: P1-P3 macrocyclization can improve potency, selectivity, and cell activity [Homo sapiens]

Sequence ID: 4K8S_B Length: 388 >Hydroxyethylamine-based inhibitors of BACE1: P1-P3 macrocyclization can improve potency, selectivity, and cell activity [Homo sapiens]

Sequence ID: 4K8S_C Length: 388 >Bace-1 inhibitor complex [Homo sapiens]

Sequence ID: 4K9H_A Length: 388 >Bace-1 inhibitor complex [Homo sapiens]

Sequence ID: 4K9H_B Length: 388 >Bace-1 inhibitor complex [Homo sapiens]

Sequence ID: 4K9H_C Length: 388

Range 1: 17 to 284

Score:56.6 bits(135), Expect:1e-07,

Method:Compositional matrix adjust.,

Identities:77/289(27%), Positives:117/289(40%), Gaps:58/289(20%)

Query 96 YSANFTVGSNSQKQNVIVDTGSSDLWVVDSSANCQEKSGYSSDYCFSGGTYDPSSSSTIQ 155

Y TVGS Q N++VDTGSS+ V ++ + F Y SST +

Sbjct 17 YYVEMTVGSPPQTLNILVDTGSSNFAVG------------AAPHPFLHRYYQRQLSSTYR 64

Query 156 ELGKSFNIRYGDGSSSSGTWVKD--------------TVGINGAIILNQQFGDVNSTSVS 201

+L K + Y + G W + TV N A I +N ++

Sbjct 65 DLRKGVYVPY-----TQGKWEGELGTDLVSIPHGPNVTVRANIAAITESDKFFINGSN-W 118

Query 202 QGILGIGLDTNESTDTIYENFPINLKEQGFINTNAYSLYL----------NAPSATSGTI 251

+GILG+ D E F +L +Q + N +SL L ++ G++

Sbjct 119 EGILGLAYAEIARPDDSLEPFFDSLVKQTHV-PNLFSLQLCGAGFPLNQSEVLASVGGSM 177

Query 252 IFGGIDHAKYTGSLTTLPLTSNREFTIQTNSATVGTSTIDINTGL--------LLDSGTT 303

I GGIDH+ YTGSL P+ RE+ + V + D+ ++DSGTT

Sbjct 178 IIGGIDHSLYTGSLWYTPI--RREWYYEVIIVRVEINGQDLKMDCKEYNYDKSIVDSGTT 235

Query 304 LTYLPQSVVDSIANAI-GGDITYNRPIGAYIWS---CNRNGKVTYN-FP 347

LP+ V ++ +I T P G ++ C + G +N FP

Sbjct 236 NLRLPKKVFEAAVKSIKAASSTEKFPDGFWLGEQLVCWQAGTTPWNIFP 284

>beta-secretase 1 precursor variant 1 [Homo sapiens]

Sequence ID: ACA05927.1 Length: 501

Range 1: 75 to 342

Score:57.0 bits(136), Expect:1e-07,

Method:Compositional matrix adjust.,

Identities:77/289(27%), Positives:117/289(40%), Gaps:58/289(20%)

Query 96 YSANFTVGSNSQKQNVIVDTGSSDLWVVDSSANCQEKSGYSSDYCFSGGTYDPSSSSTIQ 155

Y TVGS Q N++VDTGSS+ V ++ + F Y SST +

Sbjct 75 YYVEMTVGSPPQTLNILVDTGSSNFAVG------------AAPHPFLHRYYQRQLSSTYR 122

Query 156 ELGKSFNIRYGDGSSSSGTWVKD--------------TVGINGAIILNQQFGDVNSTSVS 201

+L K + Y + G W + TV N A I +N ++

Sbjct 123 DLRKGVYVPY-----TQGKWEGELGTDLVSIPHGPNVTVRANIAAITESDKFFINGSN-W 176

Query 202 QGILGIGLDTNESTDTIYENFPINLKEQGFINTNAYSLYLNAP----------SATSGTI 251

+GILG+ D E F +L +Q + N +SL L ++ G++

Sbjct 177 EGILGLAYAEIARPDDSLEPFFDSLVKQTHV-PNLFSLQLCGAGFPLNQSEVLASVGGSM 235

Query 252 IFGGIDHAKYTGSLTTLPLTSNREFTIQTNSATVGTSTIDINTGL--------LLDSGTT 303

I GGIDH+ YTGSL P+ RE+ + V + D+ ++DSGTT

Sbjct 236 IIGGIDHSLYTGSLWYTPI--RREWYYEVIIVRVEINGQDLKMDCKEYNYDKSIVDSGTT 293

Query 304 LTYLPQSVVDSIANAI-GGDITYNRPIGAYIWS---CNRNGKVTYN-FP 347

LP+ V ++ +I T P G ++ C + G +N FP

Sbjct 294 NLRLPKKVFEAAVKSIKAASSTEKFPDGFWLGEQLVCWQAGTTPWNIFP 342

>Design and synthesis of potent hydroxyethylamine (hea) bace-1 inhibitors [Homo sapiens]

Sequence ID: 4EWO_A Length: 386

>Design and synthesis of potent hydroxyethylamine (hea) bace-1 inhibitors [Homo sapiens]

Sequence ID: 4EXG_A Length: 386

Range 1: 15 to 282

Score:56.6 bits(135), Expect:1e-07,

Method:Compositional matrix adjust.,

Identities:77/289(27%), Positives:117/289(40%), Gaps:58/289(20%)

Query 96 YSANFTVGSNSQKQNVIVDTGSSDLWVVDSSANCQEKSGYSSDYCFSGGTYDPSSSSTIQ 155

Y TVGS Q N++VDTGSS+ V ++ + F Y SST +

Sbjct 15 YYVEMTVGSPPQTLNILVDTGSSNFAVG------------AAPHPFLHRYYQRQLSSTYR 62

Query 156 ELGKSFNIRYGDGSSSSGTWVKD--------------TVGINGAIILNQQFGDVNSTSVS 201

+L K + Y + G W + TV N A I +N ++

Sbjct 63 DLRKGVYVPY-----TQGKWEGELGTDLVSIPHGPNVTVRANIAAITESDKFFINGSN-W 116

Query 202 QGILGIGLDTNESTDTIYENFPINLKEQGFINTNAYSLYL----------NAPSATSGTI 251

+GILG+ D E F +L +Q + N +SL L ++ G++

Sbjct 117 EGILGLAYAEIARPDDSLEPFFDSLVKQTHV-PNLFSLQLCGAGFPLNQSEVLASVGGSM 175

Query 252 IFGGIDHAKYTGSLTTLPLTSNREFTIQTNSATVGTSTIDINTGL--------LLDSGTT 303

I GGIDH+ YTGSL P+ RE+ + V + D+ ++DSGTT

Sbjct 176 IIGGIDHSLYTGSLWYTPI--RREWYYEVIIVRVEINGQDLKMDCKEYNYDKSIVDSGTT 233

Query 304 LTYLPQSVVDSIANAI-GGDITYNRPIGAYIWS---CNRNGKVTYN-FP 347

LP+ V ++ +I T P G ++ C + G +N FP

Sbjct 234 NLRLPKKVFEAAVKSIKAASSTEKFPDGFWLGEQLVCWQAGTTPWNIFP 282

>Structure of BACE bound to SCH346572 [Homo sapiens]

Sequence ID: 3KMX_A Length: 395

>Structure of BACE bound to SCH346572 [Homo sapiens]

Sequence ID: 3KMX_B Length: 395 >Structure of BACE bound to SCH12472 [Homo sapiens]

Sequence ID: 3KMY_A Length: 395 >Structure of BACE bound to SCH12472 [Homo sapiens]

Sequence ID: 3KMY_B Length: 395 >Structure of BACE bound to SCH708236 [Homo sapiens]

Sequence ID: 3KN0_A Length: 395 >Structure of BACE bound to SCH708236 [Homo sapiens]

Sequence ID: 3KN0_B Length: 395 >Structure of BACE bound to SCH743813 [Homo sapiens]

Sequence ID: 3LNK_A Length: 395 >Structure of BACE bound to SCH743813 [Homo sapiens]

Sequence ID: 3LNK_B Length: 395 >Structure of BACE in complex with (S)-4-(3-chloro-5-(5-(prop-1-yn-1-yl)pyridin-3-yl)thiophen-2-yl)-1,4-dimethyl-6-oxotetrahydropyrimidin-2(1H)-iminium [Homo sapiens]

Sequence ID: 4FRS_A Length: 395 >Structure of BACE in complex with (S)-4-(3-chloro-5-(5-(prop-1-yn-1-yl)pyridin-3-yl)thiophen-2-yl)-1,4-dimethyl-6-oxotetrahydropyrimidin-2(1H)-iminium [Homo sapiens]

Sequence ID: 4FRS_B Length: 395

Range 1: 23 to 290

Score:56.6 bits(135), Expect:1e-07,

Method:Compositional matrix adjust.,

Identities:77/289(27%), Positives:117/289(40%), Gaps:58/289(20%)

Query 96 YSANFTVGSNSQKQNVIVDTGSSDLWVVDSSANCQEKSGYSSDYCFSGGTYDPSSSSTIQ 155

Y TVGS Q N++VDTGSS+ V ++ + F Y SST +

Sbjct 23 YYVEMTVGSPPQTLNILVDTGSSNFAVG------------AAPHPFLHRYYQRQLSSTYR 70

Query 156 ELGKSFNIRYGDGSSSSGTWVKD--------------TVGINGAIILNQQFGDVNSTSVS 201

+L K + Y + G W + TV N A I +N ++

Sbjct 71 DLRKGVYVPY-----TQGKWEGELGTDLVSIPHGPNVTVRANIAAITESDKFFINGSN-W 124

Query 202 QGILGIGLDTNESTDTIYENFPINLKEQGFINTNAYSLYL----------NAPSATSGTI 251

+GILG+ D E F +L +Q + N +SL L ++ G++

Sbjct 125 EGILGLAYAEIARPDDSLEPFFDSLVKQTHV-PNLFSLQLCGAGFPLNQSEVLASVGGSM 183

Query 252 IFGGIDHAKYTGSLTTLPLTSNREFTIQTNSATVGTSTIDINTGL--------LLDSGTT 303

I GGIDH+ YTGSL P+ RE+ + V + D+ ++DSGTT

Sbjct 184 IIGGIDHSLYTGSLWYTPI--RREWYYEVIIVRVEINGQDLKMDCKEYNYDKSIVDSGTT 241

Query 304 LTYLPQSVVDSIANAI-GGDITYNRPIGAYIWS---CNRNGKVTYN-FP 347

LP+ V ++ +I T P G ++ C + G +N FP

Sbjct 242 NLRLPKKVFEAAVKSIKAASSTEKFPDGFWLGEQLVCWQAGTTPWNIFP 290

>Structure of BACE1 bound to SCH626485 [Homo sapiens]

Sequence ID: 2QK5_A Length: 395

>Structure of BACE1 bound to SCH626485 [Homo sapiens]

Sequence ID: 2QK5_B Length: 395 >Structure of BACE Bound to SCH722924 [Homo sapiens]

Sequence ID: 2QMD_A Length: 395 >Structure of BACE Bound to SCH722924 [Homo sapiens]

Sequence ID: 2QMD_B Length: 395 >Structure of BACE Bound to SCH735310 [Homo sapiens]

Sequence ID: 2QMF_A Length: 395 >Structure of BACE Bound to SCH735310 [Homo sapiens]

Sequence ID: 2QMF_B Length: 395 >Structure of BACE Bound to SCH745966 [Homo sapiens]

Sequence ID: 2QMG_A Length: 395 >Structure of BACE Bound to SCH745966 [Homo sapiens]

Sequence ID: 2QMG_B Length: 395 >Structure of BACE Bound to SCH734723 [Homo sapiens]

Sequence ID: 2QP8_A Length: 395 >Structure of BACE Bound to SCH734723 [Homo sapiens]

Sequence ID: 2QP8_B Length: 395

Range 1: 23 to 290

Score:56.6 bits(135), Expect:1e-07,

Method:Compositional matrix adjust.,

Identities:77/289(27%), Positives:117/289(40%), Gaps:58/289(20%)

Query 96 YSANFTVGSNSQKQNVIVDTGSSDLWVVDSSANCQEKSGYSSDYCFSGGTYDPSSSSTIQ 155

Y TVGS Q N++VDTGSS+ V ++ + F Y SST +

Sbjct 23 YYVEMTVGSPPQTLNILVDTGSSNFAVG------------AAPHPFLHRYYQRQLSSTYR 70

Query 156 ELGKSFNIRYGDGSSSSGTWVKD--------------TVGINGAIILNQQFGDVNSTSVS 201

+L K + Y + G W + TV N A I +N ++

Sbjct 71 DLRKGVYVPY-----TQGKWEGELGTDLVSIPHGPNVTVRANIAAITESDKFFINGSN-W 124

Query 202 QGILGIGLDTNESTDTIYENFPINLKEQGFINTNAYSLYL----------NAPSATSGTI 251

+GILG+ D E F +L +Q + N +SL L ++ G++

Sbjct 125 EGILGLAYAEIARPDDSLEPFFDSLVKQTHV-PNLFSLQLCGAGFPLNQSEVLASVGGSM 183

Query 252 IFGGIDHAKYTGSLTTLPLTSNREFTIQTNSATVGTSTIDINTGL--------LLDSGTT 303

I GGIDH+ YTGSL P+ RE+ + V + D+ ++DSGTT

Sbjct 184 IIGGIDHSLYTGSLWYTPI--RREWYYEVIIVRVEINGQDLKMDCKEYNYDKSIVDSGTT 241

Query 304 LTYLPQSVVDSIANAI-GGDITYNRPIGAYIWS---CNRNGKVTYN-FP 347

LP+ V ++ +I T P G ++ C + G +N FP

Sbjct 242 NLRLPKKVFEAAVKSIKAASSTEKFPDGFWLGEQLVCWQAGTTPWNIFP 290

>Structure of BACE Bound to SCH727596 [Homo sapiens]

Sequence ID: 3CIB_A Length: 390

>Structure of BACE Bound to SCH727596 [Homo sapiens]

Sequence ID: 3CIB_B Length: 390 >Structure of BACE Bound to SCH709583 [Homo sapiens]

Sequence ID: 3CIC_A Length: 390 >Structure of BACE Bound to SCH709583 [Homo sapiens]

Sequence ID: 3CIC_B Length: 390 >Structure of BACE Bound to SCH726222 [Homo sapiens]

Sequence ID: 3CID_A Length: 390 >Structure of BACE Bound to SCH726222 [Homo sapiens]

Sequence ID: 3CID_B Length: 390 >Structure of BACE Bound to (S)-4-(3'-methoxy-[1,1'-biphenyl]-3-yl)-1,4-dimethyl-6-oxotetrahydropyrimidin-2(1H)-iminium [Homo sapiens]

Sequence ID: 4FS4_A Length: 390 >Structure of BACE Bound to (S)-4-(3'-methoxy-[1,1'-biphenyl]-3-yl)-1,4-dimethyl-6-oxotetrahydropyrimidin-2(1H)-iminium [Homo sapiens]

Sequence ID: 4FS4_B Length: 390 >Structure of BACE1 complex with a syn-HEA-type inhibitor [Homo sapiens]

Sequence ID: 4TRW_A Length: 390 >Structure of BACE1 complex with a syn-HEA-type inhibitor [Homo sapiens]

Sequence ID: 4TRW_B Length: 390 >Structure of BACE1 complex with a syn-HEA-type inhibitor [Homo sapiens]

Sequence ID: 4TRW_C Length: 390 >Co-crystal of BACE1 with compound 0211 [Homo sapiens]

Sequence ID: 5DQC_A Length: 390 >Co-crystal of BACE1 with compound 0211 [Homo sapiens]

Sequence ID: 5DQC_B Length: 390 >Co-crystal of BACE1 with compound 0211 [Homo sapiens]

Sequence ID: 5DQC_C Length: 390 >BACE1 in complex with a macrocyclic inhibitor [Homo sapiens]

Sequence ID: 6NV7_A Length: 390 >BACE1 in complex with a macrocyclic inhibitor [Homo sapiens]

Sequence ID: 6NV7_B Length: 390 >BACE1 in complex with a macrocyclic inhibitor [Homo sapiens]

Sequence ID: 6NV7_C Length: 390 >BACE1 in complex with a macrocyclic inhibitor [Homo sapiens]

Sequence ID: 6NV9_A Length: 390 >BACE1 in complex with a macrocyclic inhibitor [Homo sapiens]

Sequence ID: 6NV9_B Length: 390 >BACE1 in complex with a macrocyclic inhibitor [Homo sapiens]

Sequence ID: 6NV9_C Length: 390 >BACE1 in complex with a macrocyclic inhibitor [Homo sapiens]

Sequence ID: 6NW3_A Length: 390 >BACE1 in complex with a macrocyclic inhibitor [Homo sapiens]

Sequence ID: 6NW3_B Length: 390 >BACE1 in complex with a macrocyclic inhibitor [Homo sapiens]

Sequence ID: 6NW3_C Length: 390

Range 1: 18 to 285

Score:56.6 bits(135), Expect:1e-07,

Method:Compositional matrix adjust.,

Identities:77/289(27%), Positives:117/289(40%), Gaps:58/289(20%)

Query 96 YSANFTVGSNSQKQNVIVDTGSSDLWVVDSSANCQEKSGYSSDYCFSGGTYDPSSSSTIQ 155

Y TVGS Q N++VDTGSS+ V ++ + F Y SST +

Sbjct 18 YYVEMTVGSPPQTLNILVDTGSSNFAVG------------AAPHPFLHRYYQRQLSSTYR 65

Query 156 ELGKSFNIRYGDGSSSSGTWVKD--------------TVGINGAIILNQQFGDVNSTSVS 201

+L K + Y + G W + TV N A I +N ++

Sbjct 66 DLRKGVYVPY-----TQGKWEGELGTDLVSIPHGPNVTVRANIAAITESDKFFINGSN-W 119

Query 202 QGILGIGLDTNESTDTIYENFPINLKEQGFINTNAYSLYL----------NAPSATSGTI 251

+GILG+ D E F +L +Q + N +SL L ++ G++

Sbjct 120 EGILGLAYAEIARPDDSLEPFFDSLVKQTHV-PNLFSLQLCGAGFPLNQSEVLASVGGSM 178

Query 252 IFGGIDHAKYTGSLTTLPLTSNREFTIQTNSATVGTSTIDINTGL--------LLDSGTT 303

I GGIDH+ YTGSL P+ RE+ + V + D+ ++DSGTT

Sbjct 179 IIGGIDHSLYTGSLWYTPI--RREWYYEVIIVRVEINGQDLKMDCKEYNYDKSIVDSGTT 236

Query 304 LTYLPQSVVDSIANAI-GGDITYNRPIGAYIWS---CNRNGKVTYN-FP 347

LP+ V ++ +I T P G ++ C + G +N FP

Sbjct 237 NLRLPKKVFEAAVKSIKAASSTEKFPDGFWLGEQLVCWQAGTTPWNIFP 285

>BACE-1 in complex with a HEA-macrocyclic type inhibitor [Homo sapiens]

Sequence ID: 4DPF_A Length: 391

>BACE-1 in complex with HEA-macrocyclic inhibitor, MV078512 [Homo sapiens]

Sequence ID: 4DPI_A Length: 391 >BACE-1 in complex with HEA-type macrocyclic inhibitor, MV078571 [Homo sapiens]

Sequence ID: 4GMI_A Length: 391

Range 1: 20 to 287

Score:56.6 bits(135), Expect:1e-07,

Method:Compositional matrix adjust.,

Identities:77/289(27%), Positives:117/289(40%), Gaps:58/289(20%)

Query 96 YSANFTVGSNSQKQNVIVDTGSSDLWVVDSSANCQEKSGYSSDYCFSGGTYDPSSSSTIQ 155

Y TVGS Q N++VDTGSS+ V ++ + F Y SST +

Sbjct 20 YYVEMTVGSPPQTLNILVDTGSSNFAVG------------AAPHPFLHRYYQRQLSSTYR 67

Query 156 ELGKSFNIRYGDGSSSSGTWVKD--------------TVGINGAIILNQQFGDVNSTSVS 201

+L K + Y + G W + TV N A I +N ++

Sbjct 68 DLRKGVYVPY-----TQGKWEGELGTDLVSIPHGPNVTVRANIAAITESDKFFINGSN-W 121

Query 202 QGILGIGLDTNESTDTIYENFPINLKEQGFINTNAYSLYL----------NAPSATSGTI 251

+GILG+ D E F +L +Q + N +SL L ++ G++

Sbjct 122 EGILGLAYAEIARPDDSLEPFFDSLVKQTHV-PNLFSLQLCGAGFPLNQSEVLASVGGSM 180

Query 252 IFGGIDHAKYTGSLTTLPLTSNREFTIQTNSATVGTSTIDINTGL--------LLDSGTT 303

I GGIDH+ YTGSL P+ RE+ + V + D+ ++DSGTT

Sbjct 181 IIGGIDHSLYTGSLWYTPI--RREWYYEVIIVRVEINGQDLKMDCKEYNYDKSIVDSGTT 238

Query 304 LTYLPQSVVDSIANAI-GGDITYNRPIGAYIWS---CNRNGKVTYN-FP 347

LP+ V ++ +I T P G ++ C + G +N FP

Sbjct 239 NLRLPKKVFEAAVKSIKAASSTEKFPDGFWLGEQLVCWQAGTTPWNIFP 287

>Beta-secretase 1 complexed with statine-based inhibitor [Homo sapiens]

Sequence ID: 3DM6_A Length: 406

>Beta-secretase 1 complexed with statine-based inhibitor [Homo sapiens]

Sequence ID: 3DM6_B Length: 406 >Beta-secretase 1 complexed with statine-based inhibitor [Homo sapiens]

Sequence ID: 3DM6_C Length: 406 >Potent Beta-Secretase 1 hydroxyethylene Inhibitor [Homo sapiens]

Sequence ID: 3I25_A Length: 406 >Potent Beta-Secretase 1 hydroxyethylene Inhibitor [Homo sapiens]

Sequence ID: 3I25_B Length: 406 >Potent Beta-Secretase 1 hydroxyethylene Inhibitor [Homo sapiens]

Sequence ID: 3I25_C Length: 406

Range 1: 35 to 302

Score:56.6 bits(135), Expect:1e-07,

Method:Compositional matrix adjust.,

Identities:77/289(27%), Positives:117/289(40%), Gaps:58/289(20%)

Query 96 YSANFTVGSNSQKQNVIVDTGSSDLWVVDSSANCQEKSGYSSDYCFSGGTYDPSSSSTIQ 155

Y TVGS Q N++VDTGSS+ V ++ + F Y SST +

Sbjct 35 YYVEMTVGSPPQTLNILVDTGSSNFAVG------------AAPHPFLHRYYQRQLSSTYR 82

Query 156 ELGKSFNIRYGDGSSSSGTWVKD--------------TVGINGAIILNQQFGDVNSTSVS 201

+L K + Y + G W + TV N A I +N ++

Sbjct 83 DLRKGVYVPY-----TQGKWEGELGTDLVSIPHGPNVTVRANIAAITESDKFFINGSNW- 136

Query 202 QGILGIGLDTNESTDTIYENFPINLKEQGFINTNAYSLYL----------NAPSATSGTI 251

+GILG+ D E F +L +Q + N +SL L ++ G++

Sbjct 137 EGILGLAYAEIARPDDSLEPFFDSLVKQTHV-PNLFSLQLCGAGFPLNQSEVLASVGGSM 195

Query 252 IFGGIDHAKYTGSLTTLPLTSNREFTIQTNSATVGTSTIDINTGL--------LLDSGTT 303

I GGIDH+ YTGSL P+ RE+ + V + D+ ++DSGTT

Sbjct 196 IIGGIDHSLYTGSLWYTPI--RREWYYEVIIVRVEINGQDLKMDCKEYNYDKSIVDSGTT 253

Query 304 LTYLPQSVVDSIANAI-GGDITYNRPIGAYIWS---CNRNGKVTYN-FP 347

LP+ V ++ +I T P G ++ C + G +N FP

Sbjct 254 NLRLPKKVFEAAVKSIKAASSTEKFPDGFWLGEQLVCWQAGTTPWNIFP 302

>memapsin 2, partial [Homo sapiens]

Sequence ID: AAF13715.1 Length: 488

Range 1: 62 to 329

Score:56.6 bits(135), Expect:1e-07,

Method:Compositional matrix adjust.,

Identities:77/289(27%), Positives:117/289(40%), Gaps:58/289(20%)

Query 96 YSANFTVGSNSQKQNVIVDTGSSDLWVVDSSANCQEKSGYSSDYCFSGGTYDPSSSSTIQ 155

Y TVGS Q N++VDTGSS+ V ++ + F Y SST +

Sbjct 62 YYVEMTVGSPPQTLNILVDTGSSNFAVG------------AAPHPFLHRYYQRQLSSTYR 109

Query 156 ELGKSFNIRYGDGSSSSGTWVKD--------------TVGINGAIILNQQFGDVNSTSVS 201

+L K + Y + G W + TV N A I +N ++

Sbjct 110 DLRKGVYVPY-----TQGKWEGELGTDLVSIPHGPNVTVRANIAAITESDKFFINGSN-W 163

Query 202 QGILGIGLDTNESTDTIYENFPINLKEQGFINTNAYSLYLNAP----------SATSGTI 251

+GILG+ D E F +L +Q + N +SL L ++ G++

Sbjct 164 EGILGLAYAEIARPDDSLEPFFDSLVKQTHV-PNLFSLQLCGAGFPLNQSEVLASVGGSM 222

Query 252 IFGGIDHAKYTGSLTTLPLTSNREFTIQTNSATVGTSTIDINTGL--------LLDSGTT 303

I GGIDH+ YTGSL P+ RE+ + V + D+ ++DSGTT

Sbjct 223 IIGGIDHSLYTGSLWYTPI--RREWYYEVIIVRVEINGQDLKMDCKEYNYDKSIVDSGTT 280

Query 304 LTYLPQSVVDSIANAI-GGDITYNRPIGAYIWS---CNRNGKVTYN-FP 347

LP+ V ++ +I T P G ++ C + G +N FP

Sbjct 281 NLRLPKKVFEAAVKSIKAASSTEKFPDGFWLGEQLVCWQAGTTPWNIFP 329

>Crystal Structure of Unbound Beta-Secretase Catalytic Domain. [Homo sapiens]

Sequence ID: 1SGZ_A Length: 389

>Crystal Structure of Unbound Beta-Secretase Catalytic Domain. [Homo sapiens]

Sequence ID: 1SGZ_B Length: 389 >Crystal Structure of Unbound Beta-Secretase Catalytic Domain. [Homo sapiens]

Sequence ID: 1SGZ_C Length: 389 >Crystal Structure of Unbound Beta-Secretase Catalytic Domain. [Homo sapiens]

Sequence ID: 1SGZ_D Length: 389 >New substrate binding pockets for beta-secretase. [Homo sapiens]

Sequence ID: 1XN2_A Length: 389 >New substrate binding pockets for beta-secretase. [Homo sapiens]

Sequence ID: 1XN2_B Length: 389 >New substrate binding pockets for beta-secretase. [Homo sapiens]

Sequence ID: 1XN2_C Length: 389 >New substrate binding pockets for beta-secretase. [Homo sapiens]

Sequence ID: 1XN2_D Length: 389 >Crystal structure of Beta-secretase bound to a long inhibitor with additional upstream residues. [Homo sapiens]

Sequence ID: 1XN3_A Length: 389 >Crystal structure of Beta-secretase bound to a long inhibitor with additional upstream residues. [Homo sapiens]

Sequence ID: 1XN3_B Length: 389 >Crystal structure of Beta-secretase bound to a long inhibitor with additional upstream residues. [Homo sapiens]

Sequence ID: 1XN3_C Length: 389 >Crystal structure of Beta-secretase bound to a long inhibitor with additional upstream residues. [Homo sapiens]

Sequence ID: 1XN3_D Length: 389 >Crystal Structure of a cycloamide-urethane-derived novel inhibitor bound to human brain memapsin 2 (beta-secretase). [Homo sapiens]

Sequence ID: 1XS7_D Length: 389 >Crystal structure of beta-secretase bound to a potent and highly selective inhibitor. [Homo sapiens]

Sequence ID: 2G94_A Length: 389 >Crystal structure of beta-secretase bound to a potent and highly selective inhibitor. [Homo sapiens]

Sequence ID: 2G94_B Length: 389 >Crystal structure of beta-secretase bound to a potent and highly selective inhibitor. [Homo sapiens]

Sequence ID: 2G94_C Length: 389 >Crystal structure of beta-secretase bound to a potent and highly selective inhibitor. [Homo sapiens]

Sequence ID: 2G94_D Length: 389 >Crystal structure of beta-secretase bond to an inhibitor with Isophthalamide Derivatives at P2-P3 [Homo sapiens]

Sequence ID: 2P4J_A Length: 389 >Crystal structure of beta-secretase bond to an inhibitor with Isophthalamide Derivatives at P2-P3 [Homo sapiens]

Sequence ID: 2P4J_B Length: 389 >Crystal structure of beta-secretase bond to an inhibitor with Isophthalamide Derivatives at P2-P3 [Homo sapiens]

Sequence ID: 2P4J_C Length: 389 >Crystal structure of beta-secretase bond to an inhibitor with Isophthalamide Derivatives at P2-P3 [Homo sapiens]

Sequence ID: 2P4J_D Length: 389 >Crystal structure of GRL-8234 bound to BACE (Beta-secretase) [Homo sapiens]

Sequence ID: 2VKM_A Length: 389 >Crystal structure of GRL-8234 bound to BACE (Beta-secretase) [Homo sapiens]

Sequence ID: 2VKM_B Length: 389 >Crystal structure of GRL-8234 bound to BACE (Beta-secretase) [Homo sapiens]

Sequence ID: 2VKM_C Length: 389 >Crystal structure of GRL-8234 bound to BACE (Beta-secretase) [Homo sapiens]

Sequence ID: 2VKM_D Length: 389 >Discovery of Pyrrolidine-based b-Secretase Inhibitors: Lead Advancement through Conformational Design for Maintenance of Ligand Binding Efficiency [Homo sapiens]

Sequence ID: 3UFL_A Length: 389

Range 1: 18 to 285

Score:56.6 bits(135), Expect:1e-07,

Method:Compositional matrix adjust.,

Identities:77/289(27%), Positives:117/289(40%), Gaps:58/289(20%)

Query 96 YSANFTVGSNSQKQNVIVDTGSSDLWVVDSSANCQEKSGYSSDYCFSGGTYDPSSSSTIQ 155

Y TVGS Q N++VDTGSS+ V ++ + F Y SST +

Sbjct 18 YYVEMTVGSPPQTLNILVDTGSSNFAVG------------AAPHPFLHRYYQRQLSSTYR 65

Query 156 ELGKSFNIRYGDGSSSSGTWVKD--------------TVGINGAIILNQQFGDVNSTSVS 201

+L K + Y + G W + TV N A I +N ++

Sbjct 66 DLRKGVYVPY-----TQGKWEGELGTDLVSIPHGPNVTVRANIAAITESDKFFINGSN-W 119

Query 202 QGILGIGLDTNESTDTIYENFPINLKEQGFINTNAYSLYL----------NAPSATSGTI 251

+GILG+ D E F +L +Q + N +SL L ++ G++

Sbjct 120 EGILGLAYAEIARPDDSLEPFFDSLVKQTHV-PNLFSLQLCGAGFPLNQSEVLASVGGSM 178

Query 252 IFGGIDHAKYTGSLTTLPLTSNREFTIQTNSATVGTSTIDINTGL--------LLDSGTT 303

I GGIDH+ YTGSL P+ RE+ + V + D+ ++DSGTT

Sbjct 179 IIGGIDHSLYTGSLWYTPI--RREWYYEVIIVRVEINGQDLKMDCKEYNYDKSIVDSGTT 236

Query 304 LTYLPQSVVDSIANAI-GGDITYNRPIGAYIWS---CNRNGKVTYN-FP 347

LP+ V ++ +I T P G ++ C + G +N FP

Sbjct 237 NLRLPKKVFEAAVKSIKAASSTEKFPDGFWLGEQLVCWQAGTTPWNIFP 285

>Crystal Structure of Human BACE-1 in Complex with amino-1,4-oxazine compound 4 [Homo sapiens]

Sequence ID: 6FGY_A Length: 396

Range 1: 18 to 285

Score:56.6 bits(135), Expect:1e-07,

Method:Compositional matrix adjust.,

Identities:77/289(27%), Positives:117/289(40%), Gaps:58/289(20%)

Query 96 YSANFTVGSNSQKQNVIVDTGSSDLWVVDSSANCQEKSGYSSDYCFSGGTYDPSSSSTIQ 155

Y TVGS Q N++VDTGSS+ V ++ + F Y SST +

Sbjct 18 YYVEMTVGSPPQTLNILVDTGSSNFAVG------------AAPHPFLHRYYQRQLSSTYR 65

Query 156 ELGKSFNIRYGDGSSSSGTWVKD--------------TVGINGAIILNQQFGDVNSTSVS 201

+L K + Y + G W + TV N A I +N ++

Sbjct 66 DLRKGVYVPY-----TQGKWEGELGTDLVSIPHGPNVTVRANIAAITESDKFFINGSN-W 119

Query 202 QGILGIGLDTNESTDTIYENFPINLKEQGFINTNAYSLYL----------NAPSATSGTI 251

+GILG+ D E F +L +Q + N +SL L ++ G++

Sbjct 120 EGILGLAYAEIARPDDSLEPFFDSLVKQTHV-PNLFSLQLCGAGFPLNQSEVLASVGGSM 178

Query 252 IFGGIDHAKYTGSLTTLPLTSNREFTIQTNSATVGTSTIDINTGL--------LLDSGTT 303

I GGIDH+ YTGSL P+ RE+ + V + D+ ++DSGTT

Sbjct 179 IIGGIDHSLYTGSLWYTPI--RREWYYEVIIVRVEINGQDLKMDCKEYNYDKSIVDSGTT 236

Query 304 LTYLPQSVVDSIANAI-GGDITYNRPIGAYIWS---CNRNGKVTYN-FP 347

LP+ V ++ +I T P G ++ C + G +N FP

Sbjct 237 NLRLPKKVFEAAVKSIKAASSTEKFPDGFWLGEQLVCWQAGTTPWNIFP 285

>Structure of Beta-Secretase Complexed with Inhibitor [Homo sapiens]

Sequence ID: 1FKN_A Length: 391

>Structure of Beta-Secretase Complexed with Inhibitor [Homo sapiens]

Sequence ID: 1FKN_B Length: 391 >Crystal Structure of Beta-secretase complexed with Inhibitor OM00-3 [Homo sapiens]

Sequence ID: 1M4H_A Length: 391 >Crystal Structure of Beta-secretase complexed with Inhibitor OM00-3 [Homo sapiens]

Sequence ID: 1M4H_B Length: 391

Range 1: 20 to 287

Score:56.6 bits(135), Expect:1e-07,

Method:Compositional matrix adjust.,

Identities:77/289(27%), Positives:117/289(40%), Gaps:58/289(20%)

Query 96 YSANFTVGSNSQKQNVIVDTGSSDLWVVDSSANCQEKSGYSSDYCFSGGTYDPSSSSTIQ 155

Y TVGS Q N++VDTGSS+ V ++ + F Y SST +

Sbjct 20 YYVEMTVGSPPQTLNILVDTGSSNFAVG------------AAPHPFLHRYYQRQLSSTYR 67

Query 156 ELGKSFNIRYGDGSSSSGTWVKD--------------TVGINGAIILNQQFGDVNSTSVS 201

+L K + Y + G W + TV N A I +N ++

Sbjct 68 DLRKGVYVPY-----TQGKWEGELGTDLVSIPHGPNVTVRANIAAITESDKFFINGSN-W 121

Query 202 QGILGIGLDTNESTDTIYENFPINLKEQGFINTNAYSLYL----------NAPSATSGTI 251

+GILG+ D E F +L +Q + N +SL L ++ G++

Sbjct 122 EGILGLAYAEIARPDDSLEPFFDSLVKQTHV-PNLFSLQLCGAGFPLNQSEVLASVGGSM 180

Query 252 IFGGIDHAKYTGSLTTLPLTSNREFTIQTNSATVGTSTIDINTGL--------LLDSGTT 303

I GGIDH+ YTGSL P+ RE+ + V + D+ ++DSGTT

Sbjct 181 IIGGIDHSLYTGSLWYTPI--RREWYYEVIIVRVEINGQDLKMDCKEYNYDKSIVDSGTT 238

Query 304 LTYLPQSVVDSIANAI-GGDITYNRPIGAYIWS---CNRNGKVTYN-FP 347

LP+ V ++ +I T P G ++ C + G +N FP

Sbjct 239 NLRLPKKVFEAAVKSIKAASSTEKFPDGFWLGEQLVCWQAGTTPWNIFP 287

>Crystal structure of the human BACE1 catalytic domain in complex with N-(1-benzyl-piperidin-4-yl)-4-mercapto-butyramide [Homo sapiens]

Sequence ID: 2ZJH_A Length: 405

>Crystal structure of the human BACE1 catalytic domain in complex with N-[1-(5-bromo-2,3-dimethoxy-benzyl)-piperidin-4-yl]-4-mercapto-butyramide [Homo sapiens]

Sequence ID: 2ZJL_A Length: 405

Range 1: 34 to 301

Score:56.6 bits(135), Expect:2e-07,

Method:Compositional matrix adjust.,

Identities:77/289(27%), Positives:117/289(40%), Gaps:58/289(20%)

Query 96 YSANFTVGSNSQKQNVIVDTGSSDLWVVDSSANCQEKSGYSSDYCFSGGTYDPSSSSTIQ 155

Y TVGS Q N++VDTGSS+ V ++ + F Y SST +

Sbjct 34 YYVEMTVGSPPQTLNILVDTGSSNFAVG------------AAPHPFLHRYYQRQLSSTYR 81

Query 156 ELGKSFNIRYGDGSSSSGTWVKD--------------TVGINGAIILNQQFGDVNSTSVS 201

+L K + Y + G W + TV N A I +N ++

Sbjct 82 DLRKGVYVPY-----TQGKWEGELGTDLVSIPHGPNVTVRANIAAITESDKFFINGSNW- 135

Query 202 QGILGIGLDTNESTDTIYENFPINLKEQGFINTNAYSLYLNAP----------SATSGTI 251

+GILG+ D E F +L +Q + N +SL L ++ G++

Sbjct 136 EGILGLAYAEIARPDDSLEPFFDSLVKQTHV-PNLFSLQLCGAGFPLNQSEVLASVGGSM 194

Query 252 IFGGIDHAKYTGSLTTLPLTSNREFTIQTNSATVGTSTIDINTGL--------LLDSGTT 303

I GGIDH+ YTGSL P+ RE+ + V + D+ ++DSGTT

Sbjct 195 IIGGIDHSLYTGSLWYTPI--RREWYYEVIIVRVEINGQDLKMDCKEYNYDKSIVDSGTT 252

Query 304 LTYLPQSVVDSIANAI-GGDITYNRPIGAYIWS---CNRNGKVTYN-FP 347

LP+ V ++ +I T P G ++ C + G +N FP

Sbjct 253 NLRLPKKVFEAAVKSIKAASSTEKFPDGFWLGEQLVCWQAGTTPWNIFP 301

>Crystal structure of the human BACE1 catalytic domain in complex with N-[1-(5-chloro-2-isopropoxy-3-methoxy-benzyl)-piperidin-4-yl]-2-(2-methyl-4-sulfamoyl-phenoxy)-acetamide [Homo sapiens]

Sequence ID: 2ZJN_A Length: 405

>Discovery of aminoheterocycles as a novel beta-secretase inhibitor class [Homo sapiens]

Sequence ID: 3H0B_A Length: 405 >Discovery of aminoheterocycles as a novel beta-secretase inhibitor class [Homo sapiens]

Sequence ID: 3H0B_B Length: 405 >Discovery of aminoheterocycles as a novel beta-secretase inhibitor class [Homo sapiens]

Sequence ID: 3H0B_C Length: 405

Range 1: 34 to 301

Score:56.6 bits(135), Expect:2e-07,

Method:Compositional matrix adjust.,

Identities:77/289(27%), Positives:117/289(40%), Gaps:58/289(20%)

Query 96 YSANFTVGSNSQKQNVIVDTGSSDLWVVDSSANCQEKSGYSSDYCFSGGTYDPSSSSTIQ 155

Y TVGS Q N++VDTGSS+ V ++ + F Y SST +

Sbjct 34 YYVEMTVGSPPQTLNILVDTGSSNFAVG------------AAPHPFLHRYYQRQLSSTYR 81

Query 156 ELGKSFNIRYGDGSSSSGTWVKD--------------TVGINGAIILNQQFGDVNSTSVS 201

+L K + Y + G W + TV N A I +N ++

Sbjct 82 DLRKGVYVPY-----TQGKWEGELGTDLVSIPHGPNVTVRANIAAITESDKFFINGSNW- 135

Query 202 QGILGIGLDTNESTDTIYENFPINLKEQGFINTNAYSLYL----------NAPSATSGTI 251

+GILG+ D E F +L +Q + N +SL L ++ G++

Sbjct 136 EGILGLAYAEIARPDDSLEPFFDSLVKQTHV-PNLFSLQLCGAGFPLNQSEVLASVGGSM 194

Query 252 IFGGIDHAKYTGSLTTLPLTSNREFTIQTNSATVGTSTIDINTGL--------LLDSGTT 303

I GGIDH+ YTGSL P+ RE+ + V + D+ ++DSGTT

Sbjct 195 IIGGIDHSLYTGSLWYTPI--RREWYYEVIIVRVEINGQDLKMDCKEYNYDKSIVDSGTT 252

Query 304 LTYLPQSVVDSIANAI-GGDITYNRPIGAYIWS---CNRNGKVTYN-FP 347

LP+ V ++ +I T P G ++ C + G +N FP

Sbjct 253 NLRLPKKVFEAAVKSIKAASSTEKFPDGFWLGEQLVCWQAGTTPWNIFP 301

>Potent beta-secretase 1 inhibitor [Homo sapiens]

Sequence ID: 3IXK_A Length: 405

>Potent beta-secretase 1 inhibitor [Homo sapiens]

Sequence ID: 3IXK_B Length: 405 >Potent beta-secretase 1 inhibitor [Homo sapiens]

Sequence ID: 3IXK_C Length: 405 >Bace-1 in complex with a norstatine type inhibitor [Homo sapiens]

Sequence ID: 3KYR_A Length: 405 >Bace-1 in complex with a norstatine type inhibitor [Homo sapiens]

Sequence ID: 3KYR_B Length: 405 >Bace-1 in complex with a norstatine type inhibitor [Homo sapiens]

Sequence ID: 3KYR_C Length: 405

Range 1: 34 to 301

Score:56.6 bits(135), Expect:2e-07,

Method:Compositional matrix adjust.,

Identities:77/289(27%), Positives:117/289(40%), Gaps:58/289(20%)

Query 96 YSANFTVGSNSQKQNVIVDTGSSDLWVVDSSANCQEKSGYSSDYCFSGGTYDPSSSSTIQ 155

Y TVGS Q N++VDTGSS+ V ++ + F Y SST +

Sbjct 34 YYVEMTVGSPPQTLNILVDTGSSNFAVG------------AAPHPFLHRYYQRQLSSTYR 81

Query 156 ELGKSFNIRYGDGSSSSGTWVKD--------------TVGINGAIILNQQFGDVNSTSVS 201

+L K + Y + G W + TV N A I +N ++

Sbjct 82 DLRKGVYVPY-----TQGKWEGELGTDLVSIPHGPNVTVRANIAAITESDKFFINGSNW- 135

Query 202 QGILGIGLDTNESTDTIYENFPINLKEQGFINTNAYSLYL----------NAPSATSGTI 251

+GILG+ D E F +L +Q + N +SL L ++ G++

Sbjct 136 EGILGLAYAEIARPDDSLEPFFDSLVKQTHV-PNLFSLQLCGAGFPLNQSEVLASVGGSM 194

Query 252 IFGGIDHAKYTGSLTTLPLTSNREFTIQTNSATVGTSTIDINTGL--------LLDSGTT 303

I GGIDH+ YTGSL P+ RE+ + V + D+ ++DSGTT

Sbjct 195 IIGGIDHSLYTGSLWYTPI--RREWYYEVIIVRVEINGQDLKMDCKEYNYDKSIVDSGTT 252

Query 304 LTYLPQSVVDSIANAI-GGDITYNRPIGAYIWS---CNRNGKVTYN-FP 347

LP+ V ++ +I T P G ++ C + G +N FP

Sbjct 253 NLRLPKKVFEAAVKSIKAASSTEKFPDGFWLGEQLVCWQAGTTPWNIFP 301

>Crystal structure of human beta secretase complexed with NVP-AUR200 [Homo sapiens]

Sequence ID: 1YM2_A Length: 402

>Crystal structure of human beta secretase complexed with NVP-AUR200 [Homo sapiens]

Sequence ID: 1YM2_B Length: 402 >Crystal structure of human beta secretase complexed with NVP-AUR200 [Homo sapiens]

Sequence ID: 1YM2_C Length: 402 >Crystal Structure of the Bace complex with AXQ093, a macrocyclic inhibitor [Homo sapiens]

Sequence ID: 2F3E_A Length: 402 >Crystal Structure of the Bace complex with AXQ093, a macrocyclic inhibitor [Homo sapiens]

Sequence ID: 2F3E_B Length: 402 >Crystal Structure of the Bace complex with AXQ093, a macrocyclic inhibitor [Homo sapiens]

Sequence ID: 2F3E_C Length: 402 >Crystal Structure of the Bace complex with BDF488, a macrocyclic inhibitor [Homo sapiens]

Sequence ID: 2F3F_A Length: 402 >Crystal Structure of the Bace complex with BDF488, a macrocyclic inhibitor [Homo sapiens]

Sequence ID: 2F3F_B Length: 402 >Crystal Structure of the Bace complex with BDF488, a macrocyclic inhibitor [Homo sapiens]

Sequence ID: 2F3F_C Length: 402 >Crystal structure of human beta-secretase in complex with NVP-AFJ144 [Homo sapiens]

Sequence ID: 3DUY_A Length: 402 >Crystal structure of human beta-secretase in complex with NVP-AFJ144 [Homo sapiens]

Sequence ID: 3DUY_B Length: 402 >Crystal structure of human beta-secretase in complex with NVP-AFJ144 [Homo sapiens]

Sequence ID: 3DUY_C Length: 402 >Crystal structure of human beta-secretase in complex with NVP-ARV999 [Homo sapiens]

Sequence ID: 3DV1_A Length: 402 >Crystal structure of human beta-secretase in complex with NVP-ARV999 [Homo sapiens]

Sequence ID: 3DV1_B Length: 402 >Crystal structure of human beta-secretase in complex with NVP-ARV999 [Homo sapiens]

Sequence ID: 3DV1_C Length: 402 >Crystal structure of human beta-secretase in complex with NVP-BAV544 [Homo sapiens]

Sequence ID: 3DV5_A Length: 402 >Crystal structure of human beta-secretase in complex with NVP-BAV544 [Homo sapiens]

Sequence ID: 3DV5_B Length: 402 >Crystal structure of human beta-secretase in complex with NVP-BAV544 [Homo sapiens]

Sequence ID: 3DV5_C Length: 402 >Human BACE-1 complex with NB-216 [Homo sapiens]

Sequence ID: 3K5C_A Length: 402 >Human BACE-1 complex with NB-216 [Homo sapiens]

Sequence ID: 3K5C_B Length: 402 >Human BACE-1 complex with NB-216 [Homo sapiens]

Sequence ID: 3K5C_C Length: 402 >Human BACE-1 COMPLEX WITH AYH011 [Homo sapiens]

Sequence ID: 3K5F_A Length: 402 >Human BACE-1 COMPLEX WITH AYH011 [Homo sapiens]

Sequence ID: 3K5F_B Length: 402 >Human BACE-1 COMPLEX WITH AYH011 [Homo sapiens]

Sequence ID: 3K5F_C Length: 402 >Human bace-1 complex with bjc060 [Homo sapiens]

Sequence ID: 3K5G_A Length: 402 >Human bace-1 complex with bjc060 [Homo sapiens]

Sequence ID: 3K5G_B Length: 402 >Human bace-1 complex with bjc060 [Homo sapiens]

Sequence ID: 3K5G_C Length: 402 >Crystal Structure of Human Beta Secretase in Complex with BFG356 [Homo sapiens]

Sequence ID: 3PI5_A Length: 402 >Crystal Structure of Human Beta Secretase in Complex with BFG356 [Homo sapiens]

Sequence ID: 3PI5_B Length: 402 >Crystal Structure of Human Beta Secretase in Complex with BFG356 [Homo sapiens]

Sequence ID: 3PI5_C Length: 402 >Structure based design, synthesis and SAR of cyclic hydroxyethylamine (HEA) BACE-1 inhibitors [Homo sapiens]

Sequence ID: 3QBH_A Length: 402 >Structure based design, synthesis and SAR of cyclic hydroxyethylamine (HEA) BACE-1 inhibitors [Homo sapiens]

Sequence ID: 3QBH_B Length: 402 >Structure based design, synthesis and SAR of cyclic hydroxyethylamine (HEA) BACE-1 inhibitors [Homo sapiens]

Sequence ID: 3QBH_C Length: 402 >Crystal Structure of Human Beta Secretase in Complex with NVP-AVI326 [Homo sapiens]

Sequence ID: 3VEU_A Length: 402 >Crystal Structure of Human Beta Secretase in Complex with NVP-BQQ711 [Homo sapiens]

Sequence ID: 3VF3_A Length: 402 >Crystal Structure of Human Beta Secretase in Complex with NVP-BUR436, derived from a soaking experiment [Homo sapiens]

Sequence ID: 3VG1_A Length: 402 >Crystal Structure of Human Beta Secretase in Complex with NVP-BUR436, derived from a co-crystallization experiment [Homo sapiens]

Sequence ID: 4D83_A Length: 402 >Crystal Structure of Human Beta Secretase in Complex with NVP-BUR436, derived from a co-crystallization experiment [Homo sapiens]

Sequence ID: 4D83_B Length: 402 >Crystal Structure of Human Beta Secretase in Complex with NVP-BUR436, derived from a co-crystallization experiment [Homo sapiens]

Sequence ID: 4D83_C Length: 402 >Crystal Structure of Human Beta Secretase in Complex with NVP-BXQ490 [Homo sapiens]

Sequence ID: 4D88_A Length: 402 >Crystal Structure of Human Beta Secretase in Complex with NVP-BXD552, derived from a soaking experiment [Homo sapiens]

Sequence ID: 4D89_A Length: 402 >Crystal Structure of Human Beta Secretase in Complex with NVP-BXD552, derived from a co-crystallization experiment [Homo sapiens]

Sequence ID: 4D8C_A Length: 402 >Crystal Structure of Human Beta Secretase in Complex with NVP-BXD552, derived from a co-crystallization experiment [Homo sapiens]

Sequence ID: 4D8C_B Length: 402 >Crystal Structure of Human Beta Secretase in Complex with NVP-BXD552, derived from a co-crystallization experiment [Homo sapiens]

Sequence ID: 4D8C_C Length: 402 >Crystal Structure of Human Beta Secretase in Complex with Compound 11a [Homo sapiens]

Sequence ID: 4LXA_A Length: 402 >Crystal Structure of Human Beta Secretase in Complex with Compound 11a [Homo sapiens]

Sequence ID: 4LXA_B Length: 402 >Crystal Structure of Human Beta Secretase in Complex with Compound 11a [Homo sapiens]

Sequence ID: 4LXA_C Length: 402 >Crystal Structure of Human Beta Secretase in Complex with compound 11d [Homo sapiens]

Sequence ID: 4LXK_A Length: 402 >Crystal Structure of Human Beta Secretase in Complex with compound 11d [Homo sapiens]

Sequence ID: 4LXK_B Length: 402 >Crystal Structure of Human Beta Secretase in Complex with compound 11d [Homo sapiens]

Sequence ID: 4LXK_C Length: 402 >Crystal Structure of Human Beta Secretase in Complex with compound 12a [Homo sapiens]

Sequence ID: 4LXM_A Length: 402 >Crystal Structure of Human Beta Secretase in Complex with compound 12a [Homo sapiens]

Sequence ID: 4LXM_B Length: 402 >Crystal Structure of Human Beta Secretase in Complex with compound 12a [Homo sapiens]

Sequence ID: 4LXM_C Length: 402 >Crystal structure of BACE complex with BMC016 [Homo sapiens]

Sequence ID: 5QCO_A Length: 402 >Crystal structure of BACE complex with BMC016 [Homo sapiens]

Sequence ID: 5QCO_B Length: 402 >Crystal structure of BACE complex with BMC016 [Homo sapiens]

Sequence ID: 5QCO_C Length: 402 >Crystal structure of BACE complex with BMC018 [Homo sapiens]

Sequence ID: 5QCP_A Length: 402 >Crystal structure of BACE complex with BMC018 [Homo sapiens]

Sequence ID: 5QCP_B Length: 402 >Crystal structure of BACE complex with BMC018 [Homo sapiens]

Sequence ID: 5QCP_C Length: 402 >Crystal structure of BACE complex with BMC025 [Homo sapiens]

Sequence ID: 5QCQ_A Length: 402 >Crystal structure of BACE complex with BMC025 [Homo sapiens]

Sequence ID: 5QCQ_B Length: 402 >Crystal structure of BACE complex with BMC025 [Homo sapiens]

Sequence ID: 5QCQ_C Length: 402 >Crystal structure of BACE complex with BMC026 [Homo sapiens]

Sequence ID: 5QCR_A Length: 402 >Crystal structure of BACE complex with BMC026 [Homo sapiens]

Sequence ID: 5QCR_B Length: 402 >Crystal structure of BACE complex with BMC026 [Homo sapiens]

Sequence ID: 5QCR_C Length: 402 >Crystal structure of BACE complex with BMC024 [Homo sapiens]

Sequence ID: 5QCS_A Length: 402 >Crystal structure of BACE complex with BMC024 [Homo sapiens]

Sequence ID: 5QCS_B Length: 402 >Crystal structure of BACE complex with BMC024 [Homo sapiens]

Sequence ID: 5QCS_C Length: 402 >Crystal structure of BACE complex with BMC001 [Homo sapiens]

Sequence ID: 5QCT_A Length: 402 >Crystal structure of BACE complex with BMC001 [Homo sapiens]

Sequence ID: 5QCT_B Length: 402 >Crystal structure of BACE complex with BMC001 [Homo sapiens]

Sequence ID: 5QCT_C Length: 402 >Crystal structure of BACE complex with BMC022 [Homo sapiens]

Sequence ID: 5QCU_A Length: 402 >Crystal structure of BACE complex with BMC022 [Homo sapiens]

Sequence ID: 5QCU_B Length: 402 >Crystal structure of BACE complex with BMC022 [Homo sapiens]

Sequence ID: 5QCU_C Length: 402 >Crystal structure of BACE complex with BMC023 [Homo sapiens]

Sequence ID: 5QCV_A Length: 402 >Crystal structure of BACE complex with BMC023 [Homo sapiens]

Sequence ID: 5QCV_B Length: 402 >Crystal structure of BACE complex with BMC023 [Homo sapiens]

Sequence ID: 5QCV_C Length: 402 >Crystal structure of BACE complex with BMC021 [Homo sapiens]

Sequence ID: 5QCW_A Length: 402 >Crystal structure of BACE complex with BMC021 [Homo sapiens]

Sequence ID: 5QCW_B Length: 402 >Crystal structure of BACE complex with BMC021 [Homo sapiens]

Sequence ID: 5QCW_C Length: 402 >Crystal structure of BACE complex with BMC007 [Homo sapiens]

Sequence ID: 5QCX_A Length: 402 >Crystal structure of BACE complex with BMC007 [Homo sapiens]

Sequence ID: 5QCX_B Length: 402 >Crystal structure of BACE complex with BMC007 [Homo sapiens]

Sequence ID: 5QCX_C Length: 402 >Crystal structure of BACE complex with BMC008 [Homo sapiens]

Sequence ID: 5QCY_A Length: 402 >Crystal structure of BACE complex with BMC008 [Homo sapiens]

Sequence ID: 5QCY_B Length: 402 >Crystal structure of BACE complex with BMC008 [Homo sapiens]

Sequence ID: 5QCY_C Length: 402 >Crystal structure of BACE complex with BMC015 [Homo sapiens]

Sequence ID: 5QCZ_A Length: 402 >Crystal structure of BACE complex with BMC015 [Homo sapiens]

Sequence ID: 5QCZ_B Length: 402 >Crystal structure of BACE complex with BMC015 [Homo sapiens]

Sequence ID: 5QCZ_C Length: 402 >Crystal structure of BACE complex withBMC006 [Homo sapiens]

Sequence ID: 5QD0_A Length: 402 >Crystal structure of BACE complex withBMC006 [Homo sapiens]

Sequence ID: 5QD0_B Length: 402 >Crystal structure of BACE complex withBMC006 [Homo sapiens]

Sequence ID: 5QD0_C Length: 402 >Crystal structure of BACE complex with BMC011 [Homo sapiens]

Sequence ID: 5QD1_A Length: 402 >Crystal structure of BACE complex with BMC011 [Homo sapiens]

Sequence ID: 5QD1_B Length: 402 >Crystal structure of BACE complex with BMC011 [Homo sapiens]

Sequence ID: 5QD1_C Length: 402 >Crystal structure of BACE complex with BMC017 [Homo sapiens]

Sequence ID: 5QD2_A Length: 402 >Crystal structure of BACE complex with BMC017 [Homo sapiens]

Sequence ID: 5QD2_B Length: 402 >Crystal structure of BACE complex with BMC017 [Homo sapiens]

Sequence ID: 5QD2_C Length: 402 >Crystal structure of BACE complex with BMC010 [Homo sapiens]

Sequence ID: 5QD3_A Length: 402 >Crystal structure of BACE complex with BMC010 [Homo sapiens]

Sequence ID: 5QD3_B Length: 402 >Crystal structure of BACE complex with BMC010 [Homo sapiens]

Sequence ID: 5QD3_C Length: 402 >Crystal structure of BACE complex with BMC023 [Homo sapiens]

Sequence ID: 5QD4_A Length: 402 >Crystal structure of BACE complex with BMC023 [Homo sapiens]

Sequence ID: 5QD4_B Length: 402 >Crystal structure of BACE complex with BMC023 [Homo sapiens]

Sequence ID: 5QD4_C Length: 402 >Crystal structure of BACE complex with BMC009 [Homo sapiens]

Sequence ID: 5QD5_A Length: 402 >Crystal structure of BACE complex with BMC009 [Homo sapiens]

Sequence ID: 5QD5_B Length: 402 >Crystal structure of BACE complex with BMC009 [Homo sapiens]

Sequence ID: 5QD5_C Length: 402 >Crystal structure of BACE complex with BMC004 [Homo sapiens]

Sequence ID: 5QD6_A Length: 402 >Crystal structure of BACE complex with BMC004 [Homo sapiens]

Sequence ID: 5QD6_B Length: 402 >Crystal structure of BACE complex with BMC004 [Homo sapiens]

Sequence ID: 5QD6_C Length: 402 >Crystal structure of BACE complex with BMC014 [Homo sapiens]

Sequence ID: 5QD7_A Length: 402 >Crystal structure of BACE complex with BMC014 [Homo sapiens]

Sequence ID: 5QD7_B Length: 402 >Crystal structure of BACE complex with BMC014 [Homo sapiens]

Sequence ID: 5QD7_C Length: 402 >Crystal structure of BACE complex with BMC003 [Homo sapiens]

Sequence ID: 5QD8_A Length: 402 >Crystal structure of BACE complex with BMC003 [Homo sapiens]

Sequence ID: 5QD8_B Length: 402 >Crystal structure of BACE complex with BMC003 [Homo sapiens]

Sequence ID: 5QD8_C Length: 402 >Crystal structure of BACE complex with BMC005 [Homo sapiens]

Sequence ID: 5QD9_A Length: 402 >Crystal structure of BACE complex with BMC005 [Homo sapiens]

Sequence ID: 5QD9_B Length: 402 >Crystal structure of BACE complex with BMC005 [Homo sapiens]

Sequence ID: 5QD9_C Length: 402 >Crystal structure of BACE complex with BMC013 [Homo sapiens]

Sequence ID: 5QDA_A Length: 402 >Crystal structure of BACE complex with BMC013 [Homo sapiens]

Sequence ID: 5QDA_B Length: 402 >Crystal structure of BACE complex with BMC013 [Homo sapiens]

Sequence ID: 5QDA_C Length: 402 >Crystal structure of BACE complex with BMC002 [Homo sapiens]

Sequence ID: 5QDB_A Length: 402 >Crystal structure of BACE complex with BMC002 [Homo sapiens]

Sequence ID: 5QDB_B Length: 402 >Crystal structure of BACE complex with BMC002 [Homo sapiens]

Sequence ID: 5QDB_C Length: 402 >Crystal structure of BACE complex with BMC019 hydrolyzed [Homo sapiens]

Sequence ID: 5QDC_A Length: 402 >Crystal structure of BACE complex with BMC019 hydrolyzed [Homo sapiens]

Sequence ID: 5QDC_B Length: 402 >Crystal structure of BACE complex with BMC019 hydrolyzed [Homo sapiens]

Sequence ID: 5QDC_C Length: 402 >Crystal structure of BACE complex with BMC020 hydrolyzed [Homo sapiens]

Sequence ID: 5QDD_A Length: 402 >Crystal structure of BACE complex with BMC020 hydrolyzed [Homo sapiens]

Sequence ID: 5QDD_B Length: 402 >Crystal structure of BACE complex with BMC020 hydrolyzed [Homo sapiens]

Sequence ID: 5QDD_C Length: 402 >Crystal Structure of Human BACE-1 in Complex with CNP520 [Homo sapiens]

Sequence ID: 6EQM_A Length: 402

Range 1: 30 to 297

Score:56.6 bits(135), Expect:2e-07,

Method:Compositional matrix adjust.,

Identities:77/289(27%), Positives:117/289(40%), Gaps:58/289(20%)

Query 96 YSANFTVGSNSQKQNVIVDTGSSDLWVVDSSANCQEKSGYSSDYCFSGGTYDPSSSSTIQ 155

Y TVGS Q N++VDTGSS+ V ++ + F Y SST +

Sbjct 30 YYVEMTVGSPPQTLNILVDTGSSNFAVG------------AAPHPFLHRYYQRQLSSTYR 77

Query 156 ELGKSFNIRYGDGSSSSGTWVKD--------------TVGINGAIILNQQFGDVNSTSVS 201

+L K + Y + G W + TV N A I +N ++

Sbjct 78 DLRKGVYVPY-----TQGKWEGELGTDLVSIPHGPNVTVRANIAAITESDKFFINGSNW- 131

Query 202 QGILGIGLDTNESTDTIYENFPINLKEQGFINTNAYSLYLNAP----------SATSGTI 251

+GILG+ D E F +L +Q + N +SL L ++ G++

Sbjct 132 EGILGLAYAEIARPDDSLEPFFDSLVKQTHV-PNLFSLQLCGAGFPLNQSEVLASVGGSM 190

Query 252 IFGGIDHAKYTGSLTTLPLTSNREFTIQTNSATVGTSTIDINTGL--------LLDSGTT 303

I GGIDH+ YTGSL P+ RE+ + V + D+ ++DSGTT

Sbjct 191 IIGGIDHSLYTGSLWYTPI--RREWYYEVIIVRVEINGQDLKMDCKEYNYDKSIVDSGTT 248

Query 304 LTYLPQSVVDSIANAI-GGDITYNRPIGAYIWS---CNRNGKVTYN-FP 347

LP+ V ++ +I T P G ++ C + G +N FP

Sbjct 249 NLRLPKKVFEAAVKSIKAASSTEKFPDGFWLGEQLVCWQAGTTPWNIFP 297

>Rational Design and Synthesis of Aminopiperazinones as Beta Secretase (BACE) Inhibitors [Homo sapiens]

Sequence ID: 3U6A_A Length: 390

>Rational Design and Synthesis of Aminopiperazinones as Beta Secretase (BACE) Inhibitors [Homo sapiens]

Sequence ID: 3U6A_B Length: 390 >Rational Design and Synthesis of Aminopiperazinones as Beta Secretase (BACE) Inhibitors [Homo sapiens]

Sequence ID: 3U6A_C Length: 390

Range 1: 19 to 286

Score:56.6 bits(135), Expect:2e-07,

Method:Compositional matrix adjust.,

Identities:77/289(27%), Positives:117/289(40%), Gaps:58/289(20%)

Query 96 YSANFTVGSNSQKQNVIVDTGSSDLWVVDSSANCQEKSGYSSDYCFSGGTYDPSSSSTIQ 155

Y TVGS Q N++VDTGSS+ V ++ + F Y SST +

Sbjct 19 YYVEMTVGSPPQTLNILVDTGSSNFAVG------------AAPHPFLHRYYQRQLSSTYR 66

Query 156 ELGKSFNIRYGDGSSSSGTWVKD--------------TVGINGAIILNQQFGDVNSTSVS 201

+L K + Y + G W + TV N A I +N ++

Sbjct 67 DLRKGVYVPY-----TQGKWEGELGTDLVSIPHGPNVTVRANIAAITESDKFFINGSN-W 120

Query 202 QGILGIGLDTNESTDTIYENFPINLKEQGFINTNAYSLYL----------NAPSATSGTI 251

+GILG+ D E F +L +Q + N +SL L ++ G++

Sbjct 121 EGILGLAYAEIARPDDSLEPFFDSLVKQTHV-PNLFSLQLCGAGFPLNQSEVLASVGGSM 179

Query 252 IFGGIDHAKYTGSLTTLPLTSNREFTIQTNSATVGTSTIDINTGL--------LLDSGTT 303

I GGIDH+ YTGSL P+ RE+ + V + D+ ++DSGTT

Sbjct 180 IIGGIDHSLYTGSLWYTPI--RREWYYEVIIVRVEINGQDLKMDCKEYNYDKSIVDSGTT 237

Query 304 LTYLPQSVVDSIANAI-GGDITYNRPIGAYIWS---CNRNGKVTYN-FP 347

LP+ V ++ +I T P G ++ C + G +N FP

Sbjct 238 NLRLPKKVFEAAVKSIKAASSTEKFPDGFWLGEQLVCWQAGTTPWNIFP 286

>human Bace (beta secretase) in complex with Cyclohexanecarboxylic acid (2-(2-am ino-6-phenoxy-4H-quinazolin-3-yl)-2 -cyclohexyl-ethyl)- amide [Homo sapiens]

Sequence ID: 2WJO_A Length: 412

Range 1: 18 to 285

Score:56.6 bits(135), Expect:2e-07,

Method:Compositional matrix adjust.,

Identities:77/289(27%), Positives:117/289(40%), Gaps:58/289(20%)

Query 96 YSANFTVGSNSQKQNVIVDTGSSDLWVVDSSANCQEKSGYSSDYCFSGGTYDPSSSSTIQ 155

Y TVGS Q N++VDTGSS+ V ++ + F Y SST +

Sbjct 18 YYVEMTVGSPPQTLNILVDTGSSNFAVG------------AAPHPFLHRYYQRQLSSTYR 65

Query 156 ELGKSFNIRYGDGSSSSGTWVKD--------------TVGINGAIILNQQFGDVNSTSVS 201

+L K + Y + G W + TV N A I +N ++

Sbjct 66 DLRKGVYVPY-----TQGKWEGELGTDLVSIPHGPNVTVRANIAAITESDKFFINGSN-W 119

Query 202 QGILGIGLDTNESTDTIYENFPINLKEQGFINTNAYSLYL----------NAPSATSGTI 251

+GILG+ D E F +L +Q + N +SL L ++ G++

Sbjct 120 EGILGLAYAEIARPDDSLEPFFDSLVKQTHV-PNLFSLQLCGAGFPLNQSEVLASVGGSM 178

Query 252 IFGGIDHAKYTGSLTTLPLTSNREFTIQTNSATVGTSTIDINTGL--------LLDSGTT 303

I GGIDH+ YTGSL P+ RE+ + V + D+ ++DSGTT

Sbjct 179 IIGGIDHSLYTGSLWYTPI--RREWYYEVIIVRVEINGQDLKMDCKEYNYDKSIVDSGTT 236

Query 304 LTYLPQSVVDSIANAI-GGDITYNRPIGAYIWS---CNRNGKVTYN-FP 347

LP+ V ++ +I T P G ++ C + G +N FP

Sbjct 237 NLRLPKKVFEAAVKSIKAASSTEKFPDGFWLGEQLVCWQAGTTPWNIFP 285

Range 2: 18 to 285

Score:56.6 bits(135), Expect:2e-07,

Method:Compositional matrix adjust.,

Identities:77/289(27%), Positives:117/289(40%), Gaps:58/289(20%)

Query 96 YSANFTVGSNSQKQNVIVDTGSSDLWVVDSSANCQEKSGYSSDYCFSGGTYDPSSSSTIQ 155

Y TVGS Q N++VDTGSS+ V ++ + F Y SST +

Sbjct 18 YYVEMTVGSPPQTLNILVDTGSSNFAVG------------AAPHPFLHRYYQRQLSSTYR 65

Query 156 ELGKSFNIRYGDGSSSSGTWVKD--------------TVGINGAIILNQQFGDVNSTSVS 201

+L K + Y + G W + TV N A I +N ++

Sbjct 66 DLRKGVYVPY-----TQGKWEGELGTDLVSIPHGPNVTVRANIAAITESDKFFINGSN-W 119

Query 202 QGILGIGLDTNESTDTIYENFPINLKEQGFINTNAYSLYL----------NAPSATSGTI 251

+GILG+ D E F +L +Q + N +SL L ++ G++

Sbjct 120 EGILGLAYAEIARPDDSLEPFFDSLVKQTHV-PNLFSLQLCGAGFPLNQSEVLASVGGSM 178

Query 252 IFGGIDHAKYTGSLTTLPLTSNREFTIQTNSATVGTSTIDINTGL--------LLDSGTT 303

I GGIDH+ YTGSL P+ RE+ + V + D+ ++DSGTT

Sbjct 179 IIGGIDHSLYTGSLWYTPI--RREWYYEVIIVRVEINGQDLKMDCKEYNYDKSIVDSGTT 236

Query 304 LTYLPQSVVDSIANAI-GGDITYNRPIGAYIWS---CNRNGKVTYN-FP 347

LP+ V ++ +I T P G ++ C + G +N FP

Sbjct 237 NLRLPKKVFEAAVKSIKAASSTEKFPDGFWLGEQLVCWQAGTTPWNIFP 285

>co-crystal structure of BACE with inhibitor AM-6494 [Homo sapiens]

Sequence ID: 6PZ4_A Length: 411

Range 1: 33 to 300

Score:56.6 bits(135), Expect:2e-07,

Method:Compositional matrix adjust.,

Identities:77/289(27%), Positives:117/289(40%), Gaps:58/289(20%)

Query 96 YSANFTVGSNSQKQNVIVDTGSSDLWVVDSSANCQEKSGYSSDYCFSGGTYDPSSSSTIQ 155

Y TVGS Q N++VDTGSS+ V ++ + F Y SST +

Sbjct 33 YYVEMTVGSPPQTLNILVDTGSSNFAVG------------AAPHPFLHRYYQRQLSSTYR 80

Query 156 ELGKSFNIRYGDGSSSSGTWVKD--------------TVGINGAIILNQQFGDVNSTSVS 201

+L K + Y + G W + TV N A I +N ++

Sbjct 81 DLRKGVYVPY-----TQGKWEGELGTDLVSIPHGPNVTVRANIAAITESDKFFINGSNW- 134

Query 202 QGILGIGLDTNESTDTIYENFPINLKEQGFINTNAYSLYL----------NAPSATSGTI 251

+GILG+ D E F +L +Q + N +SL L ++ G++

Sbjct 135 EGILGLAYAEIARPDDSLEPFFDSLVKQTHV-PNLFSLQLCGAGFPLNQSEVLASVGGSM 193

Query 252 IFGGIDHAKYTGSLTTLPLTSNREFTIQTNSATVGTSTIDINTGL--------LLDSGTT 303

I GGIDH+ YTGSL P+ RE+ + V + D+ ++DSGTT

Sbjct 194 IIGGIDHSLYTGSLWYTPI--RREWYYEVIIVRVEINGQDLKMDCKEYNYDKSIVDSGTT 251

Query 304 LTYLPQSVVDSIANAI-GGDITYNRPIGAYIWS---CNRNGKVTYN-FP 347

LP+ V ++ +I T P G ++ C + G +N FP

Sbjct 252 NLRLPKKVFEAAVKSIKAASSTEKFPDGFWLGEQLVCWQAGTTPWNIFP 300

>Crystal Structure of BACE with Compound 1 [Homo sapiens]

Sequence ID: 3UDH_A Length: 404

>Crystal Structure of BACE with Compound 5 [Homo sapiens]

Sequence ID: 3UDJ_A Length: 404 >Crystal Structure of BACE with Compound 6 [Homo sapiens]

Sequence ID: 3UDK_A Length: 404 >Crystal Structure of BACE with Compound 8 [Homo sapiens]

Sequence ID: 3UDM_A Length: 404 >Crystal Structure of BACE with Compound 9 [Homo sapiens]

Sequence ID: 3UDN_A Length: 404 >Crystal Structure of BACE with Compound 12 [Homo sapiens]

Sequence ID: 3UDP_A Length: 404 >Crystal Structure of BACE with Compound 13 [Homo sapiens]

Sequence ID: 3UDQ_A Length: 404 >Crystal Structure of BACE with Compound 14 [Homo sapiens]

Sequence ID: 3UDR_A Length: 404 >Crystal Structure of BACE with Compound 11 [Homo sapiens]

Sequence ID: 3UDY_A Length: 404 >Crystal Structure of BACE with Compound 14g [Homo sapiens]

Sequence ID: 4FM7_A Length: 404 >Crystal Structure of BACE with Compound 12a [Homo sapiens]

Sequence ID: 4FM8_A Length: 404 >Crystal structure of human BACE-1 bound to Compound 24B [Homo sapiens]

Sequence ID: 4WY1_A Length: 404

Range 1: 18 to 285

Score:56.2 bits(134), Expect:2e-07,

Method:Compositional matrix adjust.,

Identities:77/289(27%), Positives:117/289(40%), Gaps:58/289(20%)

Query 96 YSANFTVGSNSQKQNVIVDTGSSDLWVVDSSANCQEKSGYSSDYCFSGGTYDPSSSSTIQ 155

Y TVGS Q N++VDTGSS+ V ++ + F Y SST +

Sbjct 18 YYVEMTVGSPPQTLNILVDTGSSNFAVG------------AAPHPFLHRYYQRQLSSTYR 65

Query 156 ELGKSFNIRYGDGSSSSGTWVKD--------------TVGINGAIILNQQFGDVNSTSVS 201

+L K + Y + G W + TV N A I +N ++

Sbjct 66 DLRKGVYVPY-----TQGKWEGELGTDLVSIPHGPNVTVRANIAAITESDKFFINGSNW- 119

Query 202 QGILGIGLDTNESTDTIYENFPINLKEQGFINTNAYSLYL----------NAPSATSGTI 251

+GILG+ D E F +L +Q + N +SL L ++ G++

Sbjct 120 EGILGLAYAEIARPDDSLEPFFDSLVKQTHV-PNLFSLQLCGAGFPLNQSEVLASVGGSM 178

Query 252 IFGGIDHAKYTGSLTTLPLTSNREFTIQTNSATVGTSTIDINTGL--------LLDSGTT 303

I GGIDH+ YTGSL P+ RE+ + V + D+ ++DSGTT

Sbjct 179 IIGGIDHSLYTGSLWYTPI--RREWYYEVIIVRVEINGQDLKMDCKEYNYDKSIVDSGTT 236

Query 304 LTYLPQSVVDSIANAI-GGDITYNRPIGAYIWS---CNRNGKVTYN-FP 347

LP+ V ++ +I T P G ++ C + G +N FP

Sbjct 237 NLRLPKKVFEAAVKSIKAASSTEKFPDGFWLGEQLVCWQAGTTPWNIFP 285

>Crystal structure of the human BACE1 catalytic domain in complex with 4-(4-fluoro-benzyl)-piperazine-2-carboxylic acid(3-mercapto-propyl)-amide [Homo sapiens]

Sequence ID: 2ZJK_A Length: 405

>Crystal structure of the human BACE1 catalytic domain in complex with 4-(4-fluoro-benzyl)-piperazine-2-carboxylic acid(3-mercapto-propyl)-amide [Homo sapiens]

Sequence ID: 2ZJK_B Length: 405 >Crystal structure of the human BACE1 catalytic domain in complex with 4-(4-fluoro-benzyl)-piperazine-2-carboxylic acid(3-mercapto-propyl)-amide [Homo sapiens]

Sequence ID: 2ZJK_C Length: 405

Range 1: 34 to 301

Score:56.2 bits(134), Expect:2e-07,

Method:Compositional matrix adjust.,

Identities:78/285(27%), Positives:116/285(40%), Gaps:50/285(17%)

Query 96 YSANFTVGSNSQKQNVIVDTGSSDLWVVDSSANCQEKSGYSSDYCFSGGTYDPSSSSTIQ 155

Y TVGS Q N++VDTGSS+ V ++ + F Y SST +

Sbjct 34 YYVEMTVGSPPQTLNILVDTGSSNFAVG------------AAPHPFLHRYYQRQLSSTYR 81

Query 156 ELGKSFNIRYGDGSSSSGTWVKDTVGI----------NGAIILNQQFGDVNSTSVSQGIL 205

+L K + Y G G D V I N A I +N ++ +GIL

Sbjct 82 DLRKGVYVPYCQGKWE-GELGTDLVSIPHGPNVTVRANIAAITESDKFFINGSNW-EGIL 139

Query 206 GIGLDTNESTDTIYENFPINLKEQGFINTNAYSLYL----------NAPSATSGTIIFGG 255

G+ D E F +L +Q + N +SL L ++ G++I GG

Sbjct 140 GLAYAEIARPDDSLEPFFDSLVKQTHV-PNLFSLQLCGAGFPLNQSEVLASVGGSMIIGG 198

Query 256 IDHAKYTGSLTTLPLTSNREFTIQTNSATVGTSTIDINTGL--------LLDSGTTLTYL 307

IDH+ YTGSL P+ RE+ + V + D+ ++DSGTT L

Sbjct 199 IDHSLYTGSLWYTPI--RREWYYEVIIVRVEINGQDLKMDCKEYNYDKSIVDSGTTNLRL 256

Query 308 PQSVVDSIANAI-GGDITYNRPIGAYIWS---CNRNGKVTYN-FP 347

P+ V ++ +I T P G ++ C + G +N FP

Sbjct 257 PKKVFEAAVKSIKAASSTEKFPDGFWLGEQLVCWQAGTTPWNIFP 301

>CRYSTAL STRUCTURE OF BACE-1 IN COMPLEX WITH {(1R,2R)-2-[(R)-2-Amino-4-(4-difluoromethoxy-phenyl)-4,5-dihydro-oxazol-4-yl]-cyclopropyl}-(5-chloro-pyridin-3-yl)-methanone [Homo sapiens]

Sequence ID: 5EZX_A Length: 390

>CRYSTAL STRUCTURE OF BACE-1 IN COMPLEX WITH (4S)-4-[3-(5-chloro-3-pyridyl)phenyl]-4-[4-(difluoromethoxy)-3-methyl-phenyl]-5H-oxazol-2-amine [Homo sapiens]

Sequence ID: 5EZZ_A Length: 390 >CRYSTAL STRUCTURE OF BACE-1 IN COMPLEX WITH 5-[3-[(3-chloro-8-quinolyl)amino]phenyl]-5-methyl-2,6-dihydro-1,4-oxazin-3-amine [Homo sapiens]

Sequence ID: 5F00_A Length: 390 >CRYSTAL STRUCTURE OF BACE-1 IN COMPLEX WITH (1SR,2SR)-2-((R)-2-amino-5,5-difluoro-4-methyl-5,6-dihydro-4H-1,3-oxazin-4-yl)-N-(3-chloroquinolin-8-yl)cyclopropanecarboxamide [Homo sapiens]

Sequence ID: 5F01_A Length: 390 >A multiconformer ligand model of 5T5 bound to BACE-1 [Homo sapiens]

Sequence ID: 6DMI_A Length: 390

Range 1: 19 to 286

Score:56.2 bits(134), Expect:2e-07,

Method:Compositional matrix adjust.,

Identities:77/289(27%), Positives:118/289(40%), Gaps:58/289(20%)

Query 96 YSANFTVGSNSQKQNVIVDTGSSDLWVVDSSANCQEKSGYSSDYCFSGGTYDPSSSSTIQ 155

Y TVGS Q N++VDTGSS+ V ++ + F Y SST +

Sbjct 19 YYVEMTVGSPPQTLNILVDTGSSNFAVG------------AAPHPFLHRYYQRQLSSTYR 66

Query 156 ELGKSFNIRYGDGSSSSGTWVKD--------------TVGINGAIILNQQFGDVNSTSVS 201

+L K + Y + G W + TV N A I +N ++

Sbjct 67 DLRKGVYVPY-----TQGKWEGELGTDLVSIPHGPNVTVRANIAAITESDKFFINGSN-W 120

Query 202 QGILGIGLDTNESTDTIYENFPINLKEQGFINTNAYSLYL----------NAPSATSGTI 251

+GILG+ D E F +L +Q + N +SL L ++ G++

Sbjct 121 EGILGLAYAEIARPDDSLEPFFDSLVKQTHV-PNLFSLQLCGAGFPLNQSEVLASVGGSM 179

Query 252 IFGGIDHAKYTGSLTTLPLTSNREFTIQTNSATVGTSTIDINTGL--------LLDSGTT 303

I GGIDH+ YTGSL P+ RE+ + V + D+ ++DSGTT

Sbjct 180 IIGGIDHSLYTGSLWYTPI--RREWYYEVIIVRVEINGQDLKMDCKEYNYDKSIVDSGTT 237

Query 304 LTYLPQSVVD-SIANAIGGDITYNRPIGAYIWS---CNRNGKVTYN-FP 347

LP+ V + ++A+ T P G ++ C + G +N FP

Sbjct 238 NLRLPKKVFEAAVASIKAASSTEKFPDGFWLGEQLVCWQAGTTPWNIFP 286

>Apo Structure of BACE (Beta Secretase) [Homo sapiens]

Sequence ID: 1W50_A Length: 411

>BACE (Beta Secretase) in complex with a nanomolar non-peptidic inhibitor [Homo sapiens]

Sequence ID: 1W51_A Length: 411 >Structure of Bace-1 (Beta-Secretase) in complex with (R)-3-(2-amino-6-o-tolylquinolin-3-yl)-N-((R)-2,2-dimethyltetrahydro-2H-pyran-4-yl)-2-methylpropanamide [Homo sapiens]

Sequence ID: 3RSV_A Length: 411 >Structure of Bace-1 (Beta-Secretase) in Complex with 6-(Thiophen-3-yl)quinolin-2-amine [Homo sapiens]

Sequence ID: 3RSX_A Length: 411 >Structure of Bace-1 (Beta-Secretase) in Complex with 6-(2-(3,3-Dimethylbut-1-ynyl)phenyl)quinolin-2-amine [Homo sapiens]

Sequence ID: 3RTH_A Length: 411 >Structure of Bace-1 (Beta-Secretase) in Complex with 3-(2-Aminoquinolin-3-yl)-N-cyclohexyl-N-methylpropanamide [Homo sapiens]

Sequence ID: 3RTM_A Length: 411 >Structure of Bace-1 (Beta-Secretase) in Complex with 3-(2-Amino-6-o-tolylquinolin-3-yl)-N-cyclohexylpropanamide [Homo sapiens]

Sequence ID: 3RTN_A Length: 411 >Structure of Bace-1 (Beta-Secretase) in Complex with 3-(2-Aminoquinolin-3-yl)-N-(cyclohexylmethyl)propanamide [Homo sapiens]

Sequence ID: 3RU1_A Length: 411 >Structure of Bace-1 (Beta-Secretase) in Complex with 2-((2-Amino-6-o-tolylquinolin-3-yl)methyl)-N-(cyclohexylmethyl)pentanamide [Homo sapiens]

Sequence ID: 3RVI_A Length: 411 >Aminoimidazoles as BACE-1 Inhibitors. X-RAY CRYSTAL STRUCTURE OF BETA SECRETASE COMPLEXED WITH COMPOUND 14 [Homo sapiens]

Sequence ID: 4ACU_A Length: 411 >Aminoimidazoles as BACE-1 Inhibitors. X-RAY CRYSTAL STRUCTURE OF BETA SECRETASE COMPLEXED WITH COMPOUND 23 [Homo sapiens]

Sequence ID: 4ACX_A Length: 411 >Design and Synthesis of BACE1 Inhibitors with In Vivo Brain Reduction of beta-Amyloid Peptides (COMPOUND 10) [Homo sapiens]

Sequence ID: 4AZY_A Length: 411 >Design and Synthesis of BACE1 Inhibitors with In Vivo Brain Reduction of beta-Amyloid Peptides (COMPOUND (R)-41) [Homo sapiens]

Sequence ID: 4B00_A Length: 411 >Preclinical characterization of AZD3839, a novel clinical candidate BACE1 inhibitor for the treatment of Alzheimer Disease [Homo sapiens]

Sequence ID: 4B05_A Length: 411 >Structure of Bace-1 (Beta-Secretase) in Complex with (2R)-N-((2S,3R)-1-(benzo[d][1,3]dioxol-5-yl)-3-hydroxy-4-((S)-6'-neopentyl-3',4'-dihydrospiro[cyclobutane-1,2'-pyrano[2,3-b]pyridine]-4'-ylamino)butan-2-yl)-2-methoxypropanamide [Homo sapiens]

Sequence ID: 4DH6_A Length: 411 >Crystal structure of BACE1 in complex with hydroxyethylamine inhibitor 37 [Homo sapiens]

Sequence ID: 4DI2_A Length: 411 >Crystal structure of BACE1 in complex with hydroxyethylamine inhibitor 37 [Homo sapiens]

Sequence ID: 4DI2_B Length: 411 >Crystal structure of BACE1 in complex with hydroxyethylamine inhibitor 37 [Homo sapiens]

Sequence ID: 4DI2_C Length: 411 >Structure of Bace-1 (Beta-Secretase) in complex with N-((2S,3R)-1-(4-fluorophenyl)-3-hydroxy-4-((6'-neopentyl-3',4'-dihydrospiro[cyclobutane-1,2'-pyrano[2,3-b]pyridin]-4'-yl)amino)butan-2-yl)acetamide [Homo sapiens]

Sequence ID: 4DUS_A Length: 411 >Crystal structure of BACE1 in complex with biarylspiro aminooxazoline 6 [Homo sapiens]

Sequence ID: 4FRI_A Length: 411 >Crystal structure of BACE1 in complex with aminooxazoline xanthene 9l [Homo sapiens]

Sequence ID: 4FRJ_A Length: 411 >Crystal structure of BACE1 in complex with aminooxazoline xanthene 11a [Homo sapiens]

Sequence ID: 4FRK_A Length: 411 >Crystal structure of BACE1 in complex with hydroxyethylamine-macrocyclic inhibitor 13 [Homo sapiens]

Sequence ID: 4KE0_A Length: 411 >Crystal structure of BACE1 in complex with hydroxyethylamine-macrocyclic inhibitor 13 [Homo sapiens]

Sequence ID: 4KE0_B Length: 411 >Crystal structure of BACE1 in complex with hydroxyethylamine-macrocyclic inhibitor 13 [Homo sapiens]

Sequence ID: 4KE0_C Length: 411 >Crystal structure of BACE1 in complex with hydroxyethylamine-macrocyclic inhibitor 19 [Homo sapiens]

Sequence ID: 4KE1_A Length: 411 >Crystal structure of BACE1 in complex with a 2-aminooxazoline 4-azaxanthene inhibitor [Homo sapiens]

Sequence ID: 4RCD_A Length: 411 >Crystal structure of BACE1 in complex with aminooxazoline xanthene inhibitor 2 [Homo sapiens]

Sequence ID: 4RCE_A Length: 411 >Crystal structure of BACE1 in complex with 2-aminooxazoline 4-fluoroxanthene inhibitor 49 [Homo sapiens]

Sequence ID: 4RCF_A Length: 411 >Crystal structure of BACE1 in complex with 2-aminooxazoline 3-aza-4-fluoro-xanthene inhibitor 22 [Homo sapiens]

Sequence ID: 4WTU_A Length: 411 >Crystal structure of BACE1 in complex with 2-aminooxazoline 3-azaxanthene inhibitor 28 [Homo sapiens]

Sequence ID: 4XKX_A Length: 411 >Crystal structure of BACE1 in complex with aminoquinoline compound 1 [Homo sapiens]

Sequence ID: 5I3V_A Length: 411 >Crystal structure of BACE1 in complex with 2-aminooxazoline-3-azaxanthene inhibitor 2 [Homo sapiens]

Sequence ID: 5I3W_A Length: 411 >Crystal structure of BACE1 in complex with aminoquinoline inhibitor 6 [Homo sapiens]

Sequence ID: 5I3X_A Length: 411 >Crystal structure of BACE1 in complex with aminoquinoline inhibitor 9 [Homo sapiens]

Sequence ID: 5I3Y_A Length: 411 >Crystal structure of BACE1 in complex with 3-(2-amino-6-(o-tolyl)quinolin-3-yl)-N-(3,3-dimethylbutyl)propanamide [Homo sapiens]

Sequence ID: 5IE1_A Length: 411 >Crystal structure of BACE1 in complex with 2-aminooxazoline-3-azaxanthene compound 12 [Homo sapiens]

Sequence ID: 5UYU_A Length: 411 >Structure of Bace-1 (Beta-Secretase) in complex with : N-(3-((1R,5S,6R)-3-amino-5-methyl-2-oxa-4-azabicyclo[4.1.0]hept-3-en-5-yl)-4-fluorophenyl)-5-methoxypyrazine-2-carboxamide [Homo sapiens]

Sequence ID: 6C2I_A Length: 411 >Crystal structure of BACE1 in complex with (Z)-fluoro-olefin containing compound 15 [Homo sapiens]

Sequence ID: 6WNY_A Length: 411

Range 1: 33 to 300

Score:56.2 bits(134), Expect:2e-07,

Method:Compositional matrix adjust.,

Identities:77/289(27%), Positives:117/289(40%), Gaps:58/289(20%)

Query 96 YSANFTVGSNSQKQNVIVDTGSSDLWVVDSSANCQEKSGYSSDYCFSGGTYDPSSSSTIQ 155

Y TVGS Q N++VDTGSS+ V ++ + F Y SST +

Sbjct 33 YYVEMTVGSPPQTLNILVDTGSSNFAVG------------AAPHPFLHRYYQRQLSSTYR 80

Query 156 ELGKSFNIRYGDGSSSSGTWVKD--------------TVGINGAIILNQQFGDVNSTSVS 201

+L K + Y + G W + TV N A I +N ++

Sbjct 81 DLRKGVYVPY-----TQGKWEGELGTDLVSIPHGPNVTVRANIAAITESDKFFINGSNW- 134

Query 202 QGILGIGLDTNESTDTIYENFPINLKEQGFINTNAYSLYL----------NAPSATSGTI 251

+GILG+ D E F +L +Q + N +SL L ++ G++

Sbjct 135 EGILGLAYAEIARPDDSLEPFFDSLVKQTHV-PNLFSLQLCGAGFPLNQSEVLASVGGSM 193

Query 252 IFGGIDHAKYTGSLTTLPLTSNREFTIQTNSATVGTSTIDINTGL--------LLDSGTT 303

I GGIDH+ YTGSL P+ RE+ + V + D+ ++DSGTT

Sbjct 194 IIGGIDHSLYTGSLWYTPI--RREWYYEVIIVRVEINGQDLKMDCKEYNYDKSIVDSGTT 251

Query 304 LTYLPQSVVDSIANAI-GGDITYNRPIGAYIWS---CNRNGKVTYN-FP 347

LP+ V ++ +I T P G ++ C + G +N FP

Sbjct 252 NLRLPKKVFEAAVKSIKAASSTEKFPDGFWLGEQLVCWQAGTTPWNIFP 300

>Crystal Structure of human beta-secretase (BACE) in the presence of an inhibitor (2) [Homo sapiens]

Sequence ID: 2HM1_A Length: 406

>Crystal Structure of Hydroxyethyl Secondary Amine-based Peptidomimetic Inhibitor of Human Beta-Secretase (BACE) [Homo sapiens]

Sequence ID: 2IQG_A Length: 406 >Design and Synthesis of Potent BACE-1 Inhibitors with Cellular Activity: Structure-Activity Relationship of P1 Substituents [Homo sapiens]

Sequence ID: 3IVH_A Length: 406 >Design and Synthesis of Potent BACE-1 Inhibitors with Cellular Activity: Structure-Activity Relationship of P1 Substituents [Homo sapiens]

Sequence ID: 3IVI_A Length: 406 >Design and Synthesis of Potent BACE-1 Inhibitors with Cellular Activity: Structure-Activity Relationship of P1 Substituents [Homo sapiens]

Sequence ID: 3IVI_B Length: 406 >Design and Synthesis of Potent BACE-1 Inhibitors with Cellular Activity: Structure-Activity Relationship of P1 Substituents [Homo sapiens]

Sequence ID: 3IVI_C Length: 406 >BACE-1 in complex with ELN380842 [Homo sapiens]

Sequence ID: 3N4L_A Length: 406 >BACE-1 in complex with ELN380842 [Homo sapiens]

Sequence ID: 3N4L_B Length: 406 >BACE-1 in complex with ELN380842 [Homo sapiens]

Sequence ID: 3N4L_C Length: 406 >BACE-1 in complex with ELN475957 [Homo sapiens]

Sequence ID: 3NSH_A Length: 406 >BACE-1 in complex with ELN475957 [Homo sapiens]

Sequence ID: 3NSH_B Length: 406 >BACE-1 in complex with ELN475957 [Homo sapiens]

Sequence ID: 3NSH_C Length: 406 >Structure-based design of novel dihydroisoquinoline BACE-1 inhibitors that do not engage the catalytic aspartates [Homo sapiens]

Sequence ID: 4HZT_A Length: 406 >Design and Synthesis of Thiophene Dihydroisoquinolins as Novel BACE-1 Inhibitors [Homo sapiens]

Sequence ID: 4I0D_A Length: 406 >Design and Synthesis of Thiophene Dihydroisoquinolins as Novel BACE-1 Inhibitors [Homo sapiens]

Sequence ID: 4I0E_A Length: 406 >Design and Synthesis of Thiophene Dihydroisoquinolins as Novel BACE-1 Inhibitors [Homo sapiens]

Sequence ID: 4I0F_A Length: 406 >Design and Synthesis of Thiophene Dihydroisoquinolins as Novel BACE-1 Inhibitors [Homo sapiens]

Sequence ID: 4I0G_A Length: 406 >SPR and structural analysis yield insight towards mechanism of inhibition of BACE inhibitors. [Homo sapiens]

Sequence ID: 4I0H_A Length: 406 >SPR and structural analysis yield insight towards mechanism of inhibition of BACE inhibitors. [Homo sapiens]

Sequence ID: 4I0H_B Length: 406 >SPR and structural analysis yield insight towards mechanism of inhibition of BACE inhibitors. [Homo sapiens]

Sequence ID: 4I0H_C Length: 406 >SPR and structural analysis yield insight towards mechanism of inhibition of BACE inhibitors [Homo sapiens]

Sequence ID: 4I0I_A Length: 406 >SPR and structural analysis yield insight towards mechanism of inhibition of BACE inhibitors [Homo sapiens]

Sequence ID: 4I0I_B Length: 406 >SPR and structural analysis yield insight towards mechanism of inhibition of BACE inhibitors [Homo sapiens]

Sequence ID: 4I0I_C Length: 406 >SPR and structural analysis yield insight towards mechanism of inhibition of BACE inhibitors [Homo sapiens]

Sequence ID: 4I0J_A Length: 406 >Structure-based design of novel dihydroisoquinoline BACE-1 inhibitors that do not engage the catalytic aspartates [Homo sapiens]

Sequence ID: 4I0Z_A Length: 406 >Structure-based design of novel dihydroisoquinoline BACE-1 inhibitors that do not engage the catalytic aspartates [Homo sapiens]

Sequence ID: 4I10_A Length: 406 >Structure-based design of novel dihydroisoquinoline BACE-1 inhibitors that do not engage the catalytic aspartates. [Homo sapiens]

Sequence ID: 4I11_A Length: 406 >Design and synthesis of thiophene dihydroisoquinolins as novel BACE-1 inhibitors [Homo sapiens]

Sequence ID: 4I12_A Length: 406 >Design and synthesis of thiophene dihydroisoquinolins as novel BACE-1 inhibitors [Homo sapiens]

Sequence ID: 4I1C_A Length: 406 >Spirocyclic Beta-Site Amyloid Precursor Protein Cleaving Enzyme 1 (BACE1) Inhibitors [Homo sapiens]

Sequence ID: 4JOO_A Length: 406 >Spirocyclic Beta-Site Amyloid Precursor Protein Cleaving Enzyme 1 (BACE1) Inhibitors [Homo sapiens]

Sequence ID: 4JP9_A Length: 406 >Spirocyclic Beta-Site Amyloid Precursor Protein Cleaving Enzyme 1 (BACE1) Inhibitors [Homo sapiens]

Sequence ID: 4JPC_A Length: 406 >Spirocyclic Beta-Site Amyloid Precursor Protein Cleaving Enzyme 1 (BACE1) Inhibitors [Homo sapiens]

Sequence ID: 4JPE_A Length: 406 >Aminooxazoline inhibitor of BACE-1 [Homo sapiens]

Sequence ID: 4LC7_A Length: 406 >Discovery of 7-THP chromans: BACE1 inhibitors that reduce A-beta in the CNS [Homo sapiens]

Sequence ID: 4N00_A Length: 406 >Synthesis, Characterization and PK/PD Studies of a Series of Spirocyclic Pyranochromene BACE1 Inhibitors [Homo sapiens]

Sequence ID: 4PZW_A Length: 406 >Synthesis, Characterization and PK/PD Studies of a Series of Spirocyclic Pyranochromene BACE1 Inhibitors [Homo sapiens]

Sequence ID: 4PZX_A Length: 406 >8-Tetrahydropyran-2-yl chromans: highly selective beta-site amyloid precursor protein cleaving enzyme 1 (BACE1) inhibitors [Homo sapiens]

Sequence ID: 4R5N_A Length: 406 >8-Tetrahydropyran-2-yl chromans: highly selective beta-site amyloid precursor protein cleaving enzyme 1 (BACE1) inhibitors [Homo sapiens]

Sequence ID: 4RRN_A Length: 406 >8-Tetrahydropyran-2-yl chromans: highly selective beta-site amyloid precursor protein cleaving enzyme 1 (BACE1) inhibitors [Homo sapiens]

Sequence ID: 4RRO_A Length: 406 >8-Tetrahydropyran-2-yl chromans: highly selective beta-site amyloid precursor protein cleaving enzyme 1 (BACE1) inhibitors [Homo sapiens]

Sequence ID: 4RRS_A Length: 406

Range 1: 20 to 287

Score:56.2 bits(134), Expect:2e-07,

Method:Compositional matrix adjust.,

Identities:77/289(27%), Positives:117/289(40%), Gaps:58/289(20%)

Query 96 YSANFTVGSNSQKQNVIVDTGSSDLWVVDSSANCQEKSGYSSDYCFSGGTYDPSSSSTIQ 155

Y TVGS Q N++VDTGSS+ V ++ + F Y SST +

Sbjct 20 YYVEMTVGSPPQTLNILVDTGSSNFAVG------------AAPHPFLHRYYQRQLSSTYR 67

Query 156 ELGKSFNIRYGDGSSSSGTWVKD--------------TVGINGAIILNQQFGDVNSTSVS 201

+L K + Y + G W + TV N A I +N ++

Sbjct 68 DLRKGVYVPY-----TQGKWEGELGTDLVSIPHGPNVTVRANIAAITESDKFFINGSNW- 121

Query 202 QGILGIGLDTNESTDTIYENFPINLKEQGFINTNAYSLYL----------NAPSATSGTI 251

+GILG+ D E F +L +Q + N +SL L ++ G++

Sbjct 122 EGILGLAYAEIARPDDSLEPFFDSLVKQTHV-PNLFSLQLCGAGFPLNQSEVLASVGGSM 180

Query 252 IFGGIDHAKYTGSLTTLPLTSNREFTIQTNSATVGTSTIDINTGL--------LLDSGTT 303

I GGIDH+ YTGSL P+ RE+ + V + D+ ++DSGTT

Sbjct 181 IIGGIDHSLYTGSLWYTPI--RREWYYEVIIVRVEINGQDLKMDCKEYNYDKSIVDSGTT 238

Query 304 LTYLPQSVVDSIANAI-GGDITYNRPIGAYIWS---CNRNGKVTYN-FP 347

LP+ V ++ +I T P G ++ C + G +N FP

Sbjct 239 NLRLPKKVFEAAVKSIKAASSTEKFPDGFWLGEQLVCWQAGTTPWNIFP 287

>Crystal structure of BACE1 in complex with OM99-2 at pH 5.0 [Homo sapiens]

Sequence ID: 2ZHR_A Length: 411

>Crystal structure of BACE1 in complex with OM99-2 at pH 5.0 [Homo sapiens]

Sequence ID: 2ZHR_B Length: 411 >Crystal structure of BACE1 at pH 4.0 [Homo sapiens]

Sequence ID: 2ZHS_A Length: 411 >Crystal structure of BACE1 at pH 4.5 [Homo sapiens]

Sequence ID: 2ZHT_A Length: 411 >Crystal structure of BACE1 at pH 5.0 [Homo sapiens]

Sequence ID: 2ZHU_A Length: 411 >Crystal structure of BACE1 at pH 7.0 [Homo sapiens]

Sequence ID: 2ZHV_A Length: 411

Range 1: 32 to 299

Score:56.2 bits(134), Expect:2e-07,

Method:Compositional matrix adjust.,

Identities:77/289(27%), Positives:117/289(40%), Gaps:58/289(20%)

Query 96 YSANFTVGSNSQKQNVIVDTGSSDLWVVDSSANCQEKSGYSSDYCFSGGTYDPSSSSTIQ 155

Y TVGS Q N++VDTGSS+ V ++ + F Y SST +

Sbjct 32 YYVEMTVGSPPQTLNILVDTGSSNFAVG------------AAPHPFLHRYYQRQLSSTYR 79

Query 156 ELGKSFNIRYGDGSSSSGTWVKD--------------TVGINGAIILNQQFGDVNSTSVS 201

+L K + Y + G W + TV N A I +N ++

Sbjct 80 DLRKGVYVPY-----TQGKWEGELGTDLVSIPHGPNVTVRANIAAITESDKFFINGSNW- 133

Query 202 QGILGIGLDTNESTDTIYENFPINLKEQGFINTNAYSLYL----------NAPSATSGTI 251

+GILG+ D E F +L +Q + N +SL L ++ G++

Sbjct 134 EGILGLAYAEIARPDDSLEPFFDSLVKQTHV-PNLFSLQLCGAGFPLNQSEVLASVGGSM 192

Query 252 IFGGIDHAKYTGSLTTLPLTSNREFTIQTNSATVGTSTIDINTGL--------LLDSGTT 303

I GGIDH+ YTGSL P+ RE+ + V + D+ ++DSGTT

Sbjct 193 IIGGIDHSLYTGSLWYTPI--RREWYYEVIIVRVEINGQDLKMDCKEYNYDKSIVDSGTT 250

Query 304 LTYLPQSVVDSIANAI-GGDITYNRPIGAYIWS---CNRNGKVTYN-FP 347

LP+ V ++ +I T P G ++ C + G +N FP

Sbjct 251 NLRLPKKVFEAAVKSIKAASSTEKFPDGFWLGEQLVCWQAGTTPWNIFP 299

>X-ray crystal structure of beta secretase complexed with compound 5 [Homo sapiens]

Sequence ID: 2OF0_A Length: 402

>X-ray crystal structure of beta secretase complexed with 1-amino-isoquinoline [Homo sapiens]

Sequence ID: 2OHK_A Length: 402 >X-ray crystal structure of beta secretase complexed with 2-aminoquinoline [Homo sapiens]

Sequence ID: 2OHL_A Length: 402 >X-ray crystal structure of beta secretase complexed with N~3~-benzylpyridine-2,3-diamine [Homo sapiens]

Sequence ID: 2OHM_A Length: 402 >X-ray crystal structure of beta secretase complexed with 4-(4-fluorobenzyl)piperidine [Homo sapiens]

Sequence ID: 2OHN_A Length: 402 >X-ray crystal structure of beta secretase complexed with compound 3 [Homo sapiens]

Sequence ID: 2OHP_A Length: 402 >X-ray crystal structure of beta secretase complexed with compound 4 [Homo sapiens]

Sequence ID: 2OHQ_A Length: 402 >X-ray crystal structure of beta secretase complexed with compound 6a [Homo sapiens]

Sequence ID: 2OHR_A Length: 402 >X-ray crystal structure of beta secretase complexed with compound 6b [Homo sapiens]

Sequence ID: 2OHS_A Length: 402 >X-ray crystal structure of beta secretase complexed with compound 7 [Homo sapiens]

Sequence ID: 2OHT_A Length: 402 >X-ray crystal structure of beta secretase complexed with compound 8b [Homo sapiens]

Sequence ID: 2OHU_A Length: 402

Range 1: 31 to 298

Score:56.2 bits(134), Expect:2e-07,

Method:Compositional matrix adjust.,

Identities:77/289(27%), Positives:117/289(40%), Gaps:58/289(20%)

Query 96 YSANFTVGSNSQKQNVIVDTGSSDLWVVDSSANCQEKSGYSSDYCFSGGTYDPSSSSTIQ 155

Y TVGS Q N++VDTGSS+ V ++ + F Y SST +

Sbjct 31 YYVEMTVGSPPQTLNILVDTGSSNFAVG------------AAPHPFLHRYYQRQLSSTYR 78

Query 156 ELGKSFNIRYGDGSSSSGTWVKD--------------TVGINGAIILNQQFGDVNSTSVS 201

+L K + Y + G W + TV N A I +N ++

Sbjct 79 DLRKGVYVPY-----TQGKWEGELGTDLVSIPHGPNVTVRANIAAITESDKFFINGSNW- 132

Query 202 QGILGIGLDTNESTDTIYENFPINLKEQGFINTNAYSLYL----------NAPSATSGTI 251

+GILG+ D E F +L +Q + N +SL L ++ G++

Sbjct 133 EGILGLAYAEIARPDDSLEPFFDSLVKQTHV-PNLFSLQLCGAGFPLNQSEVLASVGGSM 191

Query 252 IFGGIDHAKYTGSLTTLPLTSNREFTIQTNSATVGTSTIDINTGL--------LLDSGTT 303

I GGIDH+ YTGSL P+ RE+ + V + D+ ++DSGTT

Sbjct 192 IIGGIDHSLYTGSLWYTPI--RREWYYEVIIVRVEINGQDLKMDCKEYNYDKSIVDSGTT 249

Query 304 LTYLPQSVVDSIANAI-GGDITYNRPIGAYIWS---CNRNGKVTYN-FP 347

LP+ V ++ +I T P G ++ C + G +N FP

Sbjct 250 NLRLPKKVFEAAVKSIKAASSTEKFPDGFWLGEQLVCWQAGTTPWNIFP 298

>Structure of bace (beta secretase) in Complex with EV0 [Homo sapiens]

Sequence ID: 3HVG_A Length: 411

>Structure of bace (beta secretase) in Complex with EV0 [Homo sapiens]

Sequence ID: 3HVG_B Length: 411 >Structure of bace (beta secretase) in Complex with EV0 [Homo sapiens]

Sequence ID: 3HVG_C Length: 411 >Structure of Bace (beta secretase) in complex with ligand EV2 [Homo sapiens]

Sequence ID: 3HW1_A Length: 411 >Structure of Bace (beta secretase) in complex with ligand EV2 [Homo sapiens]

Sequence ID: 3HW1_B Length: 411 >Structure of Bace (beta secretase) in complex with ligand EV2 [Homo sapiens]

Sequence ID: 3HW1_C Length: 411 >Structure of bace (beta secretase) in complex with inhibitor [Homo sapiens]

Sequence ID: 3MSJ_A Length: 411 >Structure of bace (beta secretase) in complex with inhibitor [Homo sapiens]

Sequence ID: 3MSJ_B Length: 411 >Structure of bace (beta secretase) in complex with inhibitor [Homo sapiens]

Sequence ID: 3MSJ_C Length: 411

Range 1: 33 to 300

Score:56.2 bits(134), Expect:2e-07,

Method:Compositional matrix adjust.,

Identities:77/289(27%), Positives:117/289(40%), Gaps:58/289(20%)

Query 96 YSANFTVGSNSQKQNVIVDTGSSDLWVVDSSANCQEKSGYSSDYCFSGGTYDPSSSSTIQ 155

Y TVGS Q N++VDTGSS+ V ++ + F Y SST +

Sbjct 33 YYVEMTVGSPPQTLNILVDTGSSNFAVG------------AAPHPFLHRYYQRQLSSTYR 80

Query 156 ELGKSFNIRYGDGSSSSGTWVKD--------------TVGINGAIILNQQFGDVNSTSVS 201

+L K + Y + G W + TV N A I +N ++

Sbjct 81 DLRKGVYVPY-----TQGKWEGELGTDLVSIPHGPNVTVRANIAAITESDKFFINGSNW- 134

Query 202 QGILGIGLDTNESTDTIYENFPINLKEQGFINTNAYSLYL----------NAPSATSGTI 251

+GILG+ D E F +L +Q + N +SL L ++ G++

Sbjct 135 EGILGLAYAEIARPDDSLEPFFDSLVKQTHV-PNLFSLQLCGAGFPLNQSEVLASVGGSM 193

Query 252 IFGGIDHAKYTGSLTTLPLTSNREFTIQTNSATVGTSTIDINTGL--------LLDSGTT 303

I GGIDH+ YTGSL P+ RE+ + V + D+ ++DSGTT

Sbjct 194 IIGGIDHSLYTGSLWYTPI--RREWYYEVIIVRVEINGQDLKMDCKEYNYDKSIVDSGTT 251

Query 304 LTYLPQSVVDSIANAI-GGDITYNRPIGAYIWS---CNRNGKVTYN-FP 347

LP+ V ++ +I T P G ++ C + G +N FP

Sbjct 252 NLRLPKKVFEAAVKSIKAASSTEKFPDGFWLGEQLVCWQAGTTPWNIFP 300

>BACE1 in complex with (S)-5-(3-chloro-5-(5-(prop-1-yn-1-yl)pyridin-3-yl)thiophen-2-yl)-2,5-dimethyl-1,2,4-thiadiazinan-3-iminium 1,1-dioxide [Homo sapiens]

Sequence ID: 5HTZ_A Length: 412

>BACE1 in complex with (S)-5-(3-chloro-5-(5-(prop-1-yn-1-yl)pyridin-3-yl)thiophen-2-yl)-2,5-dimethyl-1,2,4-thiadiazinan-3-iminium 1,1-dioxide [Homo sapiens]

Sequence ID: 5HTZ_B Length: 412 >BACE1 in complex with 4-(3-(furan-2-carboxamido)phenyl)-1-methyl-5-oxo-4-phenylimidazolidin-2-iminium [Homo sapiens]

Sequence ID: 5HU0_A Length: 412 >BACE1 in complex with 4-(3-(furan-2-carboxamido)phenyl)-1-methyl-5-oxo-4-phenylimidazolidin-2-iminium [Homo sapiens]

Sequence ID: 5HU0_B Length: 412 >BACE1 in complex with (R)-N-(3-(3-amino-2,5-dimethyl-1,1-dioxido-5,6-dihydro-2H-1,2,4-thiadiazin-5-yl)-4-fluorophenyl)-5-fluoropicolinamide [Homo sapiens]

Sequence ID: 5HU1_A Length: 412 >BACE1 in complex with (R)-N-(3-(3-amino-2,5-dimethyl-1,1-dioxido-5,6-dihydro-2H-1,2,4-thiadiazin-5-yl)-4-fluorophenyl)-5-fluoropicolinamide [Homo sapiens]

Sequence ID: 5HU1_B Length: 412

Range 1: 33 to 300

Score:56.2 bits(134), Expect:2e-07,

Method:Compositional matrix adjust.,

Identities:77/289(27%), Positives:117/289(40%), Gaps:58/289(20%)

Query 96 YSANFTVGSNSQKQNVIVDTGSSDLWVVDSSANCQEKSGYSSDYCFSGGTYDPSSSSTIQ 155

Y TVGS Q N++VDTGSS+ V ++ + F Y SST +

Sbjct 33 YYVEMTVGSPPQTLNILVDTGSSNFAVG------------AAPHPFLHRYYQRQLSSTYR 80

Query 156 ELGKSFNIRYGDGSSSSGTWVKD--------------TVGINGAIILNQQFGDVNSTSVS 201

+L K + Y + G W + TV N A I +N ++

Sbjct 81 DLRKGVYVPY-----TQGKWEGELGTDLVSIPHGPNVTVRANIAAITESDKFFINGSNW- 134

Query 202 QGILGIGLDTNESTDTIYENFPINLKEQGFINTNAYSLYL----------NAPSATSGTI 251

+GILG+ D E F +L +Q + N +SL L ++ G++

Sbjct 135 EGILGLAYAEIARPDDSLEPFFDSLVKQTHV-PNLFSLQLCGAGFPLNQSEVLASVGGSM 193

Query 252 IFGGIDHAKYTGSLTTLPLTSNREFTIQTNSATVGTSTIDINTGL--------LLDSGTT 303

I GGIDH+ YTGSL P+ RE+ + V + D+ ++DSGTT

Sbjct 194 IIGGIDHSLYTGSLWYTPI--RREWYYEVIIVRVEINGQDLKMDCKEYNYDKSIVDSGTT 251

Query 304 LTYLPQSVVDSIANAI-GGDITYNRPIGAYIWS---CNRNGKVTYN-FP 347

LP+ V ++ +I T P G ++ C + G +N FP

Sbjct 252 NLRLPKKVFEAAVKSIKAASSTEKFPDGFWLGEQLVCWQAGTTPWNIFP 300

>Structure of BACE Bound to SCH589432 [Homo sapiens]

Sequence ID: 3L58_A Length: 414

>Structure of BACE Bound to SCH589432 [Homo sapiens]

Sequence ID: 3L58_B Length: 414 >Structure of BACE Bound to SCH710413 [Homo sapiens]

Sequence ID: 3L59_A Length: 414 >Structure of BACE Bound to SCH710413 [Homo sapiens]

Sequence ID: 3L59_B Length: 414 >Structure of BACE Bound to SCH713601 [Homo sapiens]

Sequence ID: 3L5B_A Length: 414 >Structure of BACE Bound to SCH713601 [Homo sapiens]

Sequence ID: 3L5B_B Length: 414 >Structure of BACE Bound to SCH723871 [Homo sapiens]

Sequence ID: 3L5C_A Length: 414 >Structure of BACE Bound to SCH723871 [Homo sapiens]

Sequence ID: 3L5C_B Length: 414 >Structure of BACE Bound to SCH723873 [Homo sapiens]

Sequence ID: 3L5D_A Length: 414 >Structure of BACE Bound to SCH723873 [Homo sapiens]

Sequence ID: 3L5D_B Length: 414 >Structure of BACE Bound to SCH736062 [Homo sapiens]

Sequence ID: 3L5E_A Length: 414 >Structure of BACE Bound to SCH736062 [Homo sapiens]

Sequence ID: 3L5E_B Length: 414 >Structure of BACE Bound to SCH736201 [Homo sapiens]

Sequence ID: 3L5F_A Length: 414 >Structure of BACE Bound to SCH736201 [Homo sapiens]

Sequence ID: 3L5F_B Length: 414 >Structure of BACE Bound to 2-imino-3-methyl-5,5-diphenylimidazolidin-4-one [Homo sapiens]

Sequence ID: 4DJU_A Length: 414 >Structure of BACE Bound to 2-imino-3-methyl-5,5-diphenylimidazolidin-4-one [Homo sapiens]

Sequence ID: 4DJU_B Length: 414 >Structure of BACE Bound to 2-imino-5-(3'-methoxy-[1,1'-biphenyl]-3-yl)-3-methyl-5-phenylimidazolidin-4-one [Homo sapiens]

Sequence ID: 4DJV_A Length: 414 >Structure of BACE Bound to 2-imino-5-(3'-methoxy-[1,1'-biphenyl]-3-yl)-3-methyl-5-phenylimidazolidin-4-one [Homo sapiens]

Sequence ID: 4DJV_B Length: 414 >Structure of BACE Bound to 2-imino-3-methyl-5-phenyl-5-(3-(pyridin-3-yl)phenyl)imidazolidin-4-one [Homo sapiens]

Sequence ID: 4DJW_A Length: 414 >Structure of BACE Bound to 2-imino-3-methyl-5-phenyl-5-(3-(pyridin-3-yl)phenyl)imidazolidin-4-one [Homo sapiens]

Sequence ID: 4DJW_B Length: 414 >Structure of BACE Bound to 5-(3-(5-chloropyridin-3-yl)phenyl)-5-cyclopropyl-2-imino-3-methylimidazolidin-4-one [Homo sapiens]

Sequence ID: 4DJX_A Length: 414 >Structure of BACE Bound to 5-(3-(5-chloropyridin-3-yl)phenyl)-5-cyclopropyl-2-imino-3-methylimidazolidin-4-one [Homo sapiens]

Sequence ID: 4DJX_B Length: 414 >Structure of BACE Bound to (R)-5-cyclopropyl-2-imino-3-methyl-5-(3-(5-(prop-1-yn-1-yl)pyridin-3-yl)phenyl)imidazolidin-4-one [Homo sapiens]

Sequence ID: 4DJY_A Length: 414 >Structure of BACE Bound to (R)-5-cyclopropyl-2-imino-3-methyl-5-(3-(5-(prop-1-yn-1-yl)pyridin-3-yl)phenyl)imidazolidin-4-one [Homo sapiens]

Sequence ID: 4DJY_B Length: 414 >Structure of BACE-1 Bound to (7aR)-6-benzoyl-7a-(4-(3-cyanophenyl)thiophen-2-yl)-3-methyl-4-oxohexahydro-1H-pyrrolo[3,4-d]pyrimidin-2(3H)-iminium [Homo sapiens]

Sequence ID: 4H1E_A Length: 414 >Structure of BACE-1 Bound to (7aR)-6-benzoyl-7a-(4-(3-cyanophenyl)thiophen-2-yl)-3-methyl-4-oxohexahydro-1H-pyrrolo[3,4-d]pyrimidin-2(3H)-iminium [Homo sapiens]

Sequence ID: 4H1E_B Length: 414 >Structure of BACE Bound to 3-(5-((7aR)-2-imino-6-(6-methoxypyridin-2-yl)-3-methyl-4-oxooctahydro-1H-pyrrolo[3,4-d]pyrimidin-7a-yl)thiophen-3-yl)benzonitrile [Homo sapiens]

Sequence ID: 4H3F_A Length: 414 >Structure of BACE Bound to 3-(5-((7aR)-2-imino-6-(6-methoxypyridin-2-yl)-3-methyl-4-oxooctahydro-1H-pyrrolo[3,4-d]pyrimidin-7a-yl)thiophen-3-yl)benzonitrile [Homo sapiens]

Sequence ID: 4H3F_B Length: 414 >Structure of BACE Bound to 2-((7aR)-7a-(4-(3-cyanophenyl)thiophen-2-yl)-2-imino-3-methyl-4-oxohexahydro-1H-pyrrolo[3,4-d]pyrimidin-6(2H)-yl)nicotinonitrile [Homo sapiens]

Sequence ID: 4H3G_A Length: 414 >Structure of BACE Bound to 2-((7aR)-7a-(4-(3-cyanophenyl)thiophen-2-yl)-2-imino-3-methyl-4-oxohexahydro-1H-pyrrolo[3,4-d]pyrimidin-6(2H)-yl)nicotinonitrile [Homo sapiens]

Sequence ID: 4H3G_B Length: 414 >Structure of BACE Bound to 3-(5-((7aR)-2-imino-6-(3-methoxypyridin-2-yl)-3-methyl-4-oxooctahydro-1H-pyrrolo[3,4-d]pyrimidin-7a-yl)thiophen-3-yl)benzonitrile [Homo sapiens]

Sequence ID: 4H3I_A Length: 414 >Structure of BACE Bound to 3-(5-((7aR)-2-imino-6-(3-methoxypyridin-2-yl)-3-methyl-4-oxooctahydro-1H-pyrrolo[3,4-d]pyrimidin-7a-yl)thiophen-3-yl)benzonitrile [Homo sapiens]

Sequence ID: 4H3I_B Length: 414 >Structure of BACE Bound to 2-fluoro-5-(5-(2-imino-3-methyl-4-oxo-6-phenyloctahydro-1H-pyrrolo[3,4-d]pyrimidin-7a-yl)thiophen-2-yl)benzonitrile [Homo sapiens]

Sequence ID: 4H3J_A Length: 414 >Structure of BACE Bound to 2-fluoro-5-(5-(2-imino-3-methyl-4-oxo-6-phenyloctahydro-1H-pyrrolo[3,4-d]pyrimidin-7a-yl)thiophen-2-yl)benzonitrile [Homo sapiens]

Sequence ID: 4H3J_B Length: 414 >Structure of BACE Bound to (S)-3-(5-(2-imino-1,4-dimethyl-6-oxohexahydropyrimidin-4-yl)thiophen-3-yl)benzonitrile [Homo sapiens]

Sequence ID: 4HA5_A Length: 414 >Structure of BACE Bound to (S)-3-(5-(2-imino-1,4-dimethyl-6-oxohexahydropyrimidin-4-yl)thiophen-3-yl)benzonitrile [Homo sapiens]

Sequence ID: 4HA5_B Length: 414 >BACE-1 in complex with (R)-4-(2-cyclohexylethyl)-4-(((R)-1-(2-cyclopentylacetyl)pyrrolidin-3-yl)methyl)-1-methyl-5-oxoimidazolidin-2-iminium [Homo sapiens]

Sequence ID: 4R8Y_A Length: 414 >BACE-1 in complex with (R)-4-(2-cyclohexylethyl)-4-(((R)-1-(2-cyclopentylacetyl)pyrrolidin-3-yl)methyl)-1-methyl-5-oxoimidazolidin-2-iminium [Homo sapiens]

Sequence ID: 4R8Y_B Length: 414 >BACE-1 in complex with (R)-4-(2-cyclohexylethyl)-4-(((1S,3R)-3-(cyclopentylamino)cyclohexyl)methyl)-1-methyl-5-oxoimidazolidin-2-iminium [Homo sapiens]

Sequence ID: 4R91_A Length: 414 >BACE-1 in complex with (R)-4-(2-cyclohexylethyl)-4-(((1S,3R)-3-(cyclopentylamino)cyclohexyl)methyl)-1-methyl-5-oxoimidazolidin-2-iminium [Homo sapiens]

Sequence ID: 4R91_B Length: 414 >BACE-1 in complex with (R)-4-(2-cyclohexylethyl)-4-(((1S,3R)-3-(isonicotinamido)cyclohexyl)methyl)-1-methyl-5-oxoimidazolidin-2-iminium [Homo sapiens]

Sequence ID: 4R92_A Length: 414 >BACE-1 in complex with (R)-4-(2-cyclohexylethyl)-4-(((1S,3R)-3-(isonicotinamido)cyclohexyl)methyl)-1-methyl-5-oxoimidazolidin-2-iminium [Homo sapiens]

Sequence ID: 4R92_B Length: 414 >BACE-1 in complex with (R)-4-(2-cyclohexylethyl)-1-methyl-5-oxo-4-(((1S,3R)-3-(3-phenylureido)cyclohexyl)methyl)imidazolidin-2-iminium [Homo sapiens]

Sequence ID: 4R93_A Length: 414 >BACE-1 in complex with (R)-4-(2-cyclohexylethyl)-1-methyl-5-oxo-4-(((1S,3R)-3-(3-phenylureido)cyclohexyl)methyl)imidazolidin-2-iminium [Homo sapiens]

Sequence ID: 4R93_B Length: 414 >BACE-1 in complex with 2-(((1R,3S)-3-(((R)-4-(2-cyclohexylethyl)-2-iminio-1-methyl-5-oxoimidazolidin-4-yl)methyl)cyclohexyl)amino)quinolin-1-ium [Homo sapiens]

Sequence ID: 4R95_A Length: 414 >BACE-1 in complex with 2-(((1R,3S)-3-(((R)-4-(2-cyclohexylethyl)-2-iminio-1-methyl-5-oxoimidazolidin-4-yl)methyl)cyclohexyl)amino)quinolin-1-ium [Homo sapiens]

Sequence ID: 4R95_B Length: 414 >BACE-1 in complex with (7aR)-7a-(4-(3-cyanophenyl)thiophen-2-yl)-6-(5-fluoropyrimidin-2-yl)-3-methyl-4-oxooctahydro-2H-pyrrolo[3,4-d]pyrimidin-2-iminium [Homo sapiens]

Sequence ID: 5HD0_A Length: 414 >BACE-1 in complex with (7aR)-7a-(4-(3-cyanophenyl)thiophen-2-yl)-6-(5-fluoropyrimidin-2-yl)-3-methyl-4-oxooctahydro-2H-pyrrolo[3,4-d]pyrimidin-2-iminium [Homo sapiens]

Sequence ID: 5HD0_B Length: 414 >BACE-1 incomplex with (7aR)-7a-(4-(3-cyanophenyl)thiophen-2-yl)-6-(5-fluoro-4-methoxypyrimidin-2-yl)-3-methyl-4-oxooctahydro-2H-pyrrolo[3,4-d]pyrimidin-2-iminium [Homo sapiens]

Sequence ID: 5HDU_A Length: 414 >BACE-1 incomplex with (7aR)-7a-(4-(3-cyanophenyl)thiophen-2-yl)-6-(5-fluoro-4-methoxypyrimidin-2-yl)-3-methyl-4-oxooctahydro-2H-pyrrolo[3,4-d]pyrimidin-2-iminium [Homo sapiens]

Sequence ID: 5HDU_B Length: 414 >BACE-1 incomplex with (7aR)-7a-(5-cyanothiophen-2-yl)-6-(5-fluoro-4-methoxy-6-methylpyrimidin-2-yl)-3-methyl-4-oxooctahydro-2H-pyrrolo[3,4-d]pyrimidin-2-iminium [Homo sapiens]

Sequence ID: 5HDV_A Length: 414 >BACE-1 incomplex with (7aR)-7a-(5-cyanothiophen-2-yl)-6-(5-fluoro-4-methoxy-6-methylpyrimidin-2-yl)-3-methyl-4-oxooctahydro-2H-pyrrolo[3,4-d]pyrimidin-2-iminium [Homo sapiens]

Sequence ID: 5HDV_B Length: 414 >BACE-1 in complex with (7aR)-7a-(5-cyanothiophen-2-yl)-6-(4-ethoxy-5-fluoro-6-methylpyrimidin-2-yl)-3-methyl-4-oxooctahydro-2H-pyrrolo[3,4-d]pyrimidin-2-iminium [Homo sapiens]

Sequence ID: 5HDX_A Length: 414 >BACE-1 in complex with (7aR)-7a-(5-cyanothiophen-2-yl)-6-(4-ethoxy-5-fluoro-6-methylpyrimidin-2-yl)-3-methyl-4-oxooctahydro-2H-pyrrolo[3,4-d]pyrimidin-2-iminium [Homo sapiens]

Sequence ID: 5HDX_B Length: 414 >BACE-1 in complex with (7aR)-7a-(5-cyanothiophen-2-yl)-6-(5-fluoro-4-methyl-6-(methylthio)pyrimidin-2-yl)-3-methyl-4-oxooctahydro-2H-pyrrolo[3,4-d]pyrimidin-2-iminium [Homo sapiens]

Sequence ID: 5HDZ_A Length: 414 >BACE-1 in complex with (7aR)-7a-(5-cyanothiophen-2-yl)-6-(5-fluoro-4-methyl-6-(methylthio)pyrimidin-2-yl)-3-methyl-4-oxooctahydro-2H-pyrrolo[3,4-d]pyrimidin-2-iminium [Homo sapiens]

Sequence ID: 5HDZ_B Length: 414 >BACE-1 in complex with (4aR,7aS)-7a-(2,6-difluorophenyl)-6-(5-fluoro-4-methoxy-6-methylpyrimidin-2-yl)-3-methyl-4-oxooctahydro-2H-pyrrolo[3,4-d]pyrimidin-2-iminium [Homo sapiens]

Sequence ID: 5HE4_A Length: 414 >BACE-1 in complex with (4aR,7aS)-7a-(2,6-difluorophenyl)-6-(5-fluoro-4-methoxy-6-methylpyrimidin-2-yl)-3-methyl-4-oxooctahydro-2H-pyrrolo[3,4-d]pyrimidin-2-iminium [Homo sapiens]

Sequence ID: 5HE4_B Length: 414 >BACE-1 in complex with (7aR)-7a-(5-cyanothiophen-2-yl)-6-(5-fluoro-4-methyl-6-(methylamino)pyrimidin-2-yl)-3-methyl-4-oxooctahydro-2H-pyrrolo[3,4-d]pyrimidin-2-iminium [Homo sapiens]

Sequence ID: 5HE5_A Length: 414 >BACE-1 in complex with (7aR)-7a-(5-cyanothiophen-2-yl)-6-(5-fluoro-4-methyl-6-(methylamino)pyrimidin-2-yl)-3-methyl-4-oxooctahydro-2H-pyrrolo[3,4-d]pyrimidin-2-iminium [Homo sapiens]

Sequence ID: 5HE5_B Length: 414 >BACE-1 in complex with (4aR,7aS)-7a-(2,4-difluorophenyl)-6-(5-fluoro-4-methoxy-6-methylpyrimidin-2-yl)-2-imino-3-methyloctahydro-4H-pyrrolo[3,4-d]pyrimidin-4-one [Homo sapiens]

Sequence ID: 5HE7_A Length: 414 >BACE-1 in complex with (4aR,7aS)-7a-(2,4-difluorophenyl)-6-(5-fluoro-4-methoxy-6-methylpyrimidin-2-yl)-2-imino-3-methyloctahydro-4H-pyrrolo[3,4-d]pyrimidin-4-one [Homo sapiens]

Sequence ID: 5HE7_B Length: 414

Range 1: 35 to 302

Score:56.2 bits(134), Expect:2e-07,

Method:Compositional matrix adjust.,

Identities:77/289(27%), Positives:117/289(40%), Gaps:58/289(20%)

Query 96 YSANFTVGSNSQKQNVIVDTGSSDLWVVDSSANCQEKSGYSSDYCFSGGTYDPSSSSTIQ 155

Y TVGS Q N++VDTGSS+ V ++ + F Y SST +

Sbjct 35 YYVEMTVGSPPQTLNILVDTGSSNFAVG------------AAPHPFLHRYYQRQLSSTYR 82

Query 156 ELGKSFNIRYGDGSSSSGTWVKD--------------TVGINGAIILNQQFGDVNSTSVS 201

+L K + Y + G W + TV N A I +N ++

Sbjct 83 DLRKGVYVPY-----TQGKWEGELGTDLVSIPHGPNVTVRANIAAITESDKFFINGSNW- 136

Query 202 QGILGIGLDTNESTDTIYENFPINLKEQGFINTNAYSLYL----------NAPSATSGTI 251

+GILG+ D E F +L +Q + N +SL L ++ G++

Sbjct 137 EGILGLAYAEIARPDDSLEPFFDSLVKQTHV-PNLFSLQLCGAGFPLNQSEVLASVGGSM 195

Query 252 IFGGIDHAKYTGSLTTLPLTSNREFTIQTNSATVGTSTIDINTGL--------LLDSGTT 303

I GGIDH+ YTGSL P+ RE+ + V + D+ ++DSGTT

Sbjct 196 IIGGIDHSLYTGSLWYTPI--RREWYYEVIIVRVEINGQDLKMDCKEYNYDKSIVDSGTT 253

Query 304 LTYLPQSVVDSIANAI-GGDITYNRPIGAYIWS---CNRNGKVTYN-FP 347

LP+ V ++ +I T P G ++ C + G +N FP

Sbjct 254 NLRLPKKVFEAAVKSIKAASSTEKFPDGFWLGEQLVCWQAGTTPWNIFP 302

>Crystal structure of BACE-1 in complex with inhibitor [Homo sapiens]

Sequence ID: 3CKP_A Length: 412

>Crystal structure of BACE-1 in complex with inhibitor [Homo sapiens]

Sequence ID: 3CKP_B Length: 412 >Crystal structure of BACE-1 in complex with inhibitor [Homo sapiens]

Sequence ID: 3CKP_C Length: 412 >Crystal structure of BACE-1 in complex with inhibitor [Homo sapiens]

Sequence ID: 3CKR_A Length: 412 >Crystal structure of BACE-1 in complex with inhibitor [Homo sapiens]

Sequence ID: 3CKR_B Length: 412 >Crystal structure of BACE-1 in complex with inhibitor [Homo sapiens]

Sequence ID: 3CKR_C Length: 412 >Crystal structure of beta-site app-cleaving enzyme 1 (BACE-DB-MUT) complex with N-(N-(4- acetamido-3-chloro-5-methylbenzyl)carbamimidoyl)-3-(4- methoxyphenyl)-5-methyl-4-isothiazolecarboxamide [Homo sapiens]

Sequence ID: 4FSL_A Length: 412 >Crystal structure of beta-site app-cleaving enzyme 1 (BACE-DB-MUT) complex with N-(N-(4- acetamido-3-chloro-5-methylbenzyl)carbamimidoyl)-3-(4- methoxyphenyl)-5-methyl-4-isothiazolecarboxamide [Homo sapiens]

Sequence ID: 4FSL_B Length: 412 >Crystal structure of beta-site app-cleaving enzyme 1 (BACE-DB-MUT) complex with N-(N-(4- acetamido-3-chloro-5-methylbenzyl)carbamimidoyl)-3-(4- methoxyphenyl)-5-methyl-4-isothiazolecarboxamide [Homo sapiens]

Sequence ID: 4FSL_D Length: 412 >Crystal structure of beta-site app-cleaving enzyme 1 (BACE-DB-MUT) complex with N-(N-(4- acetamido-3-chloro-5-methylbenzyl)carbamimidoyl)-3-(4- methoxyphenyl)-5-methyl-4-isothiazolecarboxamide [Homo sapiens]

Sequence ID: 4FSL_E Length: 412 >Crystal Structure Of Beta-site App-cleaving Enzyme 1 Complexed With N- (3-((4as,7as)-2-amino-4,4a,5,6-tetrahydro-7ah-furo[2,3-d][1, 3]thiazin-7a-yl)-4-fluorophenyl)-5-bromo-2-pyridinecarboxamide [Homo sapiens]

Sequence ID: 5TOL_A Length: 412

Range 1: 33 to 300

Score:56.2 bits(134), Expect:2e-07,

Method:Compositional matrix adjust.,

Identities:77/289(27%), Positives:117/289(40%), Gaps:58/289(20%)

Query 96 YSANFTVGSNSQKQNVIVDTGSSDLWVVDSSANCQEKSGYSSDYCFSGGTYDPSSSSTIQ 155

Y TVGS Q N++VDTGSS+ V ++ + F Y SST +

Sbjct 33 YYVEMTVGSPPQTLNILVDTGSSNFAVG------------AAPHPFLHRYYQRQLSSTYR 80

Query 156 ELGKSFNIRYGDGSSSSGTWVKD--------------TVGINGAIILNQQFGDVNSTSVS 201

+L K + Y + G W + TV N A I +N ++

Sbjct 81 DLRKGVYVPY-----TQGKWEGELGTDLVSIPHGPNVTVRANIAAITESDKFFINGSNW- 134

Query 202 QGILGIGLDTNESTDTIYENFPINLKEQGFINTNAYSLYL----------NAPSATSGTI 251

+GILG+ D E F +L +Q + N +SL L ++ G++

Sbjct 135 EGILGLAYAEIARPDDSLEPFFDSLVKQTHV-PNLFSLQLCGAGFPLNQSEVLASVGGSM 193

Query 252 IFGGIDHAKYTGSLTTLPLTSNREFTIQTNSATVGTSTIDINTGL--------LLDSGTT 303

I GGIDH+ YTGSL P+ RE+ + V + D+ ++DSGTT

Sbjct 194 IIGGIDHSLYTGSLWYTPI--RREWYYEVIIVRVEINGQDLKMDCKEYNYDKSIVDSGTT 251

Query 304 LTYLPQSVVDSIANAI-GGDITYNRPIGAYIWS---CNRNGKVTYN-FP 347

LP+ V ++ +I T P G ++ C + G +N FP

Sbjct 252 NLRLPKKVFEAAVKSIKAASSTEKFPDGFWLGEQLVCWQAGTTPWNIFP 300

>Crystal Structure of beta secetase in complex with 2-amino-3-methyl-6-((1S, 2R)-2-phenylcyclopropyl)pyrimidin-4(3H)-one [Homo sapiens]

Sequence ID: 3VV6_A Length: 416

>Crystal Structure of beta secetase in complex with 2-amino-6-((1S,2R)-2-(3'-methoxybiphenyl-3-yl)cyclopropyl)-3-methylpyrimidin-4(3H)-one [Homo sapiens]

Sequence ID: 3VV7_A Length: 416 >Crystal structure of beta secetase in complex with 2-amino-3-methyl-6-((1S,2R)-2-(3'-methylbiphenyl-4-yl)cyclopropyl)pyrimidin-4(3H)-one [Homo sapiens]

Sequence ID: 3VV8_A Length: 416 >Crystal Structure of beta secetase in complex with 2-amino-3,6-dimethyl-6-(2-phenylethyl)-3,4,5,6-tetrahydropyrimidin-4-one [Homo sapiens]

Sequence ID: 3WB4_A Length: 416 >Crystal Structure of beta secetase in complex with (6S)-2-amino-3,6-dimethyl-6-[(1R,2R)-2-phenylcyclopropyl]-3,4,5,6-tetrahydropyrimidin-4-one [Homo sapiens]

Sequence ID: 3WB5_A Length: 416 >Structure of BACE1 in complex with N-(3-((4R,5R,6S)-2-amino-6-(1,1-difluoroethyl)-5-fluoro-4-methyl-5,6-dihydro-4H-1,3-oxazin-4-yl)-4-fluorophenyl)-5-(fluoromethoxy)pyrazine-2-carboxamide [Homo sapiens]

Sequence ID: 5YGX_A Length: 416 >Crystal Structure of BACE1 in complex with (S)-N-(3-(2-amino-6-(fluoromethyl)-4 -methyl-4H-1,3-oxazin-4-yl)-4-fluorophenyl)-5-cyanopicolinamide [Homo sapiens]

Sequence ID: 5YGY_A Length: 416 >Crystal Structure of BACE1 in complex with N-(3-((4S,5R)-2-amino-4-methyl-5-phenyl-5,6-dihydro-4H-1,3-thiazin-4-yl)-4-fluorophenyl)-5-(fluoromethoxy)pyrazine-2-carboxamide [Homo sapiens]

Sequence ID: 6JSE_A Length: 416 >Crystal Structure of BACE1 in complex with N-(3-((4S,5S)-2-amino-4-methyl-5-phenyl-5,6-dihydro-4H-1,3-thiazin-4-yl)-4-fluorophenyl)-5-(fluoromethoxy)pyrazine-2-carboxamide [Homo sapiens]

Sequence ID: 6JSF_A Length: 416 >Crystal Structure of BACE1 in complex with N-{3-[(4S)-2-amino-4-methyl-5,6-dihydro-4H-1,3-thiazin-4-yl]-4-fluorophenyl}-5-chloropyridine-2-carboxamide [Homo sapiens]

Sequence ID: 6JSG_A Length: 416 >Crystal Structure of BACE1 in complex with N-{3-[(5R)-3-amino-5-methyl-9,9-dioxo-2,9lambda6-dithia-4-azaspiro[5.5]undec-3-en-5-yl]-4-fluorophenyl}-5-(fluoromethoxy)pyrazine-2-carboxamide [Homo sapiens]

Sequence ID: 6JSN_A Length: 416 >Crystal Structure of BACE1 in complex with N-{3-[(4R,5R,6R)-2-amino-5-fluoro-4,6-dimethyl-5,6-dihydro-4H-1,3-thiazin-4-yl]-4-fluorophenyl}-5-(fluoromethoxy)pyrazine-2-carboxamide [Homo sapiens]

Sequence ID: 6JT3_A Length: 416 >Crystal Structure of BACE1 in complex with N-{3-[(4S,6S)-2-amino-4-methyl-6-(trifluoromethyl)-5,6-dihydro-4H-1,3-thiazin-4-yl]-4-fluorophenyl}-5-(fluoromethoxy)pyrazine-2-carboxamide [Homo sapiens]

Sequence ID: 6JT4_A Length: 416 >Chain A, Beta-secretase 1 [Homo sapiens]

Sequence ID: 7DCZ_A Length: 416

Range 1: 37 to 304

Score:56.2 bits(134), Expect:2e-07,

Method:Compositional matrix adjust.,

Identities:77/289(27%), Positives:117/289(40%), Gaps:58/289(20%)

Query 96 YSANFTVGSNSQKQNVIVDTGSSDLWVVDSSANCQEKSGYSSDYCFSGGTYDPSSSSTIQ 155

Y TVGS Q N++VDTGSS+ V ++ + F Y SST +

Sbjct 37 YYVEMTVGSPPQTLNILVDTGSSNFAVG------------AAPHPFLHRYYQRQLSSTYR 84

Query 156 ELGKSFNIRYGDGSSSSGTWVKD--------------TVGINGAIILNQQFGDVNSTSVS 201

+L K + Y + G W + TV N A I +N ++

Sbjct 85 DLRKGVYVPY-----TQGKWEGELGTDLVSIPHGPNVTVRANIAAITESDKFFINGSNW- 138

Query 202 QGILGIGLDTNESTDTIYENFPINLKEQGFINTNAYSLYL----------NAPSATSGTI 251

+GILG+ D E F +L +Q + N +SL L ++ G++

Sbjct 139 EGILGLAYAEIARPDDSLEPFFDSLVKQTHV-PNLFSLQLCGAGFPLNQSEVLASVGGSM 197

Query 252 IFGGIDHAKYTGSLTTLPLTSNREFTIQTNSATVGTSTIDINTGL--------LLDSGTT 303

I GGIDH+ YTGSL P+ RE+ + V + D+ ++DSGTT

Sbjct 198 IIGGIDHSLYTGSLWYTPI--RREWYYEVIIVRVEINGQDLKMDCKEYNYDKSIVDSGTT 255

Query 304 LTYLPQSVVDSIANAI-GGDITYNRPIGAYIWS---CNRNGKVTYN-FP 347

LP+ V ++ +I T P G ++ C + G +N FP

Sbjct 256 NLRLPKKVFEAAVKSIKAASSTEKFPDGFWLGEQLVCWQAGTTPWNIFP 304

>BACE-1 IN COMPLEX WITH LIGAND 32397778 [Homo sapiens]

Sequence ID: 5MXD_A Length: 432

>BACE-1 IN COMPLEX WITH LIGAND 32397778 [Homo sapiens]

Sequence ID: 5MXD_B Length: 432 >BACE-1 IN COMPLEX WITH LIGAND 32397778 [Homo sapiens]

Sequence ID: 5MXD_C Length: 432 >Structure of Bace-1 in complex with Ligand 8 [Homo sapiens]

Sequence ID: 6E3Z_A Length: 432 >Structure of Bace-1 in complex with Ligand 8 [Homo sapiens]

Sequence ID: 6E3Z_B Length: 432 >Structure of Bace-1 in complex with Ligand 8 [Homo sapiens]

Sequence ID: 6E3Z_C Length: 432 >Structure of BACE-1 in complex with Ligand 13 [Homo sapiens]

Sequence ID: 6OD6_A Length: 432 >Structure of BACE-1 in complex with Ligand 13 [Homo sapiens]

Sequence ID: 6OD6_B Length: 432 >Structure of BACE-1 in complex with Ligand 13 [Homo sapiens]

Sequence ID: 6OD6_C Length: 432

Range 1: 61 to 328

Score:56.2 bits(134), Expect:2e-07,

Method:Compositional matrix adjust.,

Identities:77/289(27%), Positives:117/289(40%), Gaps:58/289(20%)

Query 96 YSANFTVGSNSQKQNVIVDTGSSDLWVVDSSANCQEKSGYSSDYCFSGGTYDPSSSSTIQ 155

Y TVGS Q N++VDTGSS+ V ++ + F Y SST +

Sbjct 61 YYVEMTVGSPPQTLNILVDTGSSNFAVG------------AAPHPFLHRYYQRQLSSTYR 108

Query 156 ELGKSFNIRYGDGSSSSGTWVKD--------------TVGINGAIILNQQFGDVNSTSVS 201

+L K + Y + G W + TV N A I +N ++

Sbjct 109 DLRKGVYVPY-----TQGKWEGELGTDLVSIPHGPNVTVRANIAAITESDKFFINGSNW- 162

Query 202 QGILGIGLDTNESTDTIYENFPINLKEQGFINTNAYSLYLNAP----------SATSGTI 251

+GILG+ D E F +L +Q + N +SL L ++ G++

Sbjct 163 EGILGLAYAEIARPDDSLEPFFDSLVKQTHV-PNLFSLQLCGAGFPLNQSEVLASVGGSM 221

Query 252 IFGGIDHAKYTGSLTTLPLTSNREFTIQTNSATVGTSTIDINTGL--------LLDSGTT 303

I GGIDH+ YTGSL P+ RE+ + V + D+ ++DSGTT

Sbjct 222 IIGGIDHSLYTGSLWYTPI--RREWYYEVIIVRVEINGQDLKMDCKEYNYDKSIVDSGTT 279

Query 304 LTYLPQSVVDSIANAI-GGDITYNRPIGAYIWS---CNRNGKVTYN-FP 347

LP+ V ++ +I T P G ++ C + G +N FP

Sbjct 280 NLRLPKKVFEAAVKSIKAASSTEKFPDGFWLGEQLVCWQAGTTPWNIFP 328

>Crystal structure of BACE1 complexed with an inhibitor [Homo sapiens]

Sequence ID: 3TPR_A Length: 433

>Crystal structure of BACE1 with its inhibitor [Homo sapiens]

Sequence ID: 3UQR_A Length: 433 >Crystal structure of BACE1 with its inhibitor [Homo sapiens]

Sequence ID: 3UQR_B Length: 433 >Crystal structure of BACE1 with its inhibitor [Homo sapiens]

Sequence ID: 3UQR_C Length: 433

Range 1: 54 to 321

Score:56.2 bits(134), Expect:2e-07,

Method:Compositional matrix adjust.,

Identities:77/289(27%), Positives:117/289(40%), Gaps:58/289(20%)

Query 96 YSANFTVGSNSQKQNVIVDTGSSDLWVVDSSANCQEKSGYSSDYCFSGGTYDPSSSSTIQ 155

Y TVGS Q N++VDTGSS+ V ++ + F Y SST +

Sbjct 54 YYVEMTVGSPPQTLNILVDTGSSNFAVG------------AAPHPFLHRYYQRQLSSTYR 101

Query 156 ELGKSFNIRYGDGSSSSGTWVKD--------------TVGINGAIILNQQFGDVNSTSVS 201

+L K + Y + G W + TV N A I +N ++

Sbjct 102 DLRKGVYVPY-----TQGKWEGELGTDLVSIPHGPNVTVRANIAAITESDKFFINGSNW- 155

Query 202 QGILGIGLDTNESTDTIYENFPINLKEQGFINTNAYSLYL----------NAPSATSGTI 251

+GILG+ D E F +L +Q + N +SL L ++ G++

Sbjct 156 EGILGLAYAEIARPDDSLEPFFDSLVKQTHV-PNLFSLQLCGAGFPLNQSEVLASVGGSM 214

Query 252 IFGGIDHAKYTGSLTTLPLTSNREFTIQTNSATVGTSTIDINTGL--------LLDSGTT 303

I GGIDH+ YTGSL P+ RE+ + V + D+ ++DSGTT

Sbjct 215 IIGGIDHSLYTGSLWYTPI--RREWYYEVIIVRVEINGQDLKMDCKEYNYDKSIVDSGTT 272

Query 304 LTYLPQSVVDSIANAI-GGDITYNRPIGAYIWS---CNRNGKVTYN-FP 347

LP+ V ++ +I T P G ++ C + G +N FP

Sbjct 273 NLRLPKKVFEAAVKSIKAASSTEKFPDGFWLGEQLVCWQAGTTPWNIFP 321

>BACE-1 complexed with compound 1 [Homo sapiens]

Sequence ID: 3BRA_A Length: 409

>BACE-1 complexed with compound 2 [Homo sapiens]

Sequence ID: 3BUF_A Length: 409 >BACE-1 complexed with compound 3 [Homo sapiens]

Sequence ID: 3BUG_A Length: 409 >BACE-1 complexed with compound 4 [Homo sapiens]

Sequence ID: 3BUH_A Length: 409 >CRYSTAL STRUCTURE OF BACE-1 IN COMPLEX WITH CHEMICAL LIGAND [Homo sapiens]

Sequence ID: 3ZMG_A Length: 409 >CRYSTAL STRUCTURE OF BACE-1 IN COMPLEX WITH CHEMICAL LIGAND [Homo sapiens]

Sequence ID: 3ZOV_A Length: 409 >CRYSTAL STRUCTURE OF BACE-1 IN COMPLEX WITH CHEMICAL LIGAND [Homo sapiens]

Sequence ID: 4BEK_A Length: 409 >CRYSTAL STRUCTURE OF BACE-1 IN COMPLEX WITH CHEMICAL LIGAND [Homo sapiens]

Sequence ID: 4BFD_A Length: 409 >CRYSTAL STRUCTURE OF BACE-1 IN COMPLEX WITH 5-Cyano-pyridine-2-carboxylic acid [3-((S)-2-amino-4-methyl-5,6-dihydro-4H-[1,3]oxazin-4-yl)-4-fluoro-phenyl]-amide [Homo sapiens]

Sequence ID: 4J0P_A Length: 409 >CRYSTAL STRUCTURE OF BACE-1 IN COMPLEX WITH 5-Ethoxy-pyridine-2-carboxylic acid [3-((R)-2-amino-5,5-difluoro-4-methyl-5,6-dihydro-4H-[1,3]oxazin-4-yl)-4-fluoro-phenyl]-amide [Homo sapiens]

Sequence ID: 4J0T_A Length: 409 >CRYSTAL STRUCTURE OF BACE-1 IN COMPLEX WITH 5-Cyano-pyridine-2-carboxylic acid [3-((4R,5R)-2-amino-5-fluoro-4-methyl-5,6-dihydro-4H-[1,3]oxazin-4-yl)-4-fluoro-phenyl]-amide [Homo sapiens]

Sequence ID: 4J0V_A Length: 409 >CRYSTAL STRUCTURE OF BACE-1 IN COMPLEX WITH 5-Cyano-pyridine-2-carboxylic acid [3-((4R,5S)-2-amino-5-fluoro-4-methyl-5,6-dihydro-4H-[1,3]oxazin-4-yl)-4-fluoro-phenyl]-amide [Homo sapiens]

Sequence ID: 4J0Y_A Length: 409 >CRYSTAL STRUCTURE OF BACE-1 IN COMPLEX WITH 5-Cyano-pyridine-2-carboxylic acid [3-((4S,5R)-2-amino-5-fluoro-4-fluoromethyl-5,6-dihydro-4H-[1,3]oxazin-4-yl)-4-fluoro-phenyl]-amide [Homo sapiens]

Sequence ID: 4J0Z_A Length: 409 >CRYSTAL STRUCTURE OF BACE-1 IN COMPLEX WITH 5-Cyano-pyridine-2-carboxylic acid [3-((S)-2-amino-4-difluoromethyl-5,6-dihydro-4H-[1,3]oxazin-4-yl)-4-fluoro-phenyl]-amide [Homo sapiens]

Sequence ID: 4J17_A Length: 409 >CRYSTAL STRUCTURE OF BACE-1 IN COMPLEX WITH 5-Cyano-pyridine-2-carboxylic acid [3-((S)-2-amino-5,5-difluoro-4-fluoromethyl-5,6-dihydro-4H-[1,3]oxazin-4-yl)-4-fluoro-phenyl]-amide [Homo sapiens]

Sequence ID: 4J1C_A Length: 409 >CRYSTAL STRUCTURE OF BACE-1 IN COMPLEX WITH 5-Cyano-pyridine-2-carboxylic acid [3-((4S,6S)-2-amino-4-fluoromethyl-6-trifluoromethyl-5,6-dihydro-4H-[1,3]oxazin-4-yl)-4-fluoro-phenyl]-amide [Homo sapiens]

Sequence ID: 4J1E_A Length: 409 >CRYSTAL STRUCTURE OF BACE-1 IN COMPLEX WITH 5-Cyano-pyridine-2-carboxylic acid [3-((4S,6S)-2-amino-4-methyl-6-trifluoromethyl-5,6-dihydro-4H-[1,3]oxazin-4-yl)-4-fluoro-phenyl]-amide [Homo sapiens]

Sequence ID: 4J1F_A Length: 409 >CRYSTAL STRUCTURE OF BACE-1 IN COMPLEX WITH 5-Cyano-pyridine-2-carboxylic acid [3-((4S,6R)-2-amino-4-methyl-6-trifluoromethyl-5,6-dihydro-4H-[1,3]oxazin-4-yl)-4-fluoro-phenyl]-amide [Homo sapiens]

Sequence ID: 4J1H_A Length: 409 >CRYSTAL STRUCTURE OF BACE-1 IN COMPLEX WITH 5-Cyano-pyridine-2-carboxylic acid [3-((4R,5R,6R)-2-amino-5-fluoro-4-methyl-6-trifluoromethyl-5,6-dihydro-4H-[1,3]oxazin-4-yl)-4-fluoro-phenyl]-amide [Homo sapiens]

Sequence ID: 4J1I_A Length: 409 >CRYSTAL STRUCTURE OF BACE-1 IN COMPLEX WITH 5-Cyano-pyridine-2-carboxylic acid [3-((4R,5R,6S)-2-amino-5-fluoro-4-methyl-6-trifluoromethyl-5,6-dihydro-4H-[1,3]oxazin-4-yl)-4-fluoro-phenyl]-amide [Homo sapiens]

Sequence ID: 4J1K_A Length: 409 >CRYSTAL STRUCTURE OF BACE-1 IN COMPLEX WITH ACTIVE SITE INHIBITOR GRL-8234 AND EXOSITE PEPTIDE [Homo sapiens]

Sequence ID: 5MCO_A Length: 409

Range 1: 30 to 297

Score:56.2 bits(134), Expect:2e-07,

Method:Compositional matrix adjust.,

Identities:77/289(27%), Positives:118/289(40%), Gaps:58/289(20%)

Query 96 YSANFTVGSNSQKQNVIVDTGSSDLWVVDSSANCQEKSGYSSDYCFSGGTYDPSSSSTIQ 155

Y TVGS Q N++VDTGSS+ V ++ + F Y SST +

Sbjct 30 YYVEMTVGSPPQTLNILVDTGSSNFAVG------------AAPHPFLHRYYQRQLSSTYR 77

Query 156 ELGKSFNIRYGDGSSSSGTWVKD--------------TVGINGAIILNQQFGDVNSTSVS 201

+L K + Y + G W + TV N A I +N ++

Sbjct 78 DLRKGVYVPY-----TQGKWEGELGTDLVSIPHGPNVTVRANIAAITESDKFFINGSNW- 131

Query 202 QGILGIGLDTNESTDTIYENFPINLKEQGFINTNAYSLYL----------NAPSATSGTI 251

+GILG+ D E F +L +Q + N +SL L ++ G++

Sbjct 132 EGILGLAYAEIARPDDSLEPFFDSLVKQTHV-PNLFSLQLCGAGFPLNQSEVLASVGGSM 190

Query 252 IFGGIDHAKYTGSLTTLPLTSNREFTIQTNSATVGTSTIDINTGL--------LLDSGTT 303

I GGIDH+ YTGSL P+ RE+ + V + D+ ++DSGTT

Sbjct 191 IIGGIDHSLYTGSLWYTPI--RREWYYEVIIVRVEINGQDLKMDCKEYNYDKSIVDSGTT 248

Query 304 LTYLPQSVVD-SIANAIGGDITYNRPIGAYIWS---CNRNGKVTYN-FP 347

LP+ V + ++A+ T P G ++ C + G +N FP

Sbjct 249 NLRLPKKVFEAAVASIKAASSTEKFPDGFWLGEQLVCWQAGTTPWNIFP 297

>CRYSTAL STRUCTURE OF BACE-1 IN COMPLEX WITH Pep#3 [Homo sapiens]

Sequence ID: 5MBW_A Length: 409

>CRYSTAL STRUCTURE OF BACE-1 IN COMPLEX WITH ACTIVE SITE AND EXOSITE BINDING PEPTIDE INHIBITOR [Homo sapiens]

Sequence ID: 5MCQ_A Length: 409

Range 1: 30 to 297

Score:56.2 bits(134), Expect:2e-07,

Method:Compositional matrix adjust.,

Identities:77/289(27%), Positives:117/289(40%), Gaps:58/289(20%)

Query 96 YSANFTVGSNSQKQNVIVDTGSSDLWVVDSSANCQEKSGYSSDYCFSGGTYDPSSSSTIQ 155

Y TVGS Q N++VDTGSS+ V ++ + F Y SST +

Sbjct 30 YYVEMTVGSPPQTLNILVDTGSSNFAVG------------AAPHPFLHRYYQRQLSSTYR 77

Query 156 ELGKSFNIRYGDGSSSSGTWVKD--------------TVGINGAIILNQQFGDVNSTSVS 201

+L K + Y + G W + TV N A I +N ++

Sbjct 78 DLRKGVYVPY-----TQGKWEGELGTDLVSIPHGPNVTVRANIAAITESDKFFINGSNW- 131

Query 202 QGILGIGLDTNESTDTIYENFPINLKEQGFINTNAYSLYL----------NAPSATSGTI 251

+GILG+ D E F +L +Q + N +SL L ++ G++

Sbjct 132 EGILGLAYAEIARPDDSLEPFFDSLVKQTHV-PNLFSLQLCGAGFPLNQSEVLASVGGSM 190

Query 252 IFGGIDHAKYTGSLTTLPLTSNREFTIQTNSATVGTSTIDINTGL--------LLDSGTT 303

I GGIDH+ YTGSL P+ RE+ + V + D+ ++DSGTT

Sbjct 191 IIGGIDHSLYTGSLWYTPI--RREWYYEVIIVRVEINGQDLKMDCKEYNYDKSIVDSGTT 248

Query 304 LTYLPQSVVDSIANAI-GGDITYNRPIGAYIWS---CNRNGKVTYN-FP 347

LP+ V ++ +I T P G ++ C + G +N FP

Sbjct 249 NLRLPKKVFEAAVKSIKAASSTEKFPDGFWLGEQLVCWQAGTTPWNIFP 297

>1,4-Oxazine BACE1 inhibitors [Homo sapiens]

Sequence ID: 5CLM_A Length: 401

Range 1: 30 to 297

Score:56.2 bits(134), Expect:2e-07,

Method:Compositional matrix adjust.,

Identities:77/289(27%), Positives:117/289(40%), Gaps:58/289(20%)

Query 96 YSANFTVGSNSQKQNVIVDTGSSDLWVVDSSANCQEKSGYSSDYCFSGGTYDPSSSSTIQ 155

Y TVGS Q N++VDTGSS+ V ++ + F Y SST +

Sbjct 30 YYVEMTVGSPPQTLNILVDTGSSNFAVG------------AAPHPFLHRYYQRQLSSTYR 77

Query 156 ELGKSFNIRYGDGSSSSGTWVKD--------------TVGINGAIILNQQFGDVNSTSVS 201

+L K + Y + G W + TV N A I +N ++

Sbjct 78 DLRKGVYVPY-----TQGKWEGELGTDLVSIPHGPNVTVRANIAAITESDKFFINGSNW- 131

Query 202 QGILGIGLDTNESTDTIYENFPINLKEQGFINTNAYSLYL----------NAPSATSGTI 251

+GILG+ D E F +L +Q + N +SL L ++ G++

Sbjct 132 EGILGLAYAEIARPDDSLEPFFDSLVKQTHV-PNLFSLQLCGAGFPLNQSEVLASVGGSM 190

Query 252 IFGGIDHAKYTGSLTTLPLTSNREFTIQTNSATVGTSTIDINTGL--------LLDSGTT 303

I GGIDH+ YTGSL P+ RE+ + V + D+ ++DSGTT

Sbjct 191 IIGGIDHSLYTGSLWYTPI--RREWYYEVIIVRVEINGQDLKMDCKEYNYDKSIVDSGTT 248

Query 304 LTYLPQSVVDSIANAI-GGDITYNRPIGAYIWS---CNRNGKVTYN-FP 347

LP+ V ++ +I T P G ++ C + G +N FP

Sbjct 249 NLRLPKKVFEAAVKSIKAASSTEKFPDGFWLGEQLVCWQAGTTPWNIFP 297

>Structure Basis of Allosteric Inhibition of BACE1 by an Exosite-Binding Antibody [Homo sapiens]

Sequence ID: 3R1G_B Length: 402

Range 1: 21 to 288

Score:56.2 bits(134), Expect:2e-07,

Method:Compositional matrix adjust.,

Identities:77/289(27%), Positives:117/289(40%), Gaps:58/289(20%)

Query 96 YSANFTVGSNSQKQNVIVDTGSSDLWVVDSSANCQEKSGYSSDYCFSGGTYDPSSSSTIQ 155

Y TVGS Q N++VDTGSS+ V ++ + F Y SST +

Sbjct 21 YYVEMTVGSPPQTLNILVDTGSSNFAVG------------AAPHPFLHRYYQRQLSSTYR 68

Query 156 ELGKSFNIRYGDGSSSSGTWVKD--------------TVGINGAIILNQQFGDVNSTSVS 201

+L K + Y + G W + TV N A I +N ++

Sbjct 69 DLRKGVYVPY-----TQGKWEGELGTDLVSIPHGPNVTVRANIAAITESDKFFINGSNW- 122

Query 202 QGILGIGLDTNESTDTIYENFPINLKEQGFINTNAYSLYLNAP----------SATSGTI 251

+GILG+ D E F +L +Q + N +SL L ++ G++

Sbjct 123 EGILGLAYAEIARPDDSLEPFFDSLVKQTHV-PNLFSLQLCGAGFPLNQSEVLASVGGSM 181

Query 252 IFGGIDHAKYTGSLTTLPLTSNREFTIQTNSATVGTSTIDINTGL--------LLDSGTT 303

I GGIDH+ YTGSL P+ RE+ + V + D+ ++DSGTT

Sbjct 182 IIGGIDHSLYTGSLWYTPI--RREWYYEVIIVRVEINGQDLKMDCKEYNYDKSIVDSGTT 239

Query 304 LTYLPQSVVDSIANAI-GGDITYNRPIGAYIWS---CNRNGKVTYN-FP 347

LP+ V ++ +I T P G ++ C + G +N FP

Sbjct 240 NLRLPKKVFEAAVKSIKAASSTEKFPDGFWLGEQLVCWQAGTTPWNIFP 288

>BACE1 with Compound 1 [Homo sapiens]

Sequence ID: 2QU2_A Length: 415

>BACE1 with Compound 2 [Homo sapiens]

Sequence ID: 2QU3_A Length: 415 >X-ray structure of Bace-1 in complex with compound 3.b.10 [Homo sapiens]

Sequence ID: 2ZDZ_A Length: 415 >X-ray structure of Bace-1 in complex with compound 6g [Homo sapiens]

Sequence ID: 2ZE1_A Length: 415 >Bace-1 with Compound 3 [Homo sapiens]

Sequence ID: 3IGB_A Length: 415 >Bace1 with Compound 30 [Homo sapiens]

Sequence ID: 3IN3_A Length: 415 >Bace1 with Compound 38 [Homo sapiens]

Sequence ID: 3IN4_A Length: 415 >Bace1 with the aminohydantoin Compound 29 [Homo sapiens]

Sequence ID: 3IND_A Length: 415 >Bace1 with the aminohydantoin Compound S-34 [Homo sapiens]

Sequence ID: 3INE_A Length: 415 >Bace1 with the aminohydantoin Compound 37 [Homo sapiens]

Sequence ID: 3INF_A Length: 415 >Bace1 with the aminohydantoin Compound R-58 [Homo sapiens]

Sequence ID: 3INH_A Length: 415 >Bace1 in complex with the aminopyridine Compound 44 [Homo sapiens]

Sequence ID: 3L38_A Length: 415 >Bace-1 with the aminopyridine Compound 32 [Homo sapiens]

Sequence ID: 3L3A_A Length: 415 >Bace1 in complex with the aminohydantoin Compound 4g [Homo sapiens]

Sequence ID: 3LHG_A Length: 415 >Bace1 in complex with the aminohydantoin Compound 102 [Homo sapiens]

Sequence ID: 3OOZ_A Length: 415 >Pyrazolyl and Thienyl Aminohydantoins as Potent BACE1 Inhibitors [Homo sapiens]

Sequence ID: 3S7L_A Length: 415 >Pyrazolyl and Thienyl Aminohydantoins as Potent BACE1 Inhibitors [Homo sapiens]

Sequence ID: 3S7M_A Length: 415 >Crystal structure of human BACE-1 bound to Compound 36 [Homo sapiens]

Sequence ID: 4WY6_A Length: 415 >Crystal structure of human BACE-1 bound to Compound 6 [Homo sapiens]

Sequence ID: 4X2L_A Length: 415 >Crystal structure of BACE1 with a pyrazole-substituted tetrahydropyran thioamidine [Homo sapiens]

Sequence ID: 4XXS_A Length: 415 >Aminomethyl-Derived Beta Secretase (BACE1) Inhibitors: Engaging Gly230 without an Anilide Functionality [Homo sapiens]

Sequence ID: 5T1U_A Length: 415 >Aminomethyl-Derived Beta Secretase (BACE1) Inhibitors: Engaging Gly230 without an Anilide Functionality [Homo sapiens]

Sequence ID: 5T1W_A Length: 415

Range 1: 30 to 297

Score:56.2 bits(134), Expect:2e-07,

Method:Compositional matrix adjust.,

Identities:77/289(27%), Positives:117/289(40%), Gaps:58/289(20%)

Query 96 YSANFTVGSNSQKQNVIVDTGSSDLWVVDSSANCQEKSGYSSDYCFSGGTYDPSSSSTIQ 155

Y TVGS Q N++VDTGSS+ V ++ + F Y SST +

Sbjct 30 YYVEMTVGSPPQTLNILVDTGSSNFAVG------------AAPHPFLHRYYQRQLSSTYR 77

Query 156 ELGKSFNIRYGDGSSSSGTWVKD--------------TVGINGAIILNQQFGDVNSTSVS 201

+L K + Y + G W + TV N A I +N ++

Sbjct 78 DLRKGVYVPY-----TQGKWEGELGTDLVSIPHGPNVTVRANIAAITESDKFFINGSNW- 131

Query 202 QGILGIGLDTNESTDTIYENFPINLKEQGFINTNAYSLYL----------NAPSATSGTI 251

+GILG+ D E F +L +Q + N +SL L ++ G++

Sbjct 132 EGILGLAYAEIARPDDSLEPFFDSLVKQTHV-PNLFSLQLCGAGFPLNQSEVLASVGGSM 190

Query 252 IFGGIDHAKYTGSLTTLPLTSNREFTIQTNSATVGTSTIDINTGL--------LLDSGTT 303

I GGIDH+ YTGSL P+ RE+ + V + D+ ++DSGTT

Sbjct 191 IIGGIDHSLYTGSLWYTPI--RREWYYEVIIVRVEINGQDLKMDCKEYNYDKSIVDSGTT 248

Query 304 LTYLPQSVVDSIANAI-GGDITYNRPIGAYIWS---CNRNGKVTYN-FP 347

LP+ V ++ +I T P G ++ C + G +N FP

Sbjct 249 NLRLPKKVFEAAVKSIKAASSTEKFPDGFWLGEQLVCWQAGTTPWNIFP 297

>Crystal structure of human beta secretase complexed with NVP-AMK640 [Homo sapiens]

Sequence ID: 1YM4_A Length: 408

>Crystal structure of human beta secretase complexed with NVP-AMK640 [Homo sapiens]

Sequence ID: 1YM4_B Length: 408 >Crystal structure of human beta secretase complexed with NVP-AMK640 [Homo sapiens]

Sequence ID: 1YM4_C Length: 408 >Crystal Structure of BACE-1 in complex with AHM178 [Homo sapiens]

Sequence ID: 3K5D_A Length: 408 >Crystal Structure of BACE-1 in complex with AHM178 [Homo sapiens]

Sequence ID: 3K5D_B Length: 408 >Crystal Structure of BACE-1 in complex with AHM178 [Homo sapiens]

Sequence ID: 3K5D_C Length: 408 >Fragment Based Discovery and Optimisation of BACE-1 Inhibitors [Homo sapiens]

Sequence ID: 3MSK_A Length: 408 >Fragment Based Discovery and Optimisation of BACE-1 Inhibitors [Homo sapiens]

Sequence ID: 3MSL_A Length: 408 >Fragment based discovery and optimisation of bace-1 inhibitors [Homo sapiens]

Sequence ID: 3S2O_A Length: 408 >Crystal Structure of Human Beta Secretase in Complex with NVP-BVI151 [Homo sapiens]

Sequence ID: 4D85_A Length: 408

Range 1: 30 to 297

Score:56.2 bits(134), Expect:2e-07,

Method:Compositional matrix adjust.,

Identities:77/289(27%), Positives:117/289(40%), Gaps:58/289(20%)

Query 96 YSANFTVGSNSQKQNVIVDTGSSDLWVVDSSANCQEKSGYSSDYCFSGGTYDPSSSSTIQ 155

Y TVGS Q N++VDTGSS+ V ++ + F Y SST +

Sbjct 30 YYVEMTVGSPPQTLNILVDTGSSNFAVG------------AAPHPFLHRYYQRQLSSTYR 77

Query 156 ELGKSFNIRYGDGSSSSGTWVKD--------------TVGINGAIILNQQFGDVNSTSVS 201

+L K + Y + G W + TV N A I +N ++

Sbjct 78 DLRKGVYVPY-----TQGKWEGELGTDLVSIPHGPNVTVRANIAAITESDKFFINGSNW- 131

Query 202 QGILGIGLDTNESTDTIYENFPINLKEQGFINTNAYSLYL----------NAPSATSGTI 251

+GILG+ D E F +L +Q + N +SL L ++ G++

Sbjct 132 EGILGLAYAEIARPDDSLEPFFDSLVKQTHV-PNLFSLQLCGAGFPLNQSEVLASVGGSM 190

Query 252 IFGGIDHAKYTGSLTTLPLTSNREFTIQTNSATVGTSTIDINTGL--------LLDSGTT 303

I GGIDH+ YTGSL P+ RE+ + V + D+ ++DSGTT

Sbjct 191 IIGGIDHSLYTGSLWYTPI--RREWYYEVIIVRVEINGQDLKMDCKEYNYDKSIVDSGTT 248

Query 304 LTYLPQSVVDSIANAI-GGDITYNRPIGAYIWS---CNRNGKVTYN-FP 347

LP+ V ++ +I T P G ++ C + G +N FP

Sbjct 249 NLRLPKKVFEAAVKSIKAASSTEKFPDGFWLGEQLVCWQAGTTPWNIFP 297

>Diethylaminosulfur Trifluoride-Mediated Intramolecular Cyclization of 2-hydroxy-benzylureas to Fused Bicyclic Aminooxazoline Compounds and Evaluation of Their Biochemical Activity Against Beta-Secretase-1 (BACE1) [Homo sapiens]

Sequence ID: 4L7G_A Length: 409

>Diethylaminosulfur Trifluoride-Mediated Intramolecular Cyclization of 2-hydroxy-benzylureas to Fused Bicyclic Aminooxazoline Compounds and Evaluation of Their Biochemical Activity Against Beta-Secretase-1 (BACE-1) [Homo sapiens]

Sequence ID: 4L7H_A Length: 409 >Diethylaminosulfur Trifluoride-Mediated Intramolecular Cyclization of 2-hydroxy-benzylureas to Fused Bicyclic Aminooxazoline Compounds and Evaluation of Their Biochemical Activity Against Beta-Secretase-1 (BACE-1) [Homo sapiens]

Sequence ID: 4L7J_A Length: 409

Range 1: 22 to 289

Score:56.2 bits(134), Expect:2e-07,

Method:Compositional matrix adjust.,

Identities:77/289(27%), Positives:117/289(40%), Gaps:58/289(20%)

Query 96 YSANFTVGSNSQKQNVIVDTGSSDLWVVDSSANCQEKSGYSSDYCFSGGTYDPSSSSTIQ 155

Y TVGS Q N++VDTGSS+ V ++ + F Y SST +

Sbjct 22 YYVEMTVGSPPQTLNILVDTGSSNFAVG------------AAPHPFLHRYYQRQLSSTYR 69

Query 156 ELGKSFNIRYGDGSSSSGTWVKD--------------TVGINGAIILNQQFGDVNSTSVS 201

+L K + Y + G W + TV N A I +N ++

Sbjct 70 DLRKGVYVPY-----TQGKWEGELGTDLVSIPHGPNVTVRANIAAITESDKFFINGSNW- 123

Query 202 QGILGIGLDTNESTDTIYENFPINLKEQGFINTNAYSLYLNAP----------SATSGTI 251

+GILG+ D E F +L +Q + N +SL L ++ G++

Sbjct 124 EGILGLAYAEIARPDDSLEPFFDSLVKQTHV-PNLFSLQLCGAGFPLNQSEVLASVGGSM 182

Query 252 IFGGIDHAKYTGSLTTLPLTSNREFTIQTNSATVGTSTIDINTGL--------LLDSGTT 303

I GGIDH+ YTGSL P+ RE+ + V + D+ ++DSGTT

Sbjct 183 IIGGIDHSLYTGSLWYTPI--RREWYYEVIIVRVEINGQDLKMDCKEYNYDKSIVDSGTT 240

Query 304 LTYLPQSVVDSIANAI-GGDITYNRPIGAYIWS---CNRNGKVTYN-FP 347

LP+ V ++ +I T P G ++ C + G +N FP

Sbjct 241 NLRLPKKVFEAAVKSIKAASSTEKFPDGFWLGEQLVCWQAGTTPWNIFP 289

>Crystal Structure of BACE with amino thiazine inhibitor LY2886721 [Homo sapiens]

Sequence ID: 4X7I_A Length: 442

>Crystal Structure of BACE with amino thiazine inhibitor LY2886721 [Homo sapiens]

Sequence ID: 4X7I_B Length: 442 >Crystal structure of BACE with amino thiazine inhibitor LY2811376 [Homo sapiens]

Sequence ID: 4YBI_A Length: 442 >Crystal structure of BACE with amino thiazine inhibitor LY2811376 [Homo sapiens]

Sequence ID: 4YBI_B Length: 442 >BACE crystal structure with bicyclic aminothiazine fragment [Homo sapiens]

Sequence ID: 4ZSM_A Length: 442 >BACE crystal structure with bicyclic aminothiazine fragment [Homo sapiens]

Sequence ID: 4ZSM_B Length: 442 >BACE crystal structure with bicyclic aminothiazine inhibitor [Homo sapiens]

Sequence ID: 4ZSP_A Length: 442 >BACE crystal structure with bicyclic aminothiazine inhibitor [Homo sapiens]

Sequence ID: 4ZSP_B Length: 442 >BACE crystal structure with tricyclic aminothiazine inhibitor [Homo sapiens]

Sequence ID: 4ZSQ_A Length: 442 >BACE crystal structure with tricyclic aminothiazine inhibitor [Homo sapiens]

Sequence ID: 4ZSQ_B Length: 442 >BACE crystal structure with tricyclic aminothiazine inhibitor [Homo sapiens]

Sequence ID: 4ZSR_A Length: 442 >BACE crystal structure with tricyclic aminothiazine inhibitor [Homo sapiens]

Sequence ID: 4ZSR_B Length: 442 >BACE crystal structure with hydroxy pyrrolidine inhibitor [Homo sapiens]

Sequence ID: 6BFD_A Length: 442 >BACE crystal structure with hydroxy pyrrolidine inhibitor [Homo sapiens]

Sequence ID: 6BFD_B Length: 442 >BACE crystal structure with hydroxy pyrrolidine inhibitor [Homo sapiens]

Sequence ID: 6BFE_A Length: 442 >BACE crystal structure with hydroxy pyrrolidine inhibitor [Homo sapiens]

Sequence ID: 6BFE_B Length: 442 >BACE crystal structure with hydroxy morpholine inhibitor [Homo sapiens]

Sequence ID: 6BFW_A Length: 442 >BACE crystal structure with hydroxy morpholine inhibitor [Homo sapiens]

Sequence ID: 6BFW_B Length: 442 >BACE crystal structure with hydroxy pyrrolidine inhibitor [Homo sapiens]

Sequence ID: 6BFX_A Length: 442 >BACE crystal structure with hydroxy pyrrolidine inhibitor [Homo sapiens]

Sequence ID: 6BFX_B Length: 442 >BACE-1 in complex with compound #3 [Homo sapiens]

Sequence ID: 6UVP_A Length: 442 >BACE-1 in complex with compound #3 [Homo sapiens]

Sequence ID: 6UVP_B Length: 442 >BACE-1 in complex with compound #17 [Homo sapiens]

Sequence ID: 6UVV_A Length: 442 >BACE-1 in complex with compound #17 [Homo sapiens]

Sequence ID: 6UVV_B Length: 442 >BACE-1 in complex with compound #18 [Homo sapiens]

Sequence ID: 6UVY_A Length: 442 >BACE-1 in complex with compound #18 [Homo sapiens]

Sequence ID: 6UVY_B Length: 442 >BACE-1 in complex with compound #32 [Homo sapiens]

Sequence ID: 6UWP_A Length: 442 >BACE-1 in complex with compound #32 [Homo sapiens]

Sequence ID: 6UWP_B Length: 442 >BACE-1 in complex with compound #34 [Homo sapiens]

Sequence ID: 6UWV_A Length: 442 >BACE-1 in complex with compound #34 [Homo sapiens]

Sequence ID: 6UWV_B Length: 442

Range 1: 63 to 330

Score:56.2 bits(134), Expect:2e-07,

Method:Compositional matrix adjust.,

Identities:77/289(27%), Positives:117/289(40%), Gaps:58/289(20%)

Query 96 YSANFTVGSNSQKQNVIVDTGSSDLWVVDSSANCQEKSGYSSDYCFSGGTYDPSSSSTIQ 155

Y TVGS Q N++VDTGSS+ V ++ + F Y SST +

Sbjct 63 YYVEMTVGSPPQTLNILVDTGSSNFAVG------------AAPHPFLHRYYQRQLSSTYR 110

Query 156 ELGKSFNIRYGDGSSSSGTWVKD--------------TVGINGAIILNQQFGDVNSTSVS 201

+L K + Y + G W + TV N A I +N ++

Sbjct 111 DLRKGVYVPY-----TQGKWEGELGTDLVSIPHGPNVTVRANIAAITESDKFFINGSNW- 164

Query 202 QGILGIGLDTNESTDTIYENFPINLKEQGFINTNAYSLYLNAP----------SATSGTI 251

+GILG+ D E F +L +Q + N +SL L ++ G++

Sbjct 165 EGILGLAYAEIARPDDSLEPFFDSLVKQTHV-PNLFSLQLCGAGFPLNQSEVLASVGGSM 223

Query 252 IFGGIDHAKYTGSLTTLPLTSNREFTIQTNSATVGTSTIDINTGL--------LLDSGTT 303

I GGIDH+ YTGSL P+ RE+ + V + D+ ++DSGTT

Sbjct 224 IIGGIDHSLYTGSLWYTPI--RREWYYEVIIVRVEINGQDLKMDCKEYNYDKSIVDSGTT 281

Query 304 LTYLPQSVVDSIANAI-GGDITYNRPIGAYIWS---CNRNGKVTYN-FP 347

LP+ V ++ +I T P G ++ C + G +N FP

Sbjct 282 NLRLPKKVFEAAVKSIKAASSTEKFPDGFWLGEQLVCWQAGTTPWNIFP 330

>BACE1 in complex with inhibitor 5g [Homo sapiens]

Sequence ID: 5V0N_A Length: 441

>BACE1 in complex with inhibitor 5g [Homo sapiens]

Sequence ID: 5V0N_B Length: 441 >BACE1 in complex with inhibitor 5g [Homo sapiens]

Sequence ID: 5V0N_C Length: 441 >X-ray structure of BACE1 in complex with a bicyclic isoxazoline carboxamide as the P3 ligand [Homo sapiens]

Sequence ID: 6DHC_A Length: 441 >X-ray structure of BACE1 in complex with a bicyclic isoxazoline carboxamide as the P3 ligand [Homo sapiens]

Sequence ID: 6DHC_B Length: 441 >X-ray structure of BACE1 in complex with a bicyclic isoxazoline carboxamide as the P3 ligand [Homo sapiens]

Sequence ID: 6DHC_C Length: 441

Range 1: 62 to 329

Score:56.2 bits(134), Expect:2e-07,

Method:Compositional matrix adjust.,

Identities:77/289(27%), Positives:117/289(40%), Gaps:58/289(20%)

Query 96 YSANFTVGSNSQKQNVIVDTGSSDLWVVDSSANCQEKSGYSSDYCFSGGTYDPSSSSTIQ 155

Y TVGS Q N++VDTGSS+ V ++ + F Y SST +

Sbjct 62 YYVEMTVGSPPQTLNILVDTGSSNFAVG------------AAPHPFLHRYYQRQLSSTYR 109

Query 156 ELGKSFNIRYGDGSSSSGTWVKD--------------TVGINGAIILNQQFGDVNSTSVS 201

+L K + Y + G W + TV N A I +N ++

Sbjct 110 DLRKGVYVPY-----TQGKWEGELGTDLVSIPHGPNVTVRANIAAITESDKFFINGSNW- 163

Query 202 QGILGIGLDTNESTDTIYENFPINLKEQGFINTNAYSLYLNAP----------SATSGTI 251

+GILG+ D E F +L +Q + N +SL L ++ G++

Sbjct 164 EGILGLAYAEIARPDDSLEPFFDSLVKQTHV-PNLFSLQLCGAGFPLNQSEVLASVGGSM 222

Query 252 IFGGIDHAKYTGSLTTLPLTSNREFTIQTNSATVGTSTIDINTGL--------LLDSGTT 303

I GGIDH+ YTGSL P+ RE+ + V + D+ ++DSGTT

Sbjct 223 IIGGIDHSLYTGSLWYTPI--RREWYYEVIIVRVEINGQDLKMDCKEYNYDKSIVDSGTT 280

Query 304 LTYLPQSVVDSIANAI-GGDITYNRPIGAYIWS---CNRNGKVTYN-FP 347

LP+ V ++ +I T P G ++ C + G +N FP

Sbjct 281 NLRLPKKVFEAAVKSIKAASSTEKFPDGFWLGEQLVCWQAGTTPWNIFP 329

>Crystal Structure of human beta-secretase (BACE) in the presence of an inhibitor [Homo sapiens]

Sequence ID: 2HIZ_A Length: 455

>Crystal Structure of human beta-secretase (BACE) in the presence of an inhibitor [Homo sapiens]

Sequence ID: 2HIZ_B Length: 455 >Crystal Structure of human beta-secretase (BACE) in the presence of an inhibitor [Homo sapiens]

Sequence ID: 2HIZ_C Length: 455 >Potent and selective isophthalamide S2 hydroxyethylamine inhibitor of BACE1 [Homo sapiens]

Sequence ID: 2P83_A Length: 455 >Potent and selective isophthalamide S2 hydroxyethylamine inhibitor of BACE1 [Homo sapiens]

Sequence ID: 2P83_B Length: 455 >Potent and selective isophthalamide S2 hydroxyethylamine inhibitor of BACE1 [Homo sapiens]

Sequence ID: 2P83_C Length: 455

Range 1: 77 to 344

Score:55.8 bits(133), Expect:2e-07,

Method:Compositional matrix adjust.,

Identities:77/289(27%), Positives:117/289(40%), Gaps:58/289(20%)

Query 96 YSANFTVGSNSQKQNVIVDTGSSDLWVVDSSANCQEKSGYSSDYCFSGGTYDPSSSSTIQ 155

Y TVGS Q N++VDTGSS+ V ++ + F Y SST +

Sbjct 77 YYVEMTVGSPPQTLNILVDTGSSNFAVG------------AAPHPFLHRYYQRQLSSTYR 124

Query 156 ELGKSFNIRYGDGSSSSGTWVKD--------------TVGINGAIILNQQFGDVNSTSVS 201

+L K + Y + G W + TV N A I +N ++

Sbjct 125 DLRKGVYVPY-----TQGKWEGELGTDLVSIPHGPNVTVRANIAAITESDKFFINGSNW- 178

Query 202 QGILGIGLDTNESTDTIYENFPINLKEQGFINTNAYSLYLNAP----------SATSGTI 251

+GILG+ D E F +L +Q + N +SL L ++ G++

Sbjct 179 EGILGLAYAEIARPDDSLEPFFDSLVKQTHV-PNLFSLQLCGAGFPLNQSEVLASVGGSM 237

Query 252 IFGGIDHAKYTGSLTTLPLTSNREFTIQTNSATVGTSTIDINTGL--------LLDSGTT 303

I GGIDH+ YTGSL P+ RE+ + V + D+ ++DSGTT

Sbjct 238 IIGGIDHSLYTGSLWYTPI--RREWYYEVIIVRVEINGQDLKMDCKEYNYDKSIVDSGTT 295

Query 304 LTYLPQSVVDSIANAI-GGDITYNRPIGAYIWS---CNRNGKVTYN-FP 347

LP+ V ++ +I T P G ++ C + G +N FP

Sbjct 296 NLRLPKKVFEAAVKSIKAASSTEKFPDGFWLGEQLVCWQAGTTPWNIFP 344

>X-ray crystal structure of beta secretase complexed with compound 8c [Homo sapiens]

Sequence ID: 2VA5_A Length: 455

>X-ray crystal structure of beta secretase complexed with compound 24 [Homo sapiens]

Sequence ID: 2VA6_A Length: 455 >X-ray crystal structure of beta secretase complexed with compound 27 [Homo sapiens]

Sequence ID: 2VA7_A Length: 455

Range 1: 77 to 344

Score:55.8 bits(133), Expect:2e-07,

Method:Compositional matrix adjust.,

Identities:77/289(27%), Positives:117/289(40%), Gaps:58/289(20%)

Query 96 YSANFTVGSNSQKQNVIVDTGSSDLWVVDSSANCQEKSGYSSDYCFSGGTYDPSSSSTIQ 155

Y TVGS Q N++VDTGSS+ V ++ + F Y SST +

Sbjct 77 YYVEMTVGSPPQTLNILVDTGSSNFAVG------------AAPHPFLHRYYQRQLSSTYR 124

Query 156 ELGKSFNIRYGDGSSSSGTWVKD--------------TVGINGAIILNQQFGDVNSTSVS 201

+L K + Y + G W + TV N A I +N ++

Sbjct 125 DLRKGVYVPY-----TQGKWEGELGTDLVSIPHGPNVTVRANIAAITESDKFFINGSNW- 178

Query 202 QGILGIGLDTNESTDTIYENFPINLKEQGFINTNAYSLYLNAP----------SATSGTI 251

+GILG+ D E F +L +Q + N +SL L ++ G++

Sbjct 179 EGILGLAYAEIARPDDSLEPFFDSLVKQTHV-PNLFSLQLCGAGFPLNQSEVLASVGGSM 237

Query 252 IFGGIDHAKYTGSLTTLPLTSNREFTIQTNSATVGTSTIDINTGL--------LLDSGTT 303

I GGIDH+ YTGSL P+ RE+ + V + D+ ++DSGTT

Sbjct 238 IIGGIDHSLYTGSLWYTPI--RREWYYEVIIVRVEINGQDLKMDCKEYNYDKSIVDSGTT 295

Query 304 LTYLPQSVVDSIANAI-GGDITYNRPIGAYIWS---CNRNGKVTYN-FP 347

LP+ V ++ +I T P G ++ C + G +N FP

Sbjct 296 NLRLPKKVFEAAVKSIKAASSTEKFPDGFWLGEQLVCWQAGTTPWNIFP 344

>Structure of BACE complexed to compound 3a [Homo sapiens]

Sequence ID: 2Q15_A Length: 385

Range 1: 14 to 281

Score:55.8 bits(133), Expect:3e-07,

Method:Compositional matrix adjust.,

Identities:78/286(27%), Positives:115/286(40%), Gaps:52/286(18%)

Query 96 YSANFTVGSNSQKQNVIVDTGSSDLWVVDSSANCQEKSGYSSDYCFSGGTYDPSSSSTIQ 155

Y TVGS Q N++VDTGSS+ V ++ + F Y SST +

Sbjct 14 YYVEMTVGSPPQTLNILVDTGSSNFAVG------------AAPHPFLHRYYQRQLSSTYR 61

Query 156 ELGKSFNIRYGDGSSSSGTWVKDTVGIN-----------GAIILNQQFGDVNSTSVSQGI 204

+L K + Y G G D V I AI + +F S +GI

Sbjct 62 DLRKGVYVPYTQGKWE-GELGTDLVSIPHGPQVTVRANIAAITESDKF--FIQGSNWEGI 118

Query 205 LGIGLDTNESTDTIYENFPINLKEQGFINTNAYSLYL----------NAPSATSGTIIFG 254

LG+ D E F +L +Q + N +SL L ++ G++I G

Sbjct 119 LGLAYAEIARPDDSLEPFFDSLVKQTHV-PNLFSLQLCGAGFPLQQSEVLASVGGSMIIG 177

Query 255 GIDHAKYTGSLTTLPLTSNREFTIQTNSATVGTSTIDINTGL--------LLDSGTTLTY 306

GIDH+ YTGSL P+ RE+ + V + D+ ++DSGTT

Sbjct 178 GIDHSLYTGSLWYTPI--RREWYYEVIIVRVEINGQDLKMDCKEYNYDKSIVDSGTTNLR 235

Query 307 LPQSVVDSIANAI-GGDITYNRPIGAYIWS---CNRNGKVTYN-FP 347

LP+ V ++ +I T P G ++ C + G +N FP

Sbjct 236 LPKKVFEAAVKSIKAASSTEKFPDGFWLGEQLVCWQAGTTPWNIFP 281

>Structure of BACE complexed to compound 1 [Homo sapiens]

Sequence ID: 2Q11_A Length: 388

>Structure of BACE complexed to compound 1 [Homo sapiens]

Sequence ID: 2Q11_B Length: 388 >Structure of BACE complexed to compound 1 [Homo sapiens]

Sequence ID: 2Q11_C Length: 388

Range 1: 17 to 284

Score:55.8 bits(133), Expect:3e-07,

Method:Compositional matrix adjust.,

Identities:78/286(27%), Positives:115/286(40%), Gaps:52/286(18%)

Query 96 YSANFTVGSNSQKQNVIVDTGSSDLWVVDSSANCQEKSGYSSDYCFSGGTYDPSSSSTIQ 155

Y TVGS Q N++VDTGSS+ V ++ + F Y SST +

Sbjct 17 YYVEMTVGSPPQTLNILVDTGSSNFAVG------------AAPHPFLHRYYQRQLSSTYR 64

Query 156 ELGKSFNIRYGDGSSSSGTWVKDTVGIN-----------GAIILNQQFGDVNSTSVSQGI 204

+L K + Y G G D V I AI + +F S +GI

Sbjct 65 DLRKGVYVPYTQGKWE-GELGTDLVSIPHGPQVTVRANIAAITESDKF--FIQGSNWEGI 121

Query 205 LGIGLDTNESTDTIYENFPINLKEQGFINTNAYSLYL----------NAPSATSGTIIFG 254

LG+ D E F +L +Q + N +SL L ++ G++I G

Sbjct 122 LGLAYAEIARPDDSLEPFFDSLVKQTHV-PNLFSLQLCGAGFPLQQSEVLASVGGSMIIG 180

Query 255 GIDHAKYTGSLTTLPLTSNREFTIQTNSATVGTSTIDINTGL--------LLDSGTTLTY 306

GIDH+ YTGSL P+ RE+ + V + D+ ++DSGTT

Sbjct 181 GIDHSLYTGSLWYTPI--RREWYYEVIIVRVEINGQDLKMDCKEYNYDKSIVDSGTTNLR 238

Query 307 LPQSVVDSIANAI-GGDITYNRPIGAYIWS---CNRNGKVTYN-FP 347

LP+ V ++ +I T P G ++ C + G +N FP

Sbjct 239 LPKKVFEAAVKSIKAASSTEKFPDGFWLGEQLVCWQAGTTPWNIFP 284

>Crystal structure of the human BACE1 catalytic domain in complex with 4-(4-fluoro-benzyl)-piperazine-2-carboxylic acid (2-mercapto-ethyl)-amide [Homo sapiens]

Sequence ID: 2ZJJ_A Length: 405

Range 1: 34 to 301

Score:55.8 bits(133), Expect:3e-07,

Method:Compositional matrix adjust.,

Identities:76/289(26%), Positives:116/289(40%), Gaps:58/289(20%)

Query 96 YSANFTVGSNSQKQNVIVDTGSSDLWVVDSSANCQEKSGYSSDYCFSGGTYDPSSSSTIQ 155

Y TVGS Q N++VDTGSS+ V ++ + F Y SST +

Sbjct 34 YYVEMTVGSPPQTLNILVDTGSSNFAVG------------AAPHPFLHRYYQRQLSSTYR 81

Query 156 ELGKSFNIRYGDGSSSSGTWVKD--------------TVGINGAIILNQQFGDVNSTSVS 201

+L K + Y + G W + TV N A I +N ++

Sbjct 82 DLRKGVYVPY-----TQGAWAGELGTDLVSIPHGPNVTVRANIAAITESDKFFINGSNW- 135

Query 202 QGILGIGLDTNESTDTIYENFPINLKEQGFINTNAYSLYL----------NAPSATSGTI 251

+GILG+ D E F +L +Q + N +SL L ++ G++

Sbjct 136 EGILGLAYAEIARPDDSLEPFFDSLVKQTHV-PNLFSLQLCGAGFPLNQSEVLASVGGSM 194

Query 252 IFGGIDHAKYTGSLTTLPLTSNREFTIQTNSATVGTSTIDINTGL--------LLDSGTT 303

I GGIDH+ YTGSL P+ RE+ + V + D+ ++DSG T

Sbjct 195 IIGGIDHSLYTGSLWYTPI--RREWYYEVIIVRVEINGQDLKMDCKEYNYDKSIVDSGCT 252

Query 304 LTYLPQSVVDSIANAI-GGDITYNRPIGAYIWS---CNRNGKVTYN-FP 347

LP+ V ++ +I T P G ++ C + G +N FP

Sbjct 253 NLRLPKKVFEAAVKSIKAASSTEKFPDGFWLGEQLVCWQAGTTPWNIFP 301
