# Supplementary material for: Design of a multi-epitope vaccine against the pathogenic fungi Candida tropicalis using an in silico approach
Source: J Genet Eng Biotechnol. 2022 Sep 29;20:140. doi: 10.1186/s43141-022-00415-3 (PMC9521867; doi:10.1186/s43141-022-00415-3)
Supplement: Supplementary file 5 — Additional file 5: Table S5. Relative surface accessibility of the amino acid residues in the epitope of the designed vaccine candidate. [file 43141_2022_415_MOESM5_ESM.docx]

Supplementary Table S5: Relative surface accessibility of the amino acid residues in the epitope of the designed vaccine candidate

| Epitope | Residue | Relative surface accessibility |
| --- | --- | --- |
| **YNRPIGAYI** |  |  |
|  | Y | Buried |
|  | N | Exposed |
|  | R | Buried |
|  | P | Exposed |
|  | I | Buried |
|  | G | Buried |
|  | A | Exposed |
|  | Y | Exposed |
|  | I | Exposed |
| **FTIQTNSAT** | F | Buried |
|  | T | Exposed |
|  | I | Buried |
|  | Q | Exposed |
|  | T | Exposed |
|  | N | Exposed |
|  | S | Exposed |
|  | A | Buried |
|  | T | Exposed |
| **EFTIQTNSA** | E | Exposed |
|  | F | Buried |
|  | T | Exposed |
|  | I | Buried |
|  | Q | Exposed |
|  | T | Buried |
|  | N | Exposed |
|  | S | Exposed |
|  | A | Exposed |
| **ILYGENFNI** |  |  |
|  | I | Buried |
|  | L | Buried |
|  | Y | Buried |
|  | G | Exposed |
|  | E | Exposed |
|  | N | Exposed |
|  | F | Buried |
|  | N | Exposed |
|  | I | Buried |
| **QELGKSFNI** |  |  |
|  | Q | Exposed |
|  | E | Exposed |
|  | L | Exposed |
|  | G | Exposed |
|  | K | Exposed |
|  | S | Exposed |
|  | F | Buried |
|  | N | Exposed |
|  | I | Buried |
| **GLMGNFFDK** |  |  |
|  | G | Exposed |
|  | L | Buried |
|  | M | Exposed |
|  | G | Buried |
|  | N | Exposed |
|  | F | Buried |
|  | F | Exposed |
|  | D | Exposed |
|  | K | Exposed |
| **KYTGSLTTL** |  |  |
|  | K | Exposed |
|  | Y | Exposed |
|  | T | Exposed |
|  | G | Exposed |
|  | S | Exposed |
|  | L | Buried |
|  | T | Exposed |
|  | T | Exposed |
|  | L | Exposed |
| **DTVGINGAI** |  |  |
|  | D | Exposed |
|  | T | Exposed |
|  | V | Exposed |
|  | G | Exposed |
|  | I | Exposed |
|  | N | Exposed |
|  | G | Exposed |
|  | A | Exposed |
|  | I | Exposed |
| **LPLTSNREF** |  |  |
|  | L | Exposed |
|  | P | Exposed |
|  | L | Exposed |
|  | T | Exposed |
|  | S | Exposed |
|  | N | Exposed |
|  | R | Exposed |
|  | E | Exposed |
|  | F | Exposed |
| **AKYTGSLTTL** |  |  |
|  | A | Exposed |
|  | K | Exposed |
|  | Y | Buried |
|  | T | Exposed |
|  | G | Exposed |
|  | S | Buried |
|  | L | Buried |
|  | T | Exposed |
|  | T | Exposed |
|  | L | Buried |
| **IGGDITYNRPIGAYIWSCNRNGK** |  |  |
|  | I | Buried |
|  | G | Exposed |
|  | G | Exposed |
|  | D | Exposed |
|  | I | Exposed |
|  | T | Exposed |
|  | Y | Exposed |
|  | N | Buried |
|  | R | Exposed |
|  | P | Exposed |
|  | I | Buried |
|  | G | Buried |
|  | A | Exposed |
|  | Y | Buried |
|  | I | Buried |
|  | W | Exposed |
|  | S | Buried |
|  | C | Buried |
|  | N | Exposed |
|  | R | Exposed |
|  | N | Exposed |
|  | G | Exposed |
|  | K | Exposed |
